# Switching Shapes: Reversible Three Species Photoisomerization of Substituted 1,2-Dihydro-1,2-azaborinines

**DOI:** 10.1021/jacs.5c20667

**Published:** 2026-02-09

**Authors:** Sonja M. Biebl, Jonas N. Lienert, Adrian J. Müller, Markus Ströbele, Andreas Dreuw, Josef Wachtveitl, Holger F. Bettinger

**Affiliations:** † Institut für Organische Chemie, 9188 Eberhard Karls Universität Tübingen, Auf der Morgenstelle 18, 72076 Tübingen, Germany; ‡ Institut für Physikalische und Theoretische Chemie, Goethe-Universität Frankfurt, Max von Laue-Straße 7, 60438 Frankfurt am Main, Germany; § Institut für Anorganische Chemie, Eberhard Karls Universität Tübingen, Auf der Morgenstelle 18, 72076 Tübingen, Germany; ∥ Interdisciplinary Center for Scientific Computing, 9144 Heidelberg University, Im Neuenheimer Feld 205, 69120 Heidelberg, Germany

## Abstract

Derivatives of 1,2-dihydro-1,2-azaborinines generally undergo selective photochemical electrocyclic ring-closure reactions to the corresponding Dewar isomers (2-aza-3-borabicyclo[2.2.0]­hex-5-ene). Depending on the substitution pattern, these photoreactions can also yield benzvalene (3-aza-4-boratricyclo­[3.1.0.0^2.6^]­hexane) analogues. Here, we report the synthesis of 1,2,3,5-tetrasubstituted dihydroazaborinines by transition-metal-catalyzed late-stage functionalization and the investigation of their photophysical and photochemical properties using transient absorption spectroscopy. The introduction of aryl groups at the 3- and 5-positions induces a pronounced bathochromic shift of the absorption maximum. Under broad-spectrum irradiation (280–400 nm), quantitative conversion to the benzvalene isomer can be achieved. The initial photoisomerization proceeds via excitation to the short-lived singlet excited state (S_1_) yielding the Dewar isomer, whereas the subsequent conversion of this intermediate occurs through a long-lived excited state. Notably, the second isomerization step is accompanied by an interchange of the carbons C3 and C4. Once formed, the benzvalene isomers exhibit exceptional thermal stability. Cycloreversion to the Dewar isomer and even to the dihydroazaborinine structure can be triggered photochemically through targeted excitation and during both processes the substituents return to the C3 and C5 positions. The thermal cycloreversion of the benzvalene isomer can yield either the educt BN-benzene isomer (1,2,3,5-substitued) or its 1,2,4,5-substituted isomer. Computational studies revealed a stepwise mechanism for the thermal back reaction reforming the educt, while a concerted, energetically less-favorable pathway leads to the 1,2,4,5-substituted analogue.

## Graphical abstract

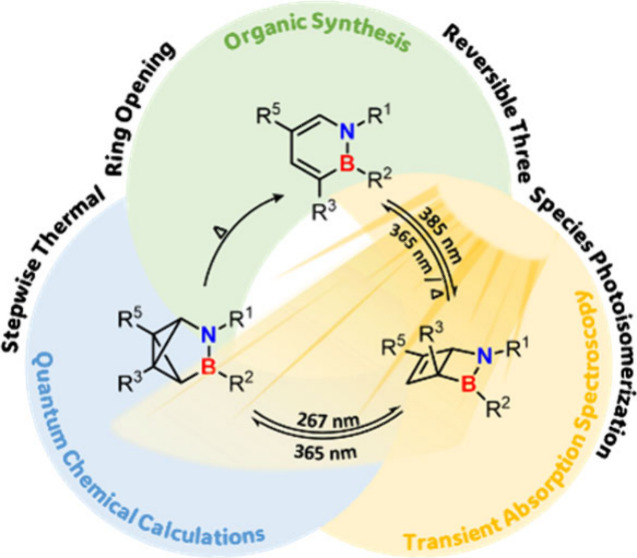

## Introduction

The photoisomerization of benzene (**B**) to various polycyclic, nonaromatic isomers gained considerable attention from both experimental^1–6^ and theoretical^7–9^ perspectives. These investigations offer profound insights into the excited-state behavior of conjugated π systems and have significant implications for understanding reaction mechanisms and designing photo responsive materials. In particular, isomerizations to Dewar benzene (bicyclo[2.2.0]­hexa-2,5-diene, **D**), benzvalene (tricyclo­[3.1.0.0^2.6^]­hexa-3-ene, **V**), fulvene (5-methylene-1,3-cyclopentadiene, **F**), and prismane (tetracyclo[2.2.0.0.0]­hexane, **P**) were observed and investigated (Scheme 1).^10^

**Scheme 1:**
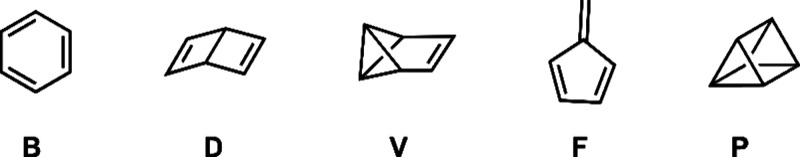
Benzene (**B**) and Its Four Most-Studied Isomers: Dewar Benzene (**D**), Benzvalene (**V**), Fulvene (**F**) and Prismane (**P**) Isomers

Due to the loss of aromatic stabilization and in some cases significant ring strain, these isomers lie energetically well above benzene.^8,11^ Nevertheless, these photo reactions proceed due to S_1_/T_1_-state antiaromaticity relief^12–14^ and some of these species are reasonably stable, as thermal, orbital symmetry-allowed cycloreversions would lead to unfavorable products. For instance, the thermally allowed conrotatory ring-opening of Dewar benzene would result in the formation of the unknown highly strained trans-benzene (*cis*,*cis*,*trans*-cyclohexa-1,3,5-triene), and thus the forbidden disrotatory path to benzene is slowly proceeding.^11,15,16^ Depending on phase and excitation wavelength, different ratios of isomers are retrieved.^2,17^ Still, it must be noted that the quantum yield of such isomerizations is typically very low (<0.05%) and benzene itself remains the predominant species in the reaction mixture.^2^

This behavior changes fundamentally upon isoelectronic replacement of a CC unit in the benzene ring with a BN pair.^18^ The resulting 1,2-dihydro-1,2-azaborinines (^***BN***^ **B**) undergo selective and almost quantitative photoisomerization reactions.^18–20^ Such reactivity was first observed in 2012 by Bettinger, Liu et al. during cryogenic matrix isolation experiments investigating the unsubstituted parent compound.^18^ Upon UV irradiation at 254 nm, exclusively the formation of the Dewar isomer (^***BN***^ **D**) was observed.^18^ Similar behavior was later on demonstrated for 1,2- and 1,2,3-substituted dihydroazaborinines (^***BN***^ **B1**) under irradiation in the 280–400 nm range (Scheme 2a).^19–21^ Moreover, steric effects introduced by the substituents increase the kinetic stability of the Dewar isomers (^***BN***^ **D1**) toward undesired side reactions, such as di- or oligomerization.^19,22^ Under standard conditions these ^***BN***^ **D1** persist for up to half a year.^19,20,23^

**Scheme 2:**
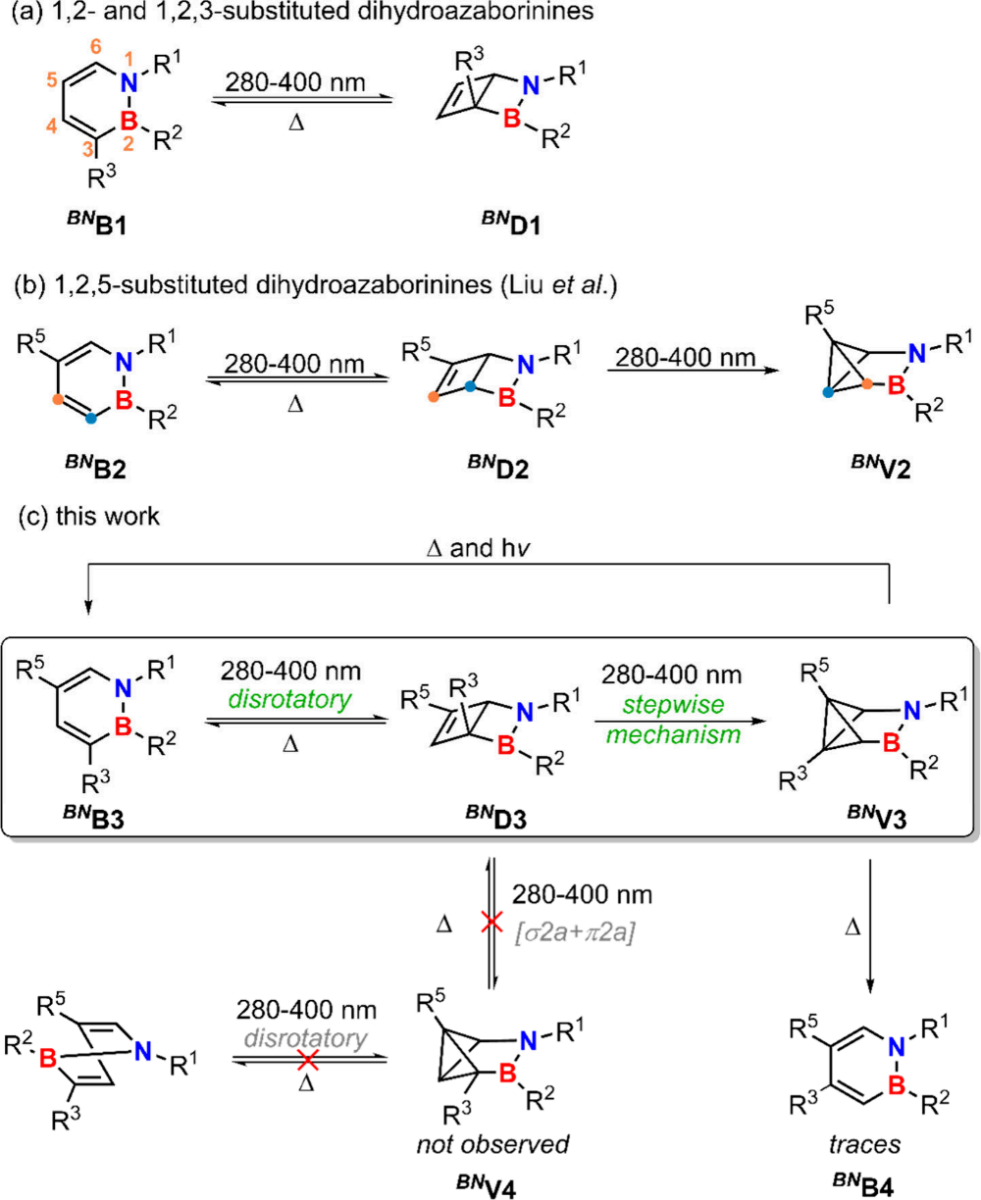
Photoisomerization Behavior of 1,2-Dihydro-1,2-azaborinines (^***BN***^ **B**) with Varying Substitution Patterns^a^

a The substituents R^3^ and R^5^ at the carbon backbones are aryl substituents in all examples shown. The pericyclic reactions written in grey generate ^**BN**^ **V4** in an orbital symmetry allowed fashion but are not observed experimentally.

In 2024, Liu and co-workers reported the selective formation of a benzvalene isomer (^***BN***^ **V2**) employing 1,2,5-substituted species under UV irradiation (280–400 nm).^24,43^ Their experiments suggest that the benzvalene isomer is not formed directly from the 1,2,5-substituted dihydroazaborinine, but rather arises as a subsequent reaction product of the initially formed Dewar isomer (^***BN***^ **D2**).^24^ This mechanistic proposal is supported by deuterium labeling at C3, which reveals an interchange of the C3 and C4 positions over the course of the isomerization (Scheme 2b).^24^ This observation cannot be accounted for by any electrocyclization pathway, irrespective of it being allowed or forbidden under the Woodward–Hoffmann rules.^11^ As illustrated in Scheme 2c, the direct photoisomerization of a dihydroazaborinine to benzvalene necessitates substantial distortion toward a *s*-*trans*-^***BN***^ **B** conformation to enable the constructive orbital overlap for a symmetry-allowed [π2s+π2s] cyclization and is unable to achieve the C3 to C4 deuterium migration.^11^ The same applies for the electrocyclization of the cyclobutene moiety in the Dewar isomer’s carbon backbone. While this transformation could theoretically proceed via a photochemical [σ2s+π2s] or [σ2a+π2a] pathway to yield the corresponding bicyclobutane derivative, it does not accommodate the observed carbon interchange.^11,25^

In case of ^***BN***^ **D1**, a thermal^19,20^ as well as a catalytic^23,26^ back reaction is feasible offering various applications in the field of molecular switches.^27–30^ Bettinger and Hauler identified a stepwise process using multiconfiguration SCF (CASSCF) and coupled cluster theory CCSD­(T) for the uncatalyzed ring opening reaction back to ^*BN*^ **B** (see Figure 1a).^31^ This mechanism is more favorable compared to the conventional con- or disrotatory pathways.^31^ However, bulky substituents at the boron and the nitrogen heteroatoms lead to a concerted mechanism of the thermal cycloreversion.^19^ Moreover a systematic, experimental study on 1,2,3-substituted ^***BN***^ **B1** revealed that the boron center plays a pivotal role in the electrocyclic ring opening by enabling a three-center-two-electron bond, which in turn allows tuning the electronic preference of the mechanism by substituents.^22^ Considering the benzvalene, no experimental data for the thermal ring opening of the ^***BN***^ **V** compound is available to date.

**Figure 1:**
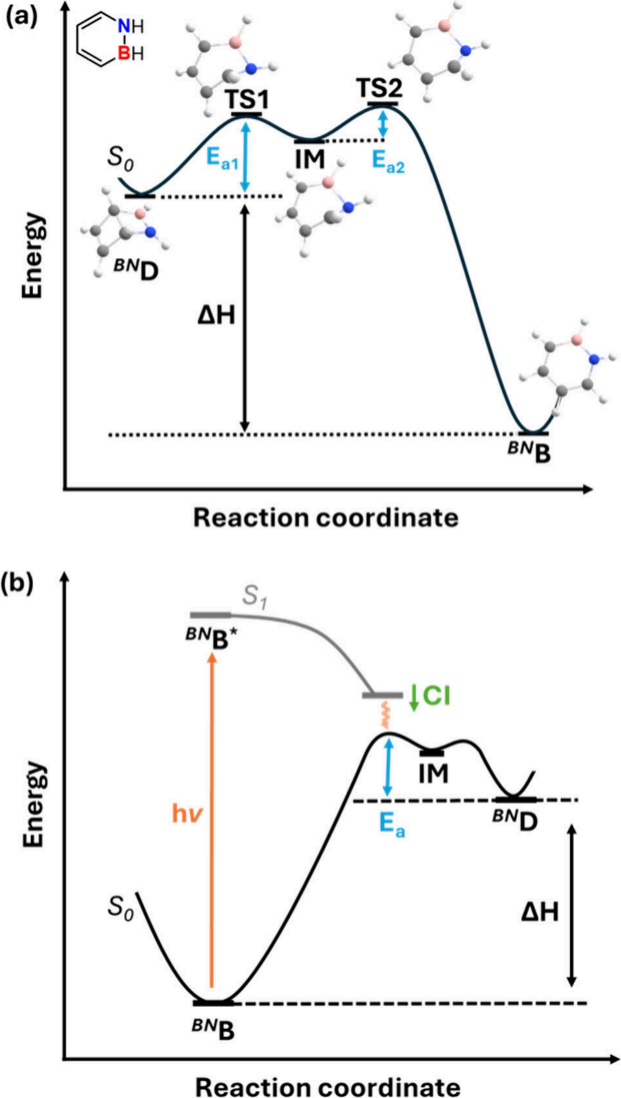
Mechanism of the thermal ring opening of 1,2-diyhdro-1,2-azaborinine (a) and energy profile of the photochemical rearrangement of the parent dihydroazaborinine (b). Both are based on calculations.^31–34^ The diagrams are not drawn to scale. TS1: first transition state, TS2: second transition state, IM: intermediate.

To enable a targeted application of the unique photochemical behavior, mechanistic insights into the photoisomerizations to ^***BN***^ **D** and ^***BN***^ **V** are essential. To date, no experimental data has been reported, and available insights were derived exclusively from theoretical investigations. According to multireference *ab initio* computations on the photoisomerization of the parent dihydroazaborinine Su et al. found that the isomerization is initiated by a vertical S_0_/S_1_ excitation to the Franck–Condon (FC) region.^32^ Subsequent radiation-free decay back to the S_0_ potential energy surface is feasible through a conical intersection, which offers access to ground state relaxation paths to the reactant or ^***BN***^ **D** (Figure 1b).^32^ Additional investigations focusing on the landscape of the excited state show that the S_1_ PES is flat in the FC region with a negligible barrier of 0.5 kcal mol^–1^ along the minimum energy path of the isomerization.^33,34^ As the S_1_/S_0_ conical intersection (CI) is lower in energy than the S_1_ FC point by 23.8 kcal mol^–1^ (in case of the parent species) a reasonable driving force toward the formation of ^***BN***^ **D** is given.^34,35^ A recent DFT study suggests that the subsequent isomerization to ^***BN***^ **V** also proceeds via the S_1_ potential energy surface.^36^ The transient species calculated can exhibit radical like behavior, however, negligible spin orbit coupling values disfavor contributions from triplet interactions.^36^ Overall the critical bond rearrangements do not appear to be mediated by these radical centers, but rather occur as a consequence of the electronic redistribution toward the conical intersection.^36^ Beyond these high-level computations, experimental insights into the mechanism of both photochemical isomerizations (^***BN***^ **D** and ^***BN***^ **V**) are not available.

We synthesized and characterized a series of 1-(*tert*-butyldimethylsilyl)-2-pentamethylphenyl-3,5-*bis*-aryl (^***BN***^ **B3**) substituted compounds and found that the nature of the substituent affects the UV–vis absorption of the ^***BN***^ **B3** ( (Scheme 1). Similar to the behavior of 1,2,5-trisubstituted species the photoisomerization proceeds through the ^***BN***^ **D3** isomer to ^***BN***^ **V3** as the final photoproduct (280–400 nm).^21^ Depending on the irradiation wavelength quantitative conversion to the benzvalene or photostationary equilibria consisting of ^***BN***^ **B**, ^***BN***^ **D** and ^***BN***^ **V** are observed. The excited-state dynamics of the photoisomerization was examined for the *p*-(dimethylaminophenyl) derivative by femtosecond transient absorption spectroscopy. Such investigations are essential for advancing the mechanistic understanding of the isomerization process and thus improve property predictions. In addition, plausible thermal back reactions in agreement with the experiment were investigated employing DFT calculations.

## Results and Discussion

### Synthesis

^***BN***^ **B5** was synthesized based on a previously published protocol (Scheme 3a, for details see SI).^22^ Its 2-fold bromination is selective toward the carbon positions C3 (first bromination) and C5 (second bromination) and achieved using elemental bromine.^22,37,38^ A key factor for a clean synthesis devoid of side products is the presence of the pentamethylphenyl substituent (Ph*) at the boron center. Unlike other sterically demanding groups such as mesityl, it cannot undergo competing electrophilic aromatic substitution processes. The brominated carbon position of ^***BN***^ **B6** could be assigned by NMR. Moreover, these NMR spectra are in excellent agreement with 2-fold brominated species just differing in the R^2^ at boron.^22^ For these species the position of the bromination was proven by single crystal diffraction in our previous work.^22^

**Scheme 3:**
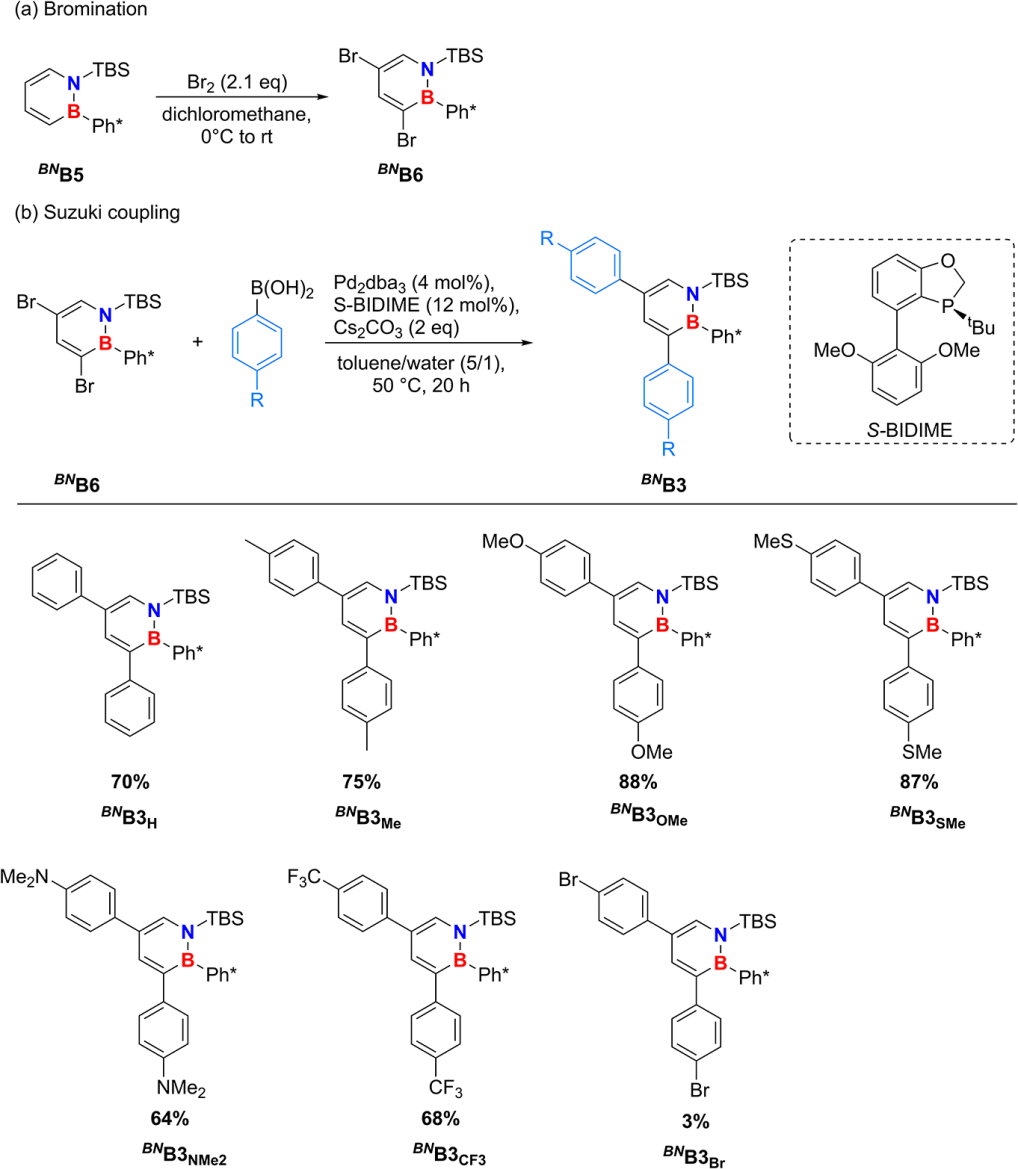
Synthesis of the Brominated Dihydroazaborinine ^***BN***^ **B6** and Its Conversion in a Suzuki Cross-Coupling Reaction Affording 3,5-Diaryl-Substituted ^***BN***^ **B3** Starting from Literature-Known ^***BN***^ **B5** ^a^

a Ph*: pentamethylphenyl.

To achieve the arylation of C3 and C5, a Suzuki cross coupling protocol that we previously developed for the functionalization of the C3 position was applicable (Scheme 3b).^22^ Initial experiments with phenylboronic acid revealed the necessity to increase the catalyst and ligand loadings compared to the previous protocol by a factor of 2 as two consecutive cross-coupling steps are now required.^22^ As there are no further changes in the catalytic system apart from that, it still remains highly similar to a protocol for 2,1-BN-naphthalenes published earlier by Song and co-workers.^39^

To evaluate the applicability of this method, cross-coupling reactions were performed using various boronic acids bearing different para-substituents. The system exhibited excellent tolerance toward boronic acids with electron-donating substituents (EDG). In contrast, significantly lower yields were obtained with electron-withdrawing groups (EWG). Although the overall degree of conversion remained significantly lower than that observed for the EDG-substituted derivatives, ^***BN***^ **B3**_**CF3**_ could be isolated after size-exclusion chromatography. As expected, the use of *p*-bromophenylboronic acid resulted in the formation of numerous side products, primarily due to homocoupling processes. Despite this, small amounts of the desired product ^***BN***^ **B3**_**Br**_ could still be isolated after multiple purification steps.

In case of ^***BN***^ **B5** and ^***BN***^ **B3**_**H**_, single crystals suitable for X-ray diffraction were grown by vapor diffusion (Figure 2). For both structures the heterocycle and the pentamethylphenyl substituent are approximately perpendicular to each other. This is reflected in a C3–B–C11-C12 dihedral angle of 90.9° for ^***BN***^ **B5** and 85.2° for ^***BN***^ **B3**_**H**_. Moreover, the boron substituent in ^***BN***^ **B3**_**H**_ is almost parallel to the phenyl ring at C3 (dihedral C11–C12–C18-C19 = 18.5 °). This indicates π-π interactions between these moieties. The phenyl rings exhibit inclinations of 45.3° (C5; C4–C5–C22-C23 dihedral) and 49.2° (C3; B–C3–C18-C19 dihedral) relative to the central heteroaromatic ring.

**Figure 2:**
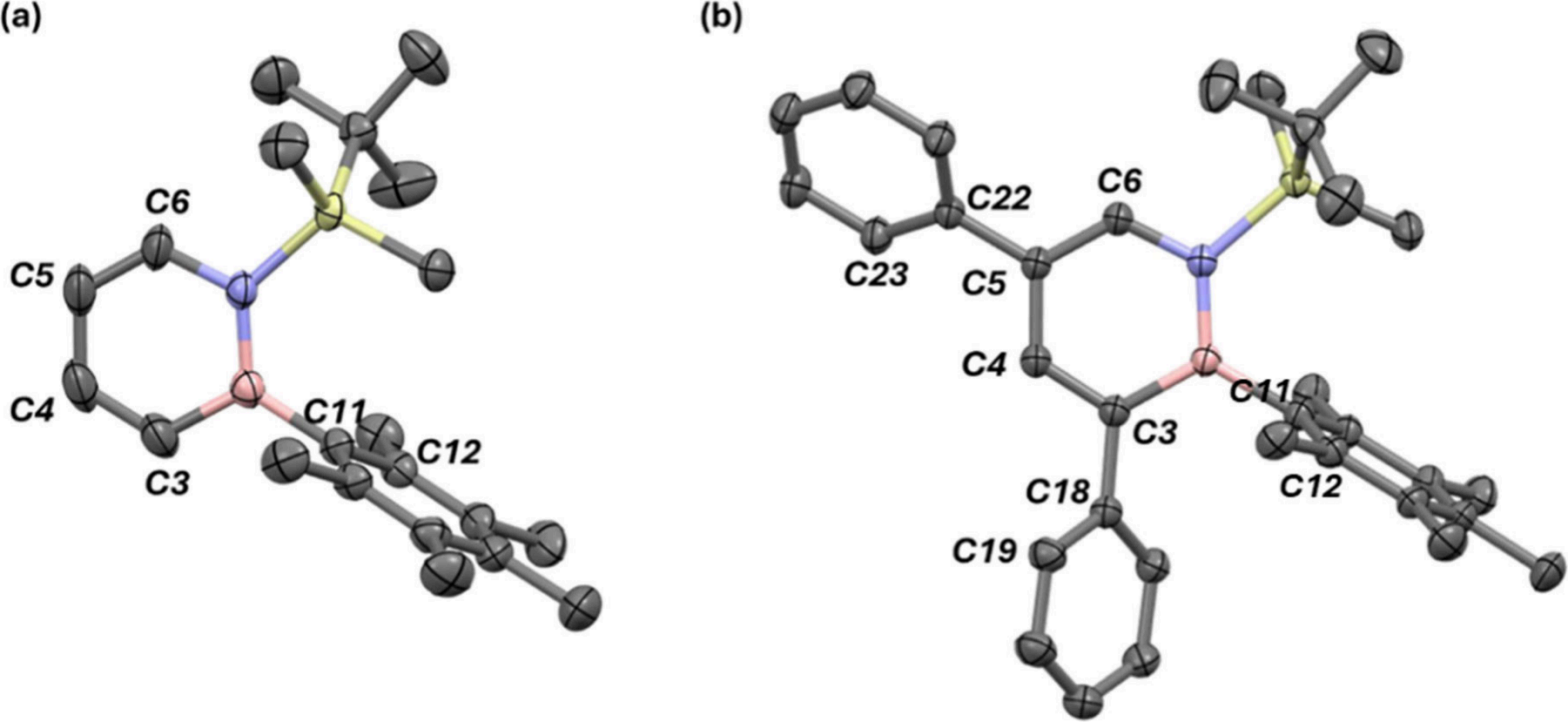
Molecular structures of ^***BN***^ **B5** (CCDC 2428116) and ^***BN***^ **B3**_**H**_ (CCDC 2424411) in the solid state of the different dihydroazaborinines synthesized. Hydrogen atoms are omitted for clarity. Thermal ellipsoids are drawn at the 50% probability level.

### UV–vis Spectra

Most of the coupling products (^***BN***^ **B3**) are colorless solids, while ^***BN***^ **B3**_**NMe2**_ has a pale-yellow tone. This observation is also reflected in the UV–vis absorption spectra of the compounds. All molecules show three distinct absorption bands whose relative intensities vary with different substitution patterns. With increasing electron-donating character of the para-substituent (Figure 3), the absorption maximum undergoes a progressive bathochromic shift. Although ^***BN***^ **B3**_**NMe2**_ and ^***BN***^ **B3**_**SMe**_ display similar primary absorption maxima, ^***BN***^ **B3**_**NMe2**_ features a significantly red-shifted secondary band, which extends just into the visible region.

**Figure 3:**
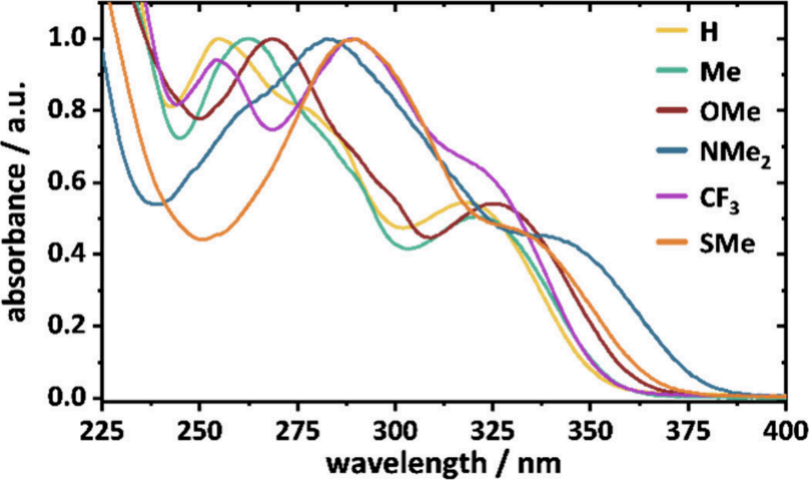
UV–vis absorption spectra for the 3,5-arylated ^***BN***^ **B 3** synthesized using the Suzuki cross coupling protocol described above. The legend gives the para substituent of the phenyl ring attached to C3 and C5. The absorption maxima are normalized, setting the main peak to 1 a.u.

Interestingly, the electron-withdrawing CF_3_ substituent likewise causes a noticeable bathochromic shift, yielding absorption maxima similar to those of ^***BN***^ **B3**_**SMe**_. A similar trend can be observed experimentally for C3-substituted ^***BN***^ **B** compounds^22^ and is also supported by theoretical investigation where both, push and pull substituents display red-shifted absorption.^40^

### Fluorescence Spectra

Emission spectra of the amine-substituted compounds ^***BN***^ **B3**_**NMe2**_ and ^***BN***^ **D3**_**NMe2**_ (see below for its formation) were measured in cyclohexane (cy) and tetrahydrofuran (THF). Both isomers display solvatochromism (Figure 4). The emission maximum of ^***BN***^ **B3**_**NMe2**_ shifts from 415 nm in the nonpolar solvent (cy) to 445 nm and less pronounced from 405 to 420 nm for ^***BN***^ **D3**_**NMe2**_. This effect can be attributed to a more polar excited state than the ground state.

**Figure 4:**
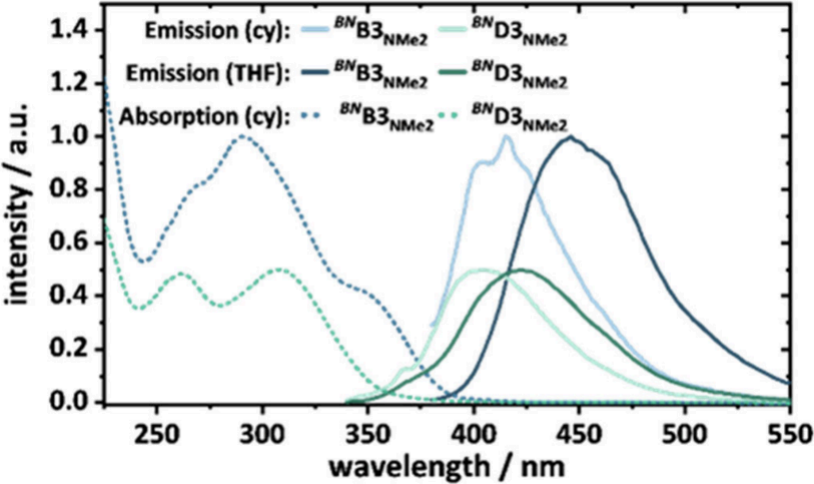
Emission spectra of ^***BN***^ **B3**_**NMe2**_ (blue) and ^***BN***^ **D3**_**NMe2**_ (green, prepared as the 385 nm photostationary state) in cyclohexane (light) and tetrahydrofuran (dark). Absorption spectra in cyclohexane (cy) of both compounds are given as a reference. All ^***BN***^ **B3**_**NMe2**_ spectra are normalized to 1 and all ^***BN***^ **D3**_**NMe2**_ are normalized to 0.5 for clarity.

### Photoisomerization

UV irradiation (280–400 nm, mercury lamp) of 0.05 to 0.1 M solutions of the different dihydroazaborinines ^***BN***^ **B3** in cyclohexane led to the formation of the corresponding ^***BN***^ **D3** isomers. Once small amounts of ^***BN***^ **D3** were present in solution, the formation of the corresponding ^***BN***^ **V3** derivative became apparent. This is particularly evident from two characteristic ^1^H NMR signals which, in comparison with the data reported by Liu et al.,^24^ can be assigned to a ^***BN***^ **V3** structure. Upon an extended irradiation in the wavelength range of 280–400 nm, complete conversion to ^***BN***^ **V3** was achieved. The concentration profiles of ^***BN***^ **B3**_**H**_, ^***BN***^ **D3**_**H**_, and ^***BN***^ **V3**_**H**_, monitored by NMR spectroscopy, were consistent with a consecutive reaction mechanism in which ^***BN***^ **D3**_**H**_ acts as an intermediate (Figure 5a).

**Figure 5:**
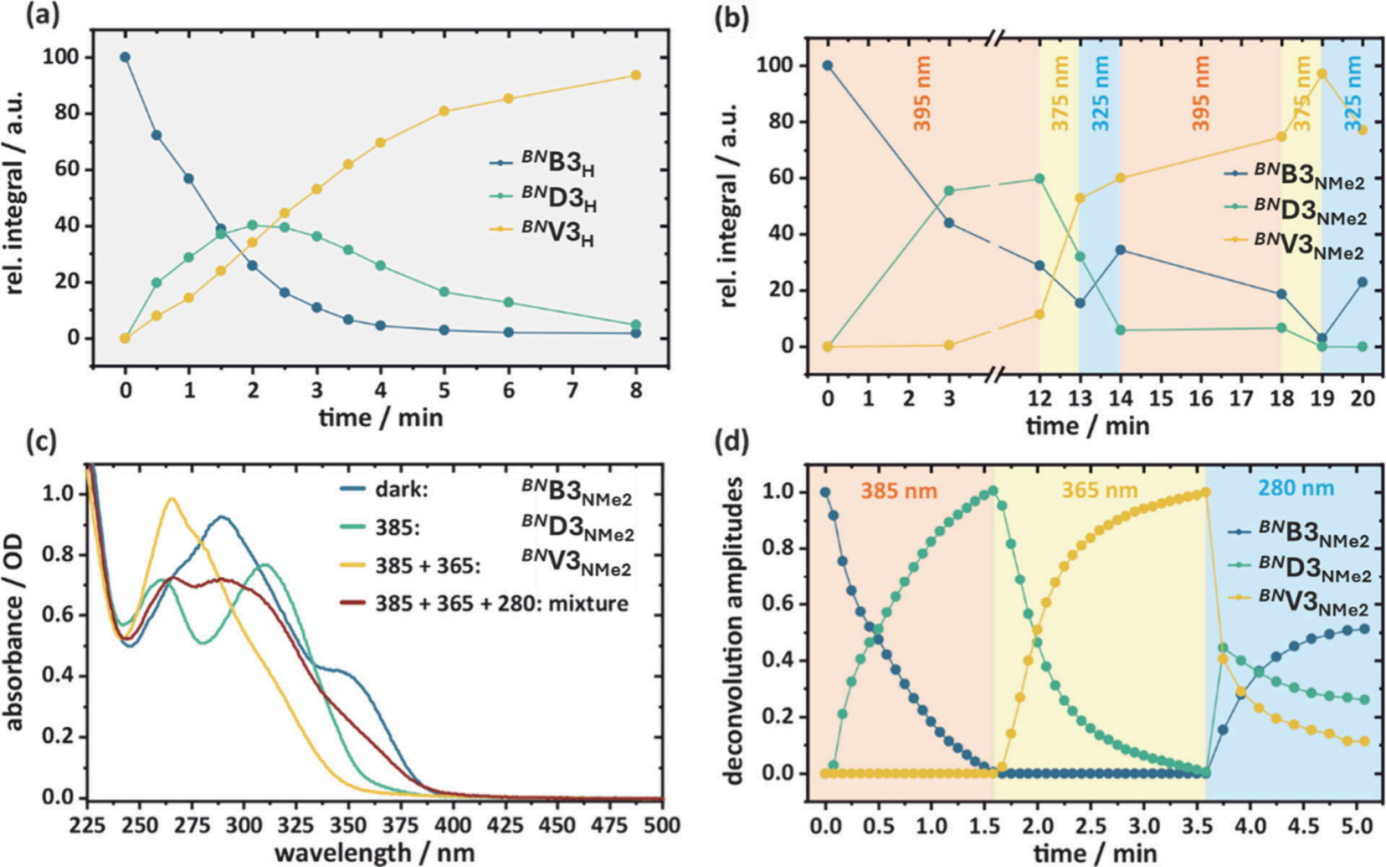
Ratios of ^***BN***^ **B3**, ^***BN***^ **D3**, and ^***BN***^ **V3** determined by ^1^H NMR spectroscopy during irradiation with different wavelengths. (a) Quantitative conversion to ^***BN***^ **V3**_**H**_ under broadband irradiation (280–400 nm) for the example of ^***BN***^ **B3**_**H**_. (b) Long-wavelength excitation of ^***BN***^ **B3**_**NMe2**_ to a photostationary mixture and subsequent irradiation with higher-energy light to ^***BN***^ **B3**_**NMe2**_ using a 325 nm LED. (c) Spectra after illumination with 385 nm, 365 and 280 nm LEDs. Purity of spectra was determined by spectral deconvolution. The deconvolution amplitudes to the corresponding photostationary states in (c) are plotted in (d).

To determine whether this secondary transformation is of thermal or photochemical nature, a mixture of the three isomers, generated by five minutes of irradiation, was heated to 50 °C for one hour. Under these conditions, only a slight decrease of the signals of ^***BN***^ **D3**_**H**_ was observed, accompanied by a corresponding increase of the signals of ^***BN***^ **B3**_**H**_. The intensity of the ^1^H NMR signal of ^***BN***^ **V3**_**H**_ remained unchanged, indicating that the conversion of ^***BN***^ **D3**_**H**_ to ^***BN***^ **V3**_**H**_ cannot be attributed to thermal activation by residual heat from the light source.

Due to its bathochromically shifted absorption onset, ^***BN***^ **B3**_**NMe2**_ was selected for further investigation. When narrowband irradiation using LEDs was applied, complete conversion to ^***BN***^ **V3**_**NMe2**_ was no longer observed. Instead, wavelength-dependent photostationary equilibria were established. Irradiation near the absorption onset of ^***BN***^ **B3**_**NMe2**_ (395 nm) led to an increased formation of ^***BN***^ **D3**_**NMe2**_ compared to ^***BN***^ **V3**_**NMe2**_. Subsequent excitation with a 375 nm LED promoted conversion to ^***BN***^ **D3**_**NMe**_2__ and ^***BN***^ **V3**_**NMe2**_, resulting in a mixture of ^***BN***^ **B3**_**NMe2**_/^***BN***^ **D3**_**NMe2**_/^***BN***^ **V3**_**NMe2**_ with a ratio of 15/32/53 as determined by ^1^H NMR spectroscopy (Figure 5b). Targeted sequential irradiation with 395 nm and 375 nm LEDs generated ^***BN***^ **V3**_**NMe2**_ nearly quantitatively. Absorption spectra of all three individual isomers could be obtained through spectral deconvolution by following the NMR determined ratios (see SI, section 1 for a detailed explanation of the deconvolution mechanism). Figure 5c illustrates the switching after illumination with 385 nm, 365 nm, and 280 nm LEDs whereas the deconvolution amplitudes of the pure compounds are plotted in Figure 5d.

With 385 nm and 365 nm irradiation approximately quantitative conversion to ^***BN***^ **D3**_**NMe2**_ and, respectively, ^***BN***^ **V3**_**NMe2**_ could be reached, as supported by NMR experiments with TMS as internal standard (see SI for additional information). Furthermore, exciting ^***BN***^ **V3**_**NMe2**_ with 280 nm (Figure 5c) led to an unexpected increase in ^***BN***^ **D3**_**NMe2**_ and ^***BN***^ **B3**_**NMe2**_ absorption, clearly suggesting a photoinduced back reaction. NMR experiments with excitation at 325 nm confirm this trend and yield a ^***BN***^ **B3**_**NMe2**_/^***BN***^ **D3**_**NMe2**_/^***BN***^ **V3**_**NMe2**_ ratio of 34/6/60 (Figure 5b). Depending only on steady-state analysis, it remains unclear whether this back isomerization proceeds directly from ^***BN***^ **V3**_**NMe2**_ to ^***BN***^ **B3**_**NMe2**_ or involves ^***BN***^ **D3**_**NMe2**_.

### Transient Absorption Spectroscopy of ^**BN**^ **B3**_**NMe2**_

To gain insight into the switching mechanisms and participating intermediates, ultrafast transient absorption spectroscopy was carried out for ^***BN***^ **B3**_**NMe2**_ (Scheme 4). To isolate the ^***BN***^ **B3**_**NMe2**_ to ^***BN***^ **D3**_**NMe2**_ isomerization, a sample of ^***BN***^ **B3**_**NMe2**_ was excited with a 370 nm laser pulse (Figure 6a). UV and Vis parts were measured and fitted individually and scaled to match the corresponding infinity lifetime intensity (see SI section 1 for detailed explanation).

**Scheme 4:**
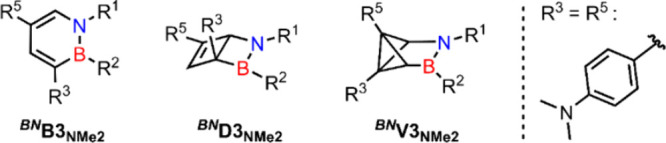
Structures of the Photoisomers Considered in the Following Discussion of the Transient Absorption Measurements

**Figure 6:**
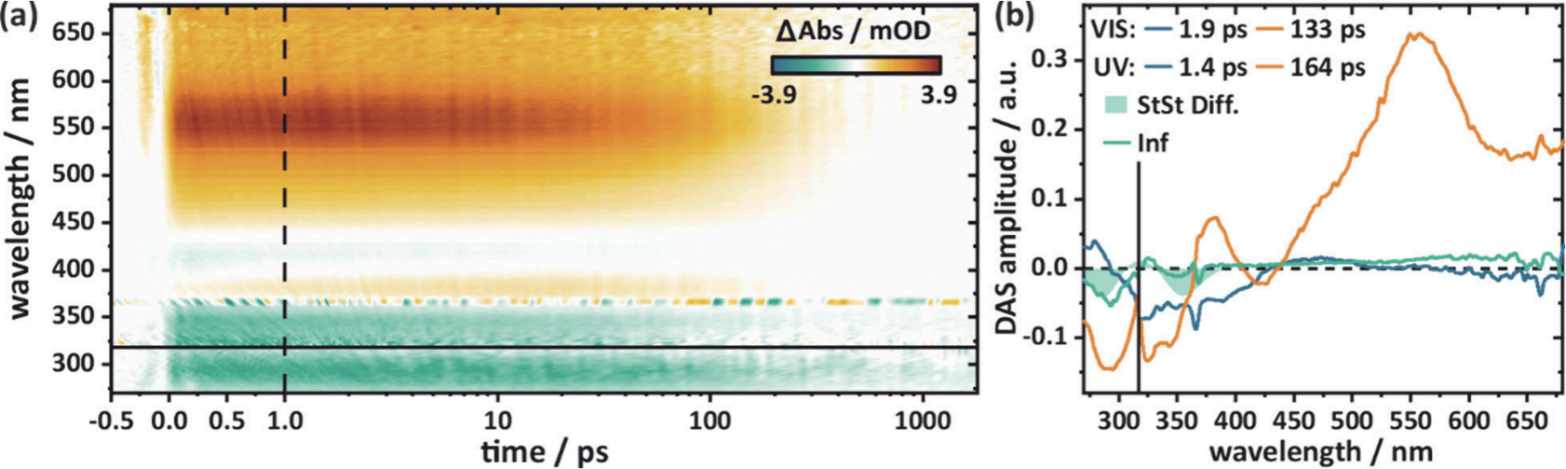
(a) Transient (light-minus-dark) absorption difference spectra of ^***BN***^ **B3**_**NMe2**_ excited with 370 nm. Scattering is visible at this wavelength. (b) Decay associated spectra (DAS) describing the kinetics of (a) with the scaled steady state difference (StSt Diff.) of ^***BN***^ **B3**_**NMe2**_ with ^***BN***^ **D3**_**NMe2**_ (green shaded area) as a reference. Positive DAS amplitudes account for decay of positive or rise of negative absorption whereas negative DAS amplitudes indicate rise of positive or decay of negative signals. UV and vis parts were measured and fitted individually (indicated by the black line) and scaled to match the infinite DAS amplitude (see SI, section 1 for a detailed explanation).

According to the observed steady state switching behavior a more red-shifted illumination would be preferable. Unfortunately, the data quality of the excitation with 386 nm is compromised due to low optical density at this wavelength. Nonetheless, the extracted dynamics agree excellently with those obtained for 370 nm excitation (see SI, Figure S91). Therefore, we discuss the 370 nm data set in the following.

Immediately after excitation at 370 nm, a broad positive signal is visible indicating excited state absorption (ESA) ranging from 450 to over 650 nm accompanied by a weaker ESA band at 375 nm (Figure 6a). Negative signals at the ^***BN***^ **B3**_**NMe2**_ ground state absorption wavelengths below 370 nm belong to ground state bleaching (GSB) whereas the other weak negative band detected at 425 nm hints at stimulated emission (SE) from the excited state, which is in good agreement with the steady state fluorescence spectra (Figure 4). All signals vanish around 200 ps except for the GSB, which remains visible throughout the whole measurement window of 1.8 ns. Global lifetime analysis^41^ reveals the kinetics of the observed photoreaction (Figure 6b). A first lifetime of 1.9 ps (1.4 ps in the UV measurement) depicts a slight bathochromic shift of the broad ESA signal. The second ESA band around 375 nm undergoes the same trend and rises from the GSB. There is probably a third ESA signal hidden underneath the negative GSB, which is indicated by positive and negative DAS amplitudes at 300 and 330 nm, respectively. This red shift of excited state absorption could stem from departure from the Franck–Condon region toward the S_1_ minimum. Its unusually long lifetime of 1.9 ps and the consistency in the excited state spectrum during this time implies a flat S_1_ surface. On the other hand, a polar excited state is expected following the steady state fluorescence, which displays a significant bathochromic shift in polar solvents. This is consistent with the shift in SE and the appearance of another ESA band at 375 nm. The excited state then persists for 133 ps (164 ps in the UV measurement) depicted by the pronounced second lifetime, whose DAS shows decay of all excited state bands (ESA, SE) back to the ground state. Remains of ground state bleaching at 300 and 350 nm are present in a third, infinite lifetime. A comparison of this DAS with the scaled steady state absorption measurements, in which the ^***BN***^ **B3**_**NMe2**_ spectrum was subtracted from the ^***BN***^ **D3**_**NMe2**_ spectrum (Figure 6b, green shaded area) shows a convincing similarity. In conclusion, the experiments support a clean photoconversion from ^***BN***^ **B3**_**NMe2**_ to ^***BN***^ **D3**_**NMe2**_. It is unclear, however, when and from which excited state ^***BN***^ **D3**_**NMe2**_ is formed since its presence is only detected by remaining ground state bleach. Prefulvene-like intermediates that could in principle form after internal conversion at the S_1_/S_0_ conical intersection according to computational studies of the parent system,^34,35^ were not observed in the investigated spectral range. Note that DFT computations for 1-SiH_3_–2-Cl-**^*BN*^B** and the 1-TBS-2-mesityl-**^*BN*^B** motif find no prefulvene-like intermediate on the ground state surface,^19,34^ which contrasts the shape of the PES of the unsubstituted case.^31,34^

### Transient Absorption Spectroscopy of ^***BN***^ **D3**_**NMe2**_

^***BN***^ **D3**_**NMe2**_ was prepared nearly quantitatively by illuminating ^***BN***^ **B3**_**NMe2**_ with a 385 nm LED to the photostationary state. 332 nm was selected as the excitation wavelength for the transient absorption experiment to ensure maximum Dewar absorption compared to the other isomers (see Figure 5c). The map in Figure 7a exhibits a broad positive excited state signal above 350 nm starting immediately after excitation and stretching over the whole investigated time scale in varying intensity. GSB is visible below 350 nm and remains throughout the entire measurement window as well. Another positive band indicative of photoproduct absorption arises after 100 ps at 275 nm. Three lifetimes are needed in the global lifetime analysis to describe the data. A fast lifetime of 0.9 ps (0.8 ps in the UV measurement) can be attributed to excited state decay of presumably a local excited state. Negative contributions in this DAS below 350 nm show relaxation back to the initial ground state. Interestingly, another small negative DAS feature hints at a rise of a positive signal at 375 nm where ^***BN***^ **B3**_**NMe2**_ absorbs. After this first lifetime, a second excited state with a different spectrum decays back to the ground state in 51 ps (80 ps in the UV measurement). At this time another positive signal reminiscent of ^***BN***^ **V3**_**NMe2**_ absorption arises, indicated by the negative DAS contribution at 280 nm. We propose that this secondary excited state absorption occurs due to further movement along an excited state surface with electronic rearrangements and even possible intermediates as proposed by Guerra et al.^36^ Other explanations for this spectral evolution cannot be excluded, however, the solvent dependence of the steady state fluorescence hints at polar excited state intermediates. The infinity lifetime displays positive and negative features in the UV region reminiscent of the scaled steady state absorption. In fact, subtracting 40% ^***BN***^ **B3**_**NMe2**_ and 40% ^***BN***^ **V3**_**NMe2**_ spectra from the ^***BN***^ **D3**_**NMe2**_ absorption closely resembles the infinity lifetime (Figure 7b, green shaded area). Additional positive contributions could be attributed to a triplet or long-lived remaining S_1_ state. Time-correlated single photon counting (TCSPC) measurements (see SI, Figure S93a) reveal a relatively short lifetime of 2.2 ns. In contrast, triplet states usually persist on time scales of hundreds of ns to μs.^42^ Further evidence for a long-lived excited state is provided by the similarity of the band structure observed after 50 ps and at the infinite lifetime. In combination with the earlier lifetimes, it seems like ^***BN***^ **D3**_**NMe2**_ switches in both directions. The local excited state is responsible for the formation of ^***BN***^ **B3**_**NMe2**_ on a subps time scale, while later appearing intermediates yield ^***BN***^ **V3**_**NMe2**_. Further evidence of the bidirectional switching can be found in the absorption spectrum of the Dewar isomer before and after the ultrafast measurement, displaying both the absorption features of ^***BN***^ **B3**_**NMe2**_ above 350 nm and absorption maximum of the ^***BN***^ **V3**_**NMe2**_ at 270 nm (see SI, Figure S92).

**Figure 7:**
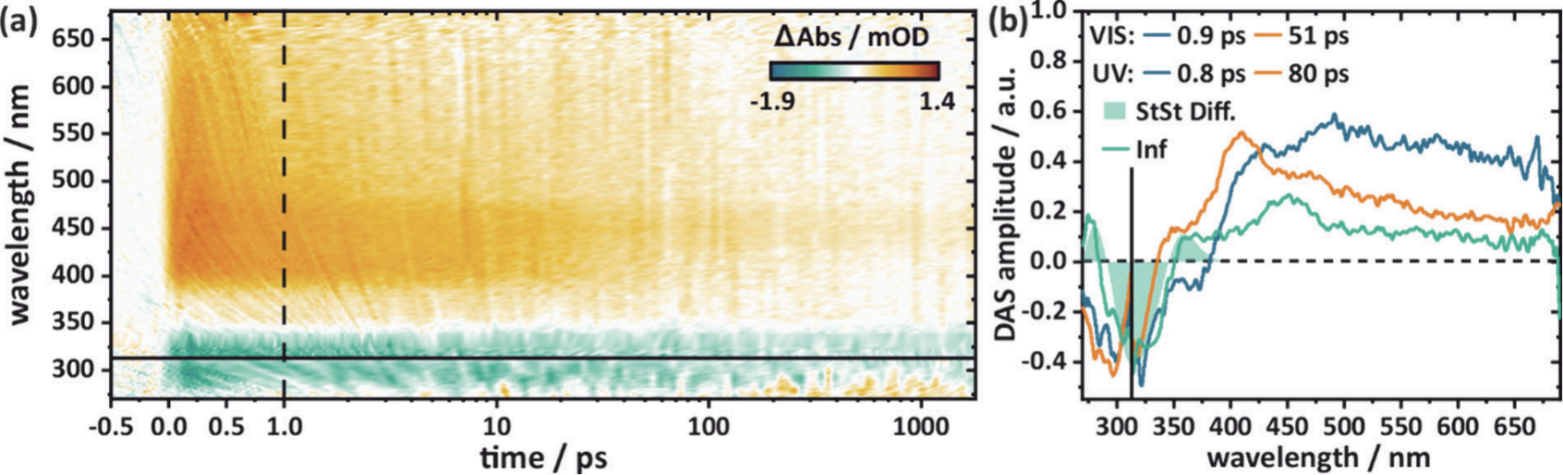
(a) Transient (light-minus-dark) absorption difference spectra of ^***BN***^ **D3**_**NMe2**_ (PSS 385 nm from ^***BN***^ **B3**_**NMe2**_) excited with 332 nm. (b) Decay associated spectra (DAS) describing the kinetics of (a) with the scaled steady state difference (StSt Diff.) of ^***BN***^ **D3**_**NMe2**_ with ^***BN***^ **B3**_**NMe2**_ and ^***BN***^ **V3**_**NMe2**_ (green shaded area) as a reference. UV and vis parts were measured and fitted individually (indicated by the black line) and scaled to match the infinite DAS amplitude (see SI, section 1 for a detailed explanation).

### Transient Absorption Spectroscopy of ^***BN***^ **V3**_**NMe2**_

To isolate the dynamics of the benzvalene isomer, ^***BN***^ **B3**_**NMe2**_ was illuminated with 365 nm light to reach a ^***BN***^ **V3**_**NMe2**_-rich photostationary state. Excitation with a 267 nm laser pulse leads to a broad ESA above 325 nm, similar to the other isomers (Figure 8a). GSB is present below 300 nm. Both signals extend over the measured time frame of 1.8 ns, indicative of either a long-lived excited state or slow photoproduct formation, or both. Global lifetime analysis reveals more complex dynamics with a fast excited state decay back to the benzvalene ground state of 0.8 ps (0.6 ps in the UV measurement). Interestingly, dips in the 0.8 ps DAS at 400 and 550 nm suggest the buildup of a second excited state (Figure 8b). We postulate this state to be of similar nature as the polar excited state intermediates found for ^***BN***^ **D3**_**NMe2**_. Its decay around 117 ps (100 ps in the UV measurement) barely changes the ground state bleaching, but produces a new positive signal at 325 nm. Considering the infinite residuals of this feature as well as the scaled steady state absorption differences of ^***BN***^ **V3**_**NMe2**_ and ^***BN***^ **D3**_**NMe2**_ (Figure 8b, green shaded area), a back switching from the ^***BN***^ **V3**_**NMe2**_ to ^***BN***^ **D3**_**NMe2**_ is highly plausible. Other positive bands above 350 nm in the infinity DAS decay with the TCSPC measured lifetime of 3.7 ns (see SI, Figure S93b). Since these residuals resemble the earlier DAS at 117 ps, and 3.7 ns would constitute an unusually fast triplet decay, we again propose that remaining long-lived excited state population is responsible for these residuals.

**Figure 8:**
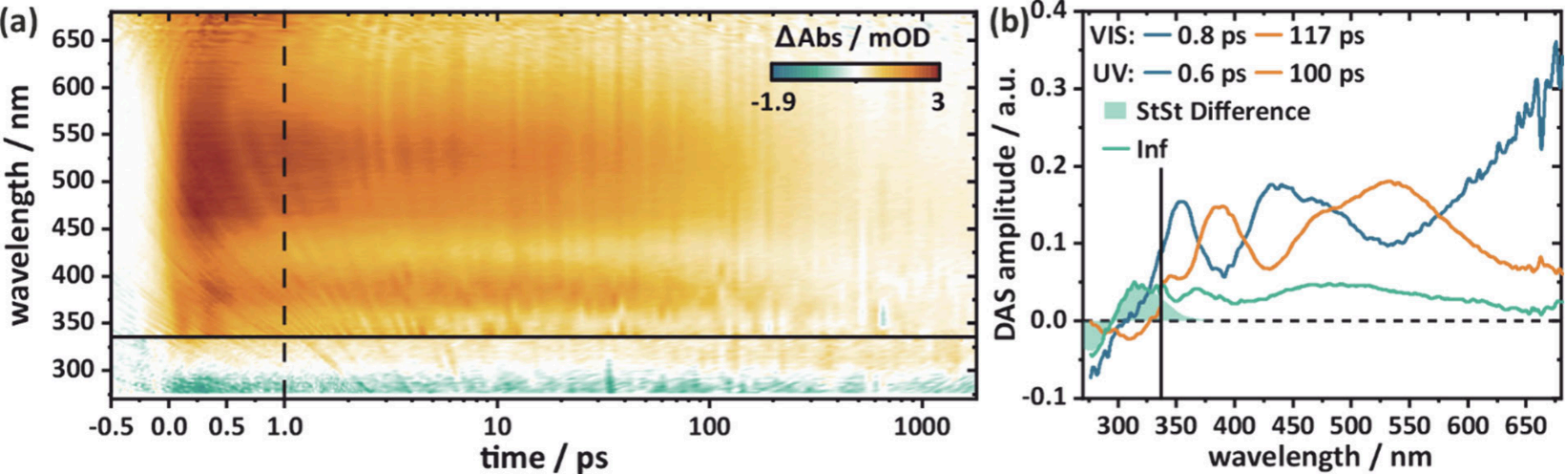
(a) Transient absorption (light-minus-dark) difference spectra of ^***BN***^ **V3**_**NMe2**_ (photo stationary state 365 nm from ^***BN***^ **B3**_**NMe2**_) excited with 267 nm. (b) Decay associated spectra (DAS) describing the kinetics of (a) with the scaled steady state difference (StSt Diff.) of ^***BN***^ **V3**_**NMe2**_ with ^***BN***^ **D3**_**NMe2**_ (green shaded area) as a reference. UV and vis parts were measured and fitted individually (indicated by the black line) and scaled to match the infinite DAS amplitude (see SI, section 1 for a detailed explanation).

### Mechanistic Considerations

Both the steady-state and transient absorption spectra consistently indicate that photochemical excitation of ^***BN***^ **B3**_**NMe2**_ initially leads to the formation of ^***BN***^ **D3**_**NMe2**_. This process can be selectively addressed by excitation near the long-wavelength absorption onset. In doing so transient absorption measurements assign a short lifetime of 1.9 ps to the singlet excited state, in agreement with quantum-chemical calculations.^35,36^ Considering the second lifetime of 133 ps and the steady-state fluorescence, the involvement of a polar excited state surface appears likely. It is however unclear, whether the first (1.9 ps) or second (133 ps) decay yields ^***BN***^ **D3**_**NMe2**_ (Scheme 5).

**Scheme 5:**
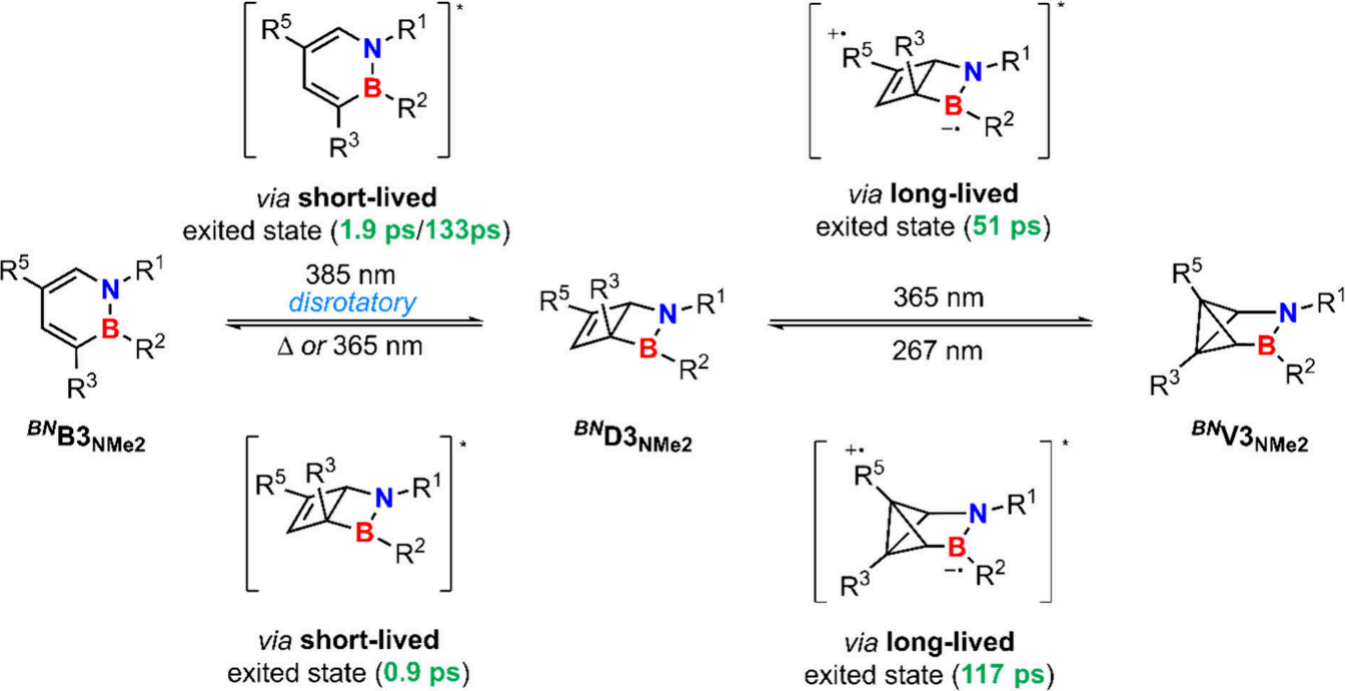
Proposed Mechanism for the Photochemical Formation of ^***BN***^ **V3**_**NMe2**_ in a Follow-up Reaction from ^***BN***^ **D3**_**NMe2**_ Based on the Transient Absorption Measurements^a^

a The lifetimes for the ^***BN***^ **B3**_**NMe2**_ system correspond to the vis measurements.

Irradiation of ^***BN***^ **D3**_**NMe2**_ results in the rapid formation (0.9 ps) of ^***BN***^ **B3**_**NMe2**_, while an additional excited species with a longer lifetime (51 ps) gives rise to the formation of ^***BN***^ **V3**_**NMe2**_. TCSPC measurements together with fluorescence spectra indicate that the latter, as well as further positive contributions, can be attributed to long-lived excited states, probably including several intermediates. Calculated detachment and attachment densities (Figure S90) suggest that the charge shifts in the excited state. Electron density is withdrawn from the NMe_2_ group of the C5 substituent, while it is added to the boron center (see Scheme 5). Very similar to this process, excitation of ^***BN***^ **V3**_**NMe2**_ also exhibits an initial short-lived state (0.8 ps). This state can relax either to the ground state or into a comparable long-lived state (117 ps). Once again, calculations support a similar charge accumulation on the boron atom, this time via the C3 or C5 aryl substituent (SI Figure S90). This electron density shift, in turn, enables a back-reaction to ^***BN***^ **D3**_**NMe2**_.

### Thermal Ring Opening of ^***BN***^ **V3**

Upon harsh heating (>110 °C) of ^***BN***^ **V3**_**H**_, a slow back-conversion was detected by NMR spectroscopy. Mesitylene was chosen as the solvent due to its high boiling point. Each 5 min a ^1^H NMR spectrum was measured over the course of at least 4 h, and this experiment was repeated at four different temperatures. However, attempts to fit the measured data using zero-, first-, or second-order kinetic models failed to produce a linear correlation in an Arrhenius or Eyring plot (see SI, Figure S76). Additionally, a gradual decrease in the overall NMR signal intensity was observed at elevated temperatures, accompanied by a yellow to orange coloration of the solution. Both findings provide evidence for thermal decomposition of one or more components at these high temperatures.

The main product detectable by NMR spectroscopy recorded after heating of ^***BN***^ **V3**_**H**_ matches the original spectrum of ^***BN***^ **B3**_**H**_ prior to irradiation. The positions of the two phenyl substituents were unambiguously assigned by 2D NMR spectroscopy to C3 and C5. As the C3- and C5-substituent are identical, an exchange is symmetry-equivalent and experimentally indistinguishable from retention of the original positions. Besides, additional low-intensity signals were observed that may correspond to the related isomer ^***BN***^ **B4**_**H**_ (Scheme 6). In addition, in the case of heating of ^***BN***^ **V3**_**NMe2**_, small amounts of the ^***BN***^ **B4**_**NMe2**_ derivative were successfully isolated by column chromatography after the thermal cycloreversion and could be fully characterized. Still, ^***BN***^ **B3**_**NMe2**_ is unequivocally identified as the main product of the thermal ring opening reaction.

**Scheme 6:**
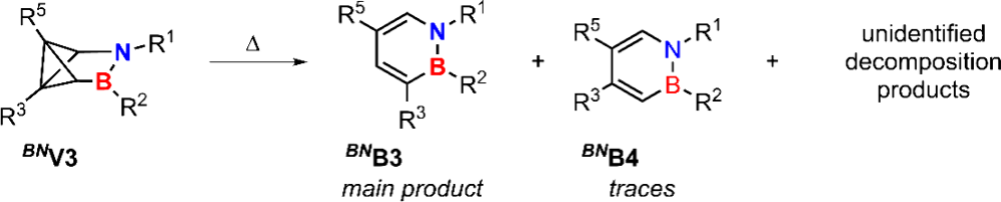
Products of the Thermal Ring-Opening Generated by Heating Temperatures above 110 °C for Several Hours

The evident decomposition, as well as the presence of multiple thermal reaction pathways, provides a plausible explanation for the lack of a consistent correlation in the kinetic analysis. The fundamentally different but parallel processes cannot be analyzed individually due to overlapping NMR signals, which distort the experimental data for the anticipated thermal ring-opening of ^***BN***^ **V3** to ^***BN***^ **D3** or ^***BN***^ **B3**.

Quantum chemical calculations of the ground state were conducted at the previously benchmarked^40^ revPBE0­(D3BJ)/def2-TZVP//PBEh-3c/def2-mSVP level of theory to investigate possible thermal back reaction pathways. The ground state studies were exemplified for the ^***BN***^ **V3**_**H**_ system where the methyl groups in meta position of the boron substituent R^2^ were replaced by hydrogen atoms for computational simplicity, while insignificantly altering the electronic structure. Hence the dihydroazaborinine considered for the computation is abbreviated in the following as ^***BN***^ **B3** without further specification or subscript. Two plausible thermal back reaction pathways were identified yielding either ^***BN***^ **B3** or ^***BN***^ **B4**. The former is formed via a stepwise mechanism while the latter forms in a concerted cycloreversion. The concerted pathway yielding ^***BN***^ **B4** can be interpreted as the perpendicular bond connecting C3 and C5 rotating back into the plane of the remaining four backbone atoms. This reaction coordinate is well represented by the N–B–C3–C5 dihedral angle that is monotonously descending and follows the absolute slope of the energy (see Figure 9). A schematic pathway is shown in Scheme 7 and the transition state **TS**_**con**_ is depicted in the SI (see Figure S88).

**Figure 9:**
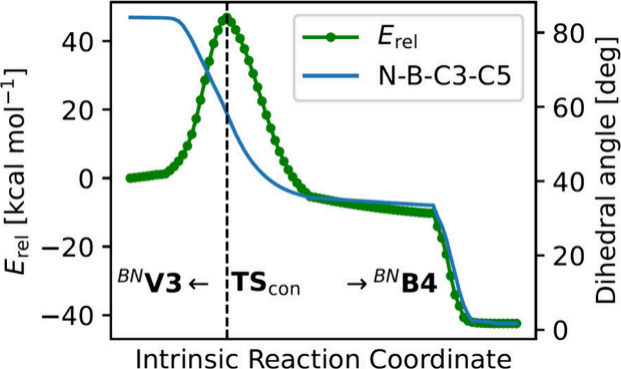
Concerted pathway interconnecting ^***BN***^ **V3** with ^***BN***^ **B4** showing the relative energies as well as the N–B–C3–C5 dihedral angle. Energies are given at the PBEh-3c/def2-mSVP level of theory. The kink toward ^***BN***^ **B4** arises from a subsequent geometry optimization initiated from the final intrinsic reaction coordinate structure.

**Scheme 7:**
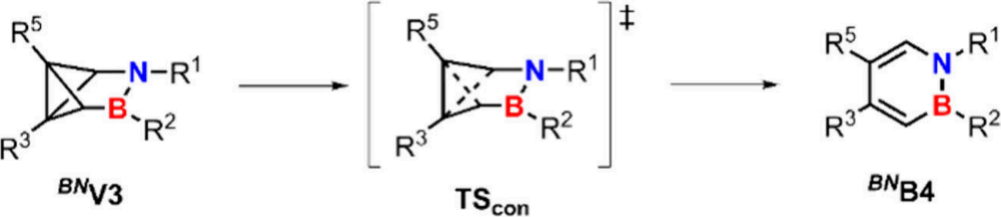
Concerted Pathway from ^***BN***^ **V3** to ^***BN***^ **B4**

The stepwise mechanism toward ^***BN***^ **B3** initially breaks the C3–C6 bond via transition state **TS1** forming intermediate **I** which is stabilized by an additional C3–B bond on one side while the lone pair of the nitrogen can stabilize the other side of the backbone (Scheme 8). Intermediate **I** can geometrically be interpreted as another benzvalene isomer with a hypervalent boron. Subsequently, the C3–C5 as well as the C4–B bond break which eventually reforms ^***BN***^ **B3** via transition state **TS2** (Scheme 8). Examining the energy profile of the two pathways (see Figure 10) shows that the concerted pathway has a barrier of 37.6 kcal/mol. For the stepwise mechanism, the rate-determining step is the first step overcoming **TS1** with a barrier of 26.8 kcal/mol yielding intermediate **I** which is energetically less favorable than ^***BN***^ **V3**. In a second step, ^***BN***^ **B3** is reformed via **TS2** with a lower barrier of 11.9 kcal/mol. Since the second barrier is significantly lower than the first, the reaction should rapidly progress toward ^***BN***^ **B3** making the observation and isolation of **I** unfeasible in the experiment. Further, this agrees with experimental results showing that the concerted pathway, being energetically unfavorable, produces only traces of ^***BN***^ **B4**. Assuming an identical prefactor for the two pathways in the Arrhenius equation and a temperature of 110 °C, the stepwise pathway is 10^6^ faster compared to the concerted pathway (see Figure S89).

**Scheme 8:**
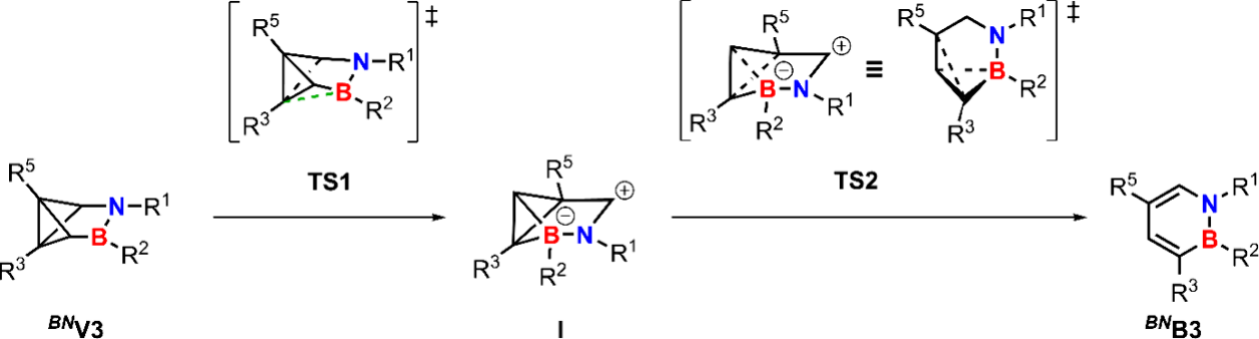
Stepwise Mechanism from ^***BN***^ **V3** to ^***BN***^ **B3** Via Intermediate I^a^

a The newly formed B–C3 bond via TS1 is indicated by a green dashed line while bonds about to break are drawn as black dashed lines. Only σ bonds are considered for simplicity.

**Figure 10:**
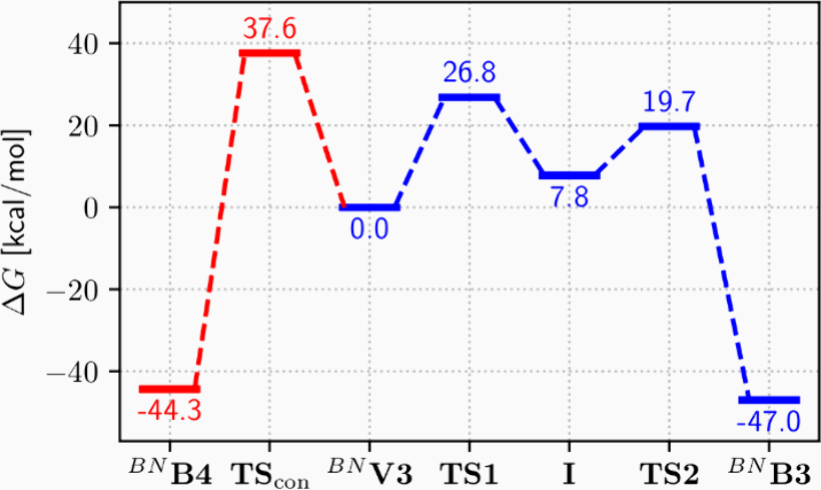
Relative Gibbs enthalpies (revPBE0­(D3BJ)/def2-TZVP//PBEh-3c/def2-mSVP) for the two possible pathways. The concerted pathway yielding ^***BN***^ **B4** (red) and the stepwise pathway reforming ^***BN***^ **B3** (blue).

As the substituents at C3 and C5 are identical, these positions are symmetry-equivalent in the ^***BN***^ **V3** structures under consideration. Consequently, the products with exchanged substituents are identical and cannot be distinguished either theoretically or experimentally.

## Conclusion

We successfully applied a previously optimized method for the Suzuki cross-coupling of C3-brominated ^***BN***^ **B6** to C3,C5-dibrominated species and synthesized the C3,C5-diarylated dihydroazaborinins ^***BN***^ **B3** in good yields. Due to the two aryl substituents, the absorption wavelengths of these compounds are bathochromically shifted into the UV-A region. Particularly strongly electron-donating groups produce a red shift that extends slightly into the visible spectrum for ^***BN***^ **B3**_**NMe2**_. Under broadband irradiation (280–400 nm) these compounds undergo an isomerization to the corresponding benzvalene isomer (^***BN***^ **V3**), with the Dewar isomer (^***BN***^ **D3**) as an intermediate. During this isomerization, the C3 and C4 positions interchange along with their attached substituents. A detailed study of this photoisomerization using transient absorption spectroscopy further provides a convincing picture of a two-step switching process. Specifically, ^***BN***^ **B3**_**NMe2**_ isomerizes cleanly to ^***BN***^ **D3**_**NMe2**_ after excitation into its red absorption edge. A second photon targeting ^***BN***^ **D3**_**NMe2**_ enables both back-switching to ^***BN***^ **B3**_**NMe2**_ and isomerization to ^***BN***^ **V3**_**NMe2**_. In contrast, ^***BN***^ **V3**_**NMe2**_ displays unprecedented photochemistry, undergoing isomerization to ^***BN***^ **D3**_**NMe2**_ upon UV–C irradiation. This photochemical switching between ^***BN***^ **D3**_**NMe2**_ and ^***BN***^ **V3**_**NMe2**_ involves long-lived excited states, probably with polar intermediates, which constitute a plausible pathway to facilitate the C3–C4 boron shift. Given the thermal stability of ^**BN**^ **V3**, precise photochemical control of this back reaction is feasible. In addition, two thermal back reaction pathways were identified for ^***BN***^ **V3**. A stepwise and energetically favorable pathway yields the starting material ^***BN***^ **B3** as the main product, while the second pathway is a concerted one yielding the side product ^***BN***^ **B4**. Taken together, we demonstrate a complete three-state photoswitch with individually addressable isomers. To the best of our knowledge, this represents the first report of back-switching from ^***BN***^ **D** without auxiliaries or catalysts, as well as the first ^***BN***^ **V** derivative with photo switching properties.

## Supplementary Material



## Data Availability

The data underlying this study are available in the published article and its Supporting Information.

## Notes

Experimental details, NMR, MS, UV–vis spectral data, computational details, Cartesian coordinates, crystal structure data (PDF)

## Abbreviations

cy: cyclohexane
THF: tetrahydrofuran
UV: ultraviolet
Vis: visible
S_1_: first singlet excited state
LE: local excited
CT: charge-transfer
FC: Franck–Condon
LED: Light emitting diode
TCSPC: time correlated single photon counting
DAS: decay associated spectra
ESA: excited state absorption
GSB: ground state bleach
SE: stimulated emission
