# Supplementary material for: Switching Shapes: Reversible Three Species Photoisomerization of Substituted 1,2-Dihydro-1,2-azaborinines
Source: J Am Chem Soc. 2026 Feb 9;148(10):10776–87. doi: 10.1021/jacs.5c20667 (PMC13003484; doi:10.1021/jacs.5c20667)
Supplement: Supplementary file 1 [file ja5c20667_si_001.pdf]

Supporting Information

Switching Shapes: Reversible Three Species Photoisomerization of Substituted 1,2-Dihydro-1,2-azaborinines

Sonja M. Biebl,[a†] Jonas N. Lienert,[b†] Adrian Müller,[d] Markus Ströbele,[c] Andreas Dreuw,[d*] Josef Wachtveitl,[b*] Holger F. Bettinger[a*]

[a] Institut für Organische Chemie, Eberhard Karls Universität Tübingen, Auf der Morgenstelle 18, 72076 Tübingen, E-Mail: holger.bettinger@uni-tuebingen.de

[b]Institut für Physikalische und Theoretische Chemie, Goethe-Universität Frankfurt, Max von Laue-Straße 7, 60438 Frankfurt am Main, E-Mail: wveitl@theochem.uni-frankfurt.de

[c] Institut für Anorganische Chemie, Eberhard Karls Universität Tübingen, Auf der Morgenstelle 18, 72076 Tübingen

[d] Interdisciplinary Center For Scientific Computing, Heidelberg University, Im Neuenheimer Feld 205, Heidelberg, E-Mail: dreuw@uni-heidelberg.de

[†]These authors contributed equally to this work and should be considered joint first author.

Table of Contents

1. Methods 4

2. Analytics 5

3. Synthesis 8

3,5-Dibromo-1-(*tert*-butyldimethylsilyl)-2-pentamethylphenyl-1,2-dihydro-1,2-azaborine . 8

Catalytic system 13

Substrate scope (boronic acid) 13

100 µmol experiments (*BN*B3) 14

NMR data of **BNB3H** 15

NMR data of **BNB3Me** 19

NMR data of **BNB3OMe** 23

NMR data of **BNB3SMe** 27

NMR data of **BNB3NMe2** 31

NMR data of **BNB3CF3** 35

NMR data of **BNB3Br** 40

4. Irradiation experiments 44

*BN*V3 scope 45

NMR data of **BNV3H** 46

NMR data of **BNV3Me** 51

NMR data of **BNV3OMe** 54

NMR data of **BNV3SMe** 57

NMR data of **BNV3NMe2** 60

5. Kinetic experiments 65

Sample preparation 65

Experimental details 65

Determination of the reaction order 65

Time dependence (0. order kinetic) 66

Time dependence (1. order kinetic) ... 66
Time dependence (2. order kinetic) ... 67
Order of the thermal cycloreversion of **^{BN}V3** ... 68
Arrhenius treatment of the measured data ... 70
6. Thermal cycloreversion of ^{BN}V3 ... 73
NMR data of **^{BN}B4$_{NMe2}$** ... 74
7. Crystal structures ... 78
8. Computations ... 85
Optimized geometry of the concerted transition state TS$_{con}$ ... 85
Arrhenius rate constant ratio of the left (concerted) and right (stepwise) pathways ... 86
Optimized structures (PBEh-3c/def2-mSVP) for the energy profile ... 86
9. Ultrafast Spectroscopy ... 105
References ... 106

1. Methods

Synthesis: Unless otherwise noted, all experiments were carried out under inert conditions using Schlenk technique with argon or nitrogen as the protective gas or in a glove box (UNIlab Pro, MBraun). Glassware was dried before use by heating. Commercial triethylamine (water content ~ 0.2%) was refluxed over KOH for two hours and then distilled before use. The remaining chemicals used were employed as received from the manufacturer without further purification. Anhydrous solvents were obtained from Thermo Fisher Scientific Inc., Acros Organics B.V. B.A., Sigma-Aldrich or, in the case of dichloromethane, diethyl ether, n-hexane, tetrahydrofuran and toluene, from an SPS-800 solvent drying system by the manufacturer MBraun.

Spectral deconvolution: Pure Spectra of $^{BN}\mathbf{B}_{\mathbf{NMe2}}$ and $^{BN}\mathbf{V}_{\mathbf{NMe2}}$ are obtained, the former as the starting compound and the latter after intense UV radiation as indicated by NMR experiments. These spectra are then fitted with multiple Gaussian functions. Solvent-subtracted fits consisting of four Gaussians for $^{BN}\mathbf{B}_{\mathbf{NMe2}}$ (or three Gaussians for $^{BN}\mathbf{V}_{\mathbf{NMe2}}$) are then used to deconvolute spectra additionally containing the third isomer, $^{BN}\mathbf{D}_{\mathbf{NMe2}}$. Figure S1c shows a spectrum prepared by 395 and 375 nm LEDs with a NMR determined ratio of $^{BN}\mathbf{B}_{\mathbf{NMe2}}$/$^{BN}\mathbf{D}_{\mathbf{NMe2}}$/$^{BN}\mathbf{V}_{\mathbf{NMe2}}$ 15/32/53. Scaling $^{BN}\mathbf{B}_{\mathbf{NMe2}}$ and $^{BN}\mathbf{V}_{\mathbf{NMe2}}$ fits according to these amplitudes and subtracting them from the original data yields a spectrum with only $^{BN}\mathbf{D}_{\mathbf{NMe2}}$ present. These remains are then fitted by two Gaussians to obtain a pure $^{BN}\mathbf{D}_{\mathbf{NMe2}}$ fit. The deconvolution ratio of these fits (determined by the area) is then $^{BN}\mathbf{B}_{\mathbf{NMe2}}$/$^{BN}\mathbf{D}_{\mathbf{NMe2}}$/$^{BN}\mathbf{V}_{\mathbf{NMe2}}$ 25/30/45, which is in good agreement with the NMR ratios mentioned above. Differences most likely stem from the difficult subtraction of solvent contributions.

Scaling of UV and Vis ultrafast measurements: For all investigated compounds, UV (260 nm to 380 nm) and Vis (330 nm to 680 nm) parts were collected in two separate transient absorption measurements and fitted individually with global lifetime analysis. To display and compare both measurements in a single figure, the intensities of the UV measurements are scaled to match the Vis part. Therefore, the infinity DAS trace is selected since it is least dependent on measurement conditions and noise. The infinity DAS amplitude in the UV measurement is then empirically scaled to match the infinity DAS amplitude in the Vis measurement in the overlapping wavelength range of 330 nm to 380 nm. All other DAS traces as well as the

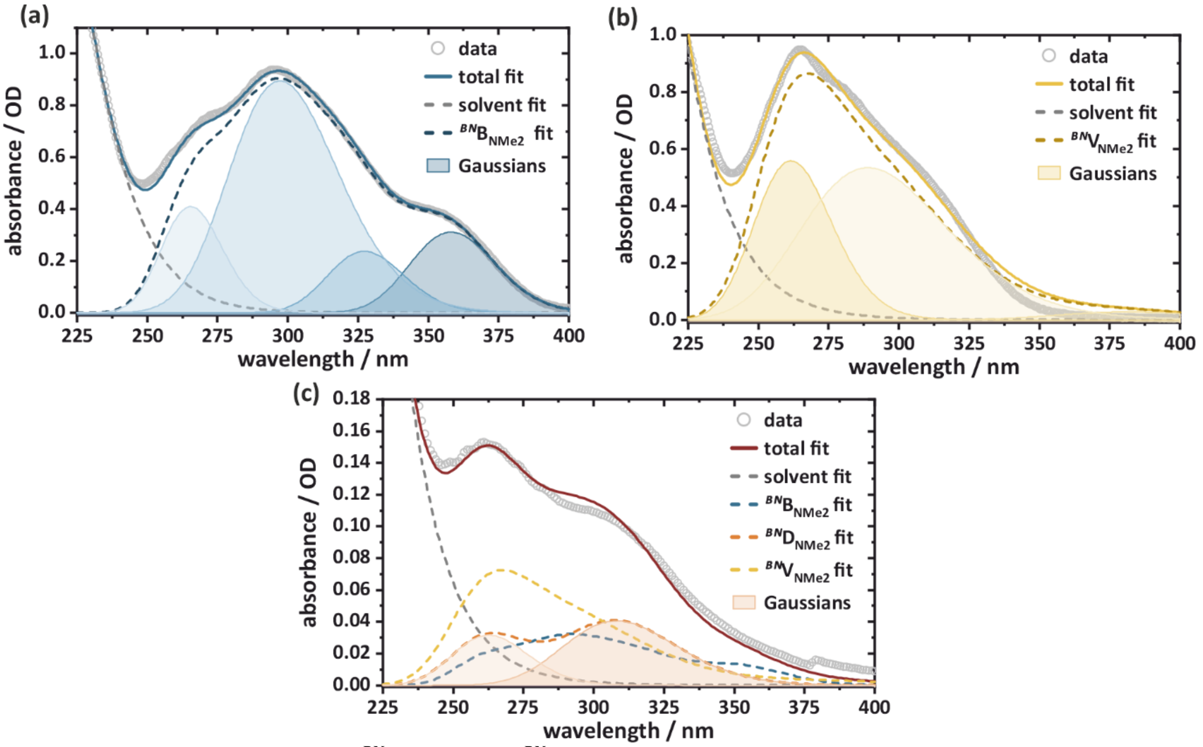

Figure S1: Deconvolution of a pure **^{BN}B$_{NMe2}$** (a) and **^{BN}V$_{NMe2}$** (b) spectrum, fitted by four/three Gaussians for the compound and an additional Gaussian for the solvent. (c) Scaling the **^{BN}B$_{NMe2}$** and **^{BN}V$_{NMe2}$** fits according to NMR data and subtracting them from a 375 nm PSS leaves pure **^{BN}D$_{NMe2}$** to be fitted with two Gaussians.

transient absorption difference map of the UV measurement are then multiplied with this factor. The factors are 0.5, 1.1 and 2 for the **^{BN}B$_{NMe2}$**, **^{BN}D$_{NMe2}$** and **^{BN}V$_{NMe2}$** measurements, respectively. In figures 6-8 of the main text, overlapping wavelengths between 330 nm and 380 nm are shown either from the UV or Vis measurements, chosen by the better data quality. A black line indicates where the cut between UV and Vis data is made. A similar scaling mechanism is used to match the steady state differences to the infinity DAS amplitudes.

2. Analytics

NMR Spectroscopy: The acquisition of ^{1}H, ^{13}C-{^{1}H}, and ^{11}B-{1H} NMR spectra were performed on a Bruker Advance III HD 400 MHz instrument (^{1}H spectra) at 101 MHz (^{13}C-{^{1}H} spectra), and 128 MHz (^{11}B-{1H} spectra). Complementarily, spectra of these nuclei were recorded on a Bruker Advance III HD 300 MHz NanoBay at 300 MHz (^{1}H spectra), 76 MHz (^{13}C-{^{1}H} spectra), and 96 MHz (^{11}B-{1H} spectra). For high-temperature NMR measurements (kinetic experiments) a Bruker AvanceIII HEX 400 spectrometer was employed. The 2D characterizations were carried out using a Bruker Avance III HDX 600 spectrometer with a measuring frequency of 600 MHz (^{1}H spectra), 151 MHz (^{13}C-{^{1}H} spectra) or 192 MHz (^{11}B-{1H} spectra) and on a Bruker Avance III HDX 700 with a measuring frequency of 700 MHz

(^{1}H spectra) or 176 MHz (^{13}C-{^{1}H} spectra). All obtained NMR spectra were referenced to the solvent peak. Deuterated dichloromethane (5.32 ppm), benzene (7.16 ppm), chloroform (7.26 ppm), cyclohexane (1.38 ppm), or mesitylene (2.26 ppm) from Sigma-Aldrich or Deutero were used for referencing.

MPLC Chromatography: Column chromatographic purifications were performed using the puriFlash 430 in combination with pre-packed silica gel columns (particle size: 30 µm) from Interchim. Detection was carried out using a puriFlash One Series UV detector (DAD 200-600 nm) from the same manufacturer.

All solvents used were of HPLC-grade purity. Solvent mixtures are indicated as V/V ratio.

GPC Chromatography: The size exclusion chromatography was conducted on a Recycling Preparative HPLC and GPC (LaboAC LC-7080 Plus II) device and a JAIGEL-2.5 HR Plus column both from Japan Analytical Industrie Co.

UV/VIS Spectroscopy: For determining the absorption maxima of the presented 1,2-substituted 1,2-dihydro-1,2-azaborinines, a Specord S600 from Analytik Jena was used, operated with the accompanying Specord software (Version 2.0.2). The wavelength range covered was from 180 nm to 960 nm. All solvents used had a cut-off wavelength below 250 nm and were water- and oxygen-free.

(NMR-)Irradiation experiments: As light source an Osram HBO-500-W/2 high pressure mercury lamp in an Oriel housing with quartz optics and a dichroic mirror (280-400 nm or 230-260 nm) was applied. Additional filters from *Schott* were used as indicated in the experimental section. Alternatively heatsink mounted, collimated LEDs (M395L5, M385L3, M375L4, M365L3, M340L5, M325L5, M300L4, M280L6) from Thorlabs GmbH were used and focused with a telescope and a plano-cylindrical lens. Photostationary states for UV-Vis and Ultrafast transient absorption analysis were reached using the same LEDs.

The samples for the kinetic studies prepared in quartz glas J.-Young-NMR tubes as the reaction vessel.

X-Ray Crystallography: Crystaly suitable for X-Ray diffraction were grown by evaporation with *n*-hexane and dichloromethane at room temperature. Single crystals were selected, coated with Parabar 10312 and fixed on a microloop.

Data were collected on a XtaLAB Synergy, Dualflex, HyPix diffractometer using *w* scans with Cu K_a radiation. The crystal was kept at a steady temperature during data collection. The

diffraction pattern was indexed and the total number of runs and images was based on the strategy calculation from the program CrysAlisPro 1.171.42.49, which was also used for refining the unit cell. The structure was solved with the **ShelXT** 2018/2 solution program[1,21] using dual methods and by using **Olex2** 1.5-ac5-024 as the graphical interface.[2] The model was refined with **olex2.refine** 1.5-ac5-024 using full matrix least squares minimisation on $\boldsymbol{F}^2$.[3]

Mass spectrometry: High resolution mass spectra were recorded on a HR-ESI/APCI-TOF device (maXis 4G, Bruker) or a HR-ESI-Orbitrap-MS (Q exactive HF, Thermo Scientific) for ESI ionization. The sample was dissolved in dichloromethane and injection was carried out using a syringe pump.

Ultrafast transient absorption spectroscopy: The laser system providing the fundamental beam is based on a Spitfire ACE 120 from Spectra Physics with 800 nm, 6 W, 1 kHz, 100 fs. It is split into pump and probe beams. Excitation of samples with specific wavelengths is achieved by a classic two-stage NOPA setup with prisms for pulse compression and sum-frequency generation for UV pulses. Probe beams are generated through a supercontinuum in a CaF_2 crystal, pumped with the fundamental of 800 nm for Vis and with 400 nm for UV white light. During a measurement, the pump-probe probe scheme is detected in a TecSpec MMS UV-VISII Spectrometer by Tec5 AG.

3. Synthesis

1-(*tert*-Butyldimethylsilyl)-2-mesityl-1,2-dihydro-1,2-azaborine (**1**) was synthesized according to Richter *et al.*[4]

3,5-Dibromo-1-(*tert*-butyldimethylsilyl)-2-pentamethylphenyl-1,2-dihydro-1,2-azaborine

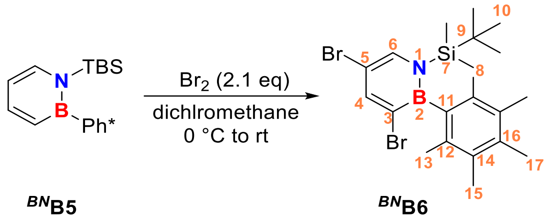

***BN*B5** ***BN*B6**

The synthesis is based on a procedure by Bettinger *et al.*[5] 1-(*tert*-butyldimethylsilyl)-2-pentamethylphenyl-1,2-dihydro-1,2-azaborine (***BN*B5**) (0.8385 g, 2.5 mmol, 1 eq) was solved in dichloromethane (10 mL) and cooled to 0 °C. A solution of bromine (0.265 mL, 5.2 mmol, 2.1 eq) in dichloromethane (10 mL) was added dropwise over 1 h. The reaction mixture was stirred for 30 minutes at 0 °C. The solution was allowed to reach room temperature and stirred for another 30 minutes, before a saturated solution of Na_2S0_3 (20 mL) was added. The aqueous layer was extracted three times with *n*-hexane (15 mL) and the combined organic layers were dried over $MgSO_4$. After removing of the solvent, the crude product was purified by column chromatography (silica, *n*-hexane/dichloromethane gradient). The product was obtained as colorless solid/crystals (1.1549 g, 94%).

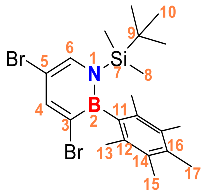

*^{BN}***B6**

$C_{21}H_{32}BBr_2NSi$ (497.20 g/mol)

^{1}H-NMR (700 MHz, **CD_2Cl_2**): δ = 7.98 (d, $^4J_{HH}$ = Hz, 1H, H-4), 7.59 (d, $^4J_{HH}$ = Hz, 1H, H-6), 2.26 (s, 3H, H-17), 2.18 (s, 6H, H-15), 2.00 (s, 6H, H-13), 0.91 (s, 9H, H-10), -0.06 (s, 6H, H-8) ppm.

^{13}C-{^{1}H}-NMR (100 MHz, **CD_2Cl_2**): δ = 149.6, 139.5, 138.3, 134.8, 134.6, 132.9, 131.9, 104.2, 27.4, 21.7, 19.4, 16.7, 16.1, -3.1 ppm.

^{11}B-{^{1}H}-NMR (128 MHz, **CD_2Cl_2**): δ = 40.5 ppm.

HR-MS (APCI): m/z calc. For [M+Na]+ 518.06624, found 518.06624.

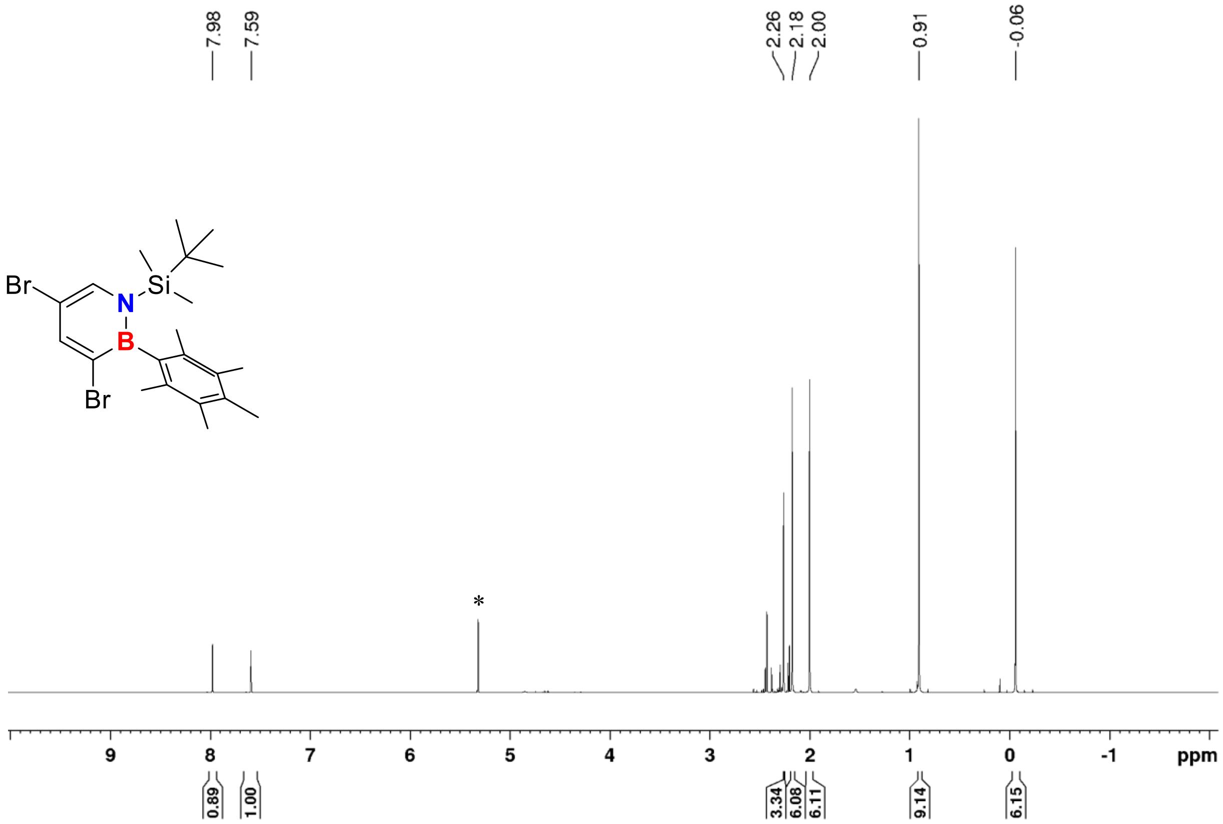

Figure S2. ^{1}H-NMR spectrum of compound ^{BN}**B6** in CD_2Cl_2 measured at a 700 MHz spectrometer. The solvent signal is marked with an asterisk.

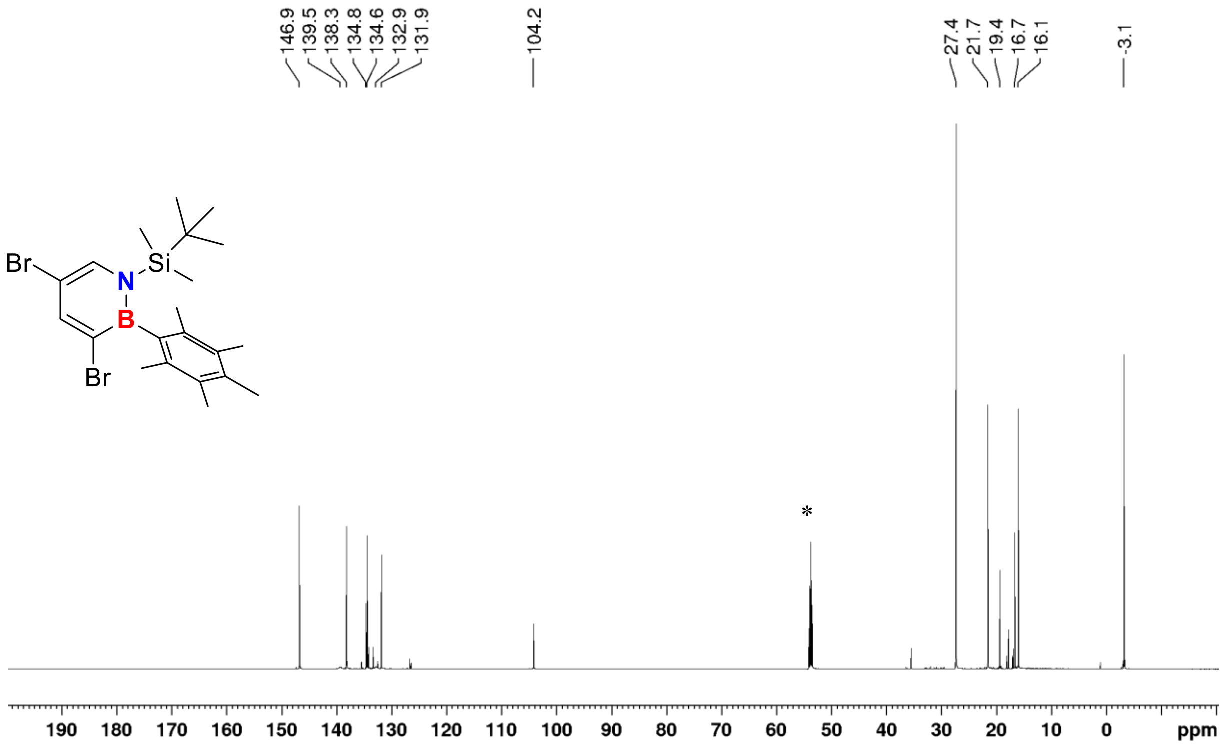

Figure S3. ^{13}C-{^{1}H}-NMR spectrum of compound ^{BN}**B6** in CD_2Cl_2 measured at a 700 MHz spectrometer. The solvent signal is marked with an asterisk.

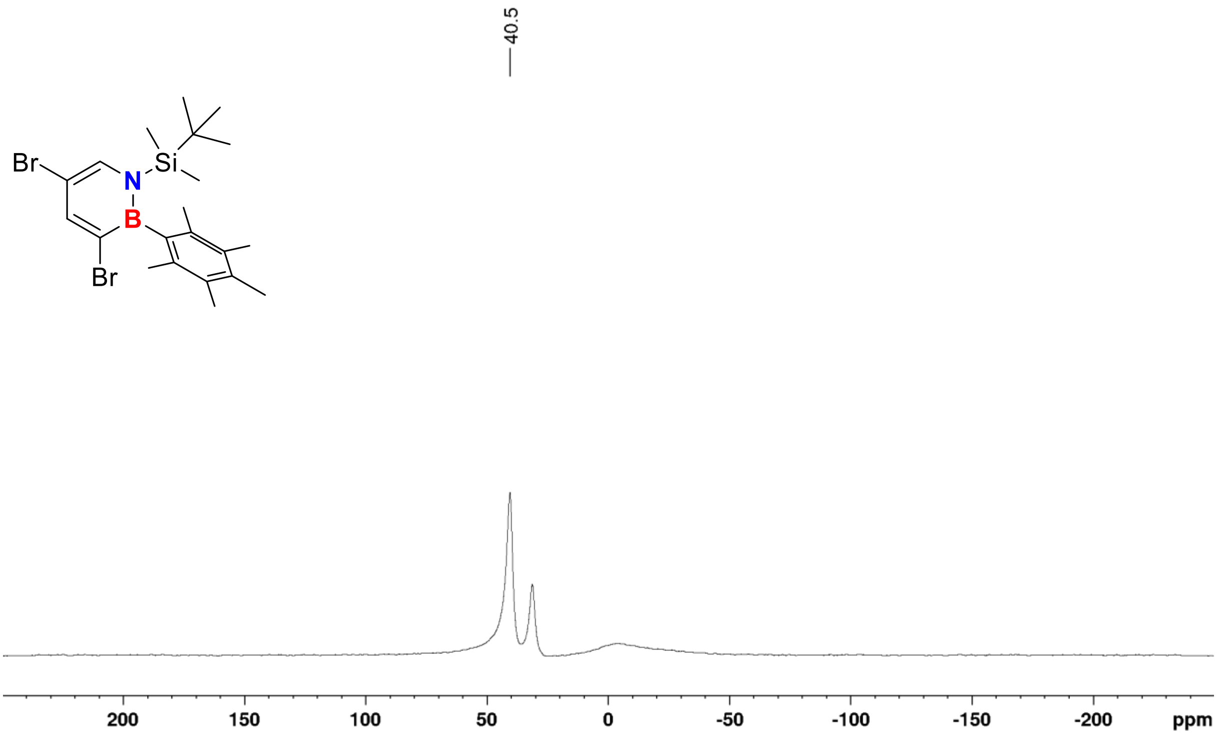

Figure S4. ^{11}B-{^{1}H}-NMR spectrum of compound ^{BN}**B6** in CD_2Cl_2 measured at a 400 MHz spectrometer.

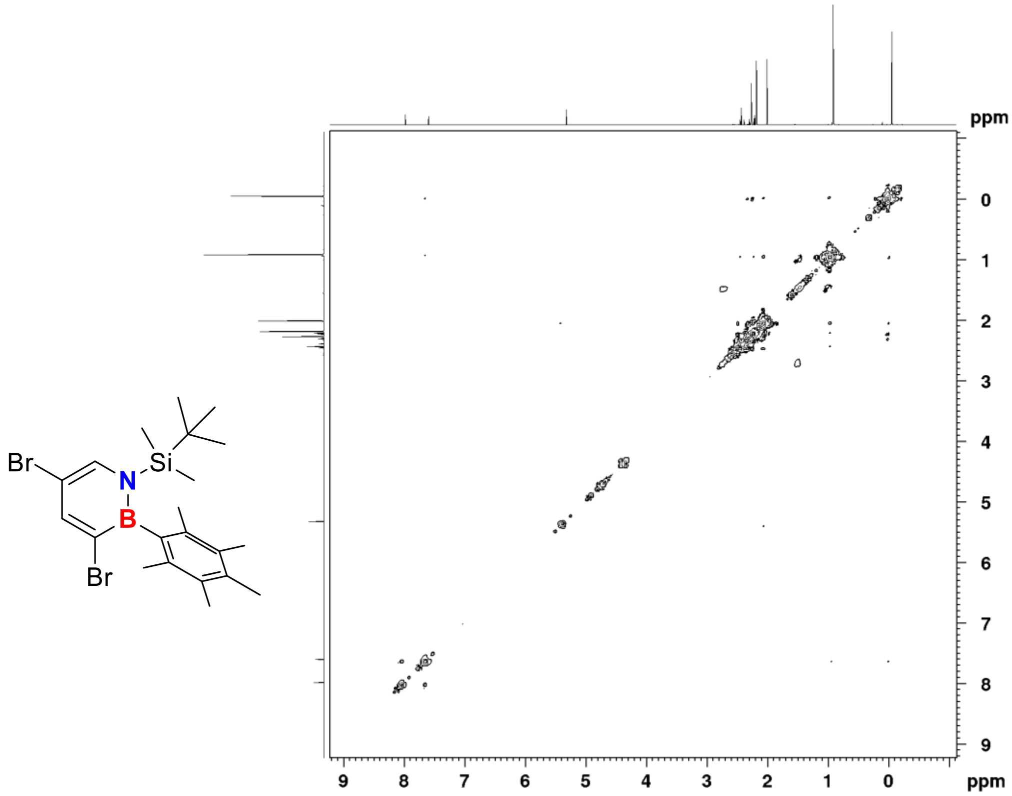

Figure S5. ^{1}H-^{1}H-COSY-NMR spectrum of compound ^{BN}**B6** in CD_2Cl_2 measured at a 700 MHz spectrometer.

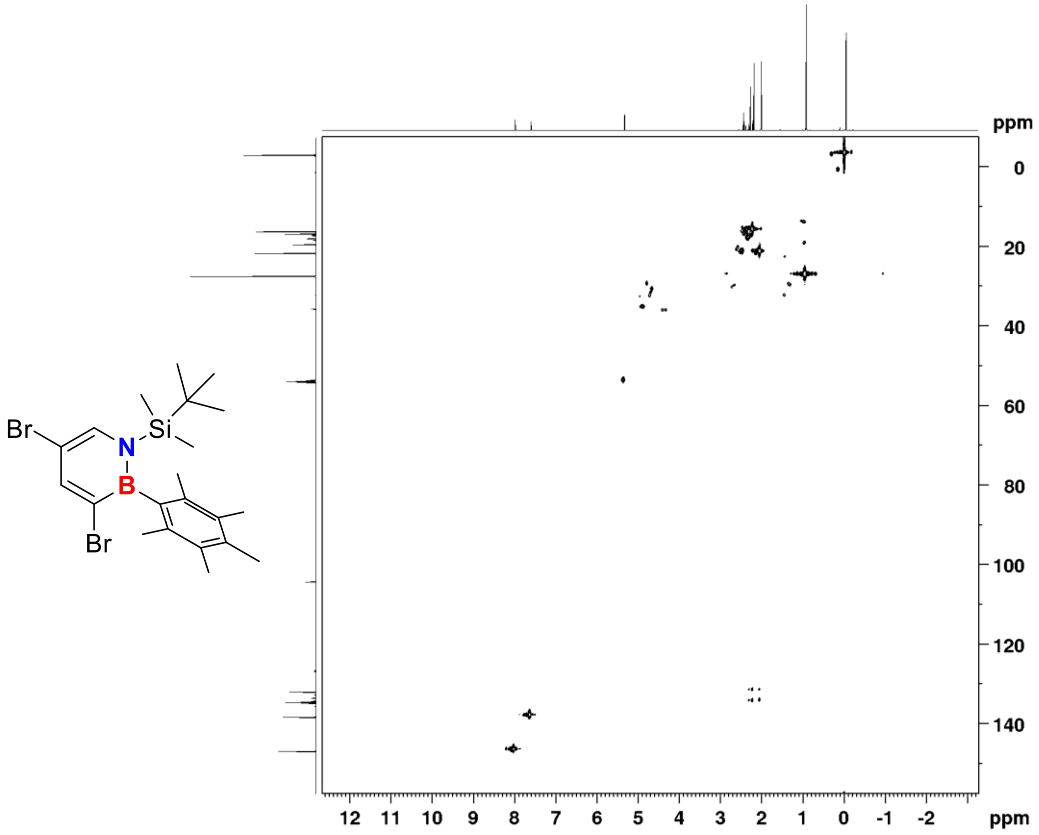

Figure S6. ^{1}H-^{13}C-HSQC-NMR spectrum of compound ^{BN}**B6** in CD_2Cl_2 measured at a 700 MHz spectrometer.

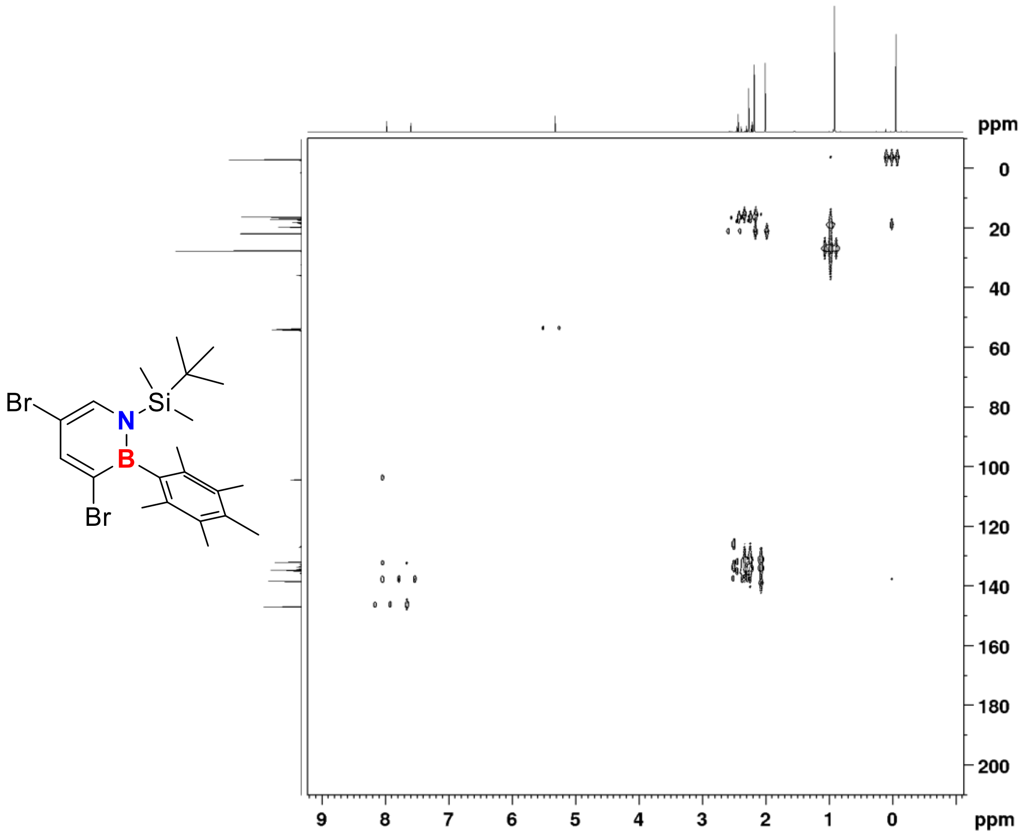

Figure S7. ^{1}H-^{13}C-HMBC-NMR spectrum of compound ^{BN}**B6** in CD_2Cl_2 measured at a 700 MHz spectrometer.

Catalytic system

The composition of the catalytic system is identical to previously optimized reaction conditions form the Suzuki coupling of the C3 position of dihydroazaborinins developed in our group.[5]

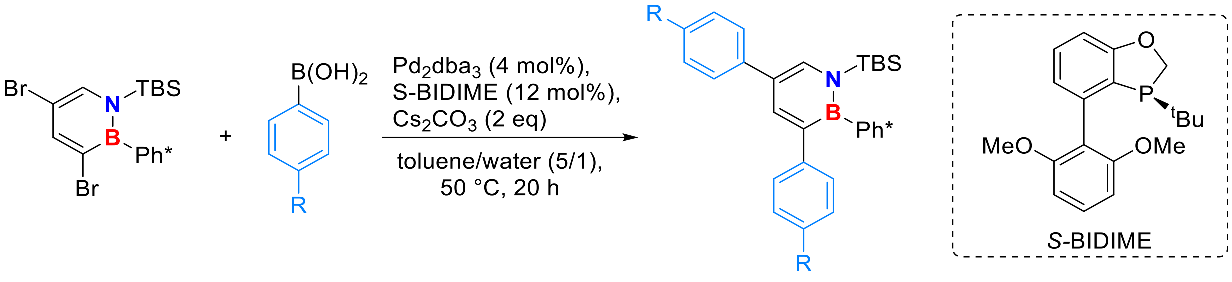

*BN*B6 *BN*B3

Substrate scope (boronic acid)

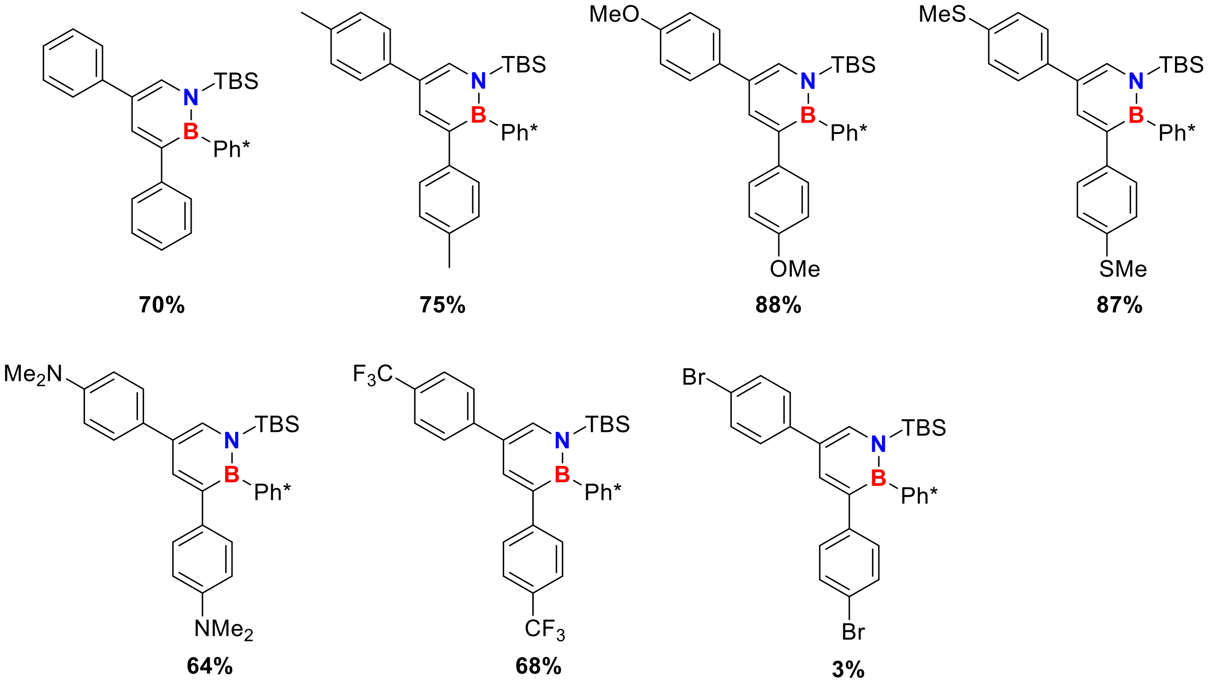

100 µmol experiments (*^{BN}*B3)

3,5-Dibromo-1-(*tert*-butyldimethylsilyl)-2-pentamethylphenyl-1,2-dihydro-1,2-azaborinine (0.05 g, 0.1 mmol, 1 eq), 4-R-phenylboronic acid (0.13 mmol, 1.3 eq), dry Cs_2CO_3 (0.065 g, 2 mmol, 2 eq), $Pd_2(dba)_3$ (3.7 mg, 0.004 mmol, 4 mol%) and S-BIDIME (4 mg, 0.012 mmol, 12 mol%) were solved in dry, degassed toluene (1.5 mL) and degassed water (0.3 mL). The reaction mixture was allowed to stir for 16 h at 50 °C. Distilled water (2 mL) was added and the aqueous layer was extracted three times with *n*-hexane (2 mL). The combined organic layers were dried over $MgSO_4$. After removing of the solvent, the crude product was purified by column chromatography (silica, n-hexane/dichloromethane gradient). The product was obtained as colorless solid in the case of ^{BN}**B3**$_{\mathbf{H}}$, ^{BN}**B3**$_{\mathbf{Me}}$, ^{BN}**B3**$_{\mathbf{OMe}}$, ^{BN}**B3**$_{\mathbf{SMe}}$, ^{BN}**B3**$_{\mathbf{CF3}}$ and ^{BN}**B3**$_{\mathbf{Br}}$. Compound ^{BN}**B3**$_{\mathbf{NMe2}}$ was a pale-yellow solid.

*NMR data of ^{BN}**B3$_H$***

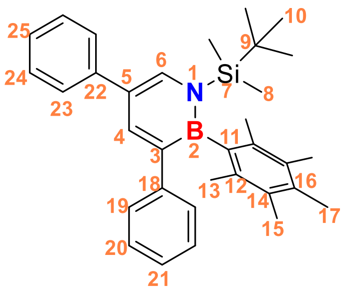

^{BN}B3$_H$

$C_{33}H_{42}BNSi$ (491.60 g/mol)

^{1}H-NMR (400 MHz, **CD_2Cl_2**): δ = 7.77 (d, $^4J_{HH}$ = 1.39 Hz, 1H, H-4), 7.67 (d, $^4J_{HH}$ = 1.39 Hz, 1H, H-6), 7.47 (m, 2H, H-19), 7.28 (m, 2H, H-24), 7.15 (m, 1H, H-23), 6.90 (m, 5H, H-19/H-20), 2.18 (s, 3H, H-17), 2.06 (s, 6H, H-15), 1.94 (s, 6H, H-13), 0.95 (s, 9H, H-10), -0.01 (s, 6H, H-8) ppm.

^{13}C-{^{1}H}-NMR (100 MHz, **CD_2Cl_2**): δ = 146.3 (C4), 142.4, 141.7 (C6), 141.6, 136.3, 135.0, 133.9, 131.7, 129.1, 128.8, 127.6, 127.0, 126.6, 125.9, 125.5, 32.5, 28.1, 27.0, 26.8, 23.5, 22.2, 19.9, 16.7, 16.1, 14.1, -2.6 ppm.

^{11}B-{^{1}H}-NMR (128 MHz, **CD_2Cl_2**): δ = 39.9 ppm.

HR-MS (ESI): m/z calc. For [M+Na]+ 514.30779, found 514.30816.

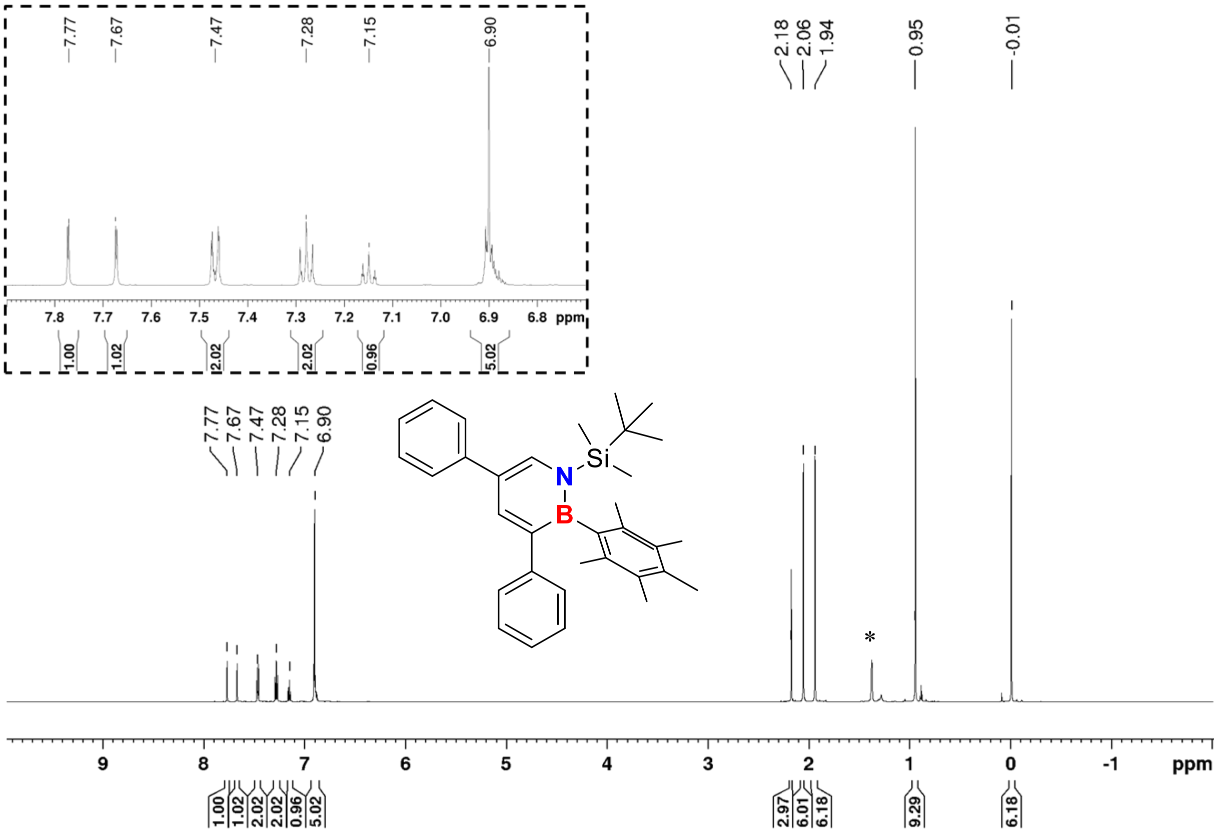

Figure S8. ^{1}H-NMR spectrum of compound ^{BN}**B3$_{H}$** in C_6D_{12} measured at a 600 MHz spectrometer. The solvent signal is marked with an asterisk.

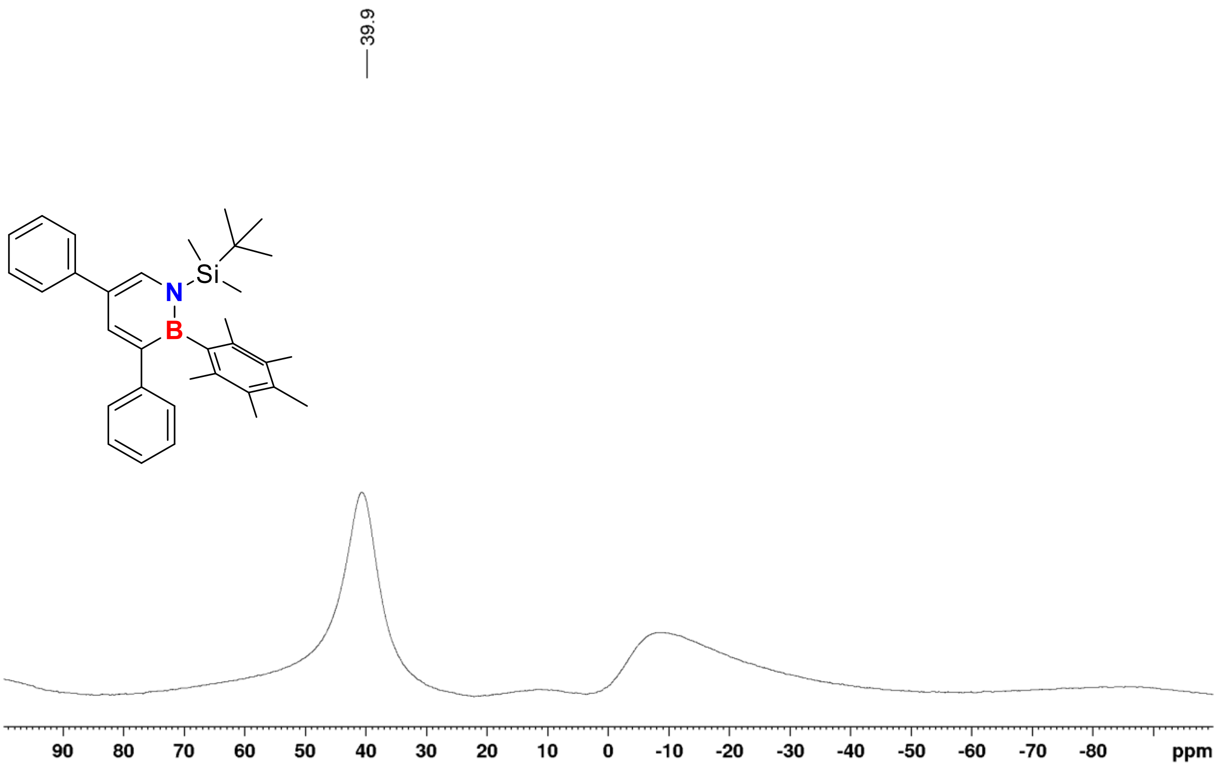

Figure S9. ^{11}B-{^{1}H}-NMR spectrum of compound ^{BN}**B3$_{H}$** in C_6D_{12} measured at a 600 MHz spectrometer.

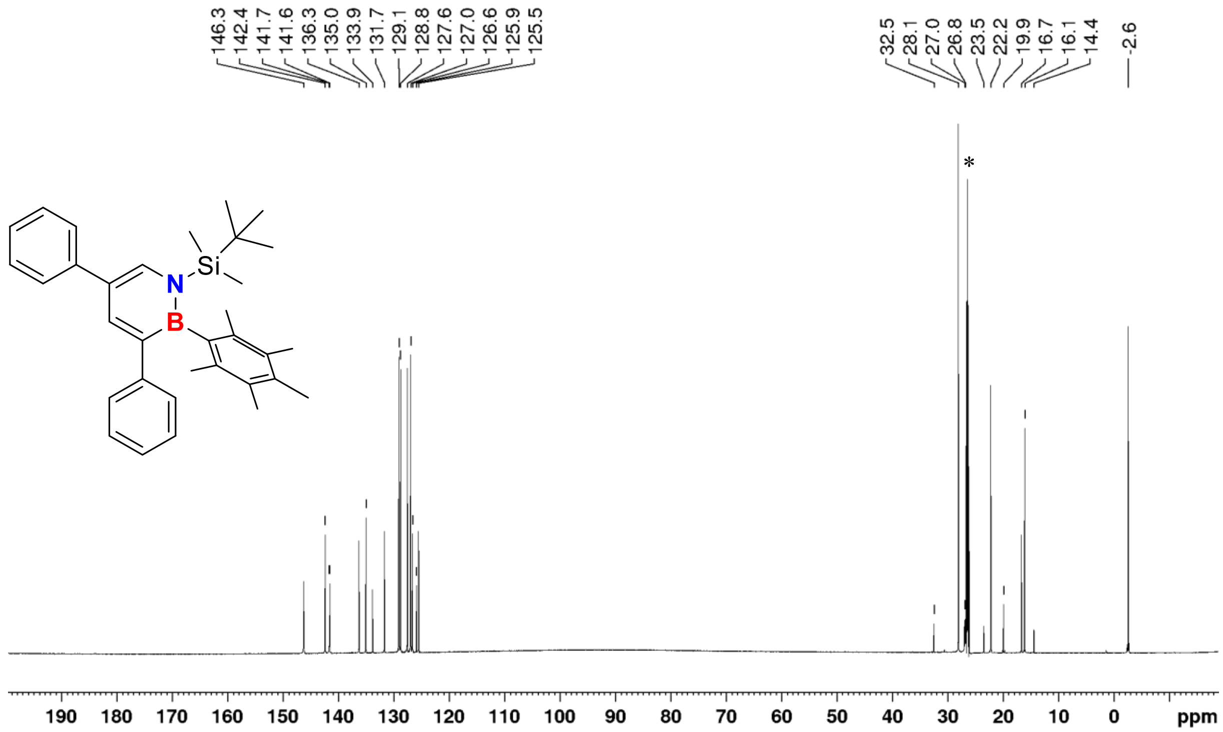

Figure S10. ^{13}C-{^{1}H}-NMR spectrum of compound ***BN*B3H** in C_6D_{12} measured at a 600 MHz spectrometer. The solvent signal is marked with an asterisk.

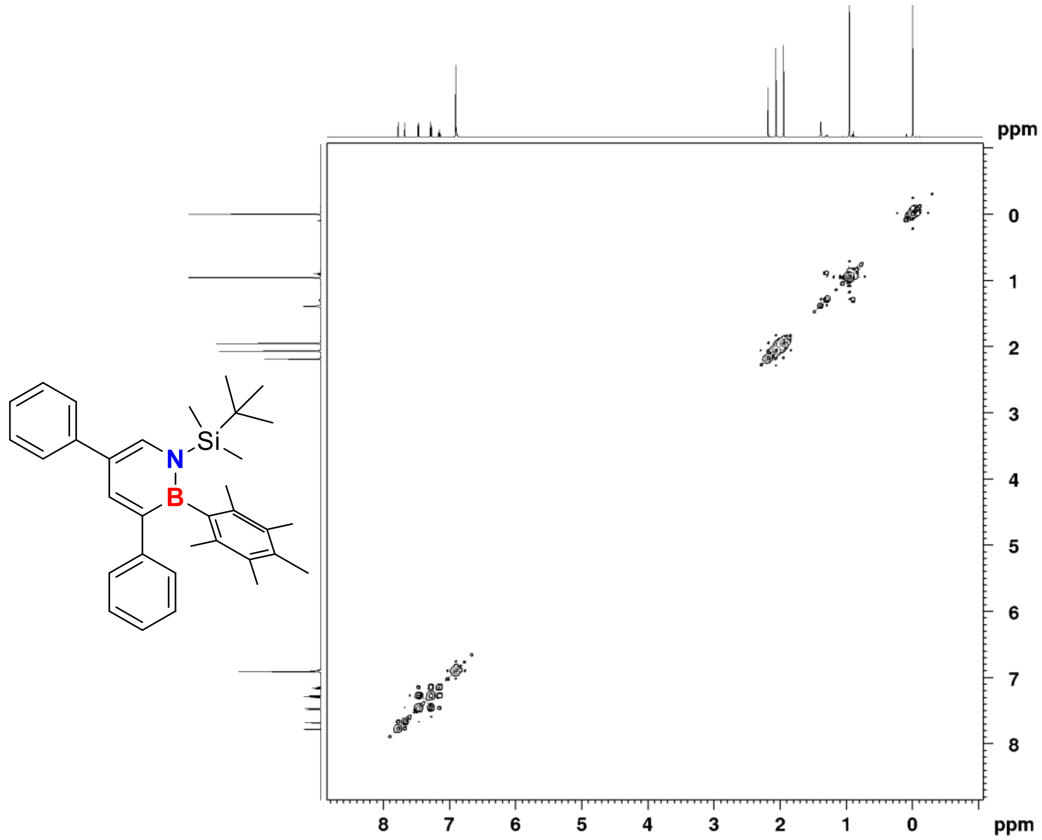

Figure S11. ^{1}H-^{1}H-NMR spectrum of compound ***BN*B3H** in C_6D_{12} measured at a 600 MHz spectrometer.

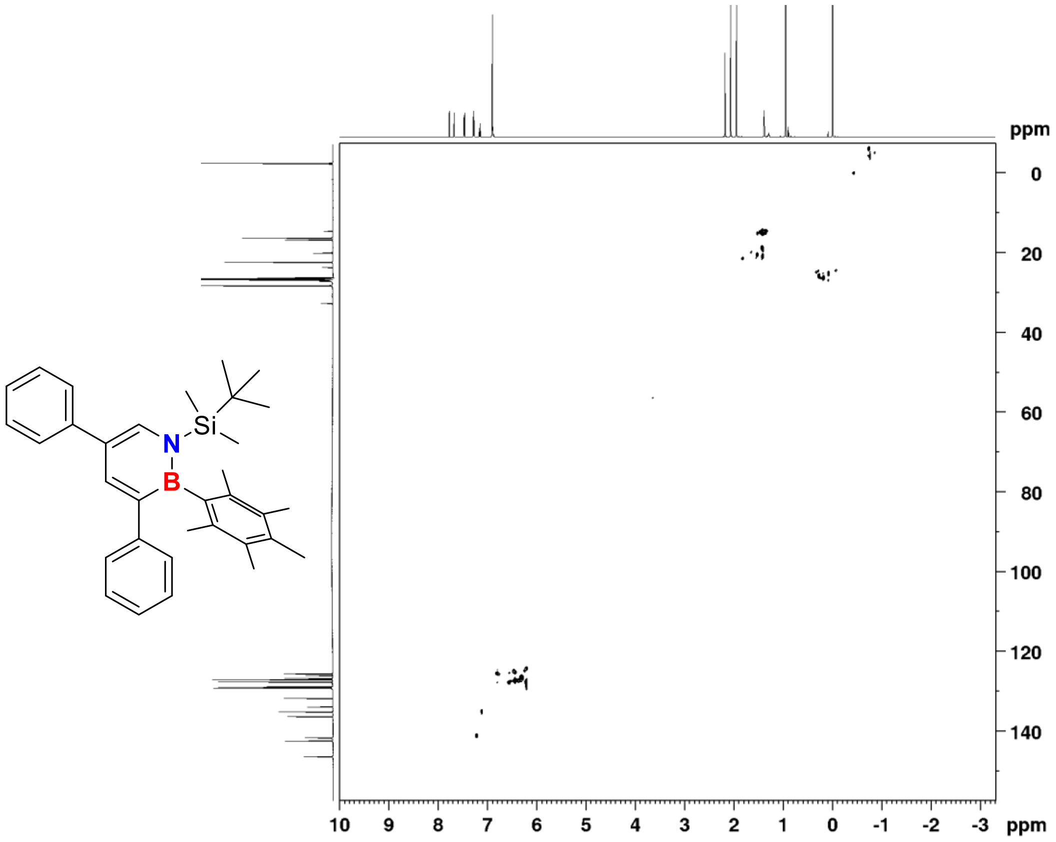

Figure S12. ^{1}H -^{13}C-HSQC-NMR spectrum of compound ^{BN}**B3**$_{\mathbf{H}}$ in C_6D_{12} measured at a 600 MHz spectrometer.

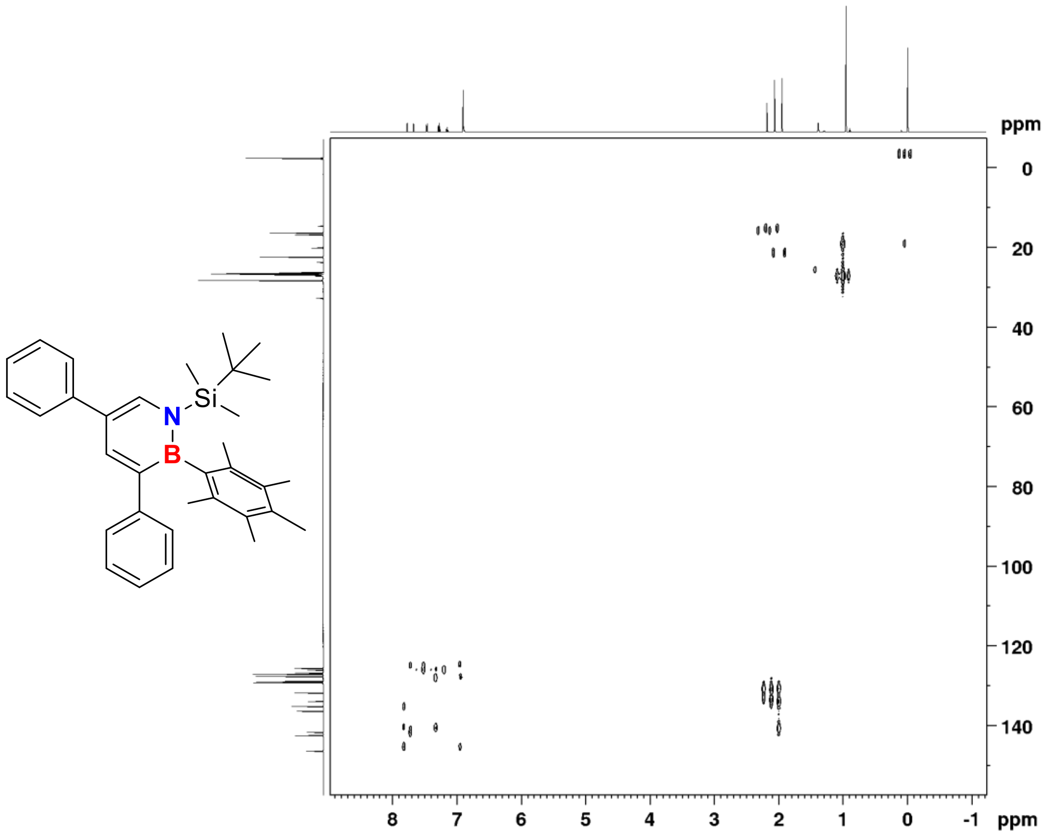

Figure S13. ^{1}H -^{13}C-HMBC-NMR spectrum of compound ^{BN}**B3**$_{\mathbf{H}}$ in C_6D_{12} measured at a 600 MHz spectrometer.

*NMR data of ^{BN}**B3**$_{Me}$*

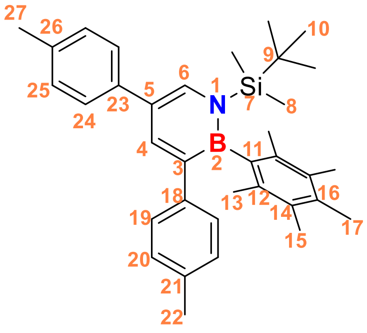

^{BN}**B3**$_{Me}$

$C_{35}H_{46}BNSi$ (519.66 g/mol)

^{1}H-NMR (400 MHz, **C_6D_{12}**): δ = 7.73 (d, $^4J_{HH}$ = 1.50 Hz, 1H, H-4), 7.61 (d, $^4J_{HH}$ = 1.50 Hz, 1H, H-6), 7.34 (d, $^3J_{HH}$ = 7.88 Hz, 2H, H-25), 7.09 (d, $^3J_{HH}$ = 7.88 Hz, 2H, H-24), 6.81 (d, $^3J_{HH}$ = 8.04 Hz, 2H, H-19), 6.73 (d, $^3J_{HH}$ = 8.04 Hz, 2H, H-20), 2.31 (s, 3H, H-27), 2.18 (s, 3H, H-17), 2.14 (s, 3H, H-22), 2.06 (s, 6H, H-15), 1.95 (s, 6H, H-13), 0.94 (s, 9H, H-10), -0.04 (s, 6H, H-8) ppm.

^{13}C-{^{1}H}-NMR (100 MHz, **C_6D_{12}**): δ = 146.0, 143.6, 142.3, 141.9, 138.9, 135.8, 135.8, 135.1, 134.4, 133.7, 131.6, 129.8, 128.7, 128.4, 126.9, 28.1, 22.2, 21.3, 21.2, 19.9, 16.7, 16.1, -2.6 ppm.

^{11}B-{^{1}H}-NMR (128 MHz, **C_6D_{12}**): δ = 39.5 ppm.

HR-MS (ESI): m/z calc. For [M+Na]+ 542.33848, found 543.33953.

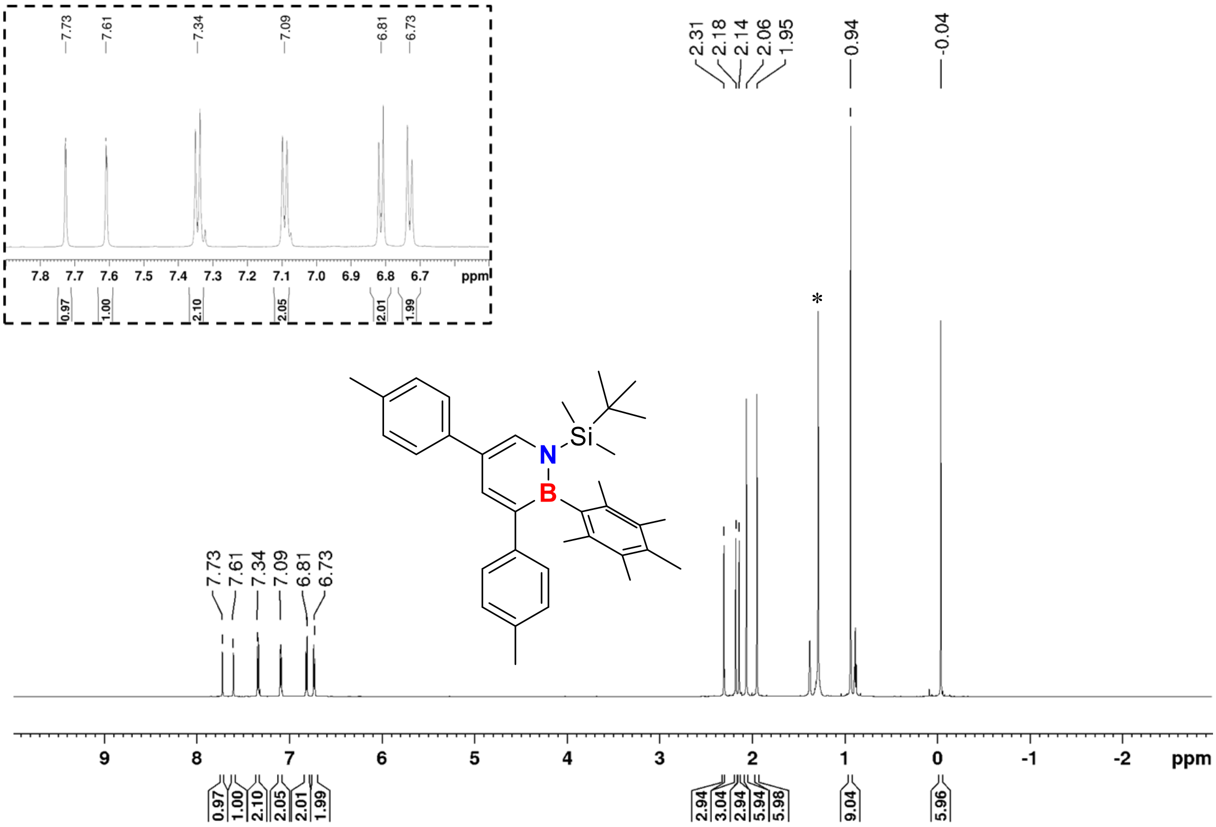

Figure S14. ^{1}H-NMR spectrum of compound ^{BN}**B3$_{Me}$** in C_6D_{12} measured at a 600 MHz spectrometer. The solvent signal is marked with an asterisk.

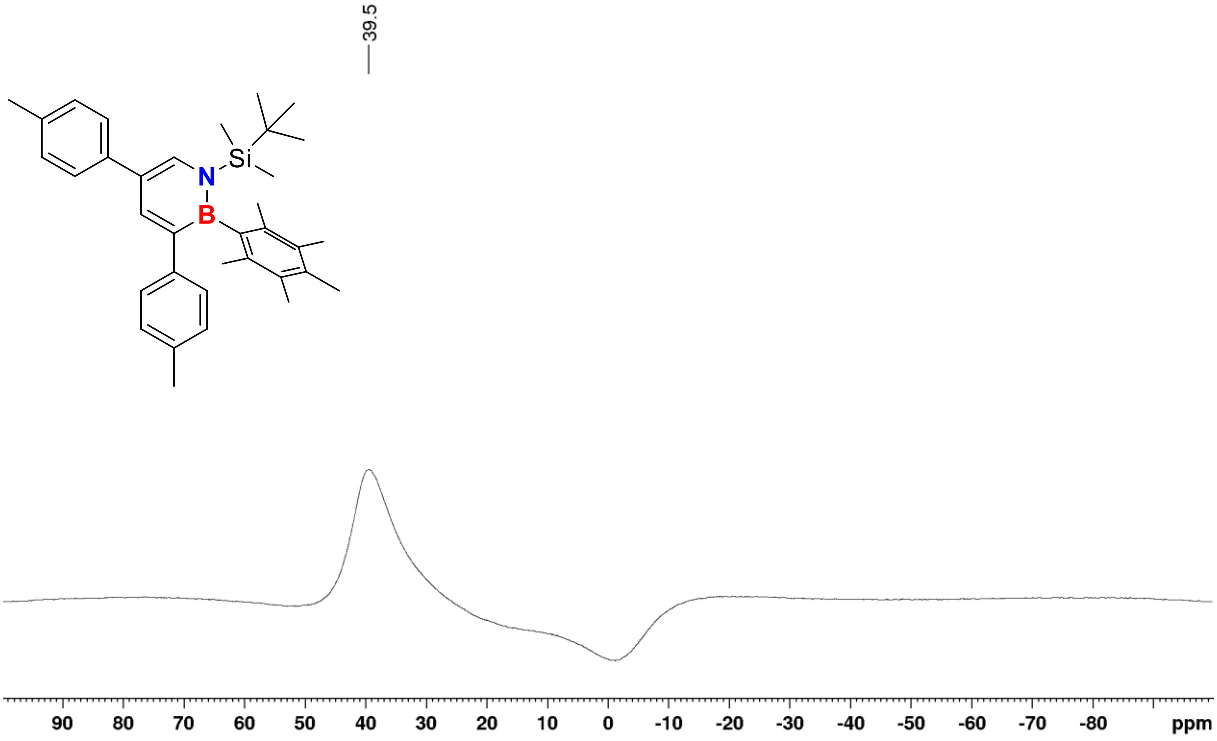

Figure S15. ^{11}B -{^{1}H}-NMR spectrum of compound ^{BN}**B3$_{Me}$** in C_6D_{12} measured at a 600 MHz spectrometer.

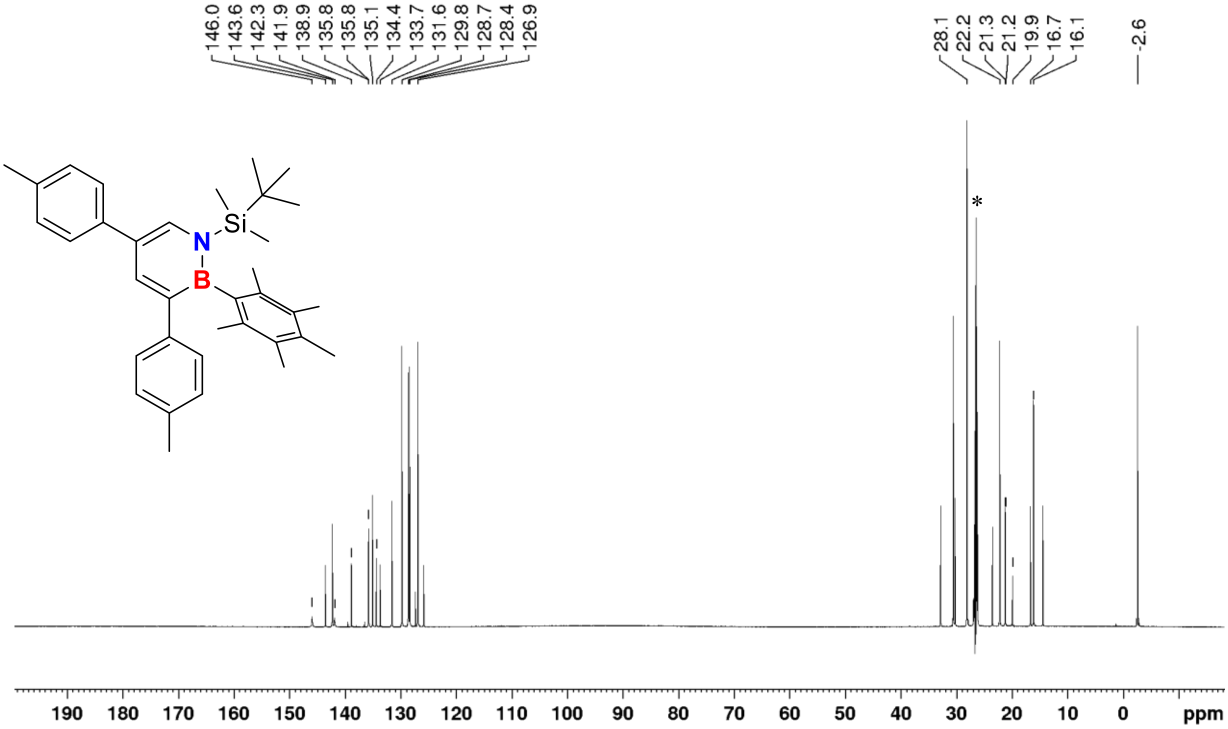

Figure S16. ^{13}C-{^{1}H}-NMR spectrum of compound ^{BN}**B3**$_{\mathbf{Me}}$ in C_6D_{12} measured at a 600 MHz spectrometer. The solvent signal is marked with an asterisk.

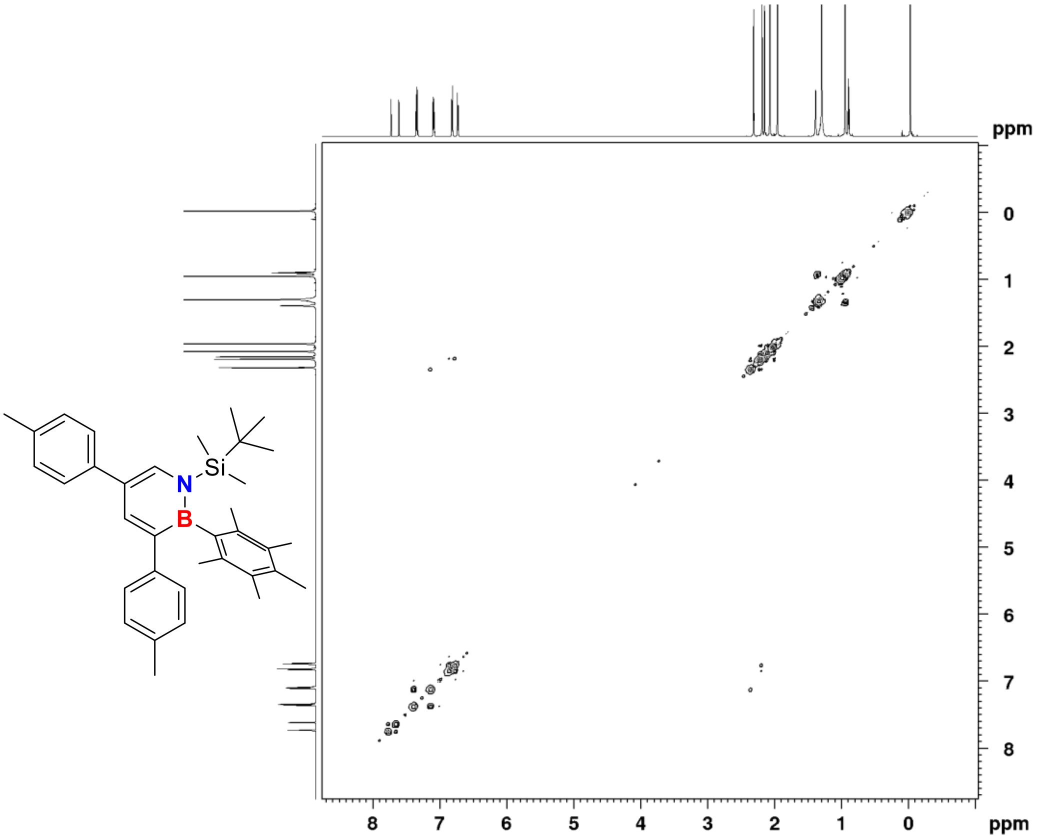

Figure S17. ^{1}H-^{1}H-NMR spectrum of compound ^{BN}**B3**$_{\mathbf{Me}}$ in C_6D_{12} measured at a 600 MHz spectrometer.

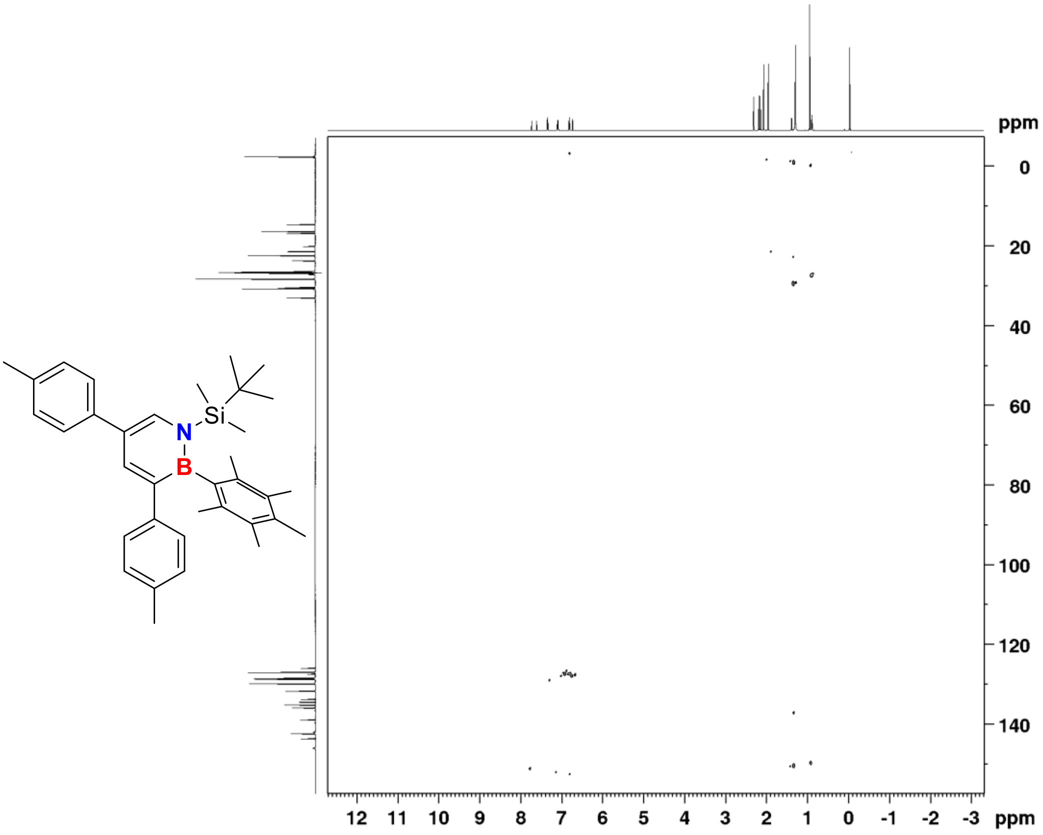

Figure S18. ^{1}H -^{13}C-HSQC-NMR spectrum of compound ^{BN}**B3**$_{\mathbf{Me}}$ in C_6D_{12} measured at a 600 MHz spectrometer.

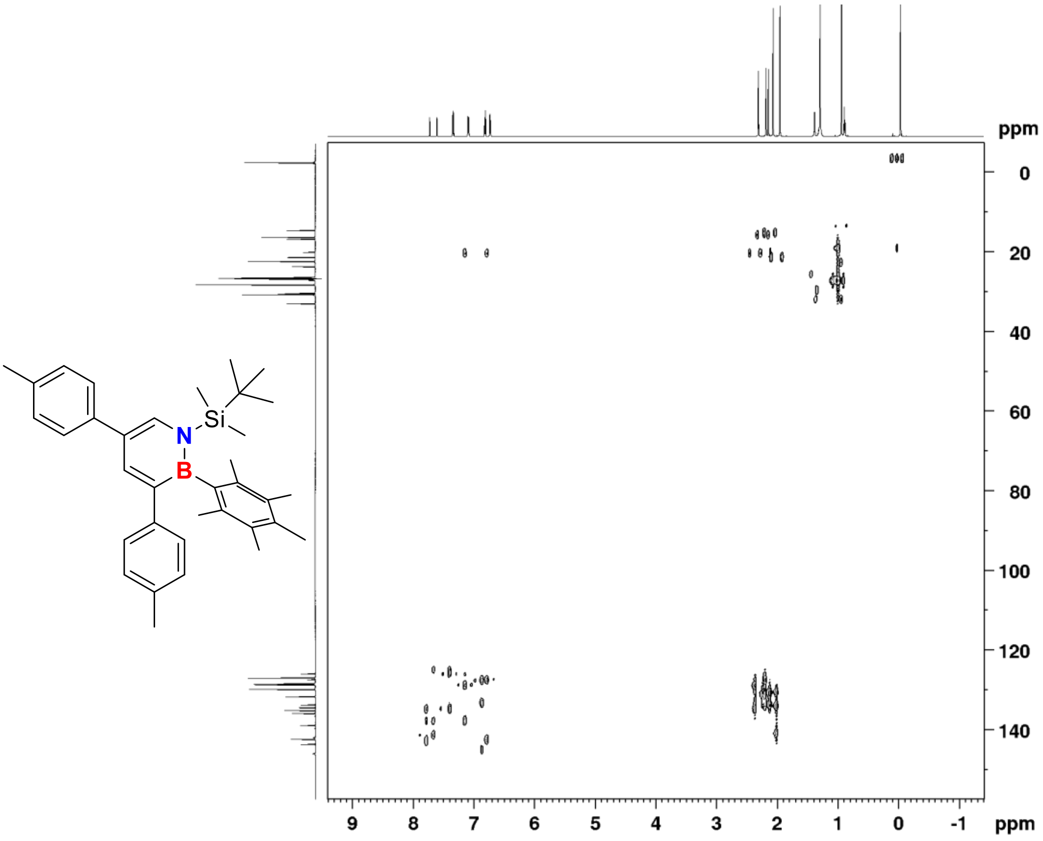

Figure S19. ^{1}H -^{13}C-HMBC-NMR spectrum of compound ^{BN}**B3**$_{\mathbf{Me}}$ in C_6D_{12} measured at a 600 MHz spectrometer.

*NMR data of ^{BN}**B3$_{OMe}$***

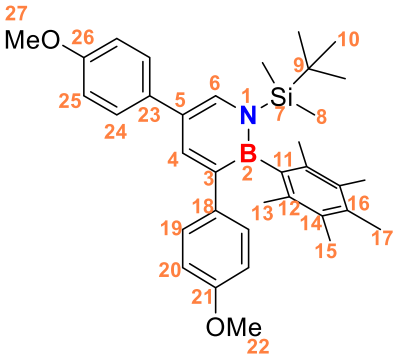

^{BN}**B3$_{OMe}$**

$C_{35}H_{46}BO_2NSi$ (551.65 g/mol)

^{1}H-NMR (400 MHz, **C_6D_{12}**): δ = 7.69 (d, $^4J_{HH}$ = 1.69 Hz, 1H, H-4), 7.55 (d, $^4J_{HH}$ = 1.69 Hz, 1H, H-6), 7.26 (d, $^3J_{HH}$ = 8.29 Hz, 2H, H-20), 6.83 (m, 4H, H-19/H-25), 6.47 (d, $^3J_{HH}$ = 8.78 Hz, 2H, H-24), 3.71 (s, 3H, H-22), 3.57 (s, 3H, H-27), 2.19 (s, 3H, H-17), 2.07 (s, 6H, H-15), 1.94 (s, 6H, H-13), 0.94 (s, 9H, H-10), -0.04 (s, 6H, H-8) ppm.

^{13}C-{^{1}H}-NMR (100 MHz, **C_6D_{12}**): δ = 158.5, 157.6, 144.6, 141.2, 137.9, 134.3, 134.2, 133.3, 132.8, 130.8, 128.7, 127.0, 124.8, 113.6, 112.3, 53.9, 53.7, 27.2, 21.2, 19.0, 15.8, 15.2, -3.5 ppm.

^{11}B-{^{1}H}-NMR (128 MHz, **C_6D_{12}**): δ = 38.6 ppm.

HR-MS (ESI): m/z calc. For [M+Na]+ 574.32831, found 574.32888.

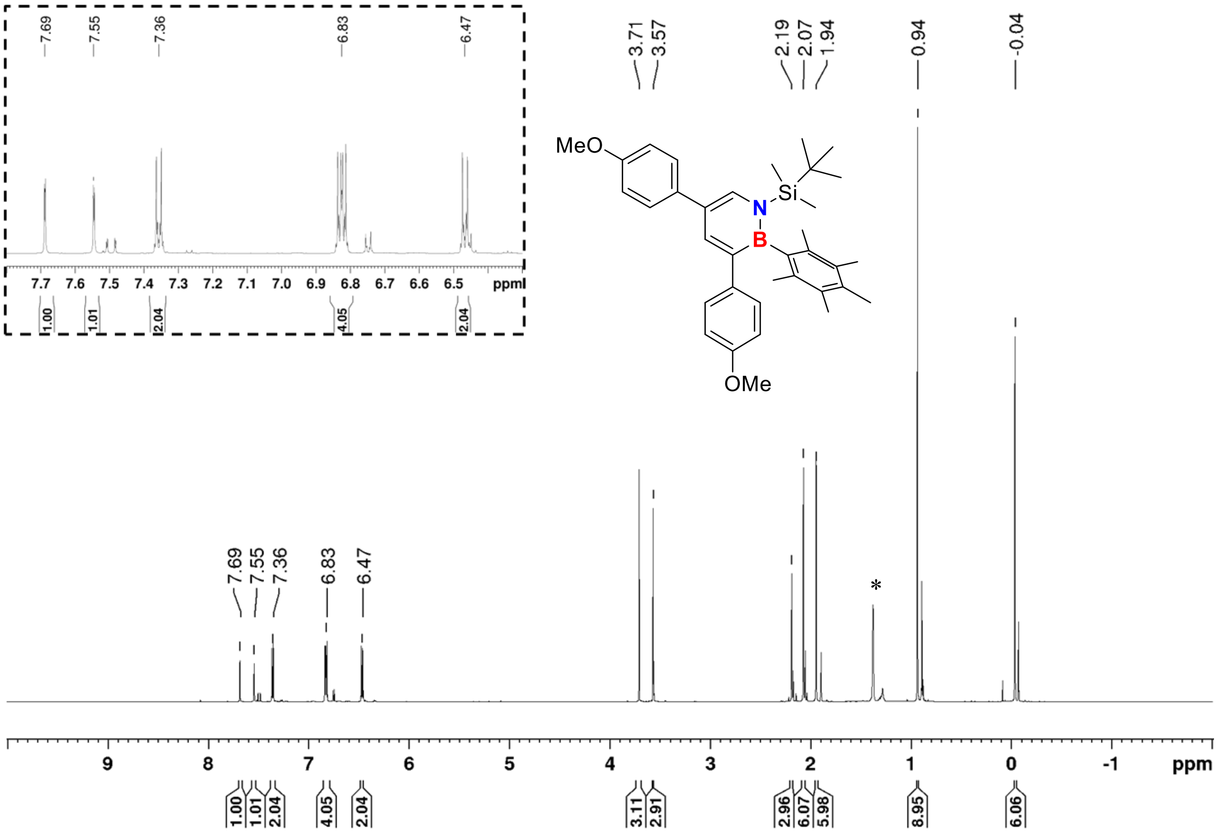

Figure S20. ^{1}H-NMR spectrum of compound ^{BN}**B3**$_{\mathbf{OMe}}$ in C_6D_{12} measured at a 600 MHz spectrometer. The solvent signal is marked with an asterisk.

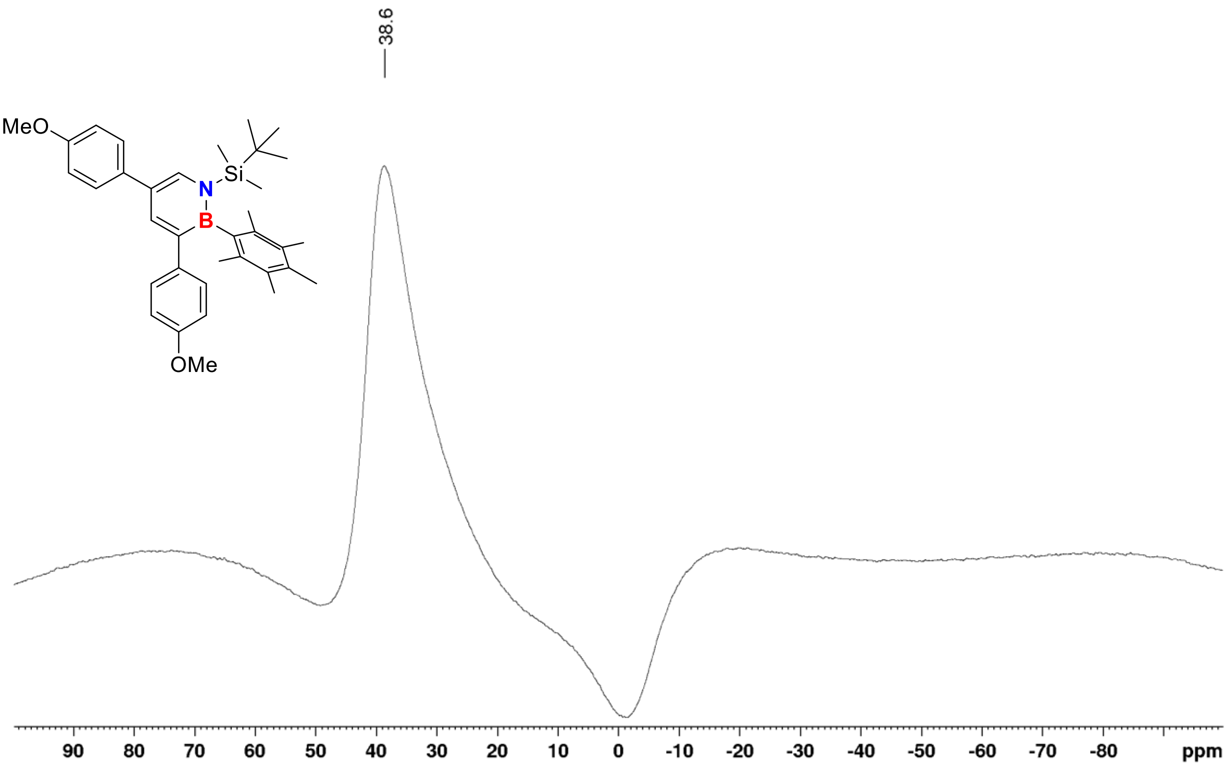

Figure S21. ^{11}B-{^{1}H}-NMR spectrum of compound ^{BN}**B3**$_{\mathbf{OMe}}$ in C_6D_{12} measured at a 600 MHz spectrometer.

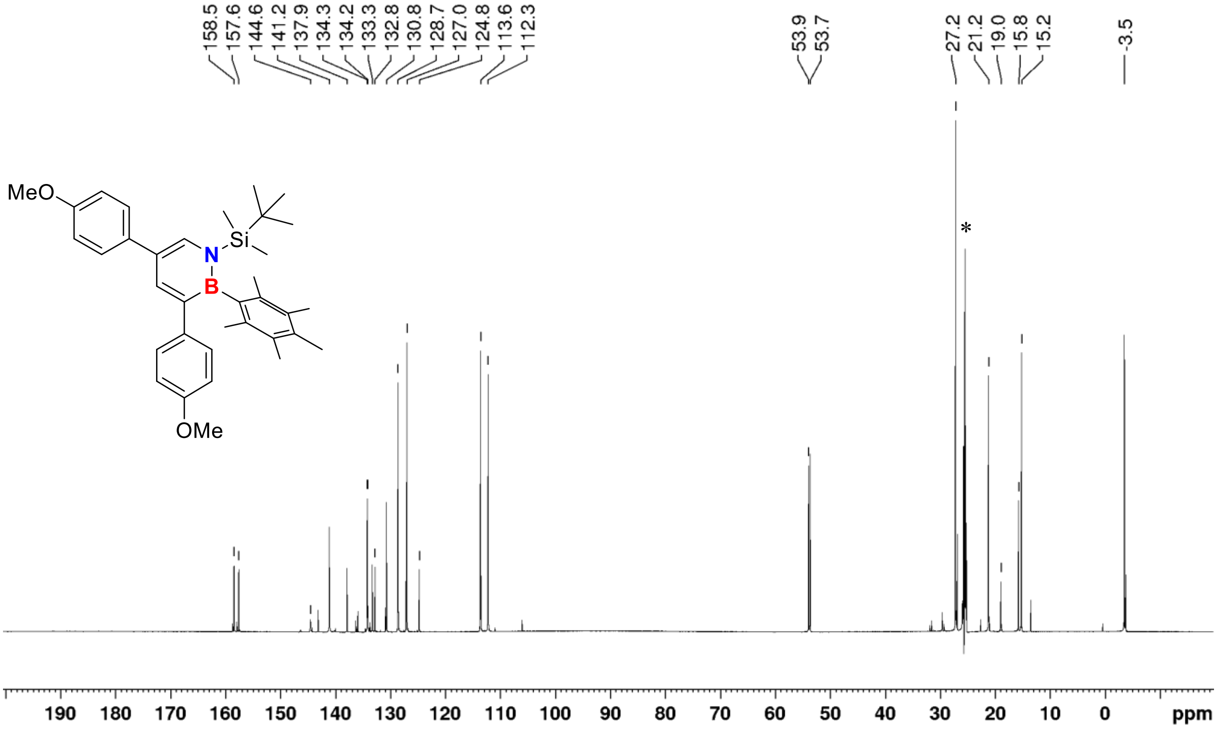

Figure S22. ^{13}C-{^{1}H}-NMR spectrum of compound ^{BN}**B3**$_{\mathbf{OMe}}$ in C_6D_{12} measured at a 600 MHz spectrometer. The solvent signal is marked with an asterisk.

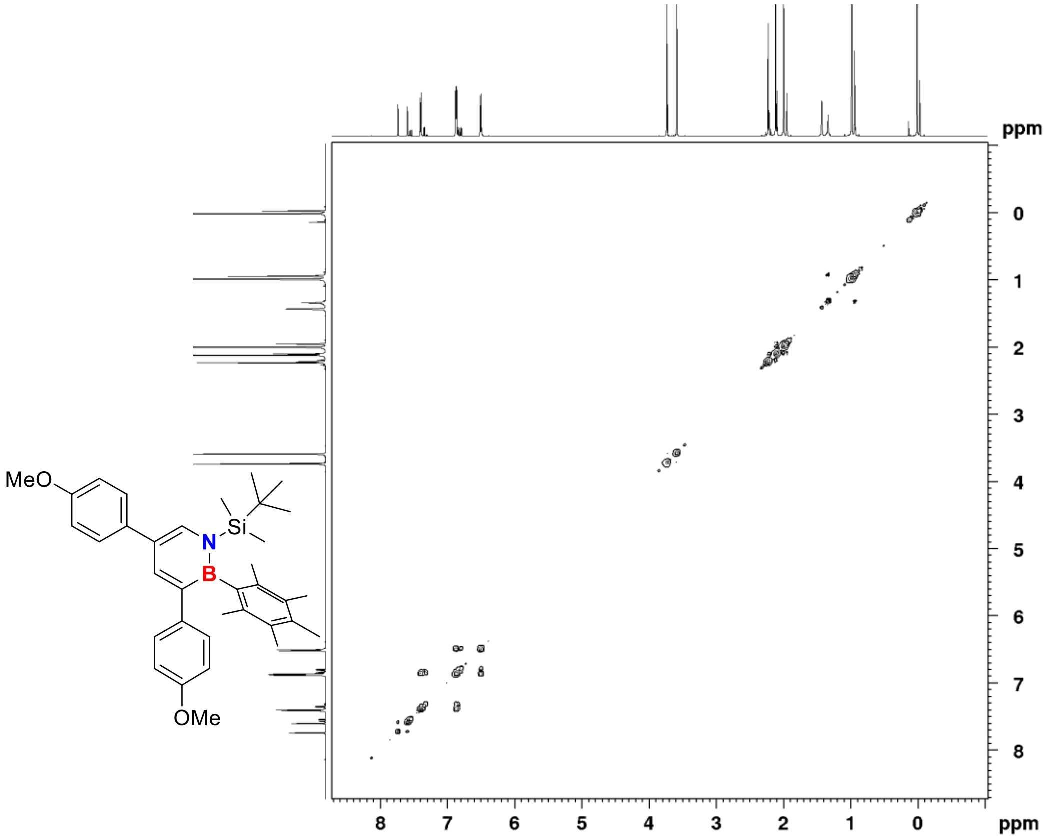

Figure S23. ^{1}H-^{1}H-COSY-NMR spectrum of compound ^{BN}**B3**$_{\mathbf{OMe}}$ in C_6D_{12} measured at a 600 MHz spectrometer.

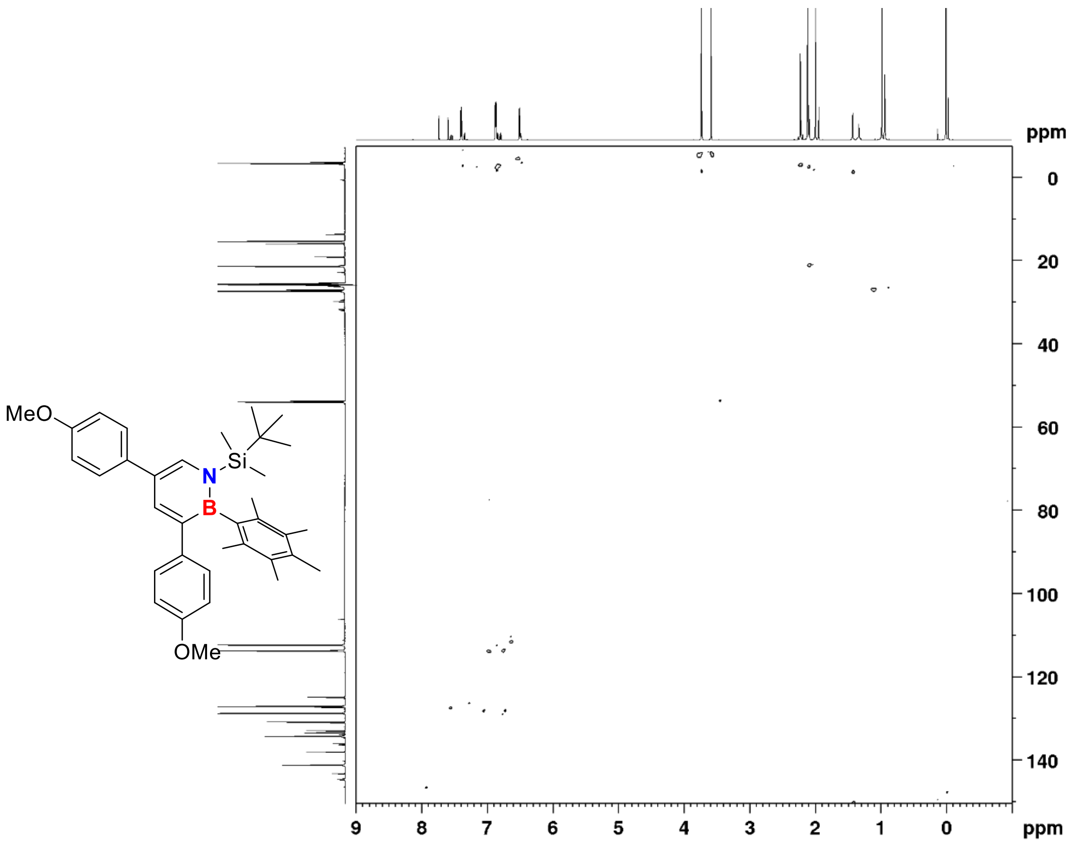

Figure S24. ^{1}H -^{13}C-HSQC-NMR spectrum of compound ^{BN}**B3$_{OMe}$** in C_6D_{12} measured at a 600 MHz spectrometer.

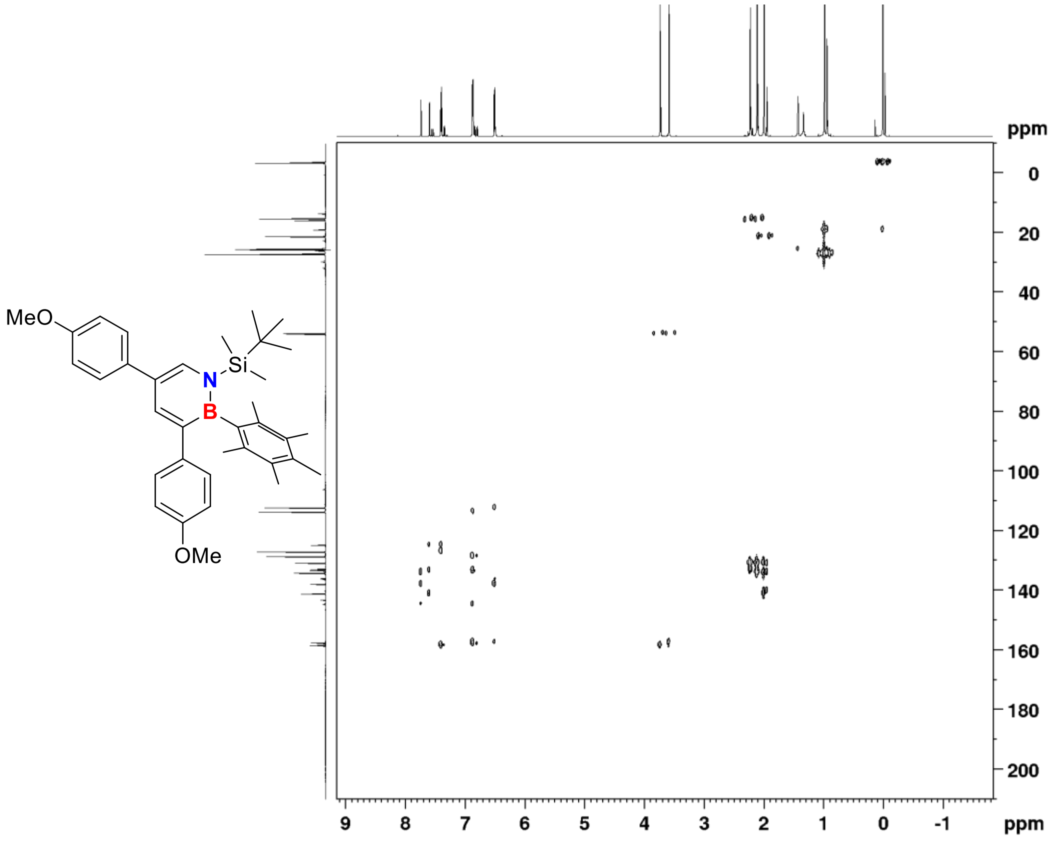

Figure S25. ^{1}H-^{13}C-HMBC-NMR spectrum of compound ^{BN}**B3$_{Me}$** in C_6D_{12} measured at a 600 MHz spectrometer.

NMR data of ***^{BN}B3$_{SMe}$***

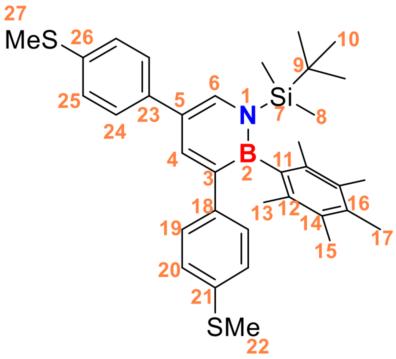

^{BN}B3$_{SMe}$

$C_{35}H_{46}BS_2NSi$ (583.78 g/mol)

^{1}H-NMR (400 MHz, **C_6D_{12}**): δ = 7.72 (d, $^{4}J_{HH}$ = 1.68 Hz, 1H, H-4), 7.63 (d, $^{4}J_{HH}$ = 1.68 Hz, 1H, H-6), 7.37 (d, $^{3}J_{HH}$ = 8.57 Hz, 2H, H-25), 7.22 (d, $^{3}J_{HH}$ = 8.57 Hz, 2H, H-24), 6.85 (m, 4H, H-19/H-20), 2.39 (s, 3H, H-27), 2.26 (s, 3H, H-22), 2.19 (s, 3H, H-17), 2.08 (s, 6H, H-15), 1.95 (s, 6H, H-13), 0.94 (s, 9H, H-10), -0.03 (s, 6H, H-8) ppm.

^{13}C-{^{1}H}-NMR (100 MHz, **C_6D_{12}**): δ = 142.1, 141.6, 138.5, 137.6, 136.2, 135.0, 134.0, 131.8, 129.1, 128.2, 127.3, 127.0, 125.4, 32.8, 30.3, 28.1, 22.2, 19.9, 16.7, 16.1, -2.6 ppm.

^{11}B-{^{1}H}-NMR (128 MHz, **C_6D_{12}**): δ = 40.9 ppm.

HR-MS (ESI): m/z calc. For [M+Na]+ 606.28262, found 606.28356.

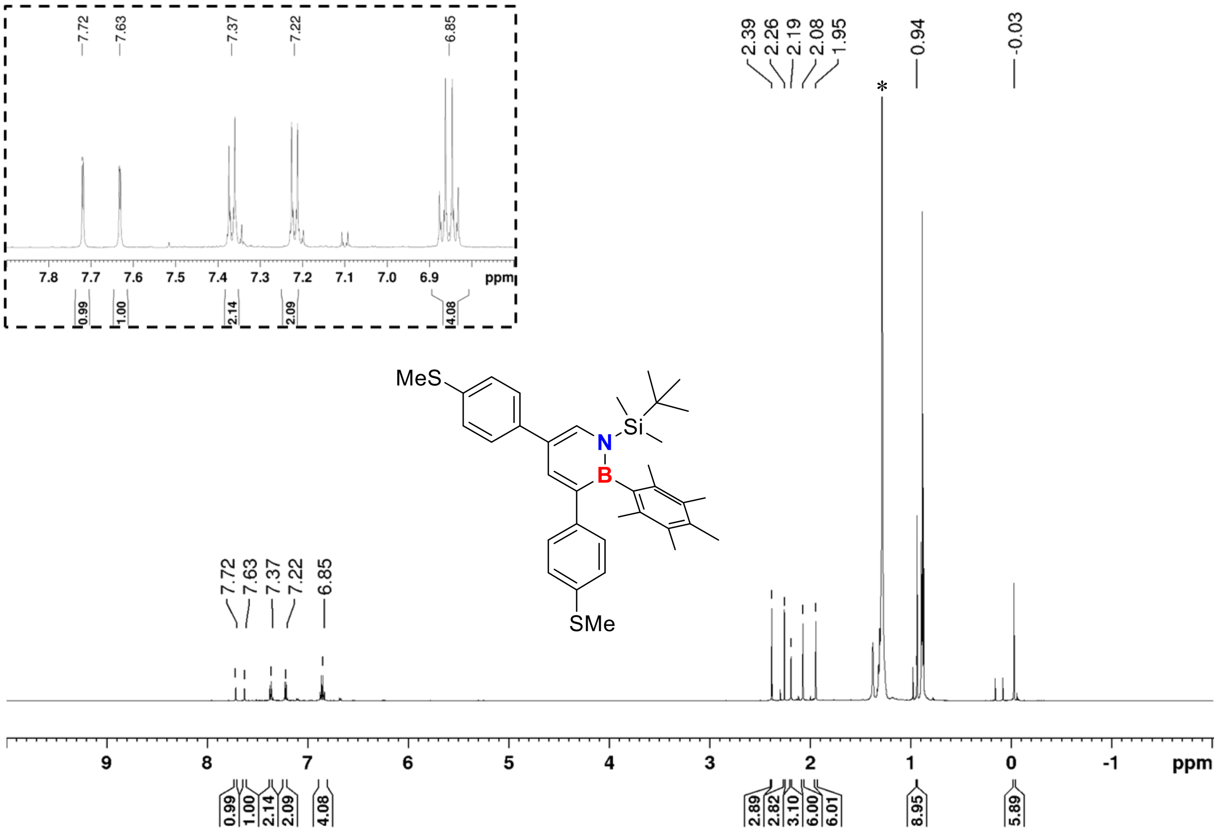

Figure S26. ^{1}H-NMR spectrum of compound $^{BN}\mathbf{B3}_{\mathbf{SMe}}$ in C_6D_{12} measured at a 600 MHz spectrometer. The solvent signal is marked with an asterisk.

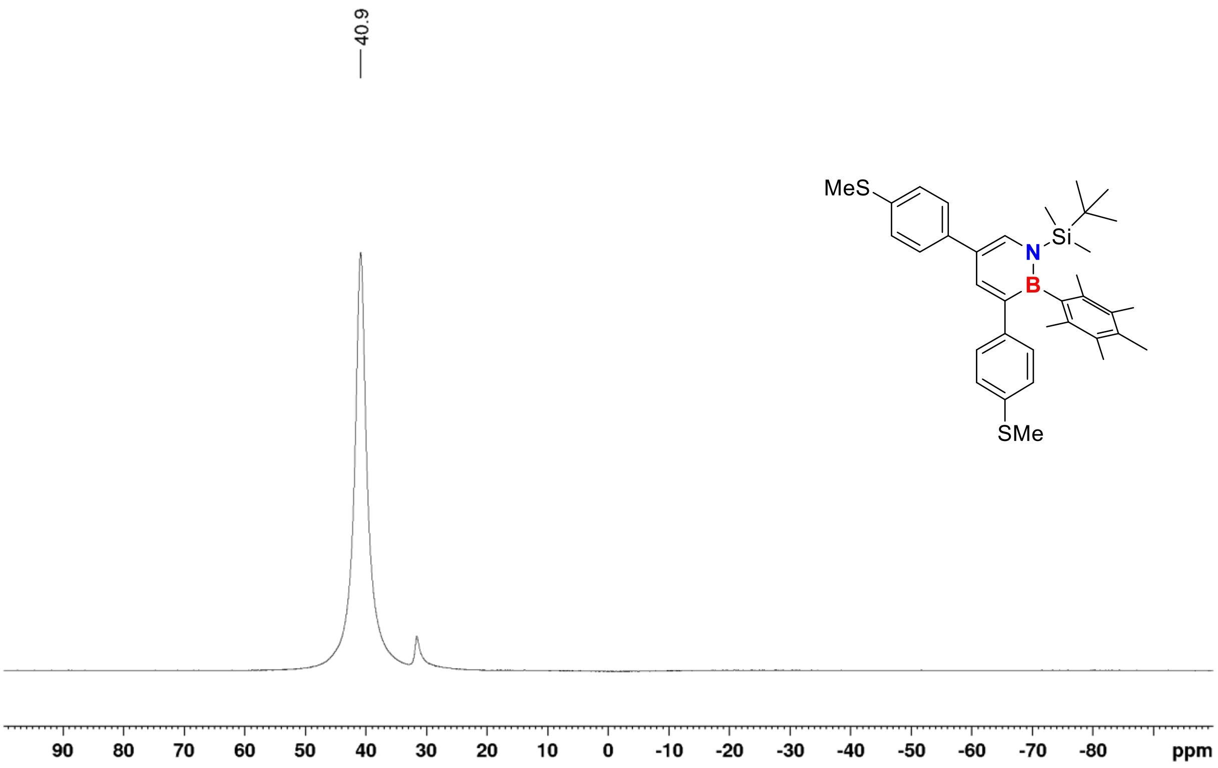

Figure S27. ^{11}B-{^{1}H}-NMR spectrum of compound $^{BN}\mathbf{B3}_{\mathbf{SMe}}$ in C_6D_{12} measured at a 600 MHz spectrometer.

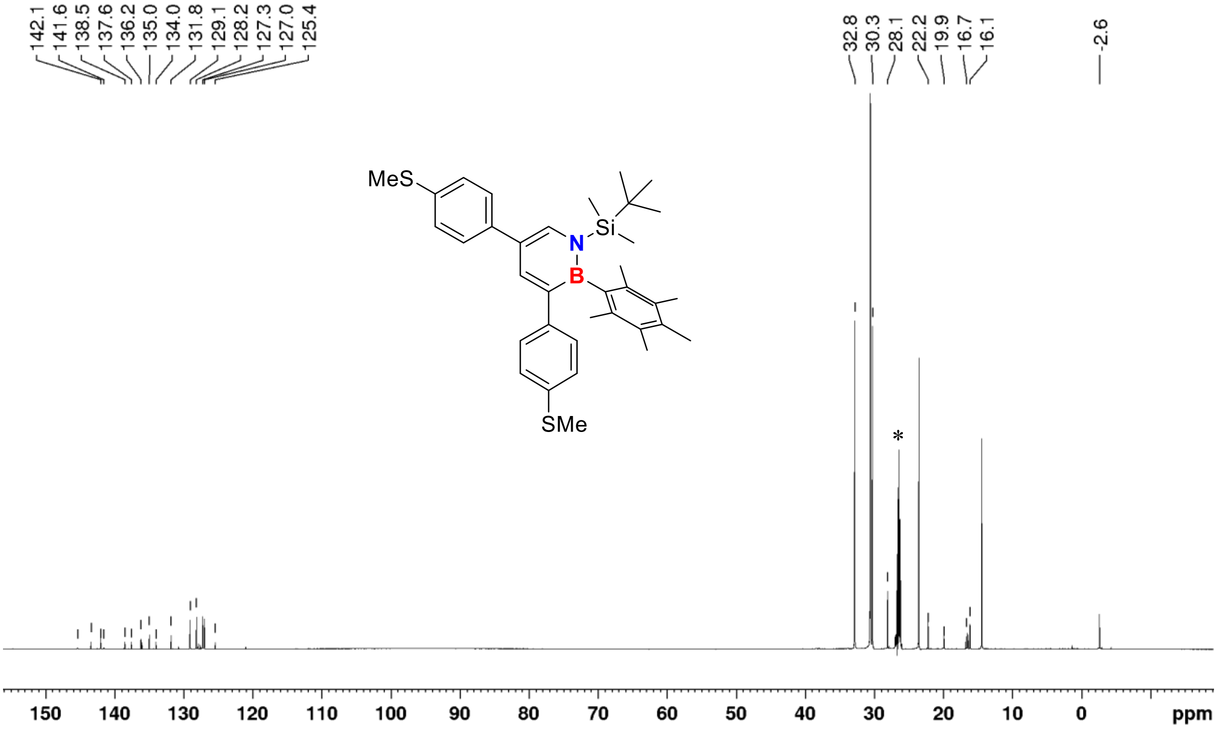

Figure S28. ^{13}C -{^{1}H}-NMR spectrum of compound ***BN*B3SMe** in C_6D_{12} measured at a 600 MHz spectrometer. The solvent signal is marked with an asterisk.

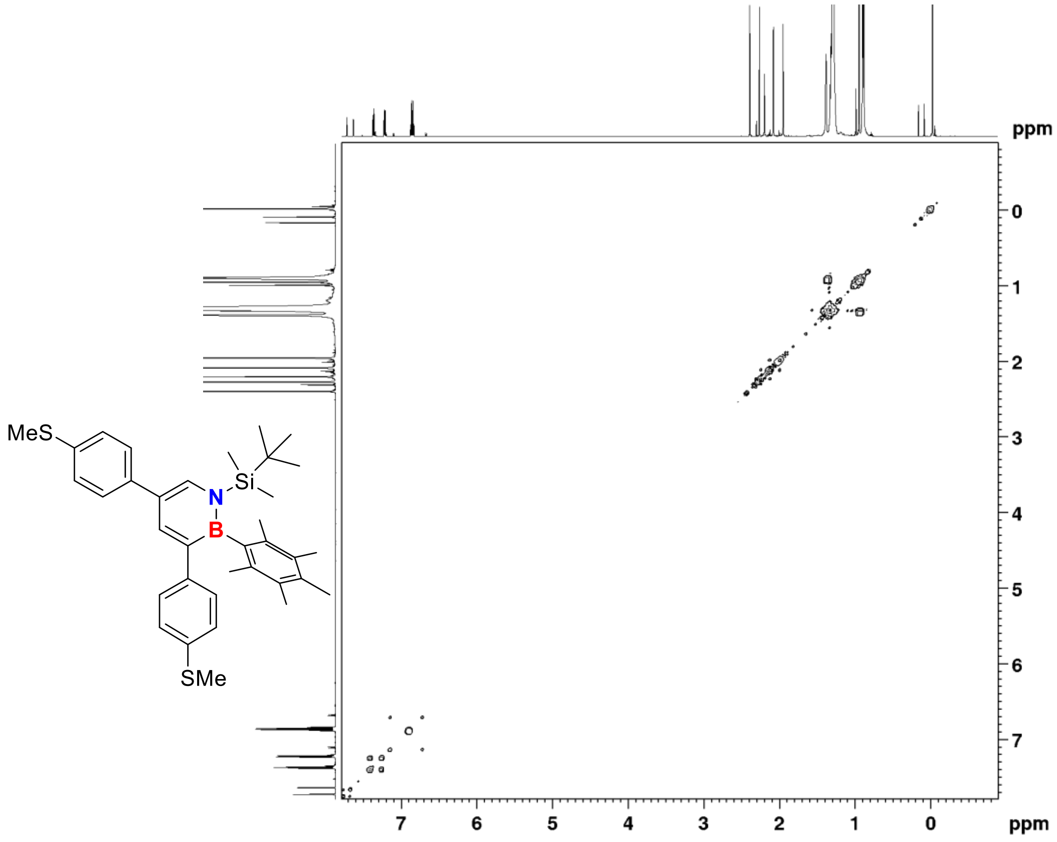

Figure S29. ^{1}H -^{1}H-NMR spectrum of compound ***BN*B3SMe** in C_6D_{12} measured at a 600 MHz spectrometer.

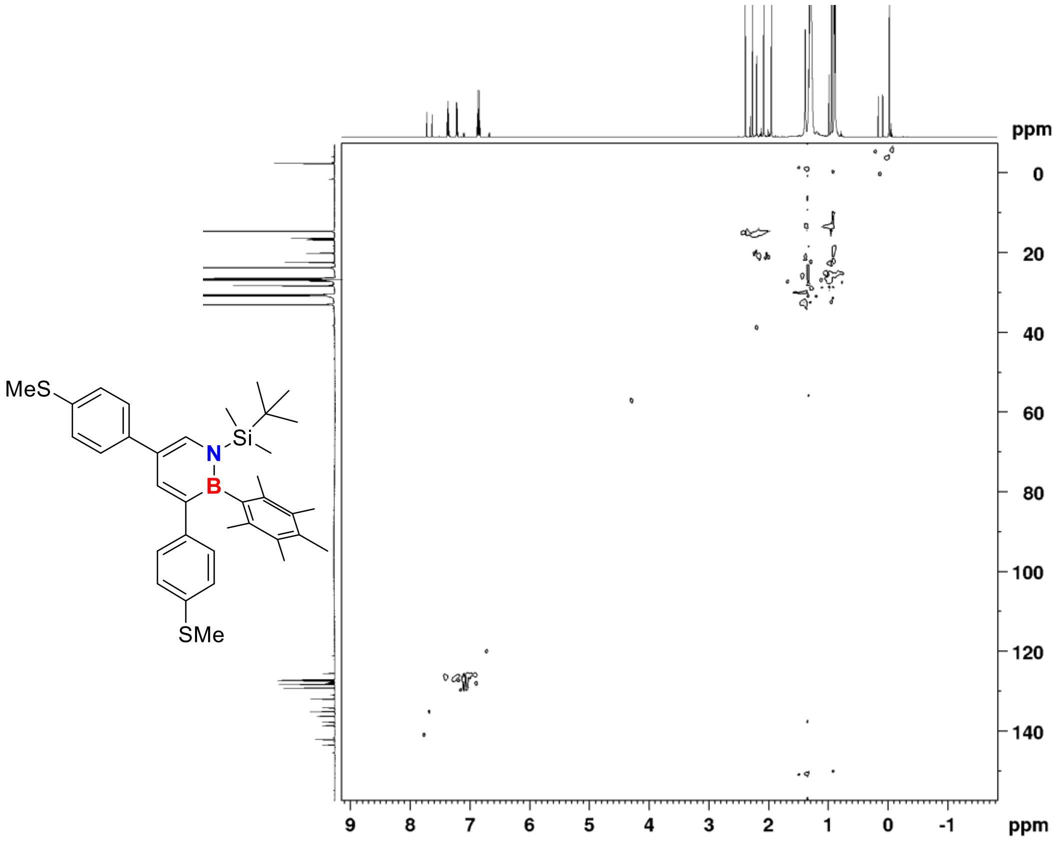

Figure S30. ^{1}H -^{13}C-HSQC-NMR spectrum of compound ^{BN}**B3**$_{\mathbf{SMe}}$ in C_6D_{12} measured at a 600 MHz spectrometer.

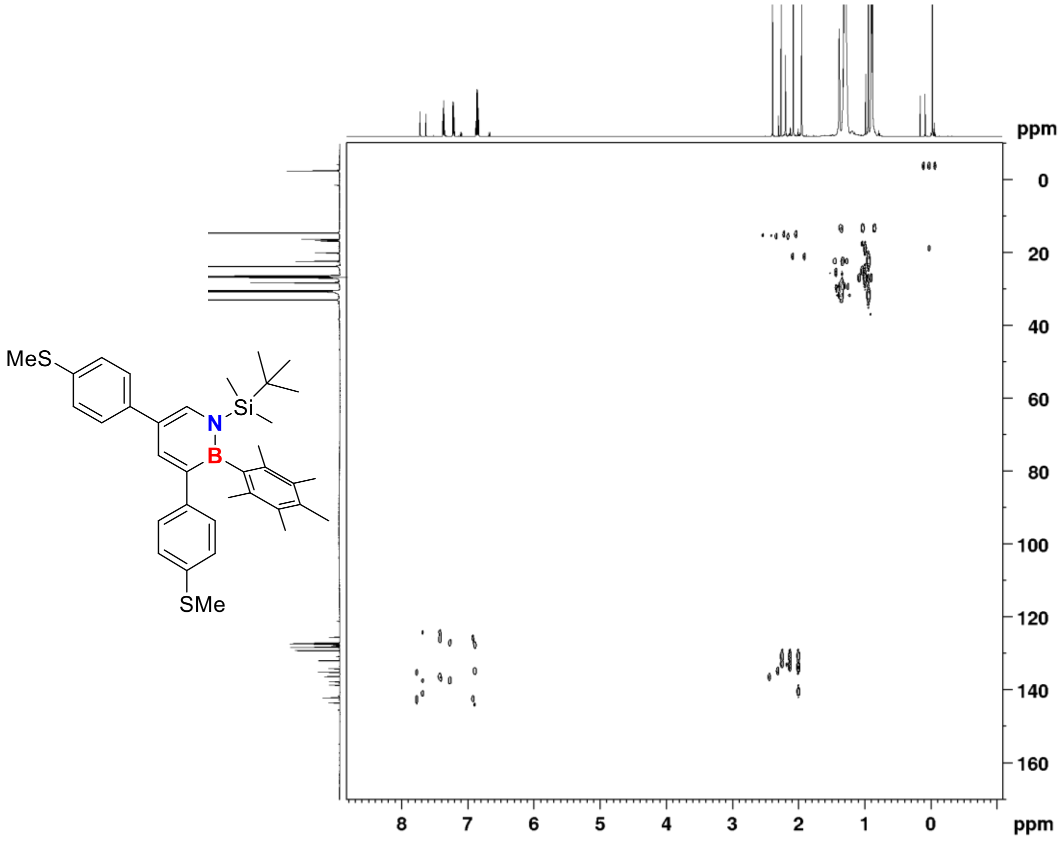

Figure S31. ^{1}H -^{13}C-NMR spectrum of compound ^{BN}**B3**$_{\mathbf{SMe}}$ in C_6D_{12} measured at a 600 MHz spectrometer.

NMR data of ^{BN}***B3***$_{NMe2}$

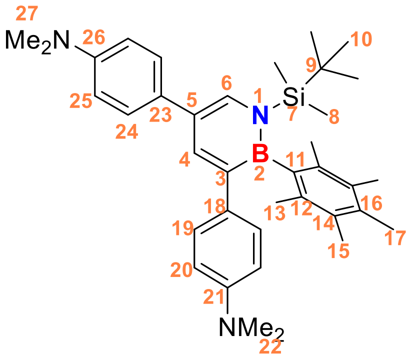

^{BN}**B3**$_{NMe2}$

$C_{37}H_{52}BN_3Si$ (577.74 g/mol)

^{1}**H-NMR** (400 MHz, $\mathbf{C_6D_{12}}$): δ = 7.70 (d, $^{4}J_{HH}$ = 1.71 Hz, 1H, H-4), 7.50, (d, $^{4}J_{HH}$ = 1.71 Hz, 1H, H-6), 7.32 (d, $^{4}J_{HH}$ = 8.74 Hz, 2H, H-25), 6.81 (d, $^{4}J_{HH}$ = 8.85 Hz, 2H, H-24), 6.68 (d, $^{4}J_{HH}$ = 8.74 Hz, 2H, H-20), 6.34 d, $^{4}J_{HH}$ = 8.85 Hz, 2H, H-19), 2.89 (s, 6H, H-27), 2.75 (s, 6H, H-22), 2.19 (s, 3H, H-17), 2.08 (s, 6H, H-15), 1.96 (s, 6H, H-13), 0.94 (s, 9H, H-10), -0.06 (s, 6H, H-8) ppm.

^{13}**C-{^{1}H}-NMR** (100 MHz, $\mathbf{C_6D_{12}}$): δ = 149.9, 149.1, 145.5, 142.6, 141.7, 135.6, 135.3, 134.1, 133.4, 131.5,129.3, 127.7, 126.1, 113.7, 112.7, 40.9, 40.9, 28.2, 22.1, 19.9, 16.7, 16.2, -2.6 ppm.

^{11}**B-{^{1}H}-NMR** (128 MHz, $\mathbf{C_6D_{12}}$): δ = 40.2 ppm.

HR-MS (ESI): m/z calc. For [M+H]+ 578.40963, found 578.40866.

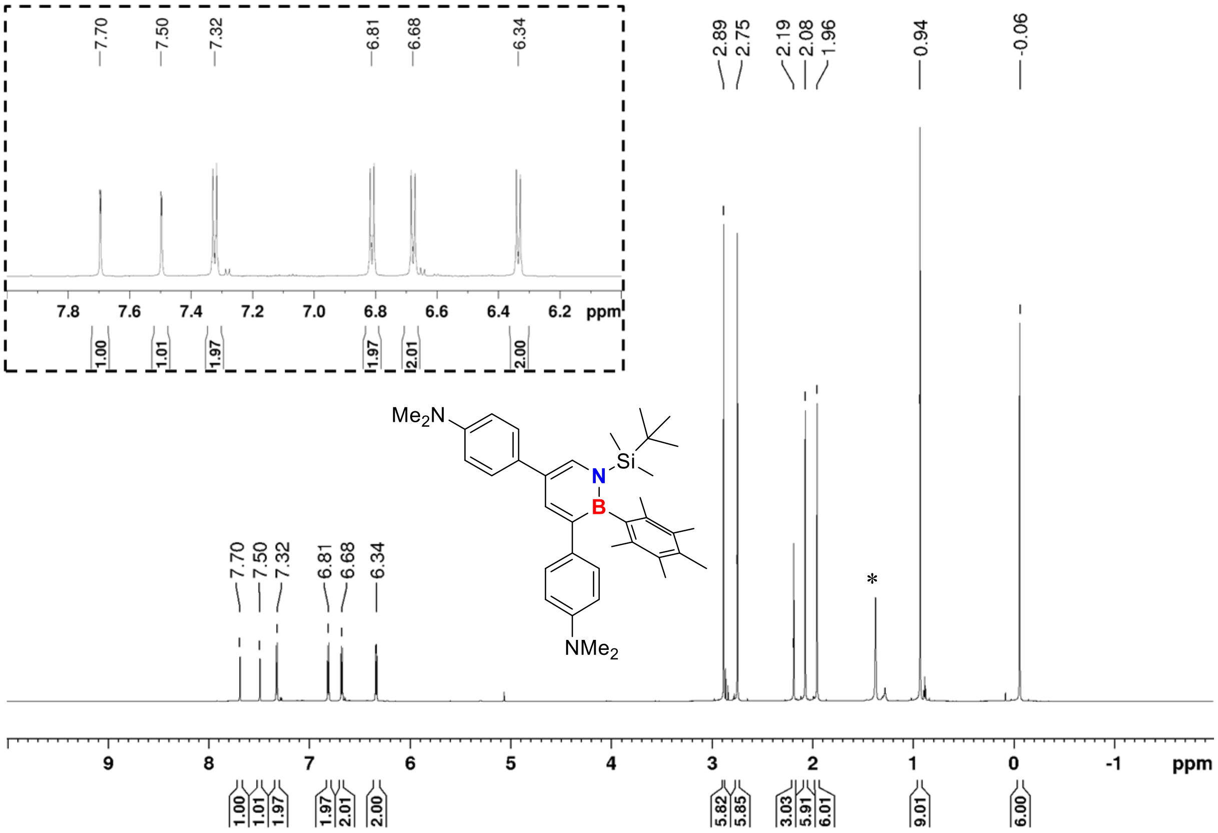

Figure S32. ^{1}H-NMR spectrum of compound ^{BN}**B3**$_{\mathbf{NMe2}}$ in C_6D_{12} measured at a 700 MHz spectrometer. The solvent signal is marked with an asterisk.

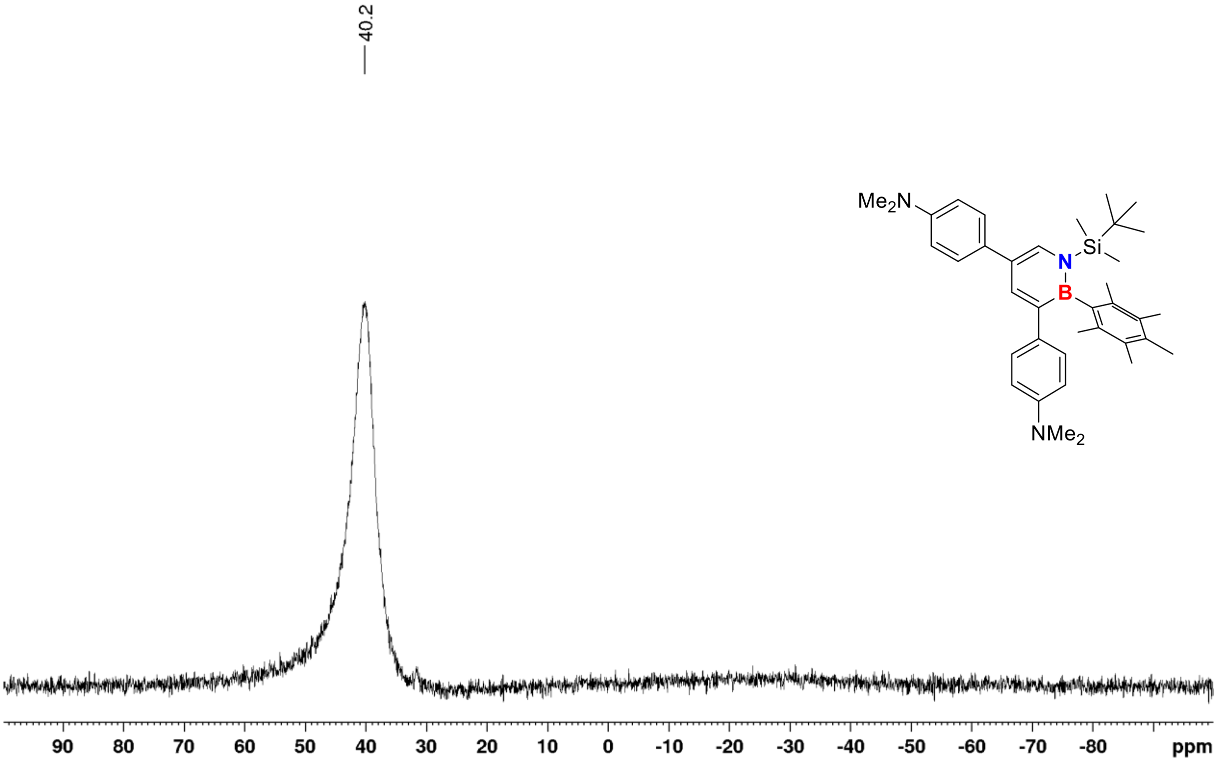

Figure S33. ^{11}B-{^{1}H}-NMR spectrum of compound ^{BN}**B3**$_{\mathbf{NMe2}}$ in C_6D_{12} measured at a 400 MHz spectrometer.

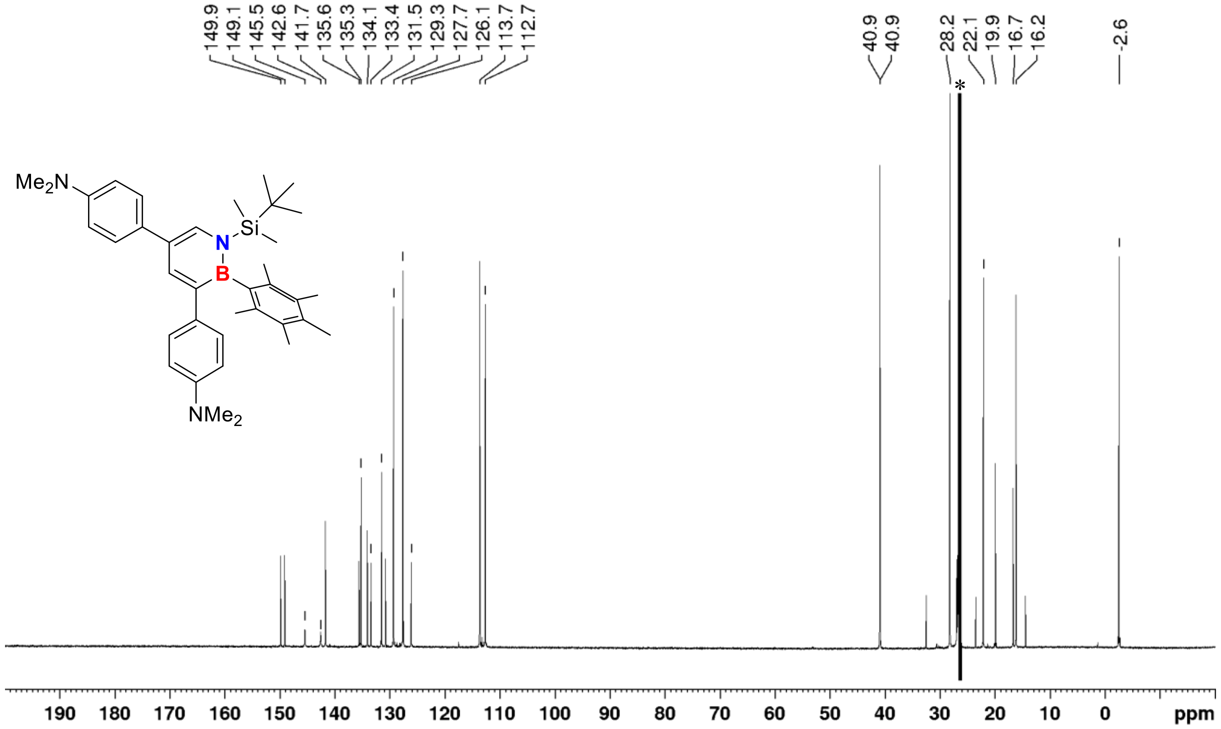

Figure S34. ^{13}C -{^{1}H}-NMR spectrum of compound ^{BN}**B3**$_{\mathbf{NMe2}}$ in C_6D_{12} measured at a 700 MHz spectrometer. The solvent signal is marked with an asterisk.

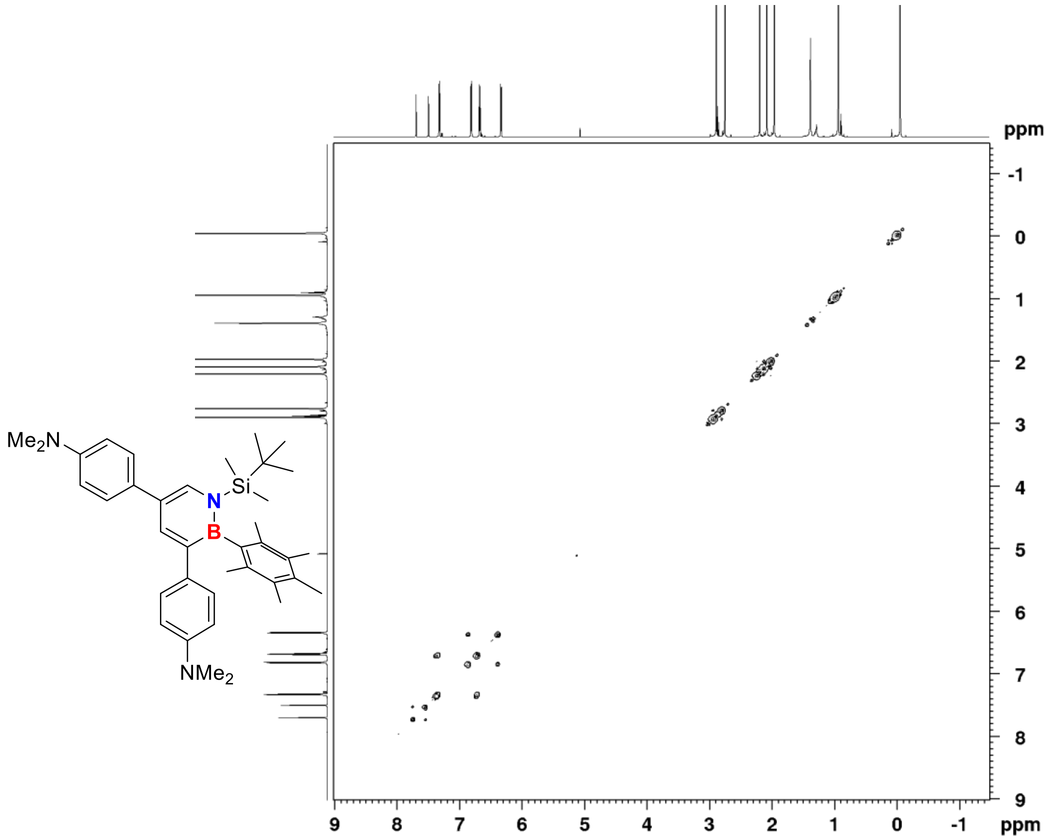

Figure S35. ^{1}H -^{1}H-CPSY-NMR spectrum of compound ^{BN}**B3**$_{\mathbf{NMe2}}$ in C_6D_{12} measured at a 700 MHz spectrometer.

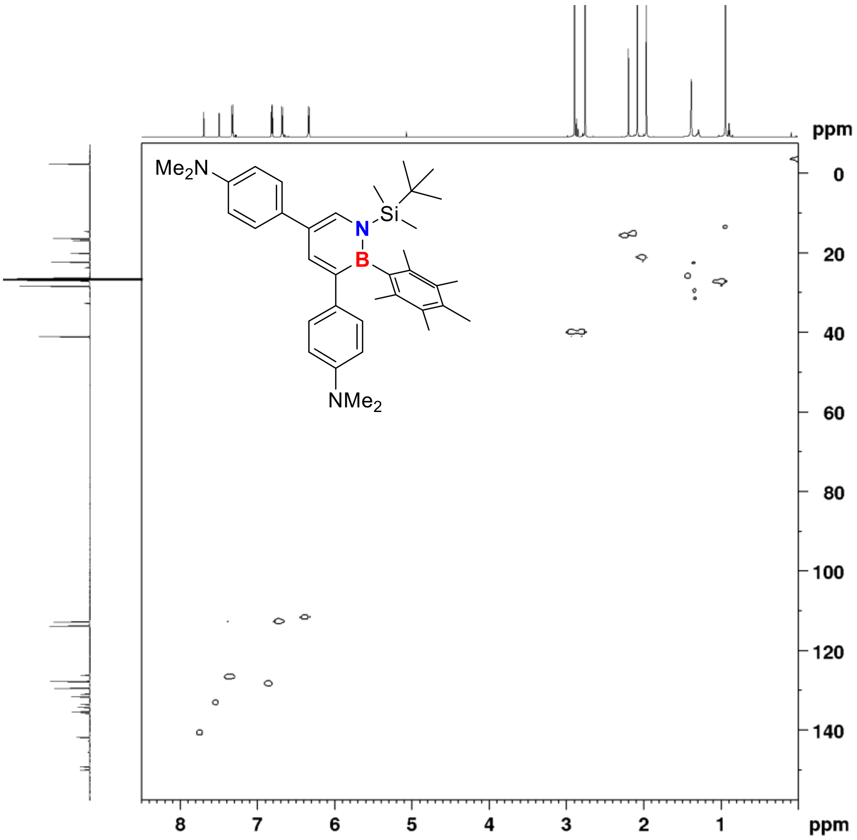

Figure S36. ^{1}H -^{13}C-HSQC-NMR spectrum of compound ^{BN}**B3**$_{\mathbf{NMe2}}$ in C_6D_{12} measured at a 700 MHz spectrometer.

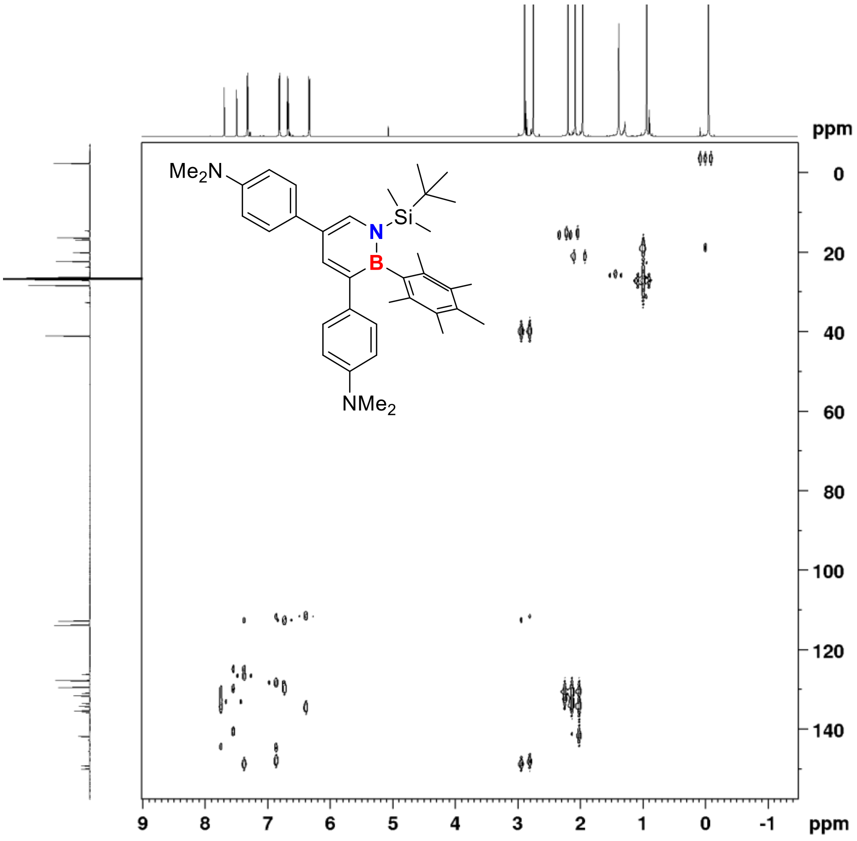

Figure S37. ^{1}H -^{13}C-HMBC-NMR spectrum of compound ^{BN}**B3**$_{\mathbf{NMe2}}$ in C_6D_{12} measured at a 700 MHz spectrometer.

*NMR data of ^{BN}**B3**$_{CF3}$*

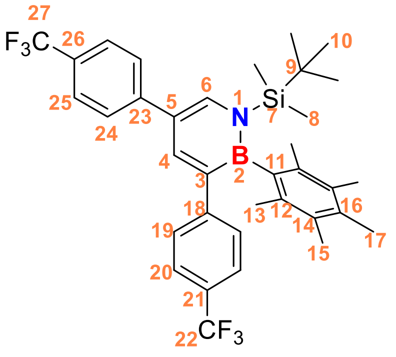

^{BN}**B3**$_{CF3}$

$C_{35}H_{40}BF_6NSi$ (627.60 g/mol)

^{1}H-NMR (400 MHz, **C_6D_{12}**): δ = 7.78 (s, 2H, H-4/H-6), 7.58 (m, 4H, H-24/H-25), 7.23 (d, $^3J_{HH}$ = 8.25 Hz, 2H, H-20), 7.02 (d, $^3J_{HH}$ = 8.25 Hz, 2H, H-19), 2.20 (s 3H, H-17), 2.08 (s, 6H, H-15), 1.95 (s, 6H, H-13), 0.96 (s, 9H, H-10), 0.01 (s, 6H, H-8) ppm.

^{13}C-{^{1}H}-NMR (100 MHz, **C_6D_{12}**): δ = 149.6, 145.1, 144.7, 142.3, 140.6, 138.3, 134.8, 134.7, 132.2, 159.9, 127.0, 126.3, 124.7, 28.0, 22.2, 19.9, 16.7, 16.1, -2.7 ppm.

^{11}B-{^{1}H}-NMR (128 MHz, **C_6D_{12}**): δ = 40.5 ppm.

HR-MS (ESI): m/z calc. For [M+Na]+ 650.28195, found 650.28244.

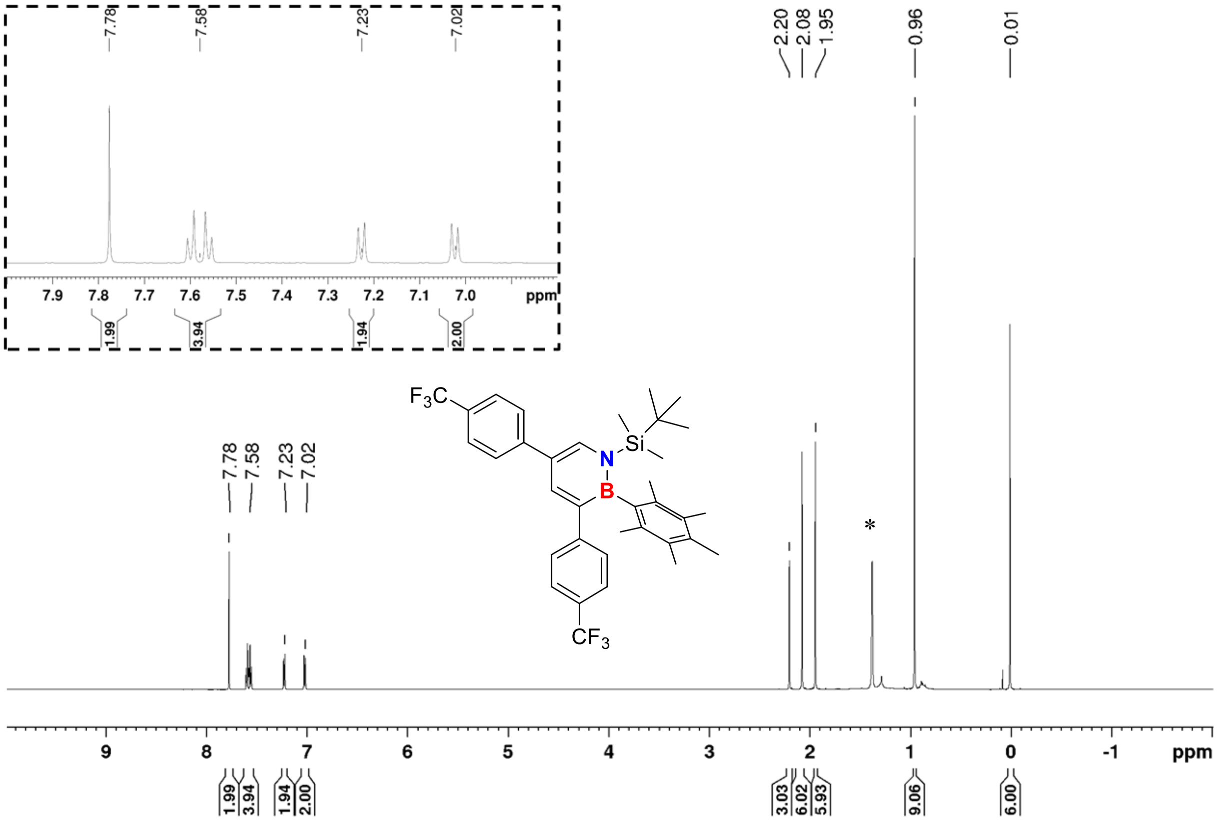

Figure S38. ^{1}H-NMR spectrum of compound ^{BN}**B3**$_{CF3}$ in C_6D_{12} measured at a 600 MHz spectrometer. The solvent signal is marked with an asterisk.

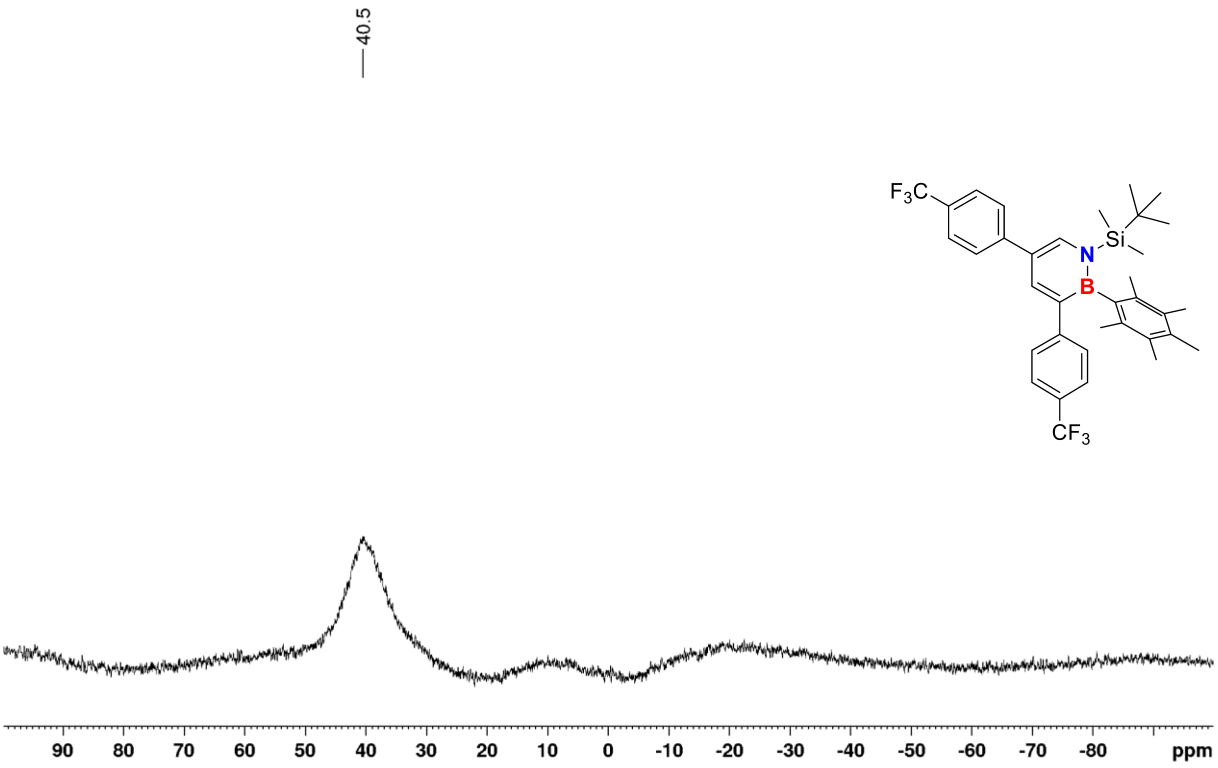

Figure S39. ^{11}B-{^{1}H}-NMR spectrum of compound ^{BN}**B3**$_{CF3}$ in C_6D_{12} measured at a 600 MHz spectrometer.

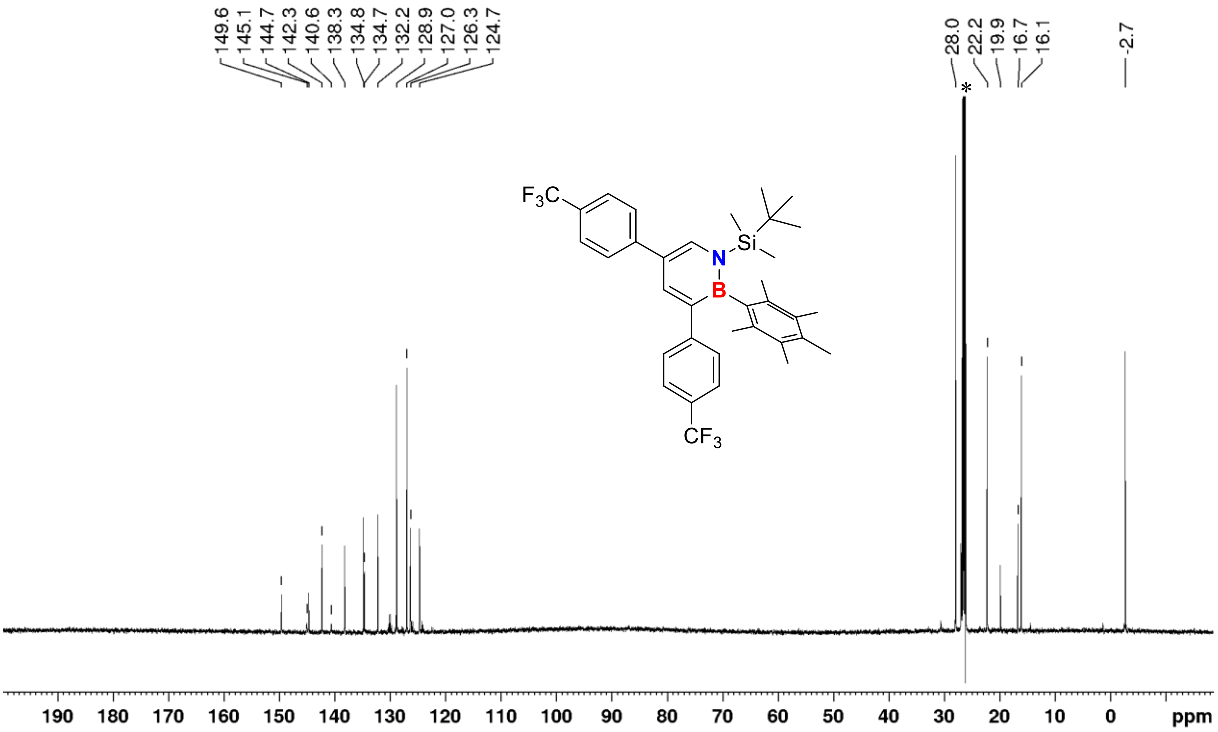

Figure S40. $^{13}C-\{^{1}H\}$-NMR spectrum of compound ^{BN}**B3**$_{CF3}$ in C_6D_{12} measured at a 600 MHz spectrometer. The solvent signal is marked with an asterisk.

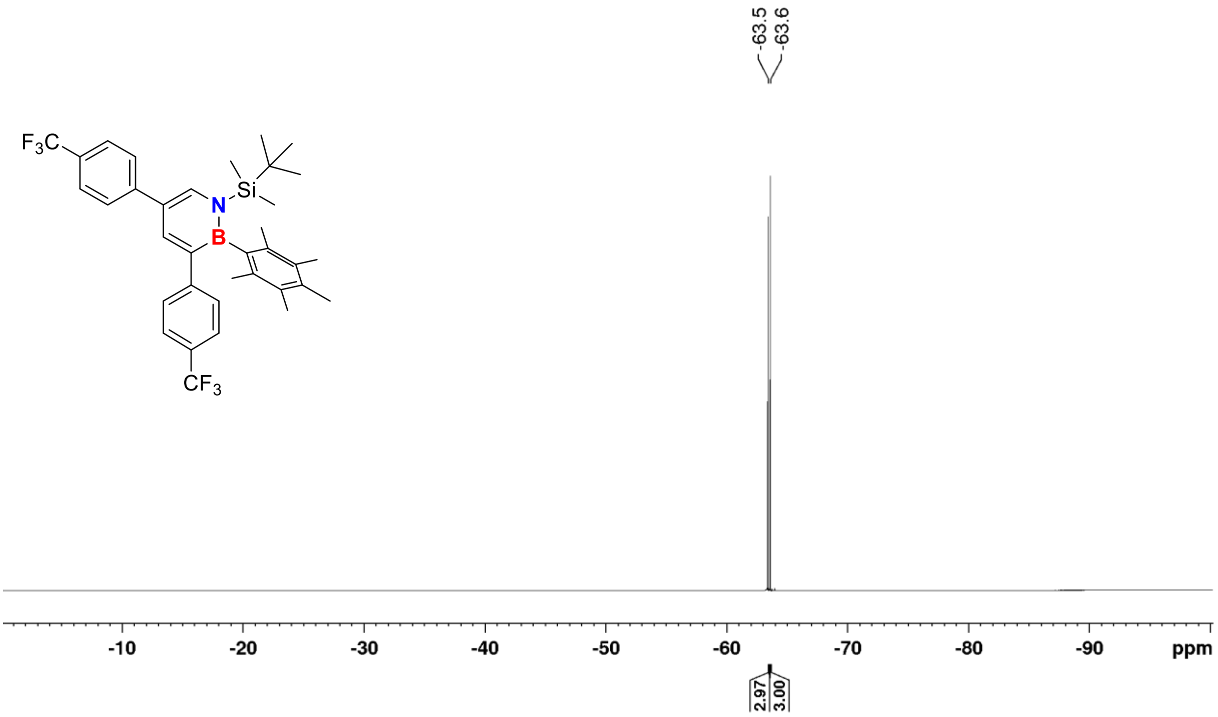

Figure S41. $^{19}F-\{^{1}H\}$-NMR spectrum of compound ^{BN}**B3**$_{CF3}$ in C_6D_{12} measured at a 600 MHz spectrometer.

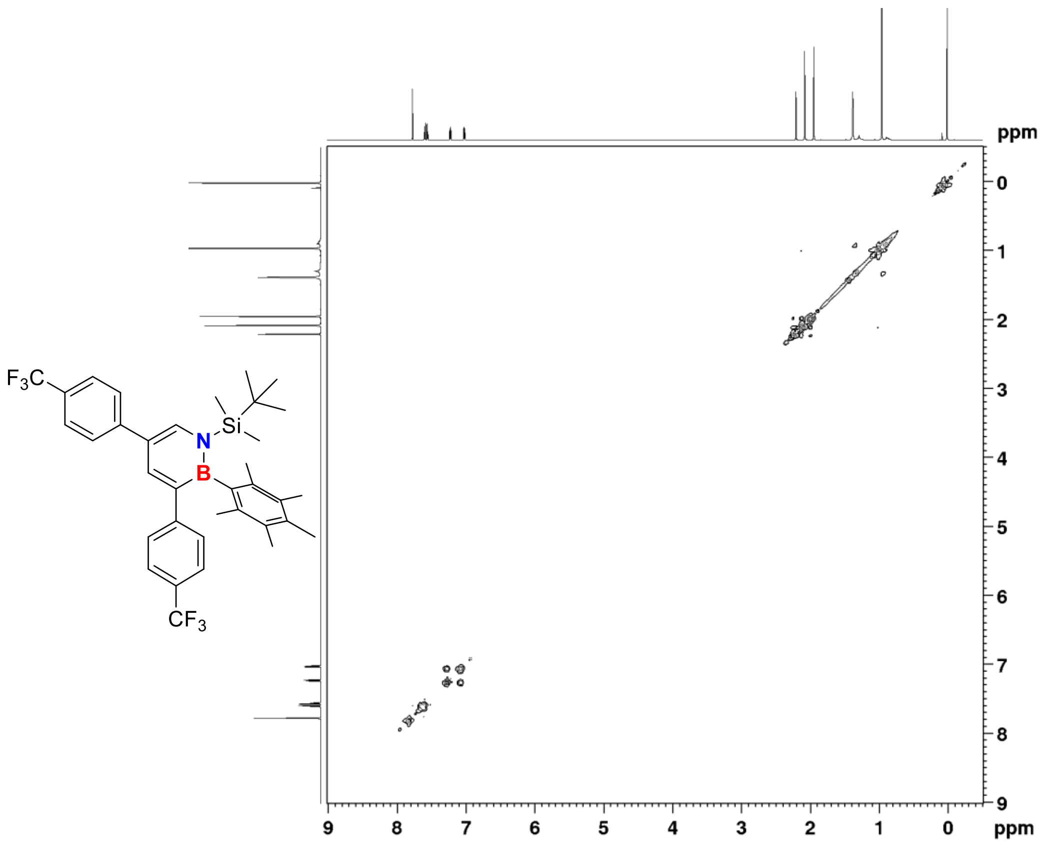

Figure S42. ^{1}H-^{1}H-COSY-NMR spectrum of compound ^{BN}**B3**$_{CF3}$ in C_6D_{12} measured at a 600 MHz spectrometer.

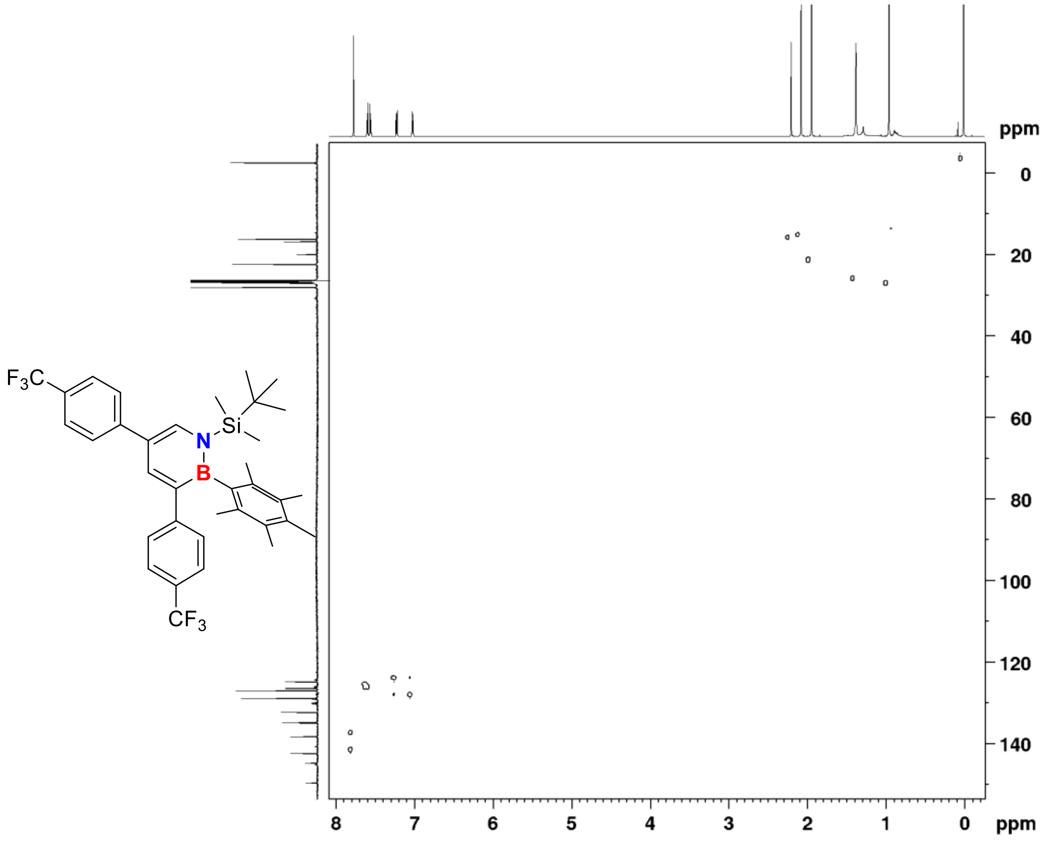

Figure S43. ^{1}H-^{13}C-HSQC-NMR spectrum of compound ^{BN}**B3**$_{CF3}$ in C_6D_{12} measured at a 600 MHz spectrometer.

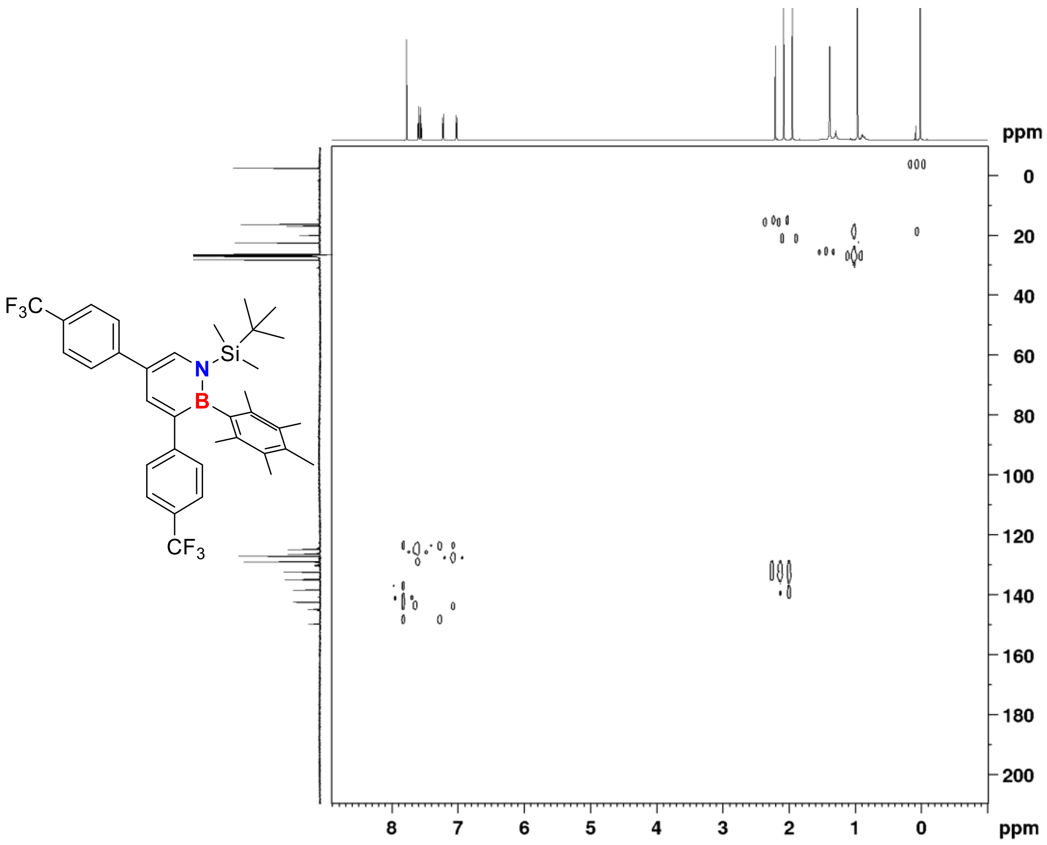

Figure S44. ^{1}H -^{13}C-HMBC-NMR spectrum of compound ^{BN}**B3**$_{CF3}$ in C_6D_{12} measured at a 600 MHz spectrometer.

*NMR data of ^{BN}**B3**$_{Br}$*

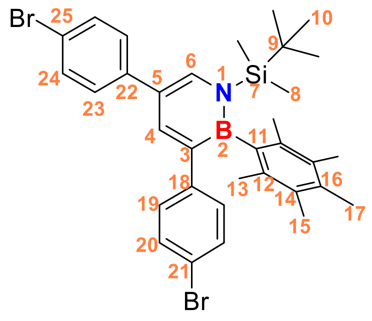

^{BN}**B3**$_{Br}$

$C_{33}H_{40}BBr_2NSi$ (649.39 g/mol)

^{1}H-NMR (400 MHz, **C_6D_{12}**): δ = 7.84 (m, 2H, H-4/H-6), 7.48 (d, $^3J_{HH}$ = 8.43 Hz, 2H, H-24), 7.30 (d, $^3J_{HH}$ = 8.43 Hz, 2H, H-23), 7.27 (m, 2H, H-20), 7.03 (d, $^3J_{HH}$ = 8.60 Hz, 2H, H-19), 2.19 (s, 3H, H-17), 2.17 (s, 6H, H-15), 2.14 (s, 6H, H-13), 0.93 (s, 9H, H-10), 0.02 (s, 6H, H-8) ppm.

^{13}C-{^{1}H}-NMR (100 MHz, **C_6D_{12}**): δ = 144.9, 142.0, 139.7, 137.0, 134.6, 134.4, 132.4, 131.9, 131.1, 130.3, 124.3, 121.0, 120.3, 27.6, 22.2, 19.3, 16.7, 16.1, -2.6 ppm.

^{11}B-{^{1}H}-NMR (128 MHz, **C_6D_{12}**): δ = 36.1 ppm.

The ionization and HR-MS detection were not successful with the ESI ion source available in the institute.

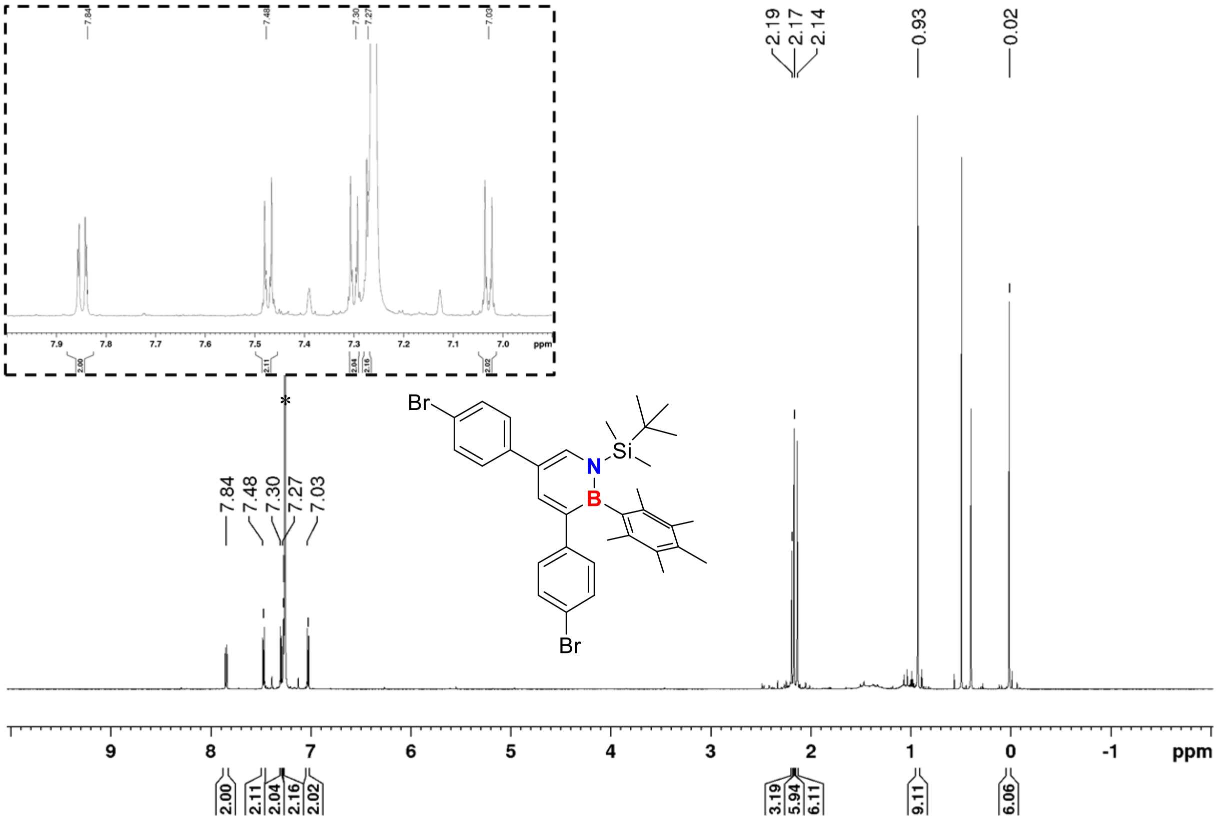

Figure S45. ^{1}H-NMR spectrum of compound ^{BN}**B3**$_{\mathbf{Br}}$ in C_6D_6 measured at a 600 MHz spectrometer. The solvent signal is marked with an asterisk.

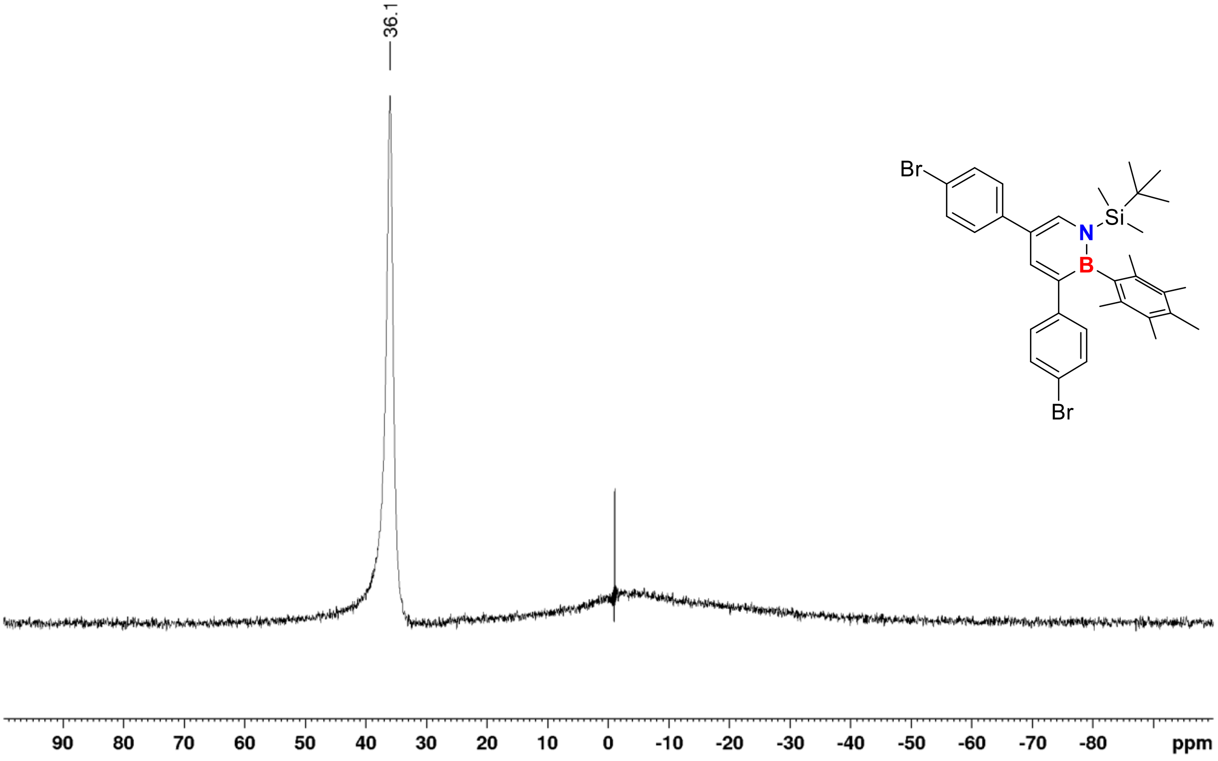

Figure S46. ^{11}B-{^{1}H}-NMR spectrum of compound ^{BN}**B3**$_{\mathbf{Br}}$ in C_6D_6 measured at a 600 MHz spectrometer.

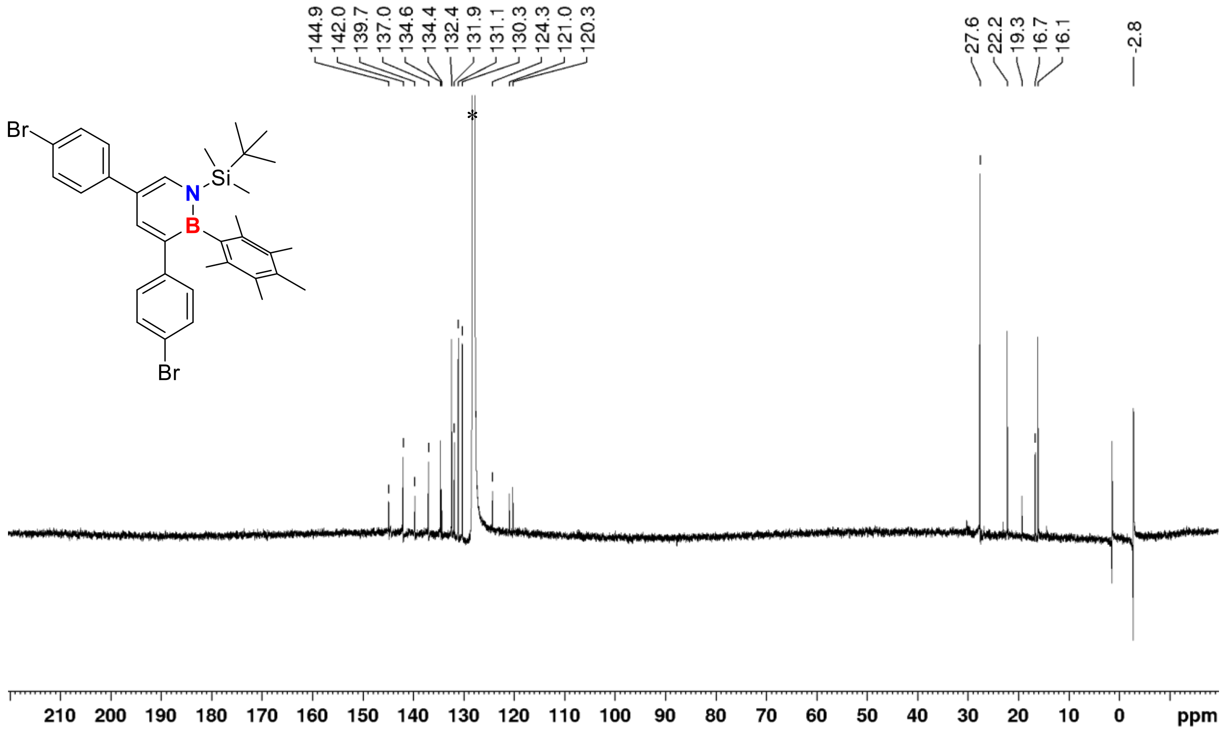

Figure S47. ^{13}C-{^{1}H}-NMR spectrum of compound ^{BN}**B3**$_{\mathbf{Br}}$ in C_6D_6 measured at a 600 MHz spectrometer. The solvent signal is marked with an asterisk.

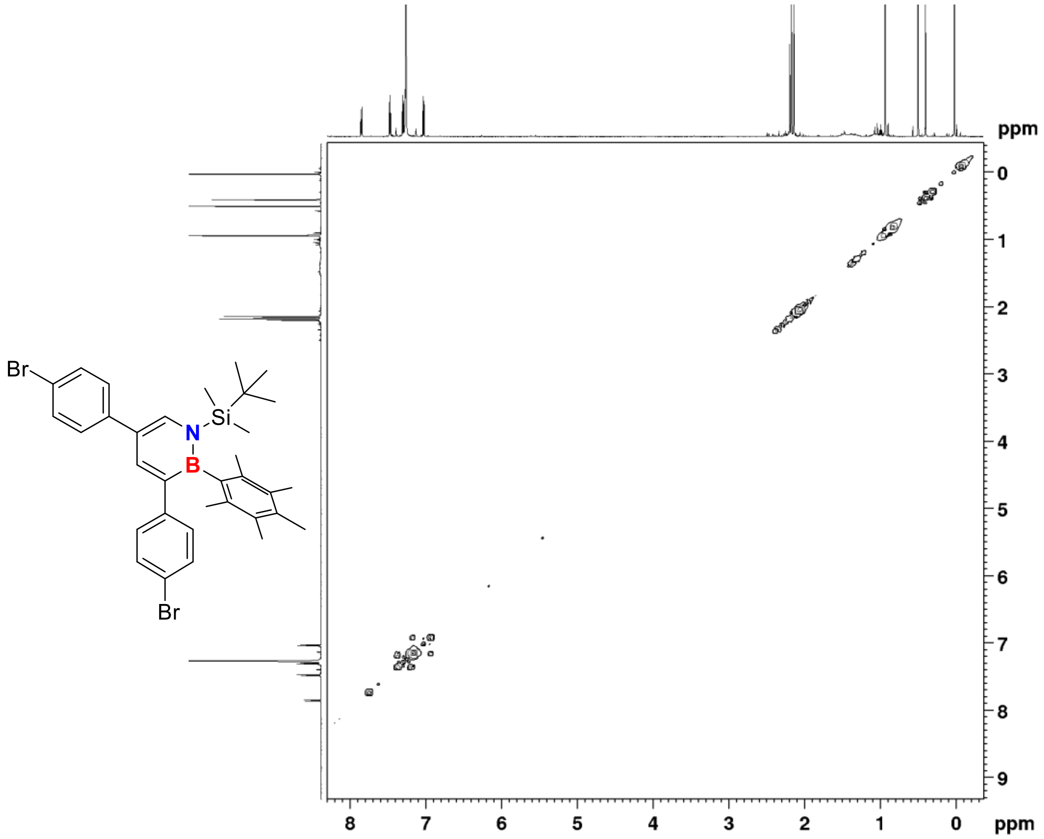

Figure S48. ^{1}H-^{1}H-HSQC-NMR spectrum of compound ^{BN}**B3**$_{\mathbf{Br}}$ in C_6D_6 measured at a 600 MHz spectrometer.

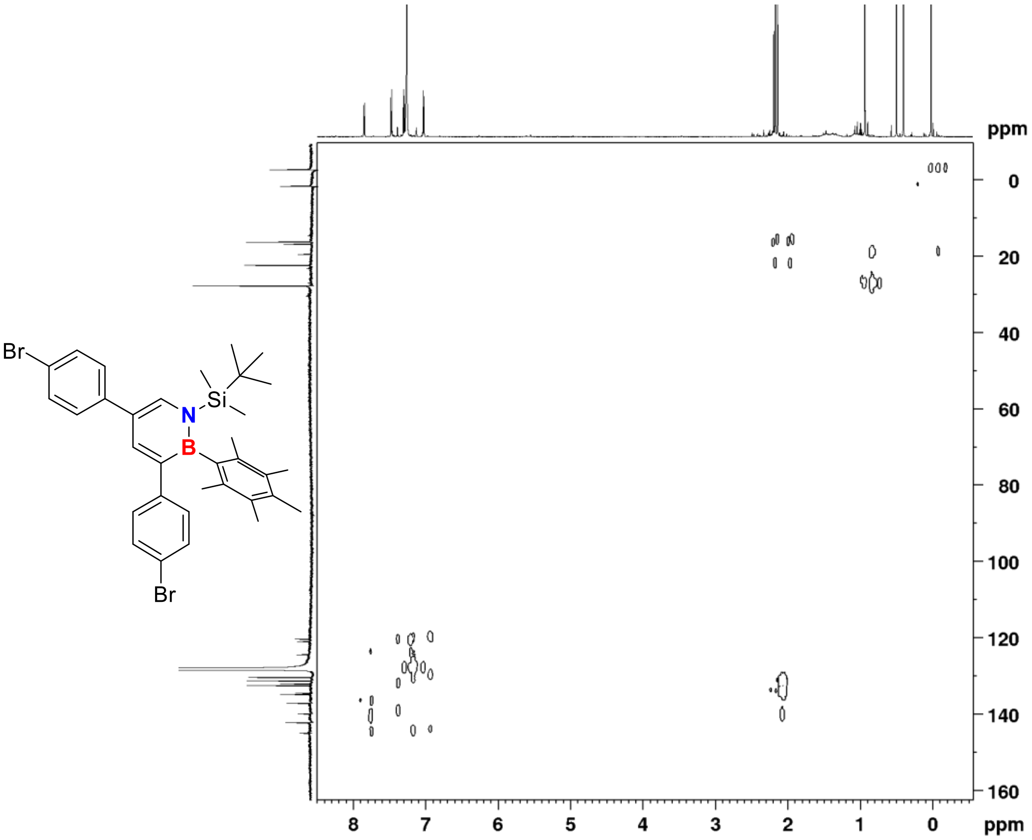

Figure S49. ^{1}H -^{13}C-HSQC-NMR spectrum of compound ^{BN}**B3**$_{\mathbf{Br}}$ in C_6D_6 measured at a 600 MHz spectrometer.

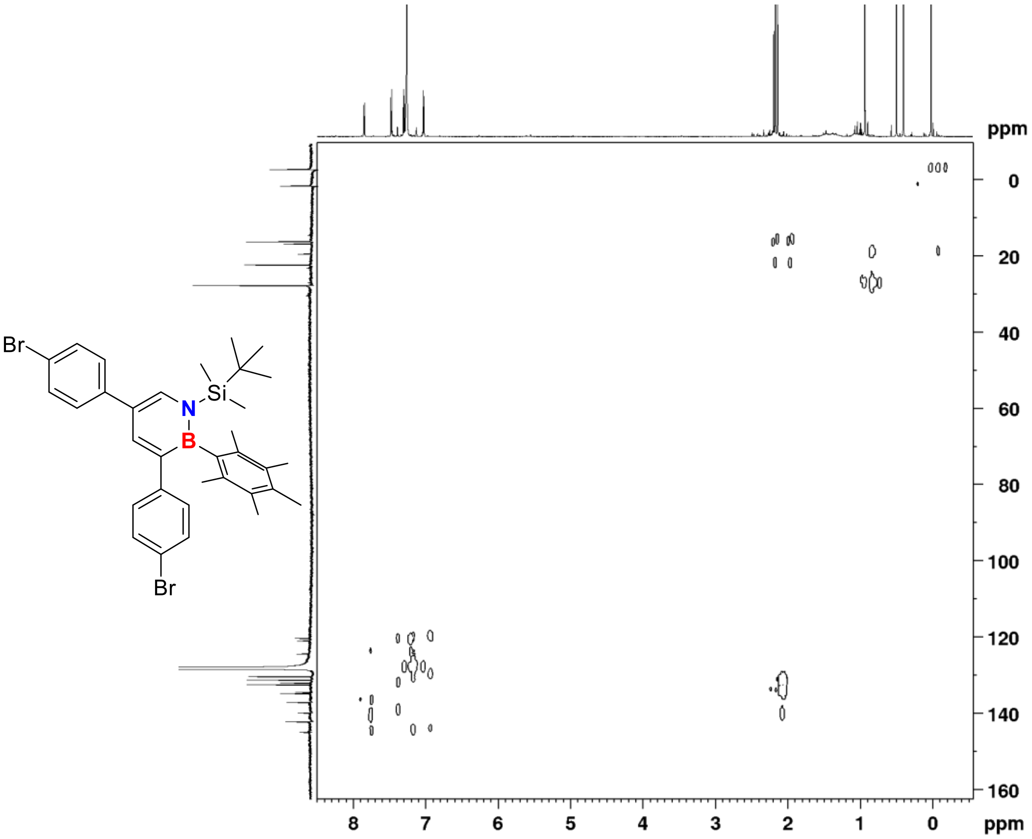

Figure S50. ^{13}H -^{13}C-HMBC-NMR spectrum of compound ^{BN}**B3**$_{\mathbf{Br}}$ in C_6D_6 measured at a 600 MHz spectrometer.

4. Irradiation experiments

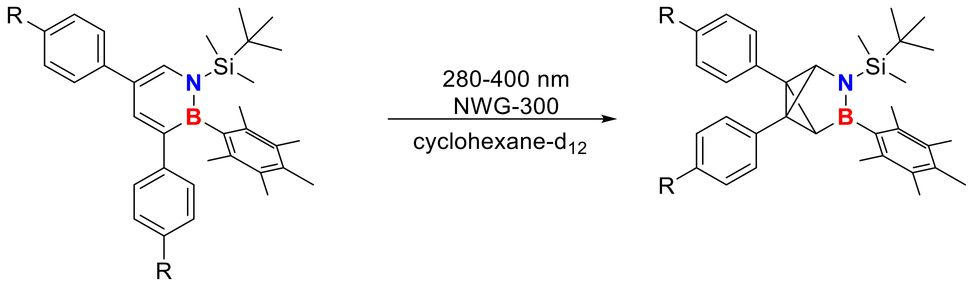

*BN*B3 *BN*V3

All irradiations were carried out using a dichroitic mirror that selects the wavelengthrange 280-400 nm, with an additional NWG-300 filter from *Schott*. The samples were placed in quartz J. Young NMR tubes and solved in deuterated cyclohexane under argon before the irradiation. Upon irradiation the sample was cooled with compressed air and a fan to prevent thermal cycloreversion. The exact irradiation time was dependent on the concentrations of the solution. The irradiation time to reach above 95% conversion to ***BN*V3** in 0.05 M solutions was 10 minutes for ***BN*V3H**. The irradiation yielded the compounds ***BN*B3** in almost quantitative yield.

^{BN}V3 scope

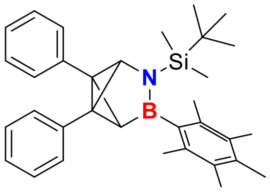

^{BN}V3$_{H}$

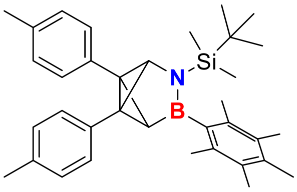

^{BN}V3$_{Me}$

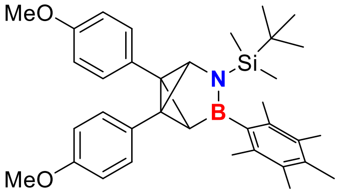

^{BN}V3$_{OMe}$

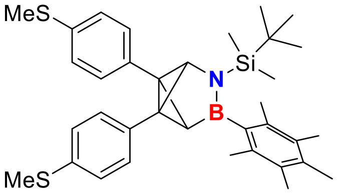

^{BN}V3$_{SMe}$

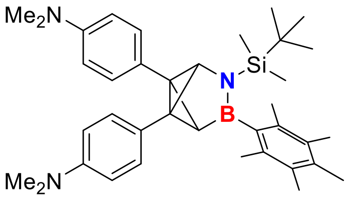

^{BN}V3$_{NMe2}$

NMR data of ^{BN}***V3***$_H$

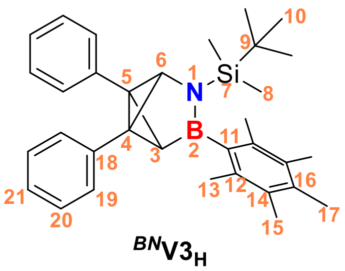

^{BN}V3$_H$

$C_{33}H_{42}BNSi$ (491.60 g/mol)

^{1}H-NMR (400 MHz, **C_6D_{12}**): δ = 7.14 (m, 8H, H-19/H-20), 7.04 (m, 2H, H-21), 4.35 (d, $^4J_{HH}$ = 6.23 Hz, 1H, H-6), 2.25 (d, $^4J_{HH}$ = 6.23 Hz, 1H, H-3), 2.22 (s, 3H, H-17), 2.16 (s, 12 H, H-13/H-15), 0.99 (s, 9H, H-10), -0.07 (s, 6H, H-8) ppm.

^{1}H-NMR (400 MHz, **C_8D_{10}**): δ = 7.22 (m, 4H, H-19), 7.12 (m, 4H, H-20), 6.99 (m, 2H, H-21), 4.41 (d, $^4J_{HH}$ = 6.28 Hz, 1H, H-6), 2.31 (d, $^4J_{HH}$ = 6.28 Hz, 1H, H-4), 2.28 (s, 6H, H-13), 2.18 (s, 9H, H-15/H-17), 1.02 (s, 9H, H-10), 0.01 (s, 6H, H-8) ppm.

^{13}C-{^{1}H}-NMR (100 MHz, **C_8D_{10}**): δ = 134.5, 133.8, 132.8, 131.1, 128.6, 128.3, 128.3, 128.1, 125.9, 125.7, 57.4, 55.8, 39.4, 26.7, 21.4, 15.9, 15.4, -5.2 ppm.

^{11}B-{^{1}H}-NMR (128 MHz, **C_8D_{10}**): δ = 53.2 ppm.

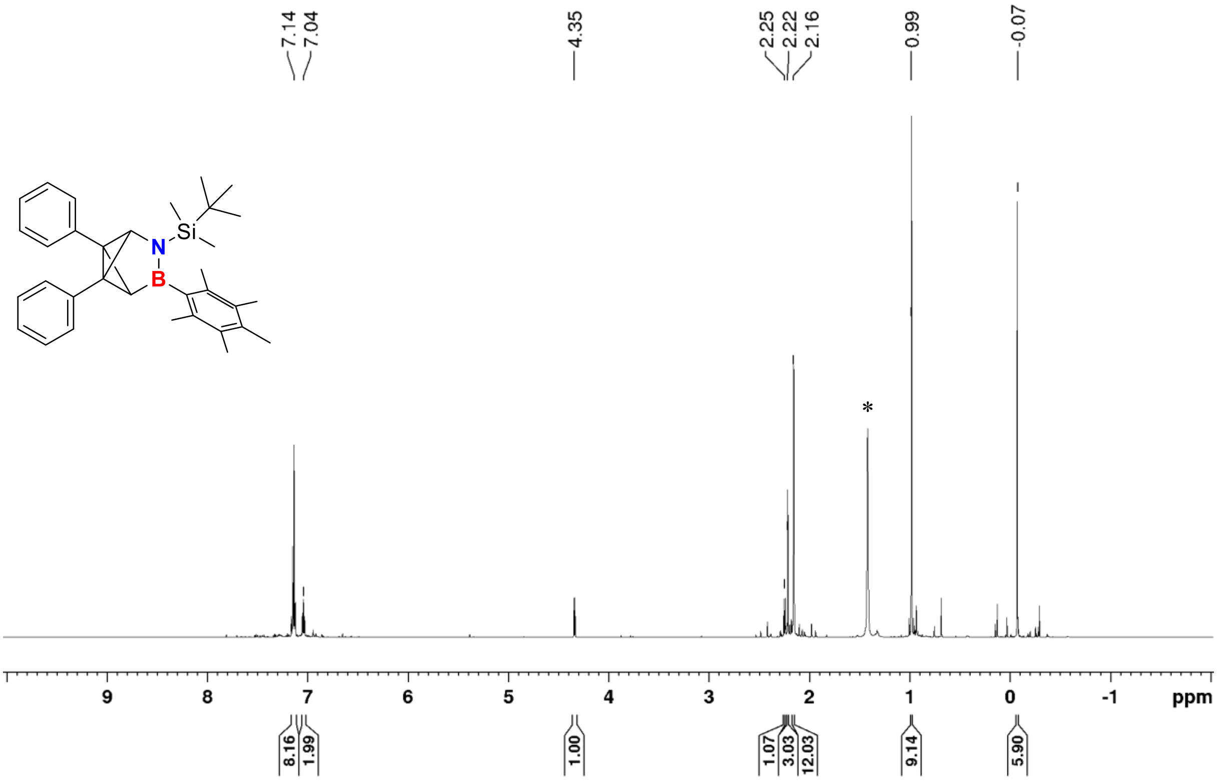

Figure S51. ^{1}H-NMR spectrum of compound ^{BN}**V3$_H$** in C_6D_{12} measured at a 600 MHz spectrometer. The solvent signal is marked with an asterisk.

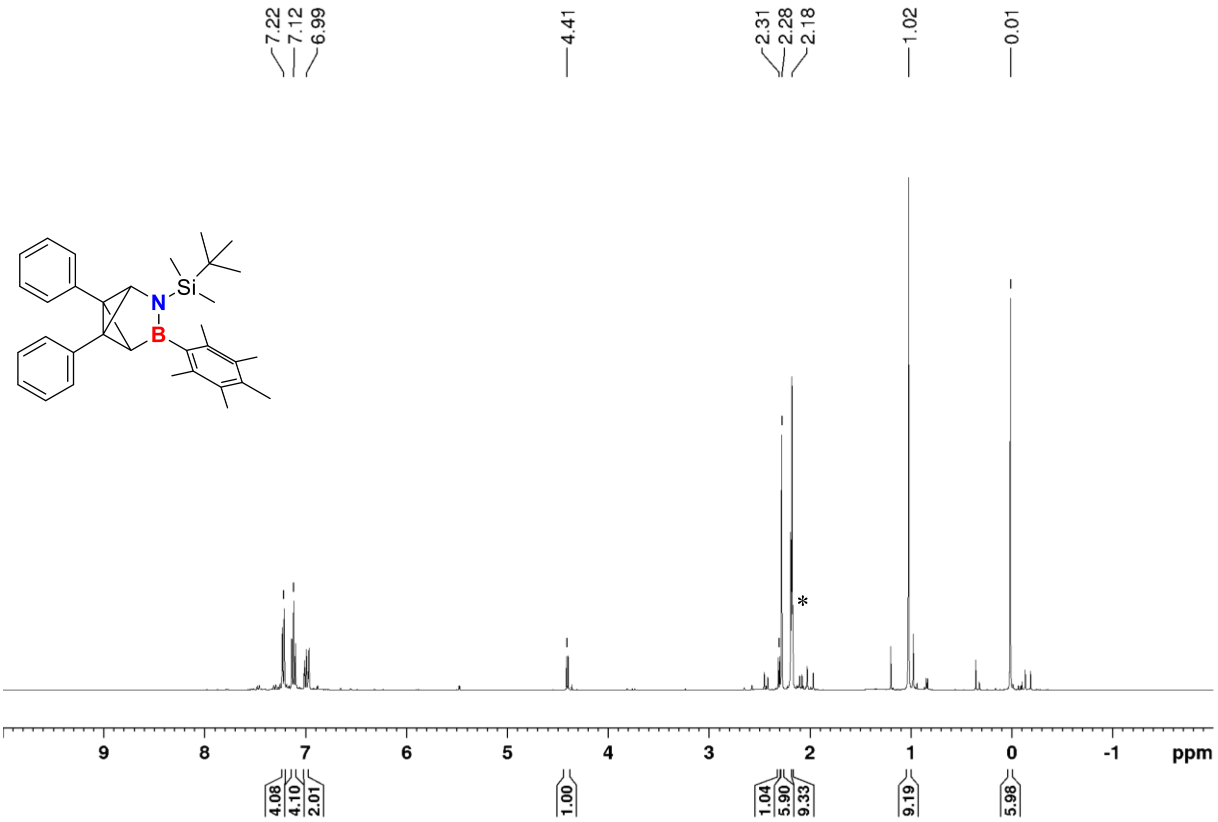

Figure S52. ^{1}H-NMR spectrum of compound ^{BN}**V3$_H$** in C_9D_{12} measured at a 600 MHz spectrometer. The solvent signal is marked with an asterisk.

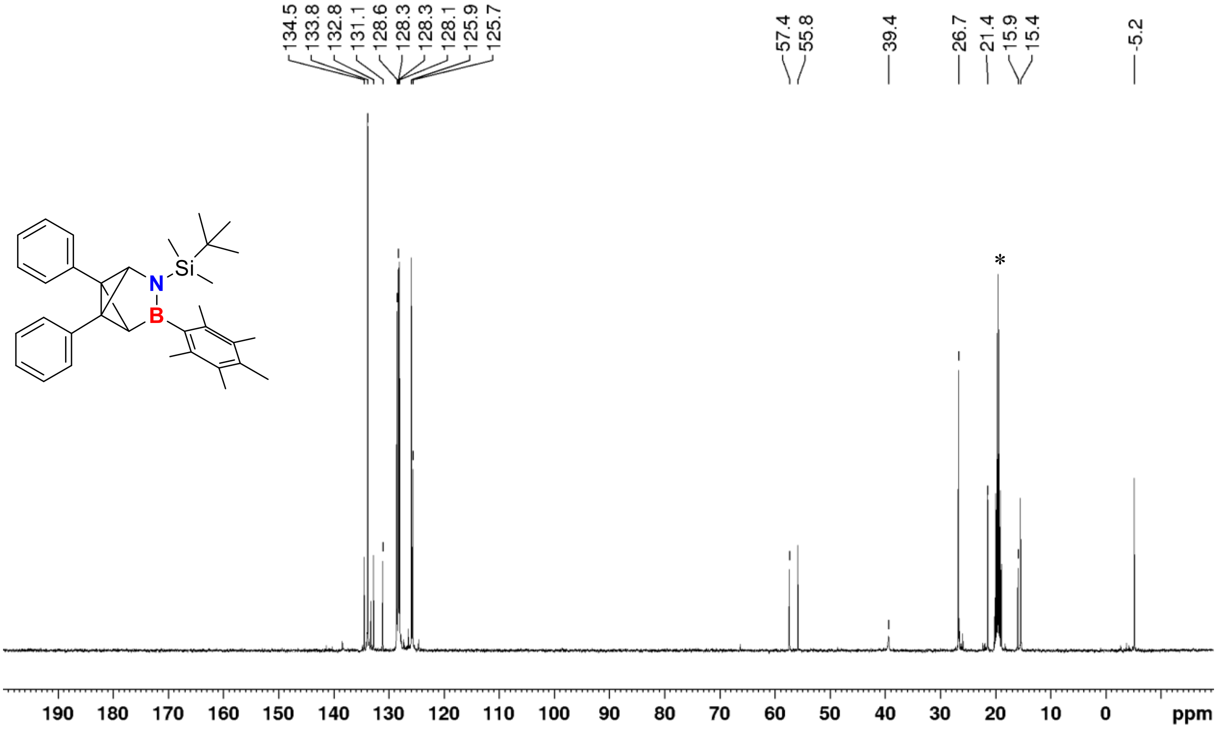

Figure S53. ^{13}C-{^{1}H}-NMR spectrum of compound ^{BN}**V3$_{H}$** in C_6D_{12} measured at a 600 MHz spectrometer. The solvent signal is marked with an asterisk.

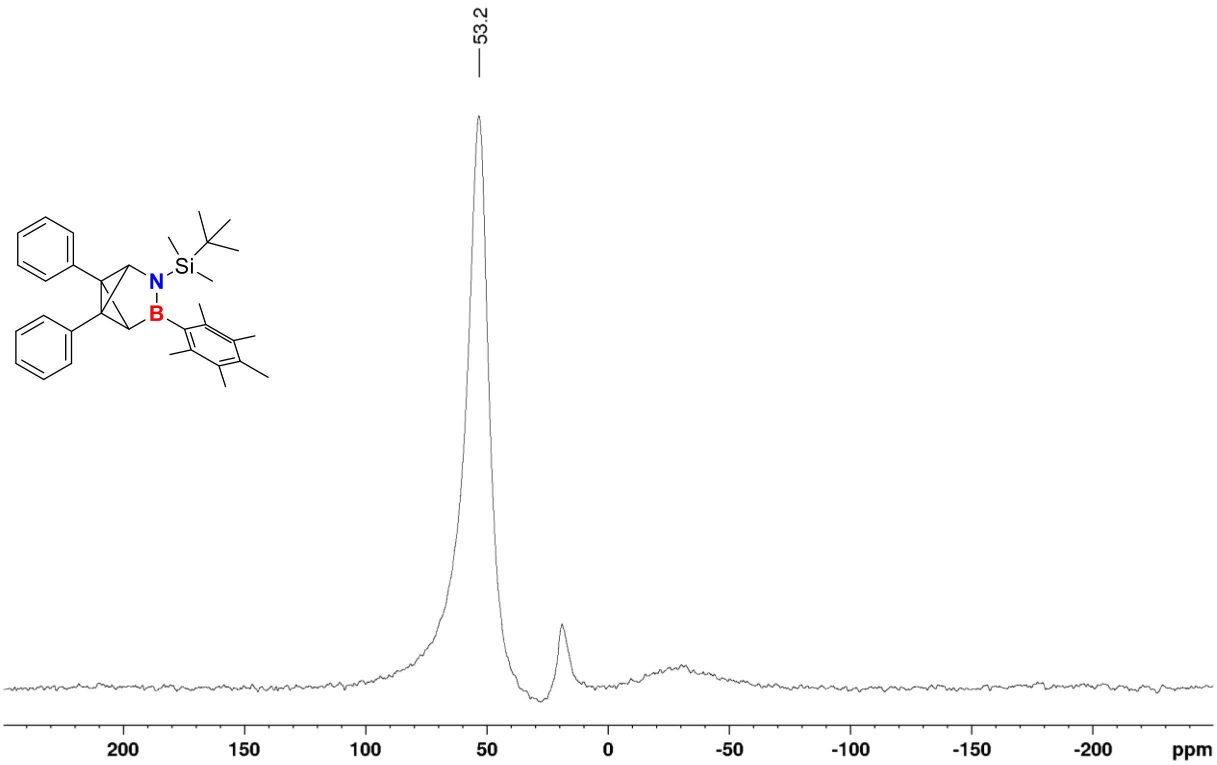

Figure S54. ^{11}B-{^{1}H}-NMR spectrum of compound ^{BN}**V3$_{H}$** in C_9D_{12} measured at a 600 MHz spectrometer.

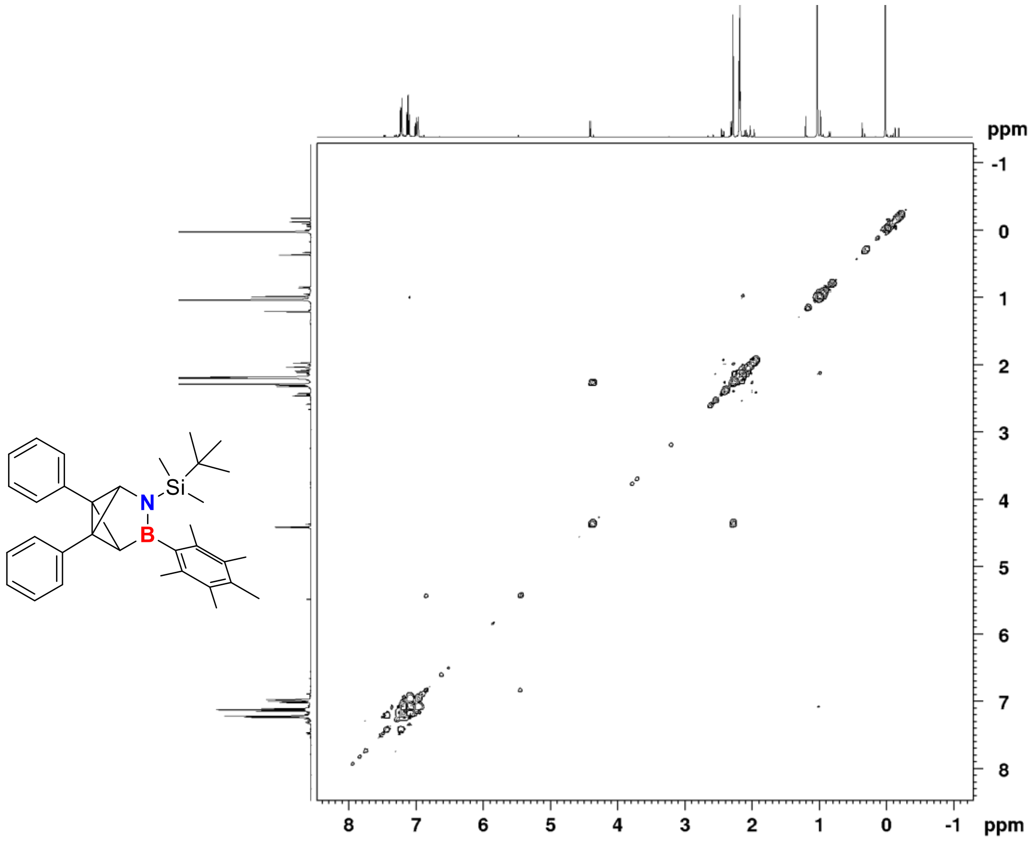

Figure S55. ^{1}H -^{1}H-COSY-NMR spectrum of compound ^{BN}**V3**$_{\mathbf{H}}$ in C_9D_{12} measured at a 600 MHz spectrometer.

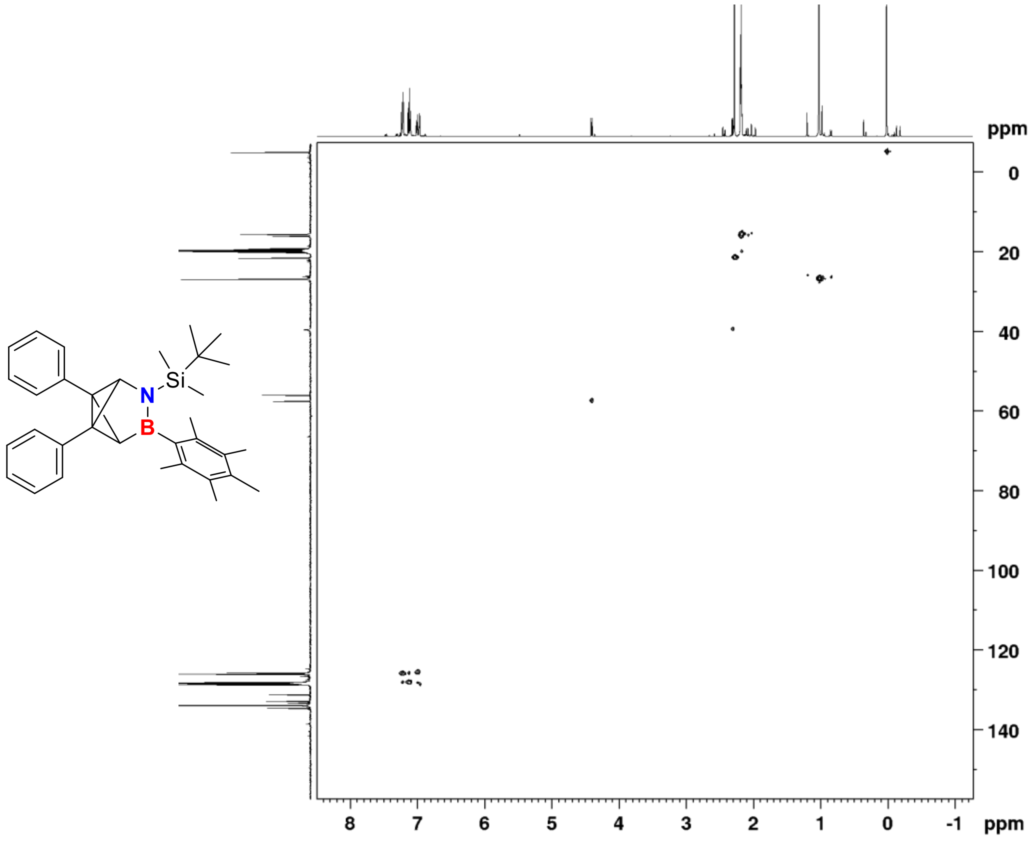

Figure S56. ^{1}H -^{13}C-HSQC-NMR spectrum of compound ^{BN}**V3**$_{\mathbf{H}}$ in C_9D_{12} measured at a 600 MHz spectrometer.

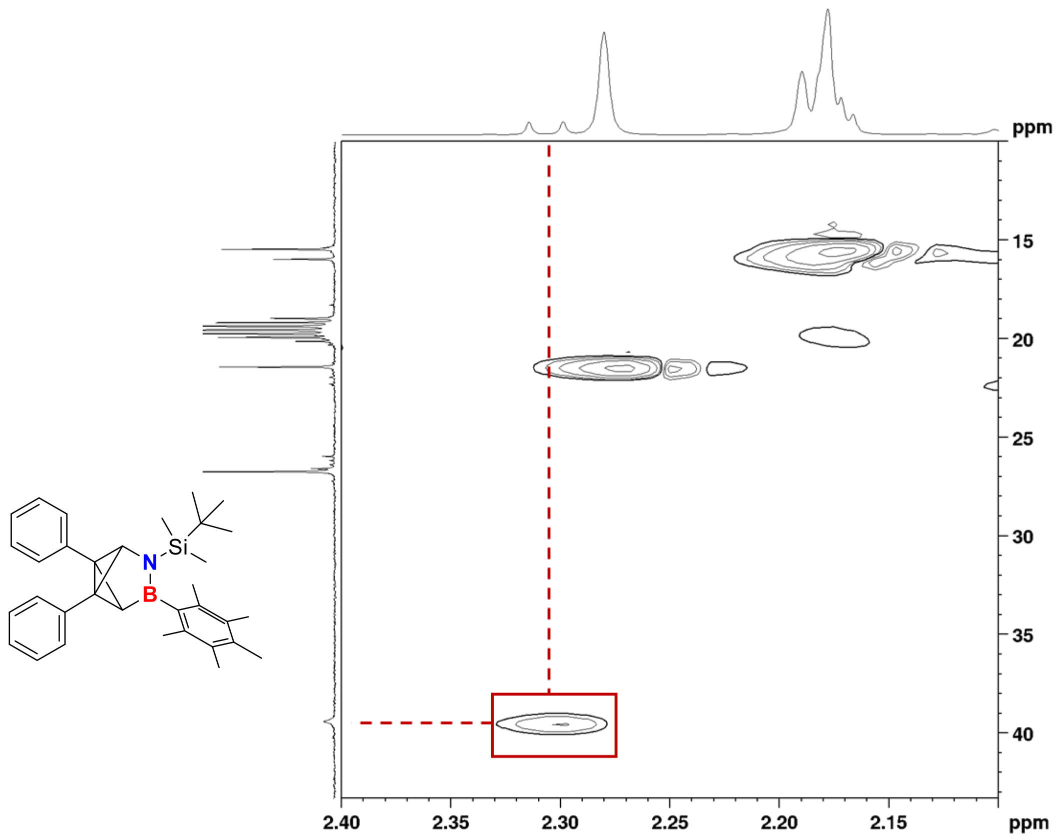

Figure S57. ^{1}H-^{13}C-HSQC-NMR spectrum of compound ^{BN}**V3**$_{\mathbf{H}}$ in C_9D_{12} measured at a 600 MHz spectrometer. Enlarged view of the correlation between C3 and H-3.

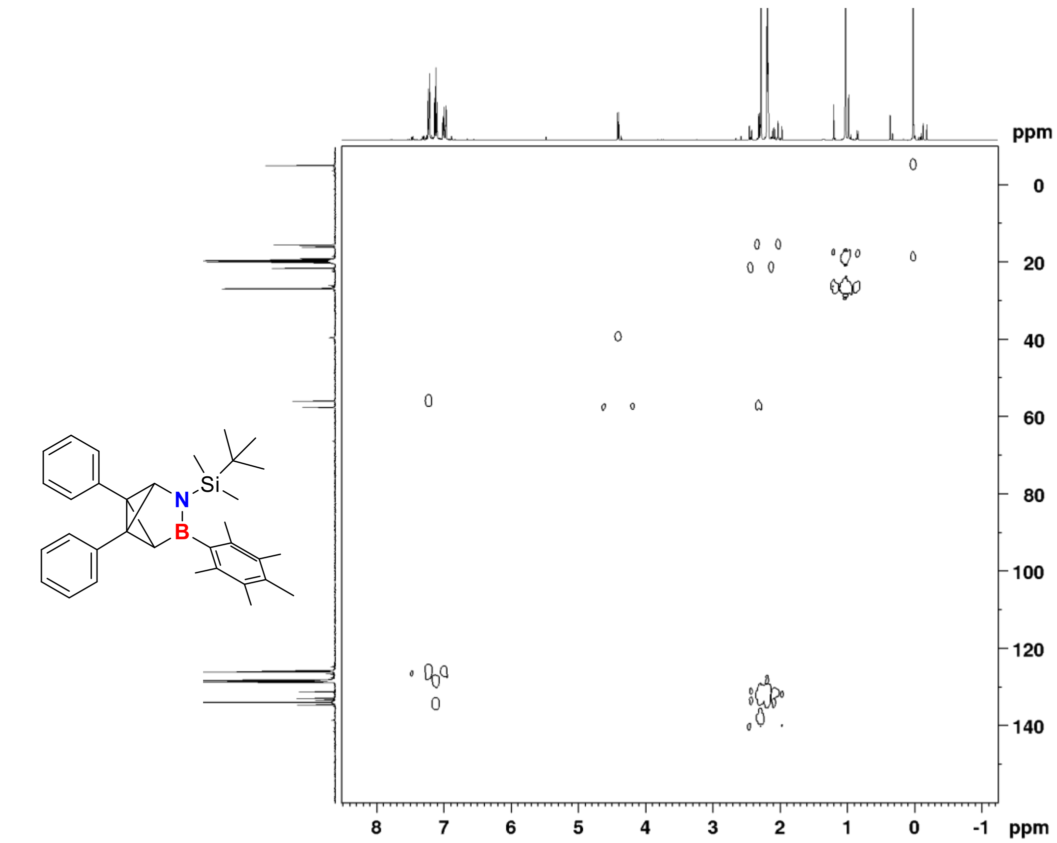

Figure S58. ^{1}H-^{13}C-HMBC-NMR spectrum of compound ^{BN}**V3**$_{\mathbf{H}}$ in C_9D_{12} measured at a 600 MHz spectrometer.

NMR data of ^{BN}V3$_{Me}$

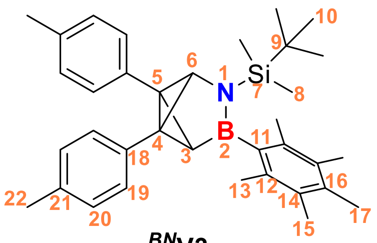

^{BN}**V3**$_{Me}$

$C_{35}H_{46}BNSi$ (519.66 g/mol)

^{1}H-NMR (400 MHz, **C_6D_{12}**): δ = 7.02 (d, $^{3}J_{HH}$ = 8.15 Hz, 4H, H-20), 6.96 (d, $^{3}J_{HH}$ = 8.15 Hz, 4H, H-19), 4.28 (d, $^{4}J_{HH}$ = 6.33 Hz, 1H, H-6), 2.25 (s, 6H, H-22/H-27), 2.21 (s, 3H, H-17), 2.17 (d, $^{4}J_{HH}$ = 6.33 Hz, 1H, H-4), 2.15 (s, 12H, H-13/H-15), 0.97 (s, 9H, H-10), -0.09 (s, 6H, H-8) ppm.

^{13}C-{^{1}H}-NMR (100 MHz, **C_6D_{12}**): δ = 128.4, 126.7, 126.5, 125.4, 124.7, 122.6, 119.7, 60.6, 51.1, 49.3, 32.7, 23.7, 20.6, 15.0, 14.4, 12.8, 9.1, -11.6 ppm.

^{11}B-{^{1}H}-NMR (128 MHz, **C_6D_{12}**): δ = 52.5 ppm.

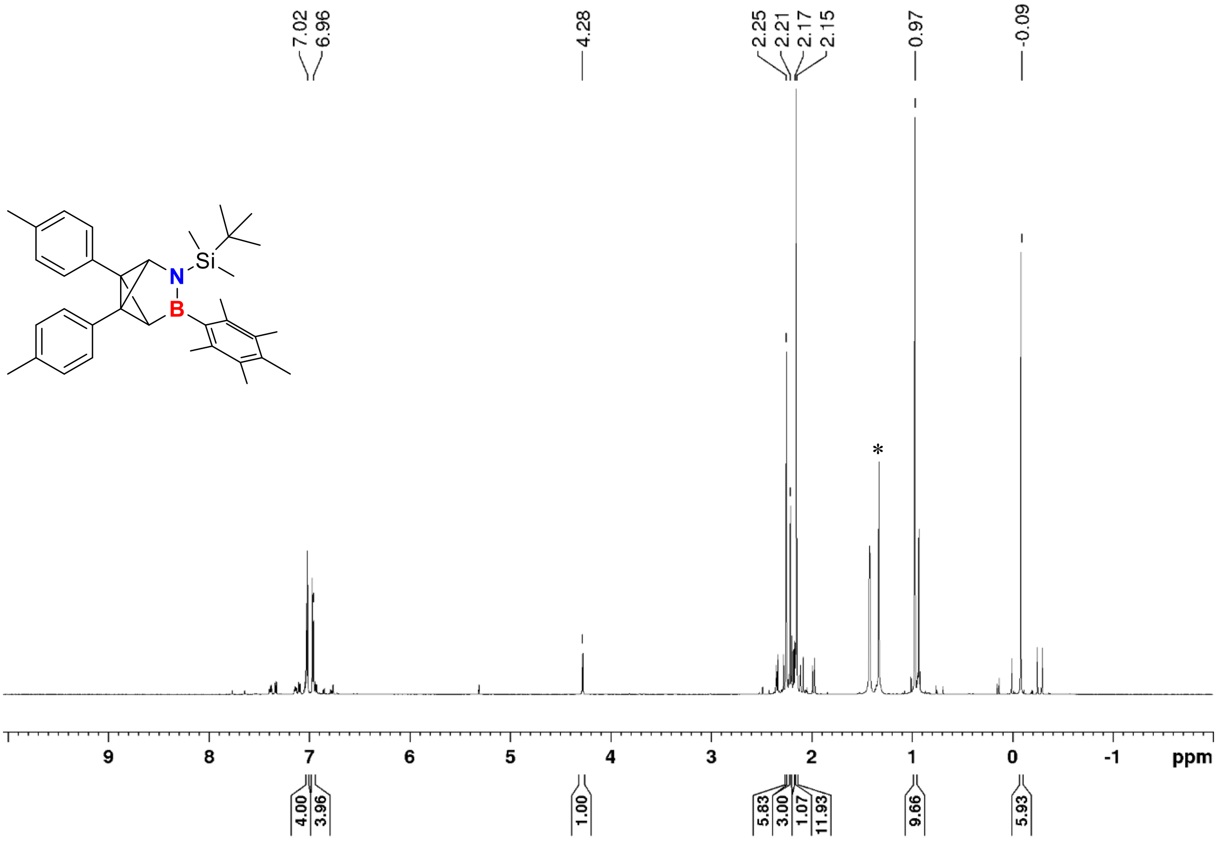

Figure S59. ^{1}H-NMR spectrum of compound ^{BN}**V3$_{Me}$** in C_6D_{12} measured at a 600 MHz spectrometer. The solvent signal is marked with an asterisk.

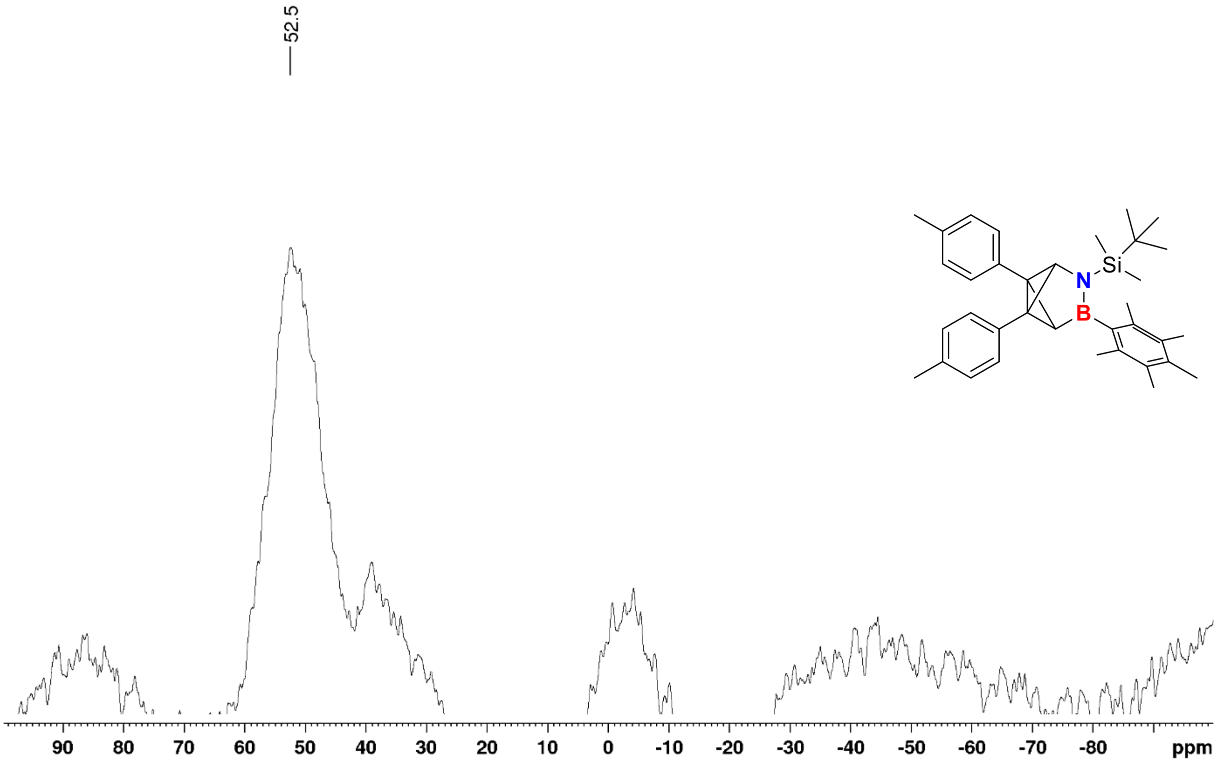

Figure S60. ^{11}B-{^{1}H}-NMR spectrum of compound ^{BN}**V3$_{Me}$** in C_6D_{12} measured at a 600 MHz spectrometer.

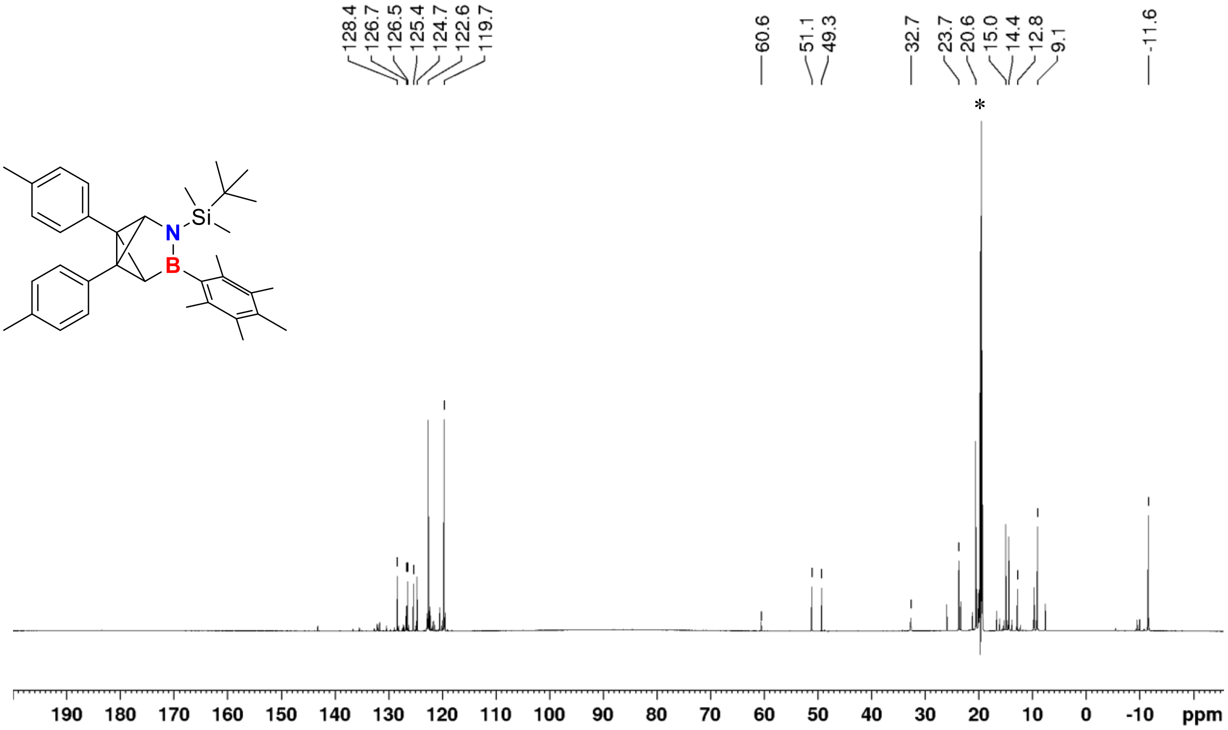

Figure S61. ^{13}C-{^{1}H}-NMR spectrum of compound ^{BN}**V3$_{Me}$** in C_6D_{12} measured at a 600 MHz spectrometer. The solvent signal is marked with an asterisk.

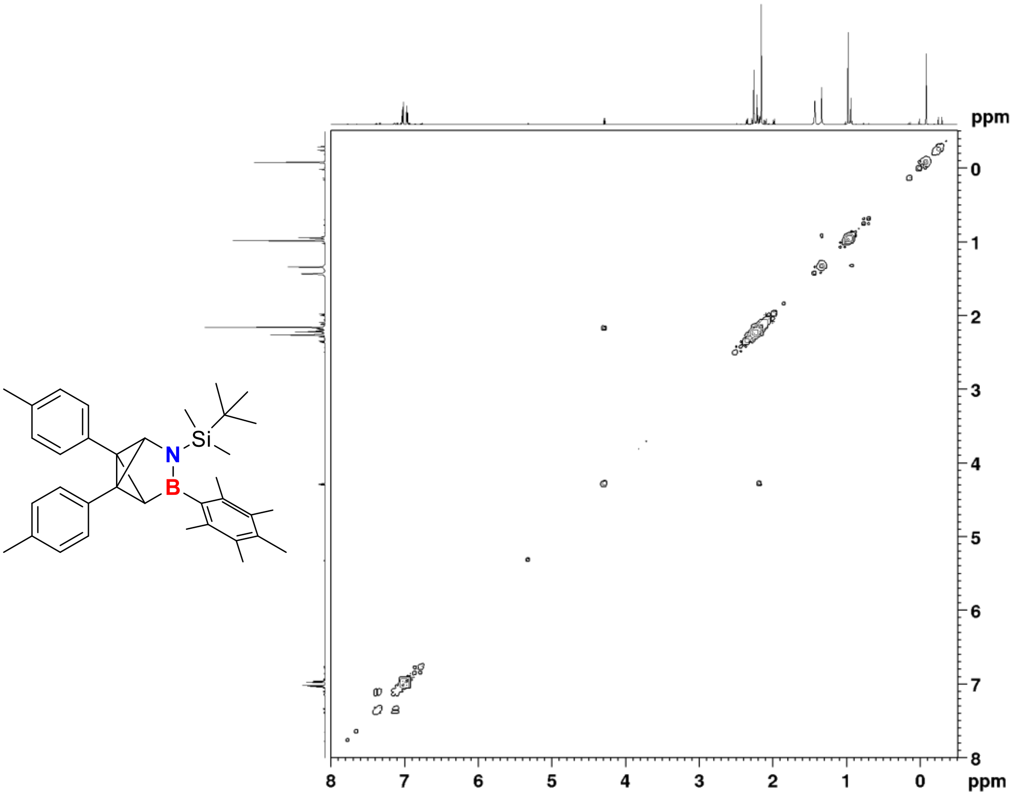

Figure S62. ^{1}H-^{1}H-COSY-NMR spectrum of compound ^{BN}**V3$_{Me}$** in C_6D_{12} measured at a 600 MHz spectrometer.

*NMR data of ^BN^V3_OMe_*

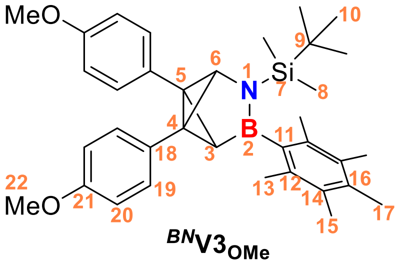

$C_{35}H_{46}BO_2NSi$ (551.65 g/mol)

^{1}H-NMR (400 MHz, **C_6D_{12}**): δ = 7.09 (d, $^3J_{HH}$ = 8.30 Hz, 4H, H-20), 7.04 (d, $^3J_{HH}$ = 8.30 Hz, 4H, H-19), 4.29 (d, $^4J_{HH}$ = 6.25 Hz, 1H, H-6), 2.35 (s, 6H, H-22), 2.22 (s, 3H, H-17), 2.20 (d, $^4J_{HH}$ = 6.25 Hz, 1H, H-4), 2.15 (s, 12H, H-15/H-13), 0.98 (s, 9H, H-10), -0.08 (s, 6H, H-8) ppm.

^{13}C-{^{1}H}-NMR (100 MHz, **C_6D_{12}**): δ = 138.8, 137.0, 133.8, 133.4, 132.1, 131.7, 128.0, 126.9, 58.2, 56.3, 39.8, 32.9, 30.2, 27.4, 21.9, 19.7, 16.6, 16.0, -4.7 ppm.

^{11}B-{^{1}H}-NMR (128 MHz, **C_6D_{12}**): δ = 51.6 ppm.

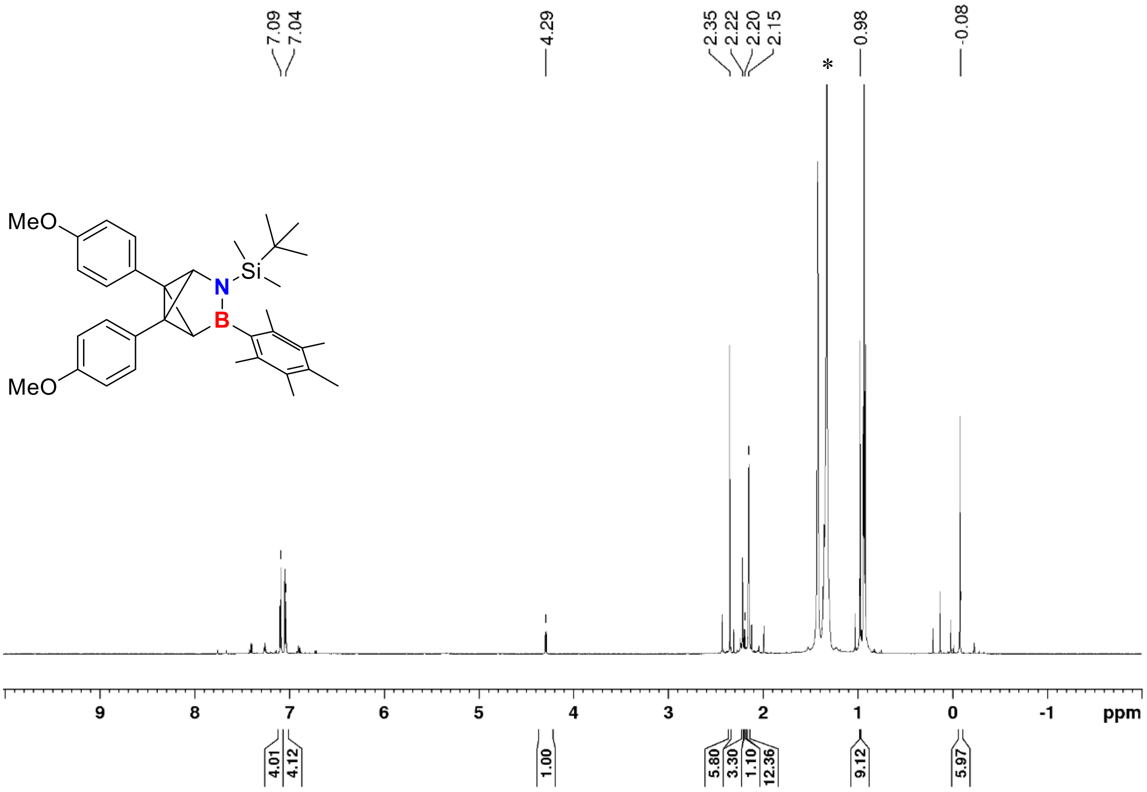

Figure S63. ^{1}H-NMR spectrum of compound ^{BN}**V3$_{OMe}$** in C_6D_{12} measured at a 600 MHz spectrometer. The solvent signal is marked with an asterisk.

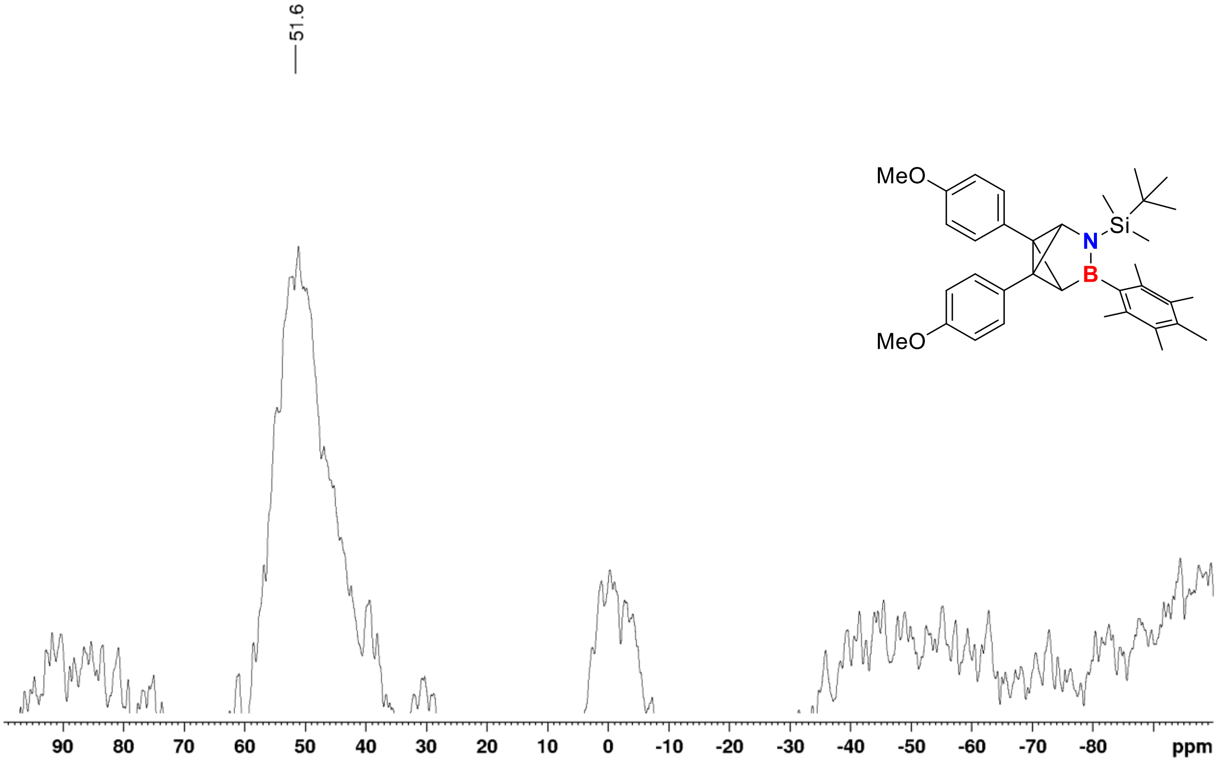

Figure S64. ^{11}B-{^{1}H}-NMR spectrum of compound ^{BN}**V3$_{OMe}$** in C_6D_{12} measured at a 600 MHz spectrometer.

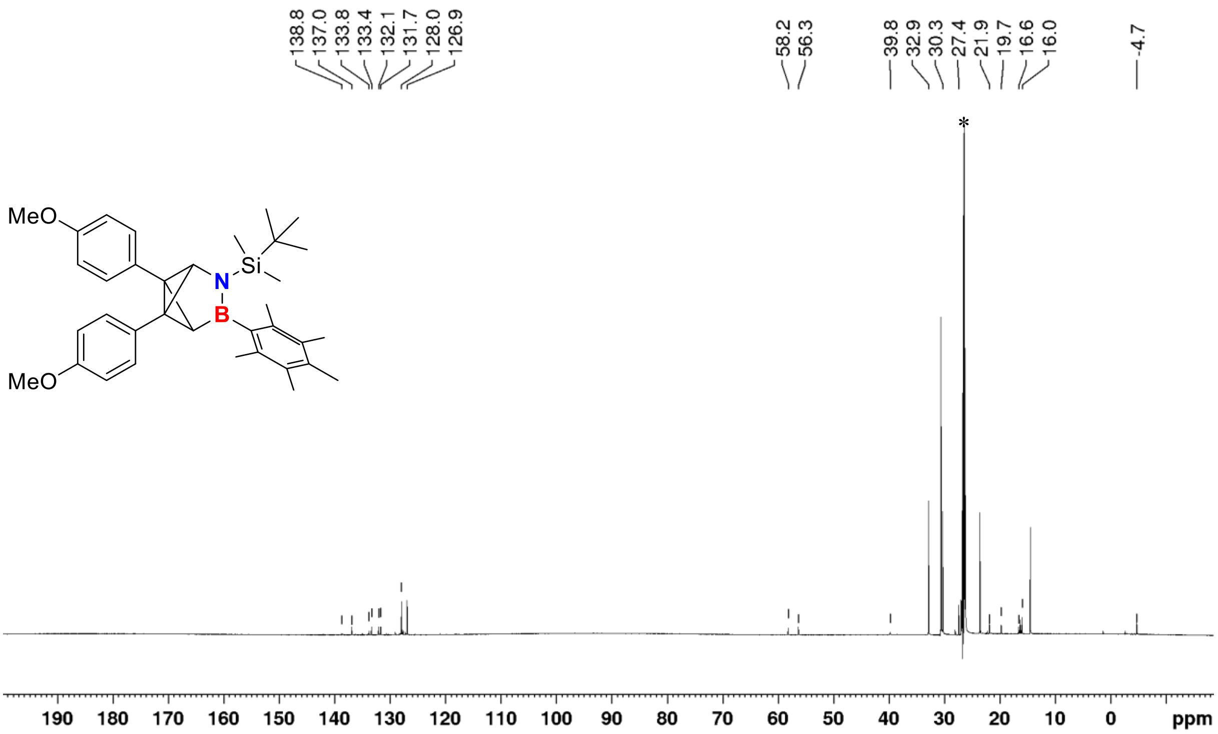

Figure S65. ^{13}C -{^{1}H}-NMR spectrum of compound ^{BN}**V3$_{OMe}$** in C_6D_{12} measured at a 600 MHz spectrometer. The solvent signal is marked with an asterisk.

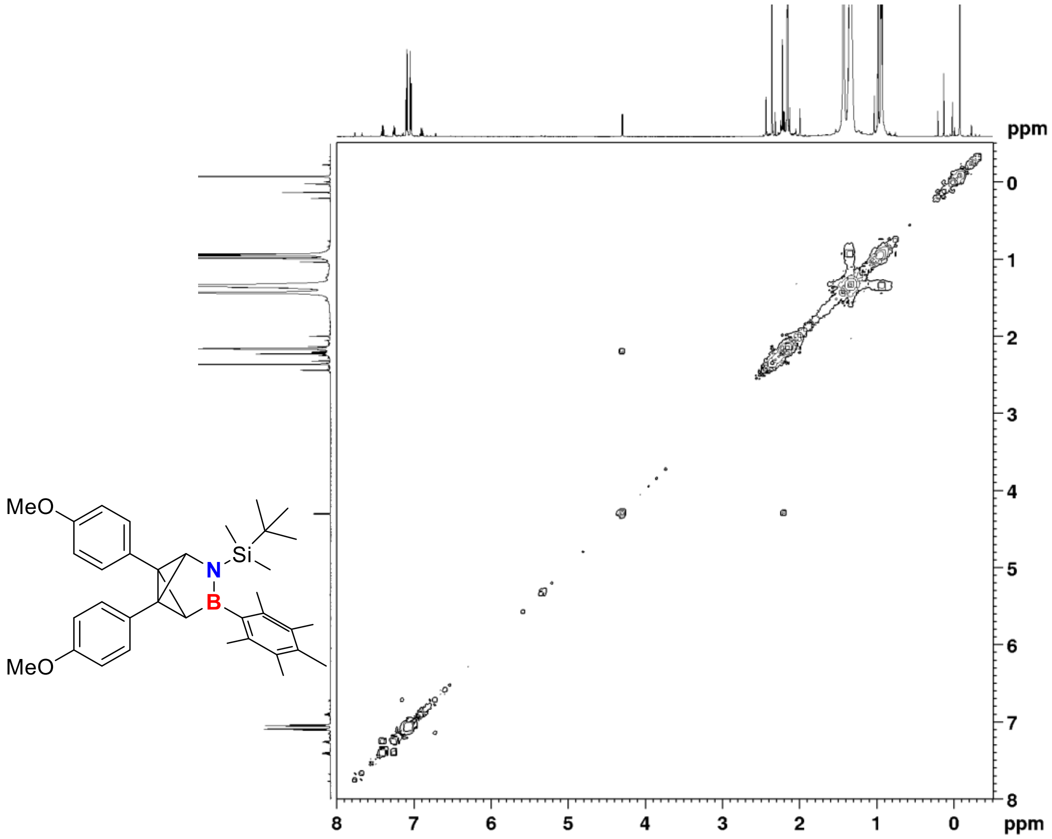

Figure S66. ^{1}H -^{1}H-NMR spectrum of compound ^{BN}**V3$_{OMe}$** in C_6D_{12} measured at a 600 MHz spectrometer.

*NMR data of ^{BN}**V3**$_{SMe}$*

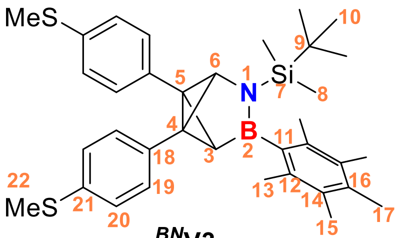

$C_{35}H_{46}BS_2NSi$ (583.78 g/mol)

^{1}H-NMR (400 MHz, **C_6D_{12}**): δ = 7.09 (d, $^3J_{HH}$ = 8.30 Hz, 4H, H-20), 7.04 (d, $^3J_{HH}$ = 8.30 Hz, 4H, H-19), 4.29 (d, $^4J_{HH}$ = 6.25 Hz, 1H, H-6), 2.35 (s, 6H, H-22), 2.22 (s, 3H, H-17), 2.20 (d, $^4J_{HH}$ = 6.25 Hz, 1H, H-4), 2.15 (s, 12H, H-13/H15), 0.98 (s, 9H, H-10), -0.08 (s, 6H, H-8) ppm.

^{13}C-{^{1}H}-NMR (100 MHz, **C_6D_{12}**): δ = 138.8, 137.0, 133.8, 133.4, 132.1, 131.7, 128.0, 126.9, 58.2, 56.3, 39.8, 32.9, 30.2, 27.4, 21.9, 19.7, 16.6, 16.0, -4.7 ppm.

^{11}B-{^{1}H}-NMR (128 MHz, **C_6D_{12}**): δ = 51.6 ppm.

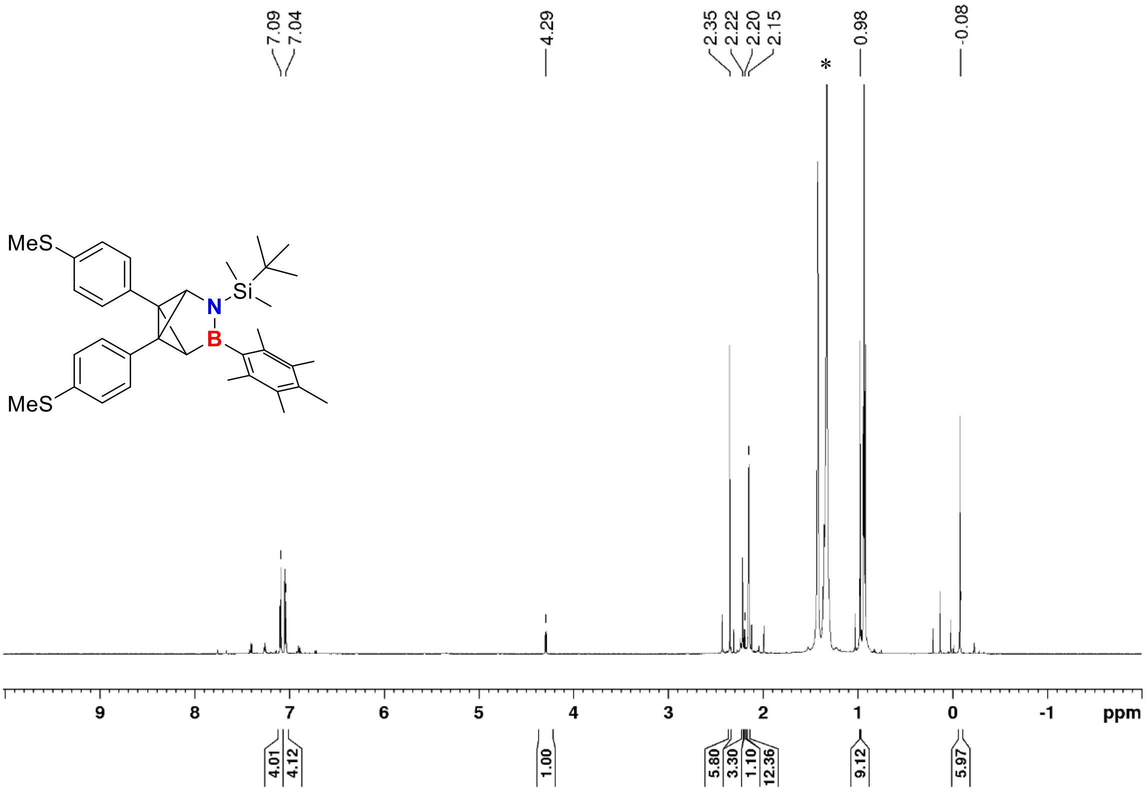

Figure S67. ^{1}H-NMR spectrum of compound ^{BN}**V3**$_{\mathbf{SMe}}$ in C_6D_{12} measured at a 600 MHz spectrometer. The solvent signal is marked with an asterisk.

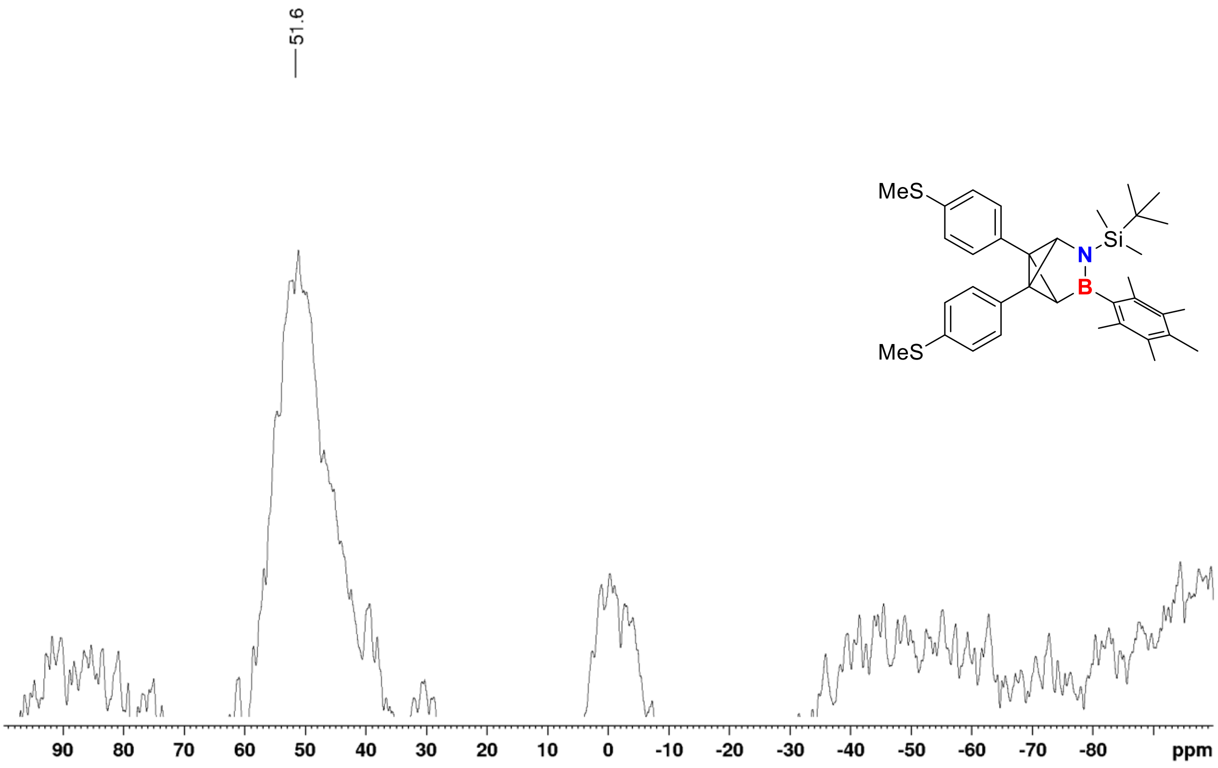

Figure S68. ^{11}B-{^{1}H}-NMR spectrum of compound ^{BN}**V3**$_{\mathbf{SMe}}$ in C_6D_{12} measured at a 600 MHz spectrometer.

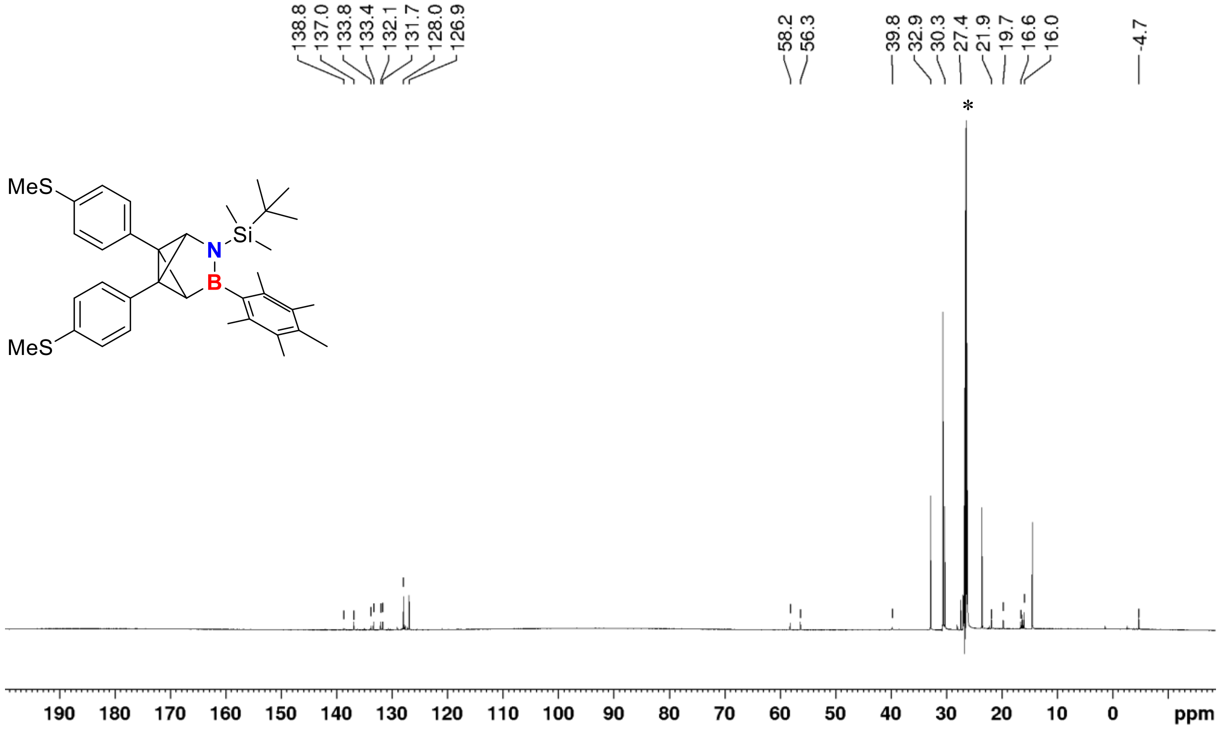

Figure S69. ^{13}C -{^{1}H}-NMR spectrum of compound ^{BN}**V3**$_{\mathbf{SMe}}$ in C_6D_{12} measured at a 600 MHz spectrometer. The solvent signal is marked with an asterisk.

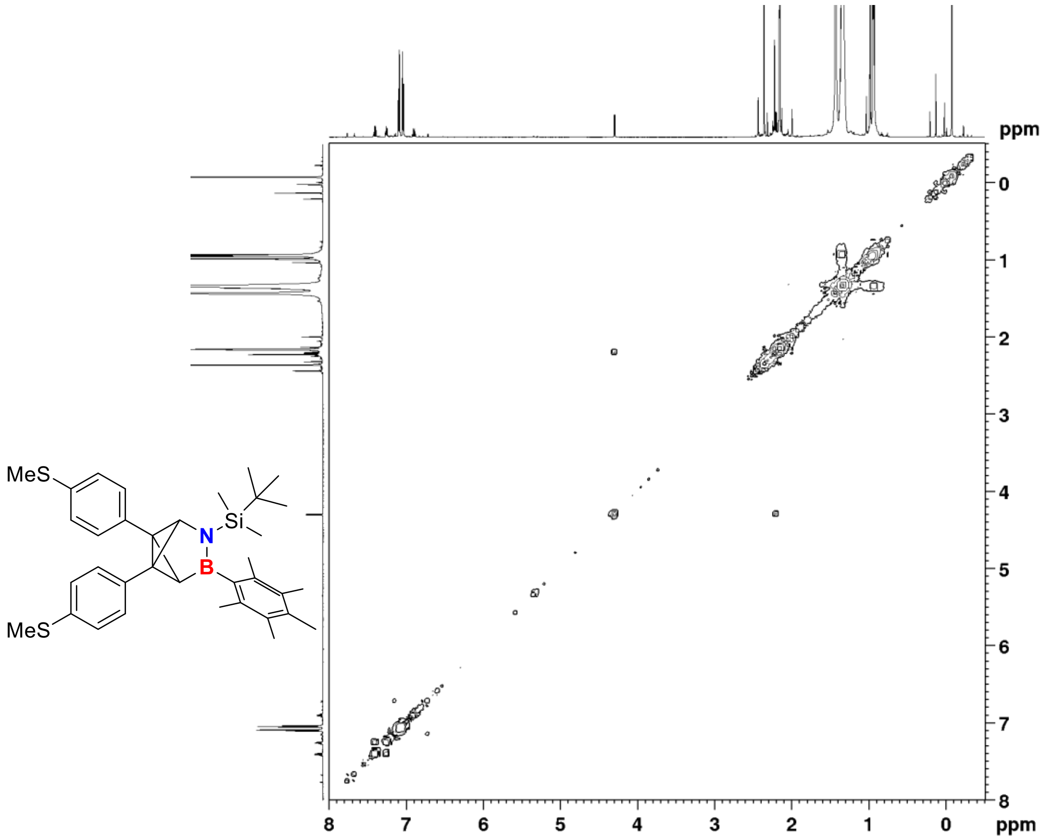

Figure S70. ^{1}H -^{1}H-COSY-NMR spectrum of compound ^{BN}**V3**$_{\mathbf{SMe}}$ in C_6D_{12} measured at a 600 MHz spectrometer.

*NMR data of ^{BN}**V3**$_{NMe2}$*

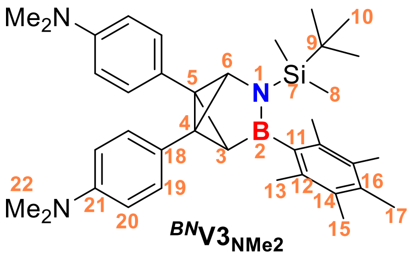

^{BN}**V3**$_{NMe2}$

$C_{37}H_{52}BN_3Si$ (577.74 g/mol)

^{1}H-NMR (400 MHz, **C_6D_{12}**): δ = 6.99 (d, $^3J_{HH}$ = 9.10 Hz, 4H, H-20), 6.54 (d, $^3J_{HH}$ = 9.10 Hz, 4H, H-19), 4.07 (d, $^4J_{HH}$ = 6.22 Hz, 1H, H-6), 2.81 (s, 12 H, H-22), 2.16 (s, 3H, H-17), 2.12 (s, 6H, H-15), 2.11 (s, 6H, H-13), 1.94 (d, $^4J_{HH}$ = 6.22 Hz, 1H, H-6), 0.93 (s, 9H, H-10), -0.14 (s, 6H, H-8) ppm.

^{13}C-{^{1}H}-NMR (100 MHz, **C_6D_{12}**): δ = 141.7, 139.6, 135.3, 135.5, 133.5, 131.4, 129.3, 127.8, 123.7, 113.6, 68.1, 58.3, 54.9, 40.9, 40.1, 27.5, 21.9, 19.8, 16.6, 16.0, -4.6 ppm.

^{11}B-{^{1}H}-NMR (128 MHz, **C_6D_{12}**): δ = 52.8 ppm.

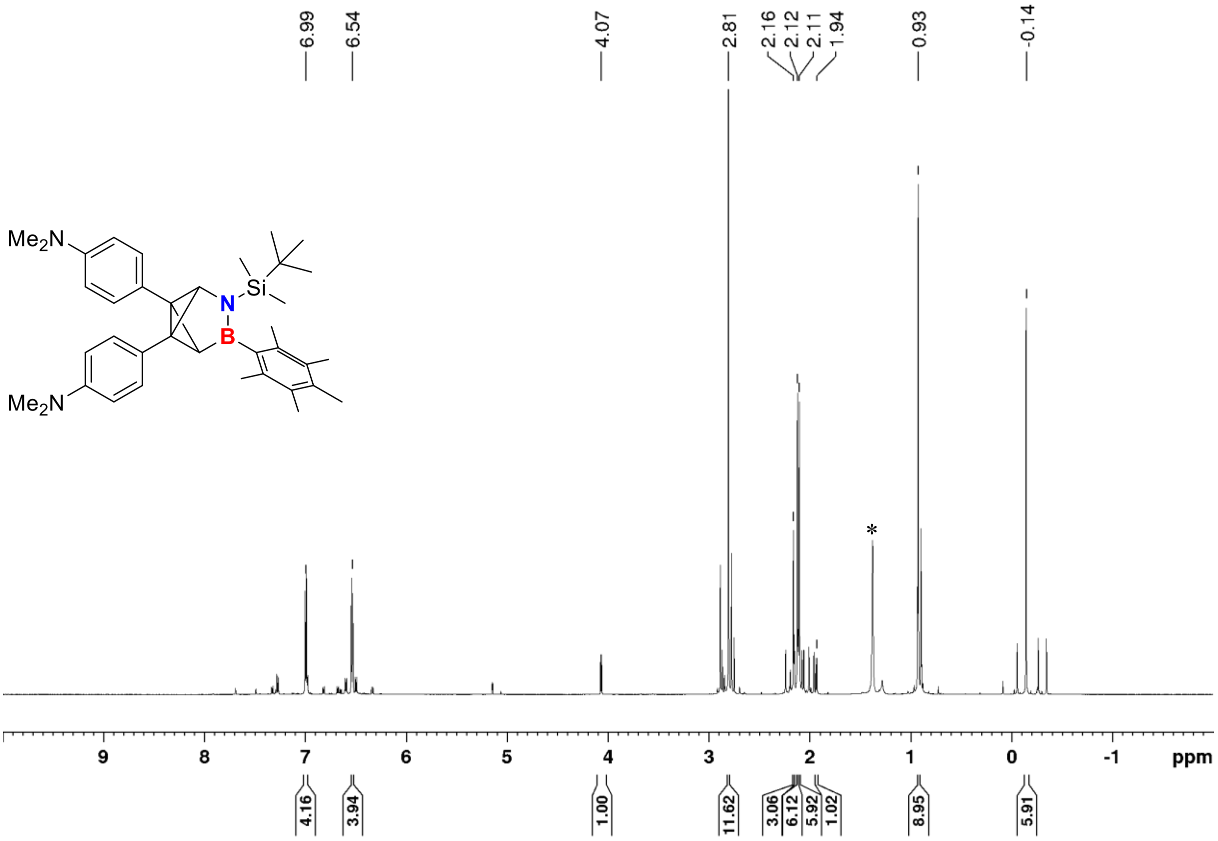

Figure S71. ^{1}H-NMR spectrum of compound ^{BN}**V3**$_{\mathbf{NMe2}}$ in C_6D_{12} measured at a 600 MHz spectrometer. The solvent signal is marked with an asterisk.

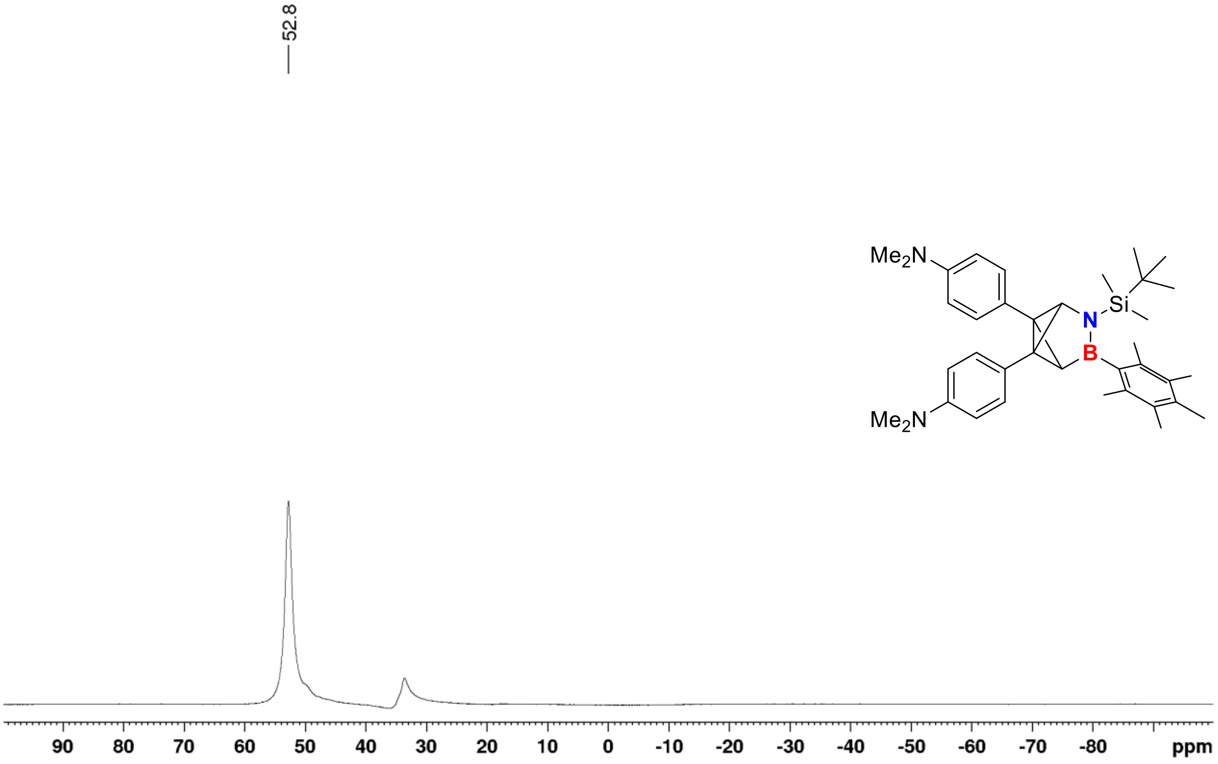

Figure S72. ^{11}B-{^{1}H}-NMR spectrum of compound ^{BN}**V3**$_{\mathbf{NMe2}}$ in C_6D_{12} measured at a 600 MHz spectrometer.

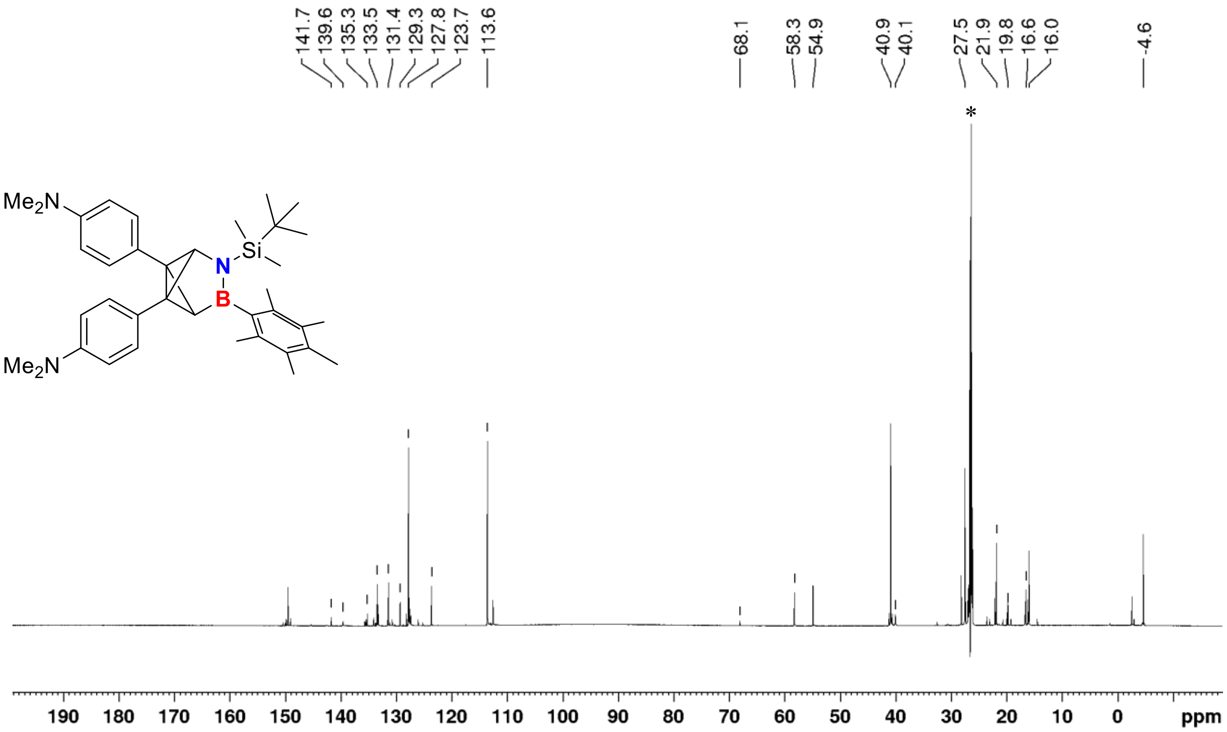

Figure S73. ^{13}C -{^{1}H}-NMR spectrum of compound ^{BN}**V3**$_{\mathbf{NMe2}}$ in C_6D_{12} measured at a 600 MHz spectrometer. The solvent signal is marked with an asterisk.

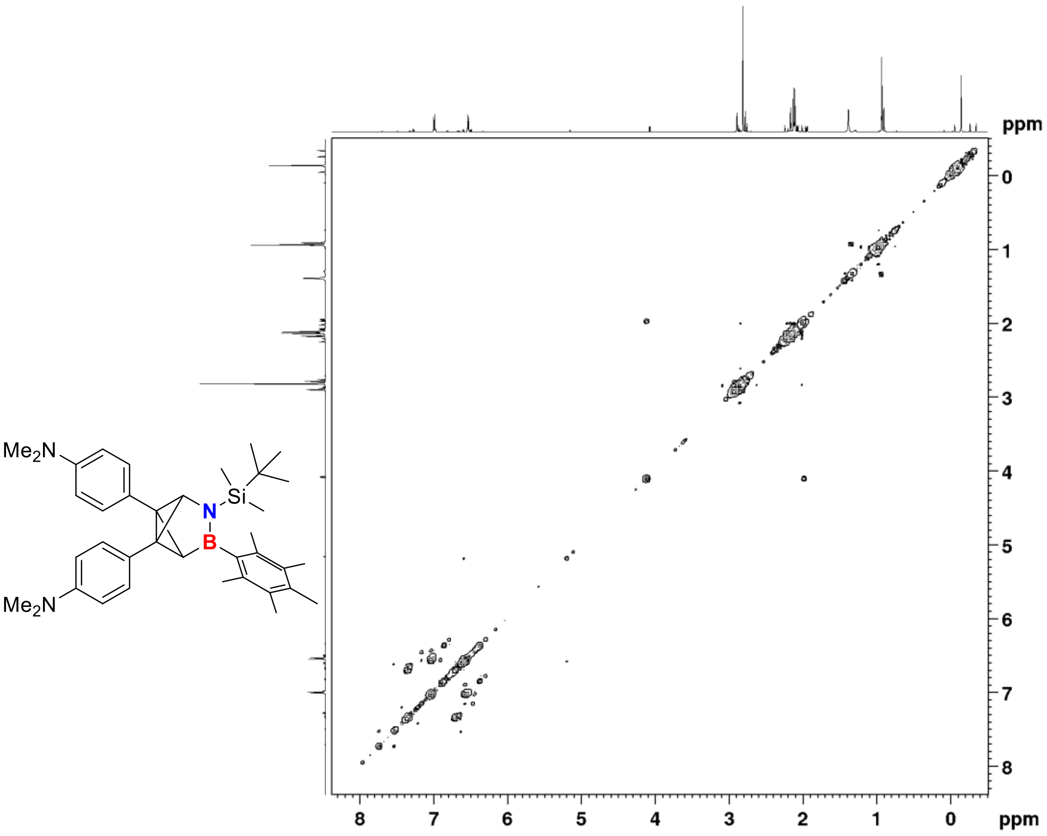

Figure S74. ^{1}H -^{1}H-COSY-NMR spectrum of compound ^{BN}**V3**$_{\mathbf{NMe2}}$ in C_6D_{12} measured at a 600 MHz spectrometer.

Irradiation of ^{BN}**B3**$_{\mathbf{NMe2}}$ in the presence of TMS (Tetramethylsilane)

To confirm that the observed photoisomerizations proceed without simultaneous decomposition, additional irradiation experiments were performed using tetramethyl silane (TMS) as an internal standard. For LED irradiation a 0.12 µM as well as a 1 µM solution of ^{BN}**B3**$_{\mathbf{NMe2}}$ in C_6D_{12} were used, while a more concentrated (0.01 mM) solution was employed for the broadband irradiation (280-400 nm, high pressure mercury lamp).

In an argon glovebox the ^{BN}**B3**$_{\mathbf{NMe2}}$ solutions and the internal standard were combined in a quartz glass J.-Young NMR tube. Before and after each irradiation (see table S1 for wavelength and irradiation time) the conversion relative to the internal NMR standard was determined by NMR integration (the integral of the TMS standard was calibrated to 100 a. u. in all experiments). To maintain the highest reproducibility and consistency of the integration ranges possible the integration was conducted automized using TopSpin. Figure S75 shows the spectra of the 0.01 mM solution before and after irradiation.

As listed in table S1 the signal intensity relative to TMS shows only minor variations, which supports the conclusion that no decomposition occurs in parallel to the photoisomerization.

Table S1. Parameters of the irradiation and NMR measurements using tetramethyl silane (TMS) as the internal standard. Entry 1 refers to the 0.1 µM solution, entry 2 to the 1 µM solution, and entry 3 to the 0.01 mM solution. The integrals of the three photoisomers are reported in arbitrary units (a.u.), with the TMS signal set to 100 a. u. The sum of the integrals of the three photoisomers is also given in arbitrary units (a.u.).

	T / K	λ / nm	t / min	^{BN}**B3**$_{\mathbf{NMe2}}$	^{BN}**D3**$_{\mathbf{NMe2}}$	^{BN}**BV**$_{\mathbf{NMe2}}$	Σ
1		-	-	20.29	-	-	20.29
		385	5	3.39	16.00	-	19.39
		365	3	1.42	3.14	14.75	19.31
2	293 K (NMR)	-	-	21.79	-	-	21.79
		385	10	2.60	14.50	4.24	21.34
		365	8	-	-	21.19	21.19
3		-	-	18.97	-	-	18.97
		280-400	4	0.36	-	18.15	18.51

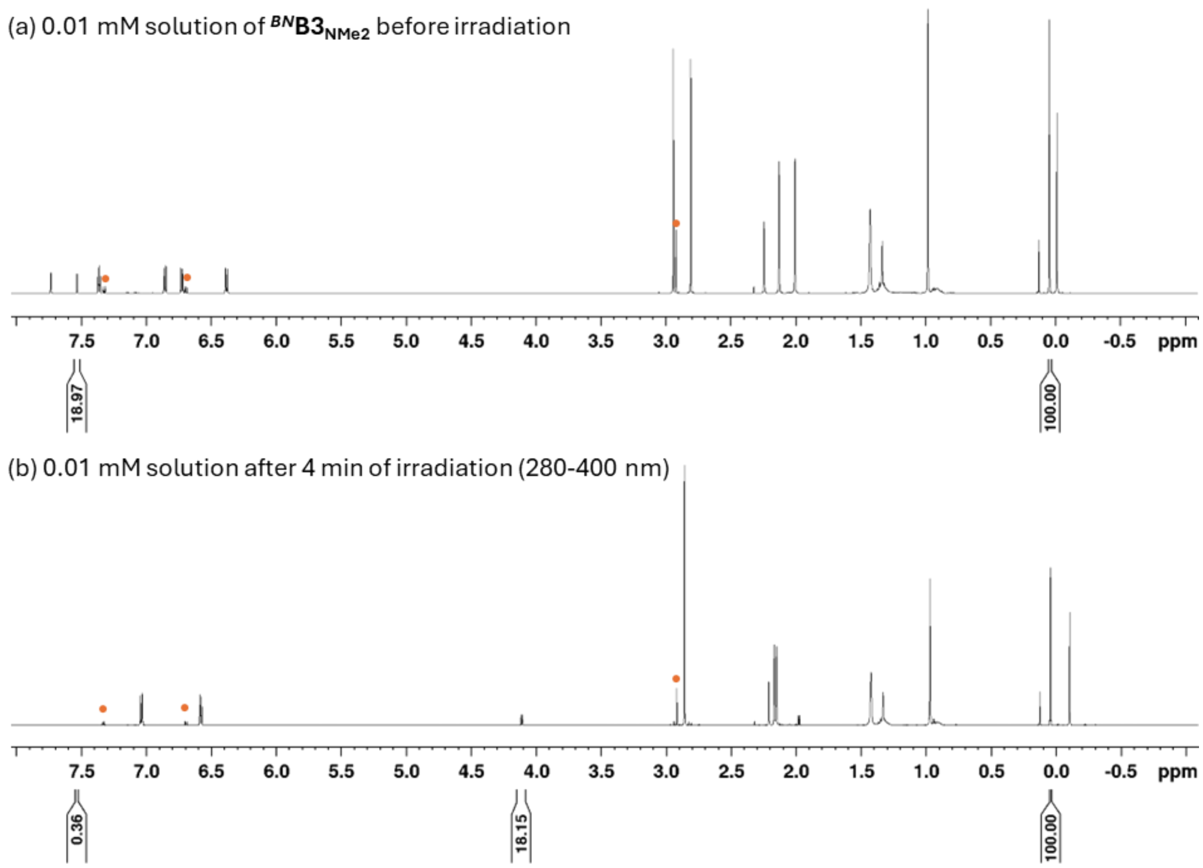

Figure S75. ^{1}H NMR spectra of the 0.01 mM solution before and after irradiation with 280-400 nm for 4 minutes. The signals assigned with orange dots belong to an impurity with *para-N,N*-(dimethylamino)boronic acid.

5. Kinetic experiments

Sample preparation

Dihydroazaborinin ***^{BN}*B3$_H$** was solved in deuterated cyclohexane (C_6D_{12}). The solution was 0.04 M placed in a J. Young NMR tube (quartz glass) and an irradiation as described in section 4 was conducted to obtain ***^{BN}*V3$_H$**.

Experimental details

In all kinetic experiments the sample was heated within the NMR-spectrometer and ^{1}H NMR spectra (16 scans; total experiment time = 126 s) were recorded every 5 Minutes to monitor the conversion.

Initial experiments in deuterated cyclohexane (C_6D_{12}) at 50 °C and 75 °C did not lead to the formation of ***^{BN}*B3$_H$** or any other detectable change of the ***^{BN}*V3$_H$** solution. To enable further heating the solvent was removed in vacuo and deuterated mesitylene (C_9H_{12}) was added instead.

The measured temperatures, as well as the duration of the experiment and the conversion achieved within this time frame are listed in Table S1.

Table S2. Temperature, time and conversion of the high-temperature NMR measurements for the investigation of reaction kinetics.

No.	temperature / K	time / h	conversion / %
1	383	7.0	32
2	388	5.5	35
3	398	4.5	32
4	403	2.0	17
5	403	8.0	56

Determination of the reaction order

As the thermal cycloreversion of ^{BN}**V** to ^{BN}**B** was not reported or investigated before, the order of the reaction was analyzed based on the measured data. Since this is a unimolecular reaction, it is reasonable to assume that it follows first-order kinetics.

Time dependence (0. order kinetic)

For deriving the concentration dependence, the following assumptions are made. The reaction under consideration is of the type:

$$A \xrightarrow{k_0} B$$

The process is assumed to be irreversible, follows a zero order kinetic and has the rate constant k_0. The initial concentration of compound A is $A_0 = 1$. While the initial concentrations of B is $B_0 = 0$. The reaction of substrate A is independent of its concentration, therefore its concentrations behavior is given as:

$$\frac{d[A]}{dt} = -k_0$$

This equation can be integrated to

$$[A] = A_0 - k_0 \cdot t \tag{1}$$

Time dependence (1. order kinetic)

For deriving the concentration dependence, the following assumptions are made. The reaction under consideration is of the type:

$$A \xrightarrow{k_1} B$$

The process is assumed to be irreversible, follows a first order kinetic and has the rate constant k_1. The initial concentration of compound A is $A_0 = 1$. While the initial concentrations of B is $B_0 = 0$.

If compound A follows a simple first order kinetic, its concentrations behavior is given as:

$$\frac{d[A]}{dt} = -k_1 \cdot [A]$$

This equation can be transformed to

$$\frac{d[A]}{[A]} = -k_1 \cdot dt$$

An integration from A_0 to A (left side) or 0 to t (right side) respectively, results in

$$ln[A] - lnA_0 = -k_1 \cdot t$$

which finally rearranges to the well-known formula equation 1.

$$[A] = A_0 \cdot e^{-k \ \cdot t} \qquad (2)$$

Time dependence (2. order kinetic)

For deriving the concentration dependence, the following assumptions are made. The reaction under consideration is of the type:

$$2\ A \xrightarrow{k_2} 2\ B$$

The process is assumed to be irreversible, follows a first order kinetic and has the rate constant k_2. The initial concentration of compound A is $A_0 = 1$. While the initial concentrations of B is $B_0 = 0$.

If compound A follows a simple first order kinetic, its concentrations behavior is given as:

$$\frac{d[A]}{dt} = -k_2 \cdot [A]^2$$

This equation can be transformed to

$$\frac{d[A]}{[A]^2} = -k_2 \cdot dt$$

An integration from A_0 to A (left side) or 0 to t (right side) respectively, results in

$$\frac{1}{A_0} - \frac{1}{[A]} = -k_2 \cdot t$$

which finally rearranges to the well-known formula equation 1.

$$[A] = \frac{A_0}{(1 + 2k_2[A_0]t)} \tag{3}$$

Order of the thermal cycloreversion of ^{BN}***V3***

Linearization of the experimental data according to zero-, first-, and second-order reaction models (see Fig. S74) confirms this assumption. The coefficient of determination (R^2) of the regression is highest for the first-order model. A longer measurement at 403 K should achieve a higher degree of conversion and thus allow a more reliable conclusion regarding the reaction order. In this case, the best linearizability was again obtained when treating the data as first-order kinetics.

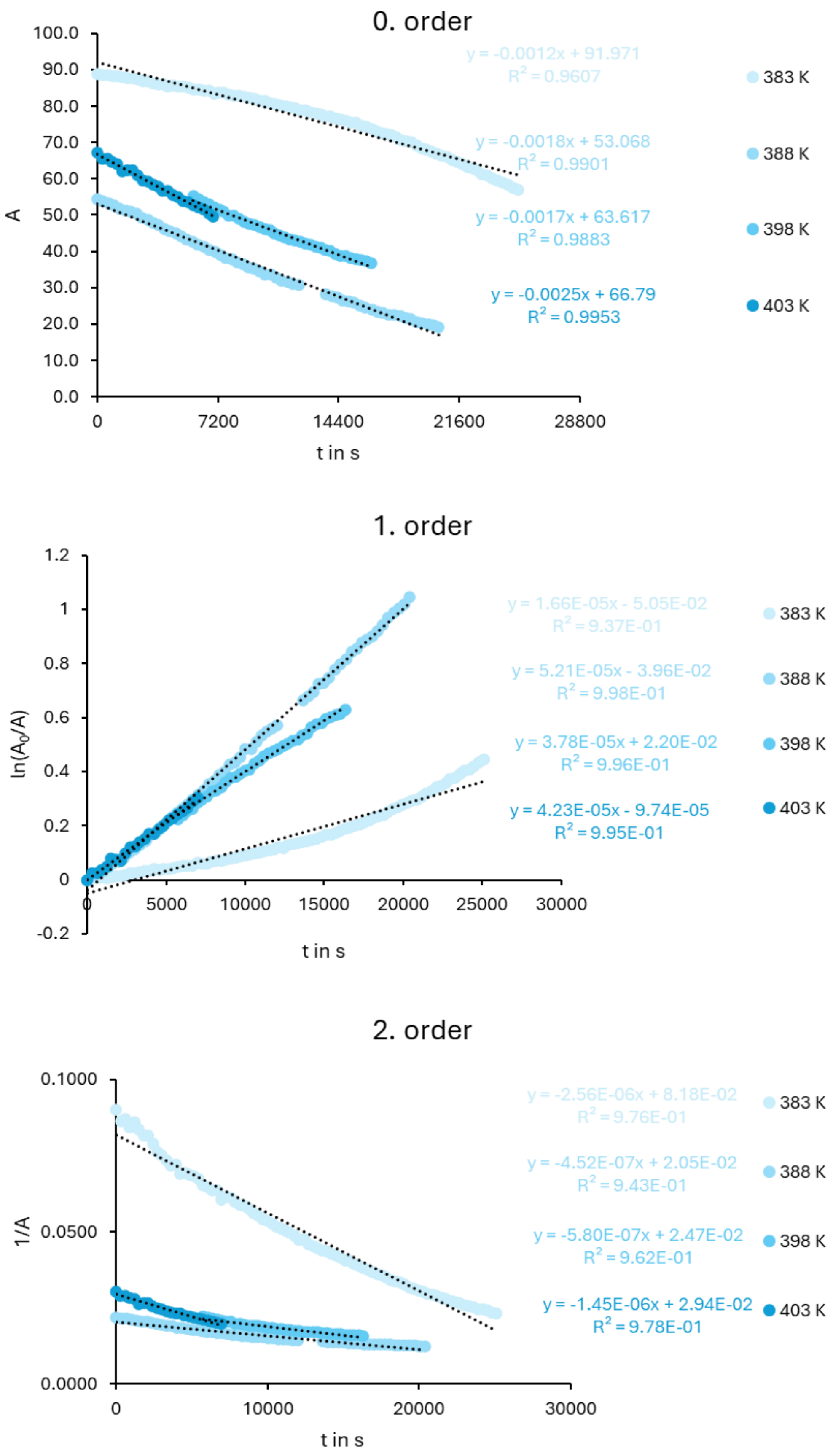

Figure S76. Linearization of the experimentally obtained data at 383 K, 388 K, 398 K, and 403 K assuming zero-order (top), first-order (middle), or second-order (bottom) kinetics.

Arrhenius treatment of the measured data

The same applies to the Arrhenius evaluation of the data.

The Arrhenius equation is expressed as follows:

$$k = A \cdot e^{\frac{-E_a}{R \cdot T}}$$

A denotes the pre-exponential factor, R is the universal gas constant, k represents the rate constant, T is the absolute temperature, and E_a corresponds to the activation energy (activation barrier). By taking the natural logarithm, the equation can be rearranged into the following form:

$$ln(k) = ln(A) - \frac{E_a}{R} \cdot \frac{1}{T}$$

If ln(k) is plotted against the reciprocal temperature (1/T), this yields a straight line of the general form

$$y = a + b \cdot x$$

with a being the y-intercept and b is the slope. Consequently, the y-intercept a and the slope b of the Arrhenius plot correlate with the activation barrier and the pre-exponential factor A, respectively, as described by the following equations.

$$A = e^a$$

$$E_a = -b \cdot R$$

The Arrhenius plot derived from the experimental data, along with the equations of the corresponding linear fits, are presented below.

Table S3. Rate constants at 383 K, 388 K, 398 K, and 403 K assuming zero-, first-, or second-order kinetics

	rate constants				
	383 K	388 K	398 K	403 K	403 K (2)
**0. order in M/s**	$1.2 \cdot 10^{-3}$	$1.8 \cdot 10^{-3}$	$1.7 \cdot 10^{-3}$	$2.5 \cdot 10^{-3}$	$2.0 \cdot 10^{-3}$
**1. order in 1/s**	$1.7 \cdot 10^{-5}$	$5.2 \cdot 10^{-5}$	$3.8 \cdot 10^{-5}$	$4.2 \cdot 10^{-5}$	$4.2 \cdot 10^{-5}$
**2. order in 1/Ms**	$2.6 \cdot 10^{-6}$	$0.5 \cdot 10^{-6}$	$5.8 \cdot 10^{-6}$	$1.5 \cdot 10^{-6}$	$1.5 \cdot 10^{-6}$

Even for first-order kinetics, the coefficient of determination (R^2) of the Arrhenius plot is only 0.7347, indicating a very poor linear correlation. With respect to the rate constants and concentration profiles, several inconsistencies are observed. The rate constants at 388 K and 389 K are almost identical despite the clear temperature difference. Moreover, Fig. S77 shows that the concentration profiles at 398 K and 403 K are nearly indistinguishable, while the profile at 383 K cannot be successfully linearized under the assumption of first-order kinetics.

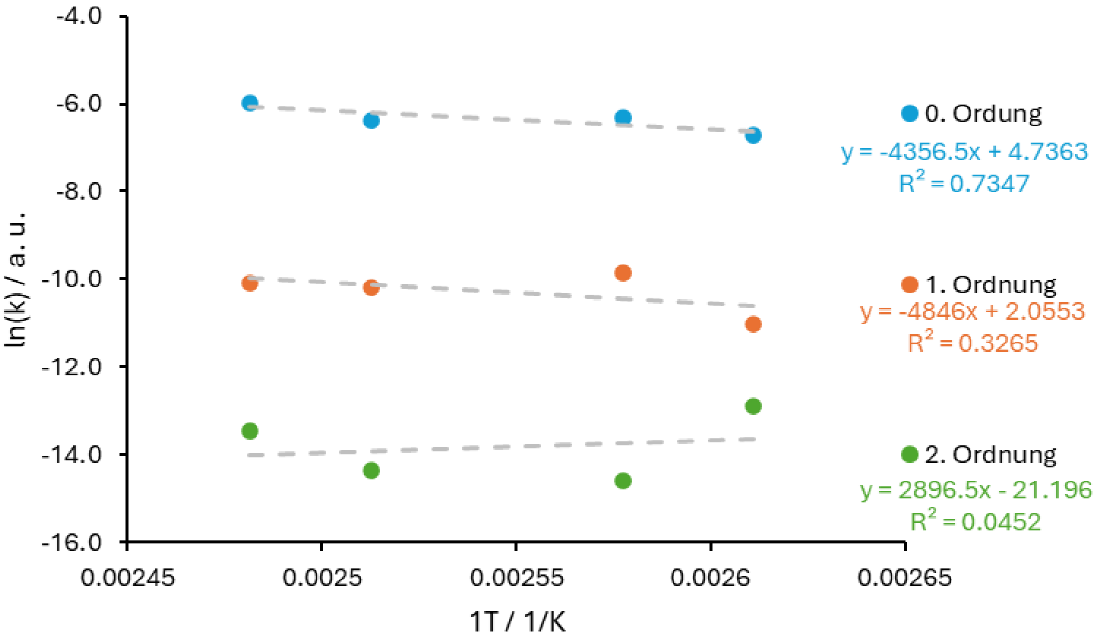

Figure S77. Arrhenius plots derived from the rate constants listed in Table S2.

When excluding the measurements at 388 K or 398 K, R^2 values of up to 0.90 can be obtained from the Arrhenius treatment of the data. As listed in Table S4, this also leads to somewhat higher activation barriers for the reaction. Nevertheless, the calculated activation barriers remain far too low to account for the thermally apparently very stable ^{BN}**V3**$_{\mathbf{H}}$. According to these barriers, ^{BN}**V3**$_{\mathbf{H}}$ should exhibit half-lives of only a few milliseconds at room temperature. In reality, however, no changes in the ^{1}H NMR spectrum of ^{BN}**V3**$_{\mathbf{H}}$ is detected even after several months of storage, and, as summarized in Table S4, only minor conversions is observed after several hours at elevated temperatures.

Table S4. Activation barriers for the thermal ring opening calculated using the Arrhenius equation and half-lives of ^{BN}**V3$_H$** at room temperature (298 K) determined with respect to the assumed first order kinetik.

	**1.order**	**all temerpatures**	**w/o 388 K**	**w/o 389 K**
Arrhenius	$E_{a1, exp}$ (kcal/mol)	8.66	10.24	10.28
	$t_{1/2,1}$ (ms)	35.3	6.9	5.8

Since the overall intensity of the NMR signals decreased markedly during heating and an orange to brown coloration of the solution was observed, it is reasonable to assume that the poor agreement between the Arrhenius analysis and the experimental findings is due to decomposition and side reactions parallel to the investigated ring-opening process. Accordingly, it is not possible to consider this process independently or to analyze it reliably.

6. Thermal cycloreversion of ^{BN}V3

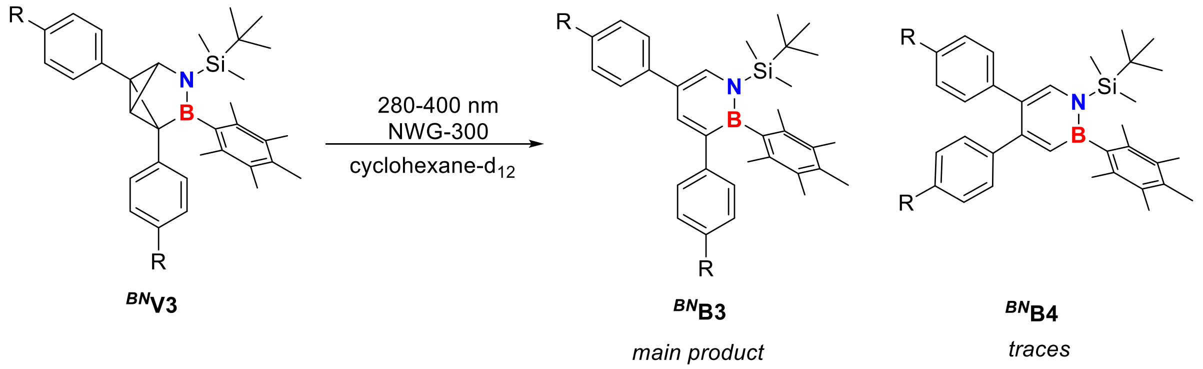

In order to thermally regenerate the dihydroazaborinines ^{BN}**B3**, solutions of ^{BN}**V3** were heated in tightly sealed J. Young NMR tubes at 100 °C for 7 days. The NMR spectra recorded after heating were consistent with those of the starting compounds (^{BN}**B3**). In addition to the product signals, weak resonances of unidentified impurities were observed. All dihydroazaborinines could be purified by column chromatography (SiO_2, n-hexane/dichloromethane gradient). Apart from the starting compounds, no additional fractions were obtained. Only in the case of ^{BN}**B3**$_{\mathbf{NMe2}}$, traces (< 1 mg) of ^{BN}**B4**$_{\mathbf{NMe2}}$ could be isolated and characterized.

NMR data of ^{BN}***B4***$_{NMe2}$

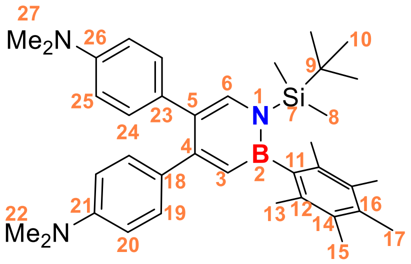

^{BN}B4$_{NMe2}$

$C_{37}H_{52}BN_3Si$ (577.74 g/mol)

^{1}H-NMR (400 MHz, **C_6D_{12}**): δ =7.36 (s, 1H, H-6), 6.95 (m, 4H, H-), 6.54 (s, 1H, H-3), 6.49 (d, $^3J_{HH}$ = 8.82 Hz, 2H, H-), 6.39 (d, $^3J_{HH}$ = 8.82 Hz, 2H, H-), 2.86 (s, 6H, H-), 2.82 (s, 6H, H-), 2.20 (s, 3H, H-17), 2.14 (s, 6H, H-), 2.10 (s, 6H , H-), 0.96 (s, 9H, H-10), -0.03 (s, 6H, H-8) ppm.

^{13}C-{^{1}H}-NMR (100 MHz, **C_6D_{12}**): δ = 156.2, 149.9, 149.2, 136.9, 134.6, 133.3, 133.1, 132.7, 131.4,131.1, 131.0, 130.8, 126.3, 112.6, 112.0, 40.7, 40.6, 28.1, 22.6, 19.9, 16.6, 16.2, -2.8 ppm.

^{11}B-{^{1}H}-NMR (128 MHz, **C_6D_{12}**): δ = 40.3 ppm.

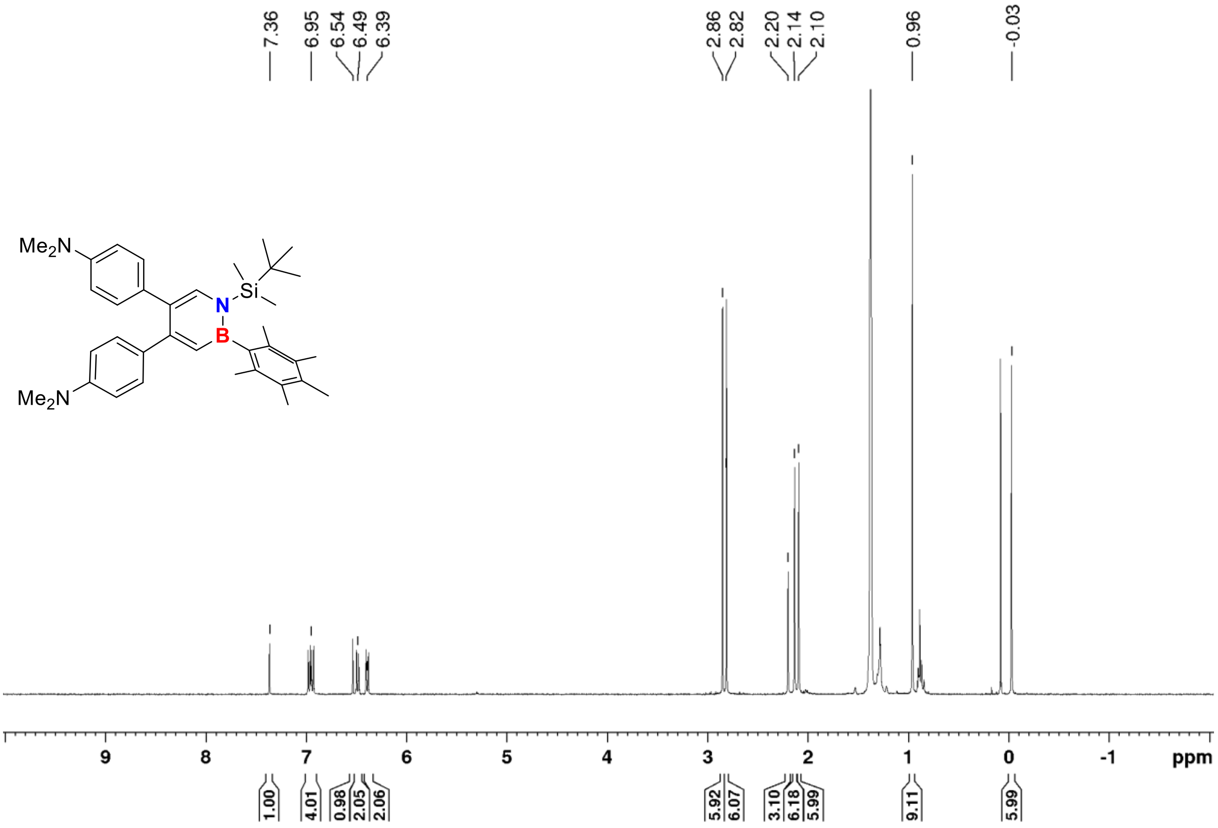

Figure S78. ^{1}H-NMR spectrum of compound ^{BN}**B4**$_{NMe2}$ in C_6D_{12} measured at a 600 MHz spectrometer. The solvent signal is marked with an asterisk.

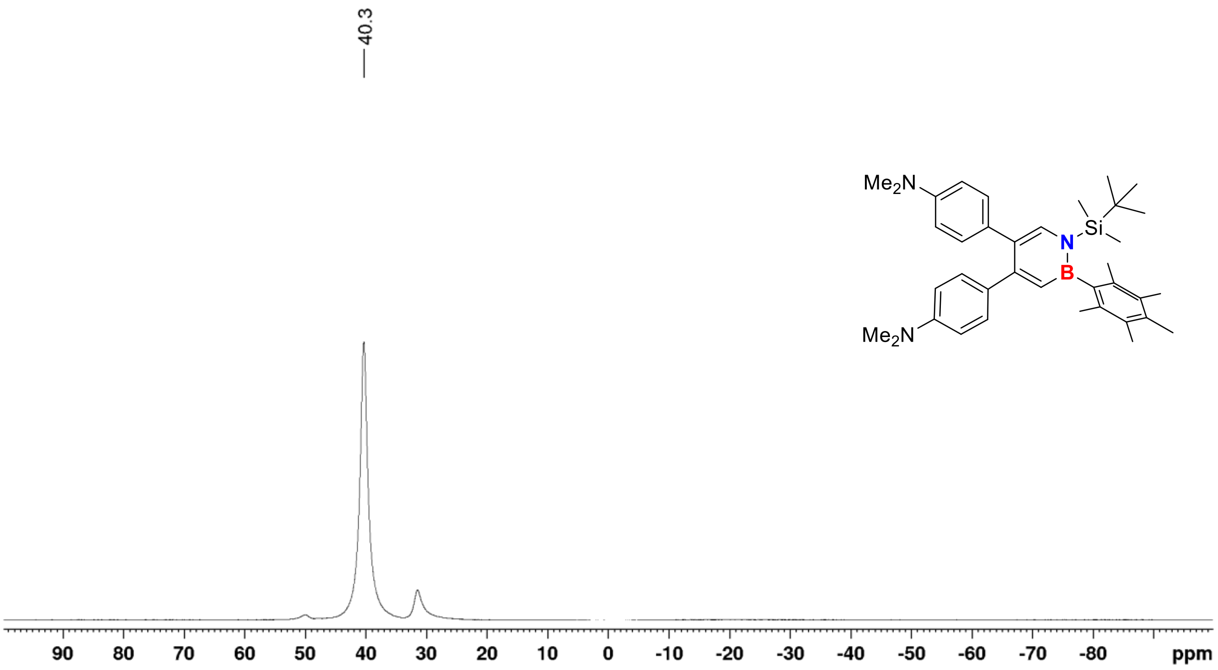

Figure S79. ^{11}B-{^{1}H}-NMR spectrum of compound ^{BN}**B4**$_{NMe2}$ in C_6D_{12} measured at a 600 MHz spectrometer.

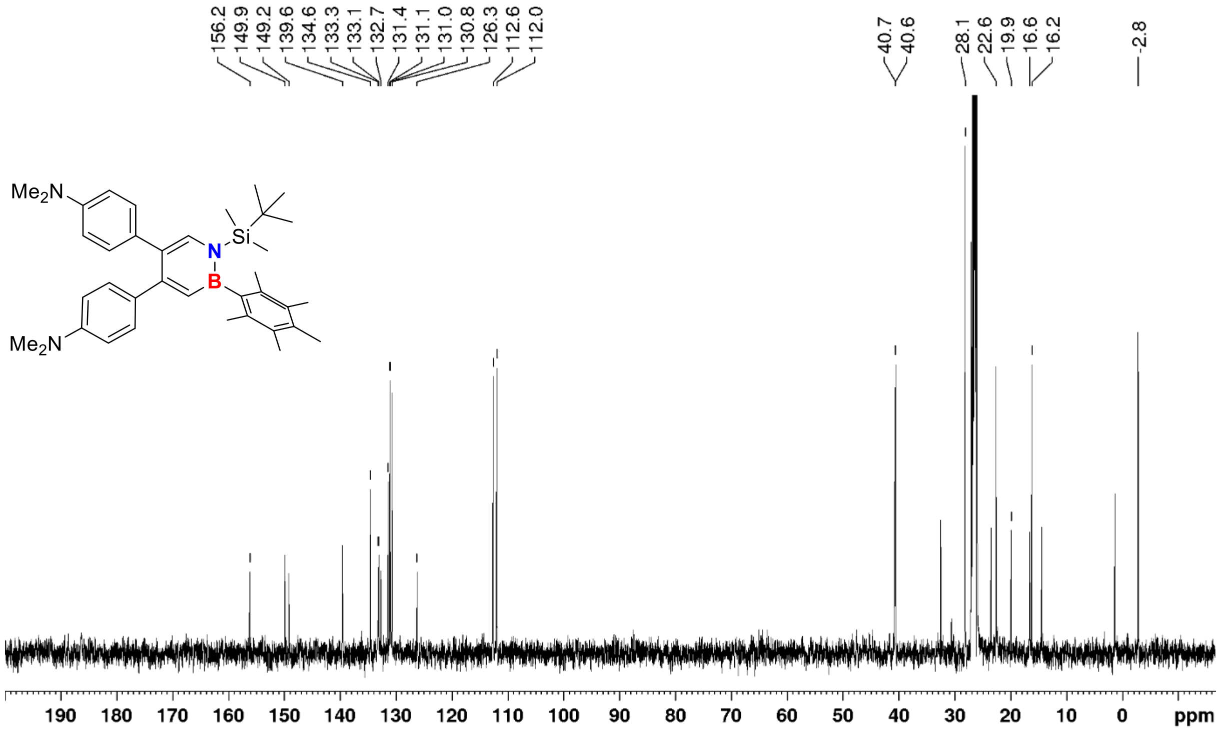

Figure S80. ^{13}C -{^{1}H}-NMR spectrum of compound ^{BN}**B4**$_{NMe2}$ in C_6D_{12} measured at a 600 MHz spectrometer. The solvent signal is marked with an asterisk.

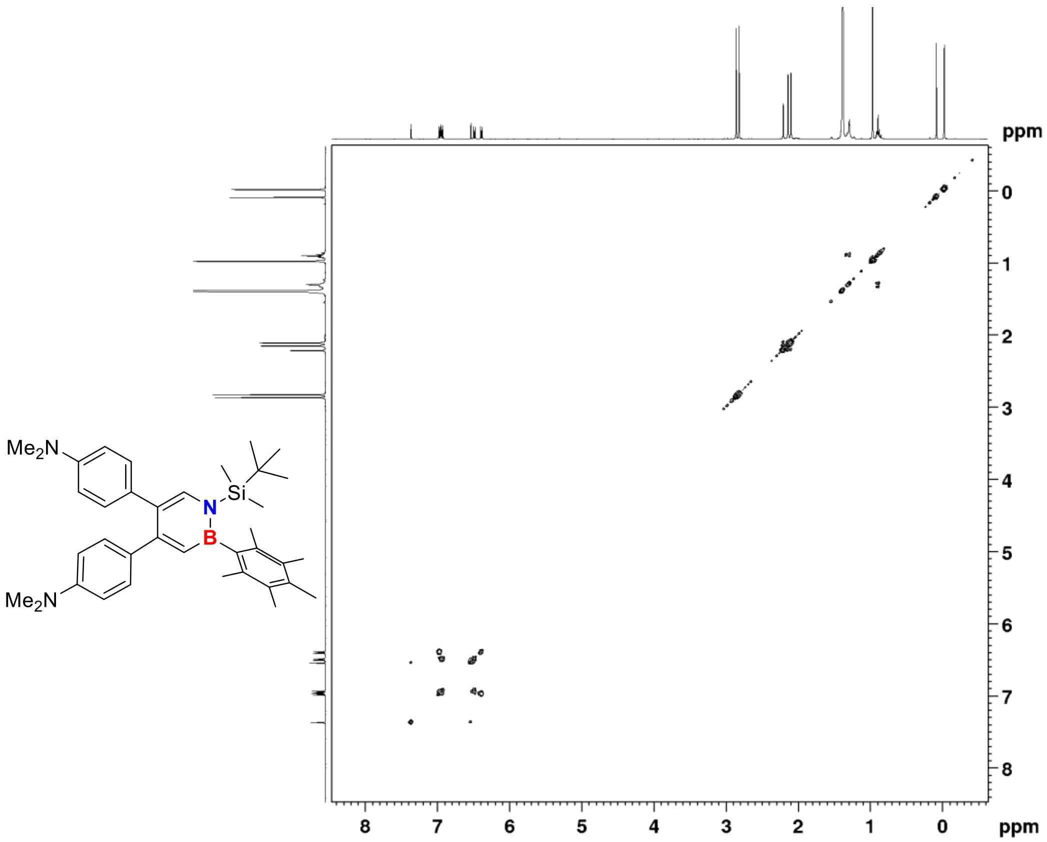

Figure S81. ^{1}H -^{1}H-COSY-NMR spectrum of compound ^{BN}**B4**$_{NMe2}$ in C_6D_{12} measured at a 600 MHz spectrometer.

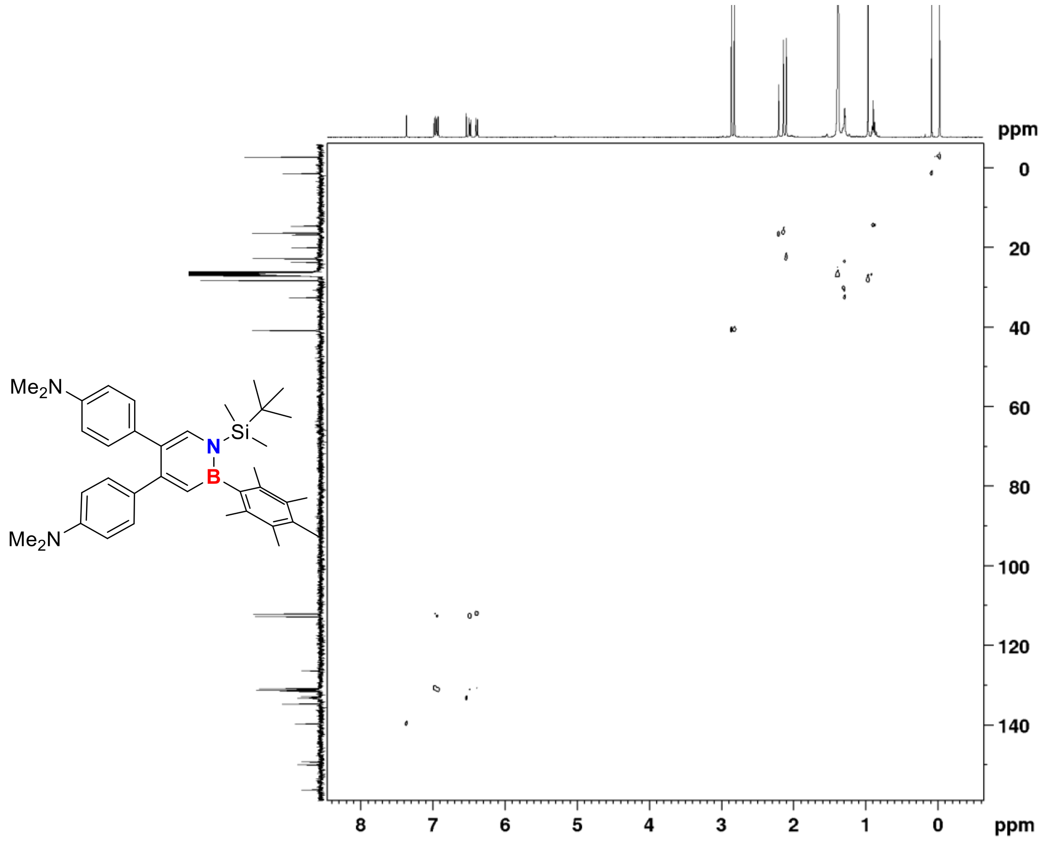

Figure S82. ^{1}H -^{13}C-HSQC-NMR spectrum of compound ^{BN}**B4**$_{\mathbf{NMe2}}$ in C_6D_{12} measured at a 600 MHz spectrometer.

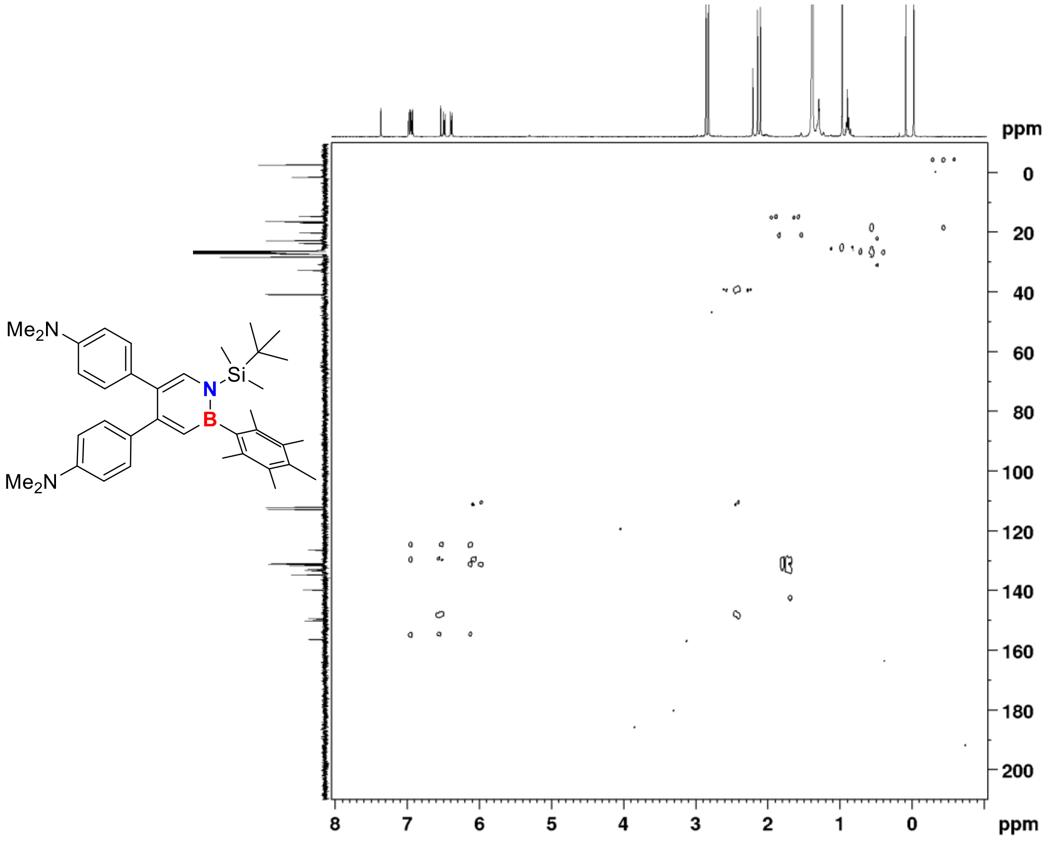

Figure S83. ^{1}H -^{13}C-HMBC-NMR spectrum of compound ^{BN}**B4**$_{\mathbf{NMe2}}$ in C_6D_{12} measured at a 600 MHz spectrometer.

7. Crystal structures

Refinement details

The implementation NoSpherA2 for non-spherical atom form factors in *Olex2* makes use of tailor made aspherical atomic form factors calculated from a Hirshfeld-partitioned electron density (ED), not from spherical atom form factors.[6] The electron density is calculated from a gaussian basis set single determinant SCF wavefunction for a fragment of the crystal. This fragment can be embedded in an electrostatic crystal field by employing cluster charges or modelled using implicit solvation models, depending on the software used. This was computed using B3LYP/6-31G(d,p),[7-10] normal integration accuracy, a charge of 0 and a multiplicity of 1 with Orca 5.0.[11]

Table S5. Parameters and results of the X-ray diffraction measurement of compound ^{BN}**B5**.

**Parameter**	**unit**	^{BN}**B5**
Molecular formula		$C_{21}H_{34}BNSi$
CCDC		2428116
$D_{calc.}$	g cm^{-3}	1.070
μ	mm^{-1}	0.967
Molar weight	g/mol	339.420
Colour		clear colourless
Shape		block-shaped
Size	mm^3	0.20×0.17×0.09
T/K		150.00(10)
Crystal System		monoclinic
Space Group		$P2_1/c$
a/Å		10.6563(1)
b/Å		11.3950(1)
c/Å		17.8536(2)
α/°		90
β/°		103.570(1)
γ/°		90
V	$Å^3$	2107.42(4)
Z		4
Z'		1
Wavelength	Å	1.54184
Radiation type		Cu K_α
Θ_{min}	°	4.27
Θ_{max}	°	79.87
Measured Refl's.		89671
Indep't Refl's		4592
Refl's I≥2 σ(I)		4498
R_{int}		0.0158
Parameters		523
Restraints		0
Largest Peak		0.1678
Deepest Hole		-0.1859
GooF		1.0823
wR_2 (all data)		0.0378
wR_2		0.0377
R_1 (all data)		0.0172
R_1		0.0169

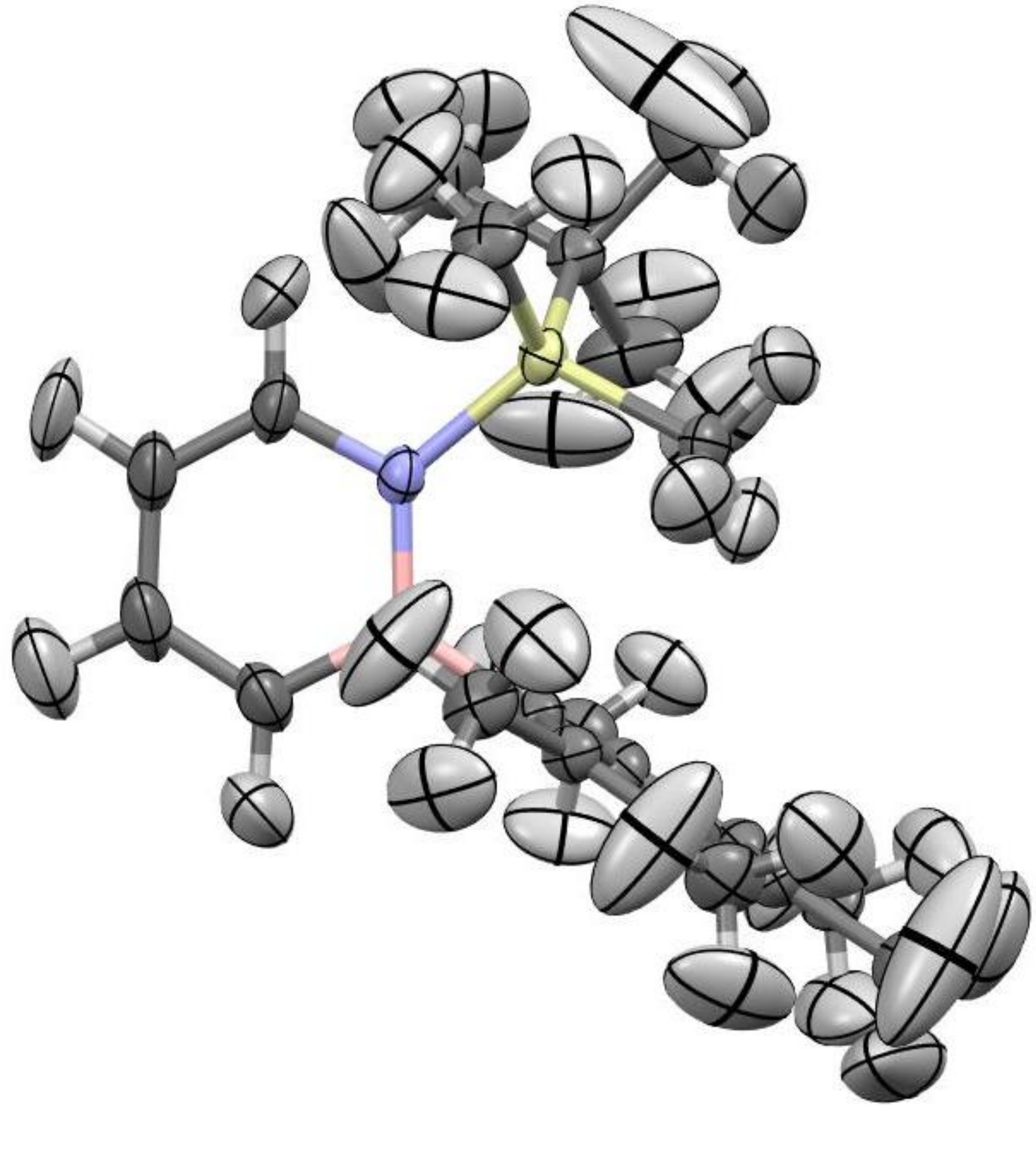

Figure S84. Crystal structure of ^{BN}**B5**. Thermal ellipsoids are drawn at the 50% probability level.

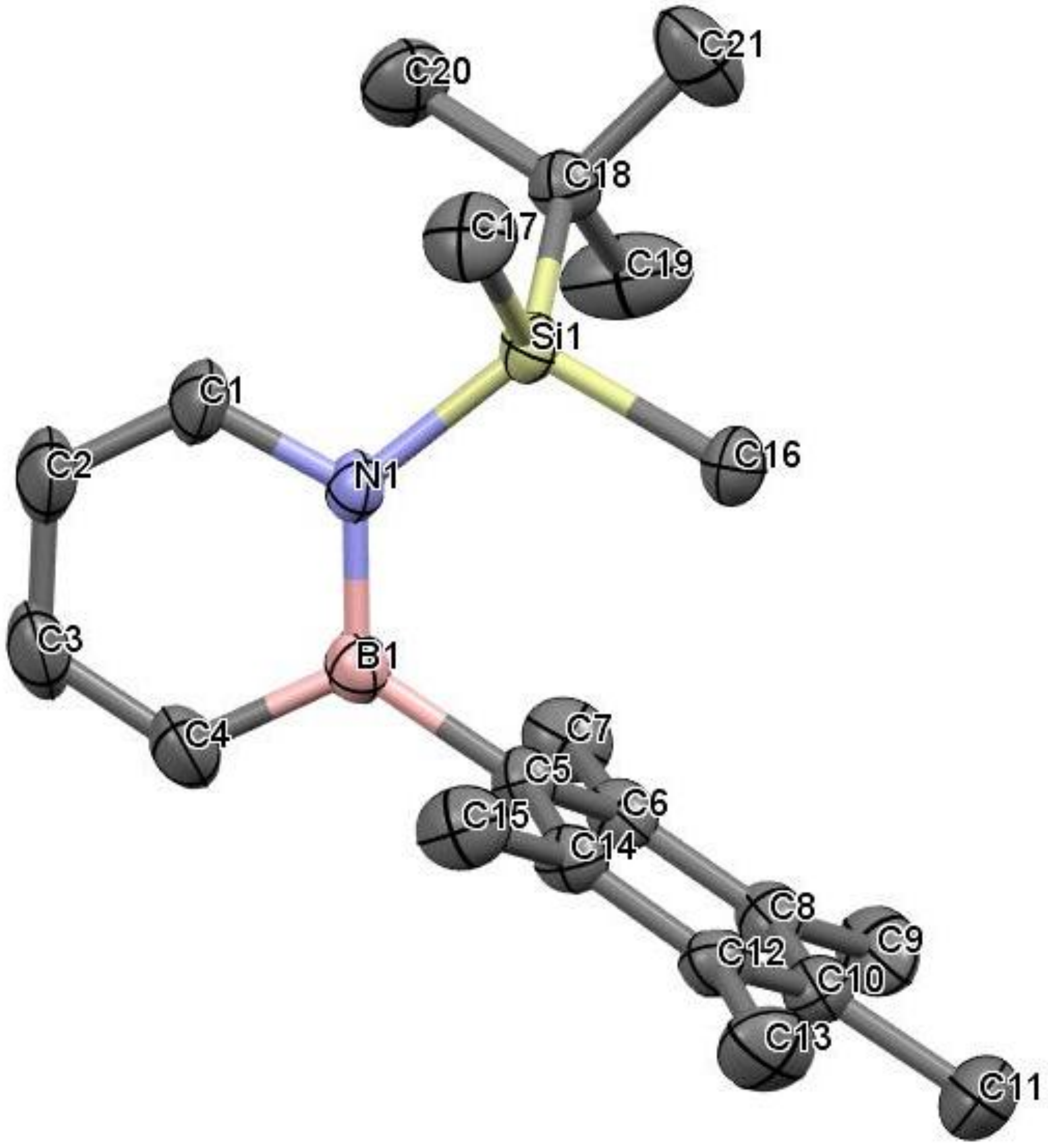

Figure S85. Crystal structure of ^{BN}**B5**. Hydrogens are omitted for clarity and thermal ellipsoids are drawn at the 50% probability level.

Table S6. Parameters and results of the X-ray diffraction measurement of compound ^{BN}**B3**$_{\mathbf{H}}$.

Parameter	unit	^{BN}B3$_{H}$
Molecular formula		$C_{33}H_{42}BNSi$
CCDC		2424411
$D_{calc.}$	g cm^{-3}	1.117
μ	mm^{-1}	0.846
Molar weight	g/mol	491.618
Colour		clear colourless
Shape		block-shaped
Size	mm^3	0.14×0.13×0.08
T/K		150.00(19)
Crystal System		triclinic
Space Group		P-1
a/Å		10.3131(3)
b/Å		12.2086(4)
c/Å		12.6389(4)
α/°		83.033(3)
β/°		72.481(3)
γ/°		74.552(2)
V	Å^3	1461.25(8)
Z		2
Z'		1
Wavelength	Å	1.54184
Radiation type		Cu K$_{\alpha}$
Θ_{min}	°	3.67
Θ_{max}	°	79.98
Measured Refl's.		66360
Indep't Refl's		6311
Refl's I≥2 σ(I)		6072
R_{int}		0.0150
Parameters		703
Restraints		0
Largest Peak		0.3424
Deepest Hole		-0.1243
GooF		1.1887
wR_2 (all data)		0.0381
wR_2		0.0378
R_1 (all data)		0.0157
R_1		0.0151

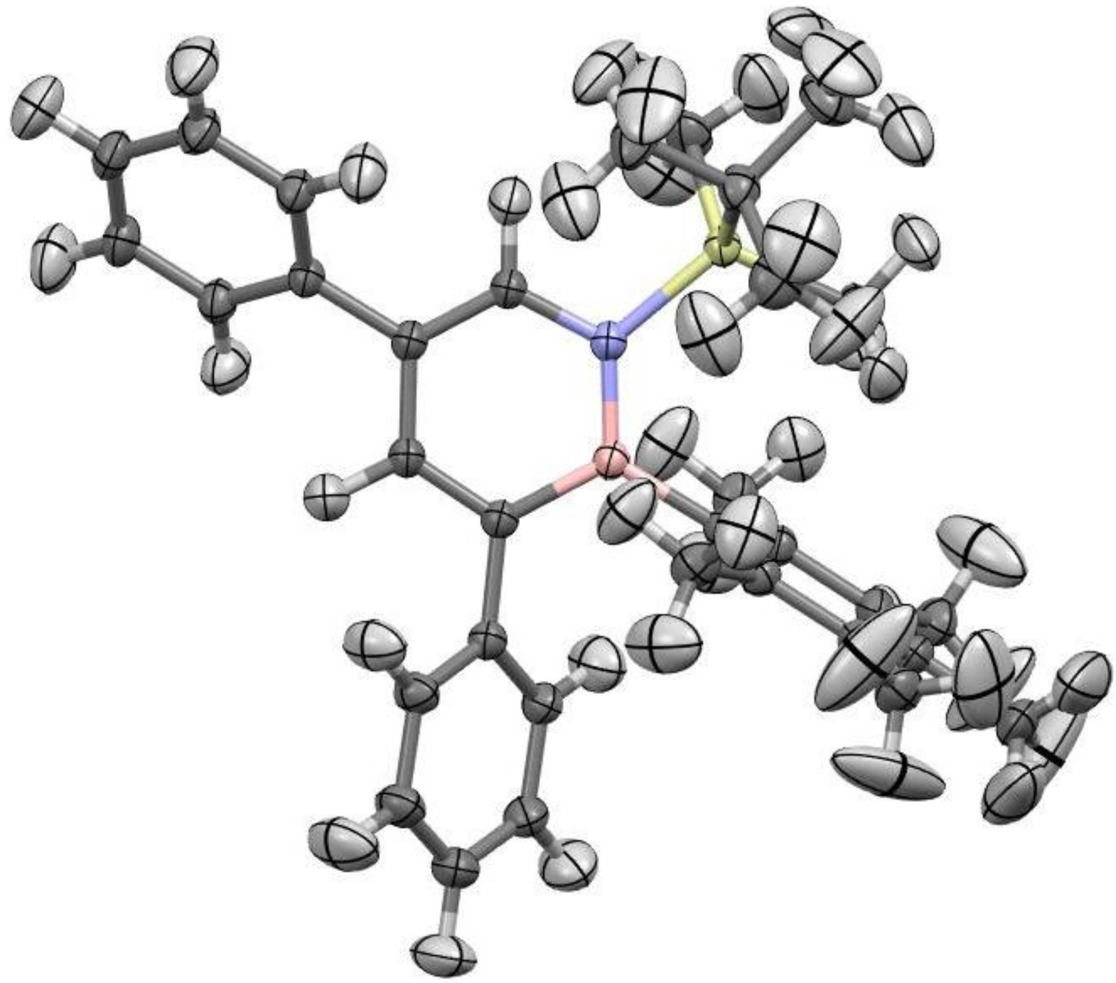

Figure S86. Crystal structure of ^{BN}**B3**$_{\mathbf{H}}$. Thermal ellipsoids are drawn at the 50% probability level.

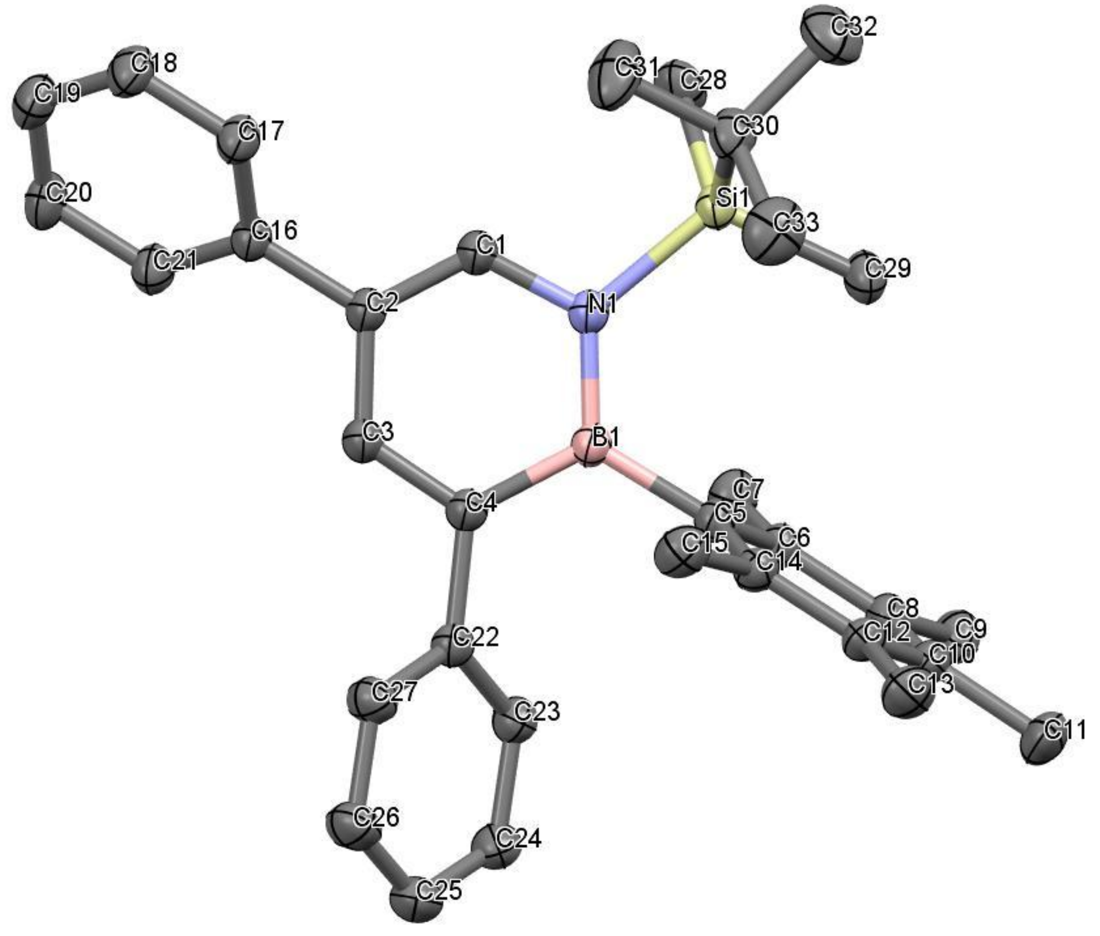

Figure S87. Crystal structure of ^{BN}**B3$_H$**. Hydrogens are omitted for clarity and thermal ellipsoids are drawn at the 50% probability level.

8. Computations

The computational investigation of the thermal back reaction of the benzvalene was performed in the Orca 6.0.1 software package.[3,4] The geometries were optimized using the PBEh-3c/def2-mSVP,[5] employing the finer grid DEFGRID3,[6] with subsequent frequency calculations at the same level of theory verifying minima and first order transition states. Guess structures for the transition states were taken from preliminary Nudged-Elastic-Band[7] computations at the GFN2-xTB[8] level of theory. Energies of the optimized structures were then recomputed at the higher level of theory revPBE0[9,10](D3BJ)[11]/def2-TZVP[12] with thermal Gibbs corrections at room temperature at the PBEh-3c level of theory.

Optimized geometry of the concerted transition state TS_{con}

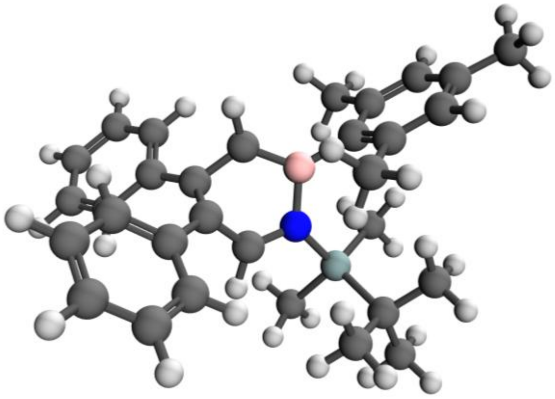

Figure S88. Optimized geometry of the concerted transition state TS_{con} connecting V3 and side product B4.

Arrhenius rate constant ratio of the left (concerted) and right (stepwise) pathways

Arrhenius equation for left and right pathway:

$$k_l = A_l \exp\left(\frac{-E_{a,l}}{RT}\right)$$
$$k_r = A_r \exp\left(\frac{-E_{a,r}}{RT}\right)$$

Assuming $A_l = A_r$ and E_a being independent of T:

$$\frac{k_r}{k_l} = \exp\left(\frac{-\Delta E_a}{RT}\right)$$

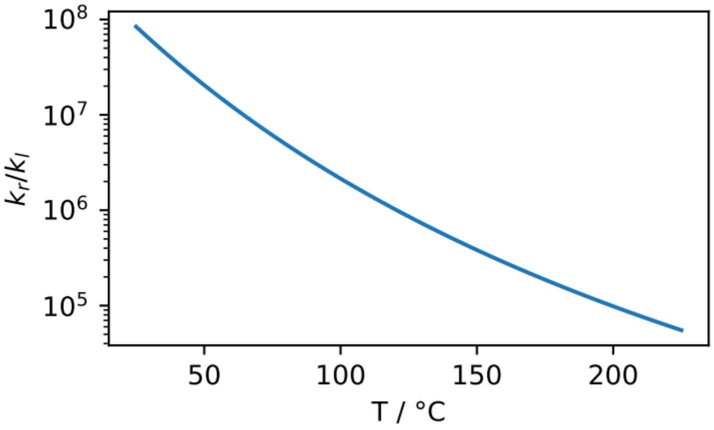

Figure S89. Plot of the temperature dependent Arrhenius rate constant ratio k_r/k_l of the left (concerted) and right (stepwise) pathways.)

Optimized structures (PBEh-3c/def2-mSVP) for the energy profile

B4

C	0.78370638607891	-0.66278025606179	0.97143603516696
N	-0.54346637401029	-0.37457182267227	0.82988247615230
C	0.17374810174154	1.07837319982494	-1.08420713880522
C	1.47883280417241	0.73209194095936	-0.86999774727177
C	1.79289053130946	-0.16993455491266	0.19921837832687
H	-0.03328099240558	1.79554268222377	-1.87209949600078
H	1.05354333910349	-1.35959497648987	1.75640256558125
B	-0.93938606653835	0.54861899482470	-0.20667839059841
Si	-1.61769977170903	-1.32259387465307	1.94341872932215

C	-0.85411856627803	-1.18078325554795	3.66239569421377
H	-0.65953268467247	-0.13658572016243	3.91394502598647
H	-1.54711377299208	-1.56707190536183	4.41168114527856
H	0.08244102682971	-1.72558161295597	3.78455624409523
C	-3.36987236375388	-0.66834864367973	2.04799827287032
H	-3.92006941536401	-0.70759149333421	1.10990126016973
H	-3.90724692694441	-1.27845749072055	2.77831290019207
H	-3.41145375247016	0.36023834815135	2.40158018378517
C	-1.65158496524865	-3.14321263169808	1.37367658275597
C	-0.25719456638146	-3.77229507287071	1.31999522563198
H	-0.33497511136545	-4.83238314880076	1.05919024597296
H	0.38169455678437	-3.30743868672538	0.56783341352150
H	0.26359188944280	-3.72087724400486	2.27903903714191
C	-2.28824488092395	-3.23782856789536	-0.01395431889320
H	-2.35920647278024	-4.28259246731101	-0.33247840535140
H	-3.29894021425921	-2.82514001550804	-0.03582484150510
H	-1.69952539059565	-2.71069457124150	-0.76622258334881
C	-2.50363729663399	-3.93289434937668	2.37577189408569
H	-3.53227298024075	-3.57146594223803	2.42621918514990
H	-2.55086444601145	-4.98595519271675	2.08207881298477
H	-2.08984847606775	-3.90292092083514	3.38623499453994
C	-2.41358941898446	1.07404480722796	-0.37814005835993
C	-2.85189086629697	2.13096843189240	0.43989307163690
C	-3.27249651099901	0.59559204308867	-1.36802469915197
C	-4.13153746333370	2.63958246092561	0.29377580461480
C	-4.55995800260178	1.12004266320314	-1.48615461240358
C	-5.01159277475016	2.13371267057640	-0.65991178834132
C	-2.85533654707392	-0.48204813340922	-2.33165585567770
H	-1.78975182503604	-0.70111846971310	-2.27356626937712
H	-3.39125054878012	-1.41534619852376	-2.14841641189213

H	-3.07035483875057	-0.18906569522720	-3.36045933318799
H	5.28054558889990	2.90046758109999	-3.95819970434707
C	4.51651151866736	2.45921141511992	-3.33176427700462
H	3.33097141911407	1.77138408493450	-4.98137526746626
H	5.47184016994910	3.01818305365273	-1.49430844188831
C	-6.39890692433922	2.69165467443863	-0.78902983640902
H	-6.38017975759038	3.76529844038113	-0.98266347369532
H	-6.94727123345483	2.22022017811949	-1.60377442243994
H	-6.97511461475036	2.54077600353000	0.12547415297144
H	-5.22000606067238	0.72917523077707	-2.25431066869650
H	1.59501198007593	0.76736552025801	-3.54901825786380
C	3.42344406476236	1.82776049671341	-3.90462281532668
H	3.74963436357172	2.00358613147257	-0.06984407120102
C	-1.93245094963953	2.71990524804064	1.47504506826368
H	-0.98806313429829	3.04355786404142	1.03371103090147
H	-2.38265392912322	3.58412188686736	1.96291912726790
H	-1.67964908368396	2.00047308416961	2.25768887380498
C	3.16646306121836	-0.63688196978069	0.48990150907336
C	3.69041379608478	-0.53372321119444	1.77682430738189
C	3.95660115332155	-1.21617319865903	-0.50246325217957
C	4.96391559216496	-1.00056445941867	2.06844387575802
H	3.09917377102673	-0.06365808038563	2.55352373611444
C	5.22955117365187	-1.67743900581895	-0.21454579059895
H	3.56861078534822	-1.30815703497982	-1.50859561129490
C	5.73916144513895	-1.57227910890224	1.07245781142422
H	5.35307402727661	-0.90695112185946	3.07400283337499
H	5.82661541556969	-2.12406399317082	-0.99877401506508
H	6.73510761558308	-1.93176941819979	1.29522987786214
C	2.54917921999199	1.31477717514680	-1.71143945329110
C	3.65329911806017	1.94982412827719	-1.14627622387087

C	2.44519412807172	1.26499919378133	-3.09929120591622
C	4.62473695839366	2.52245098753889	-1.94979075065974
H	-4.45533502952165	3.45443289579971	0.93359010592458

TS$_{con}$

C	0.98548775375242	-0.62328440676864	0.54964893270332
N	-0.30996913154620	-0.32566688756398	0.73446780056638
C	0.42777910827304	1.34738717955148	-0.80117967926048
C	1.77886031914868	1.12201741256738	-0.24762399924130
C	1.79464396827344	-0.21140420607491	-0.59338582280386
H	0.45371336019662	1.80077812487901	-1.79028508830039
H	1.52299969104959	-1.18242851349855	1.31249471922558
B	-0.76853873640149	0.65859983907459	-0.26730935833993
Si	-1.14434959270115	-0.90823741136773	2.21481355139464
C	0.13717804066621	-0.86161245736846	3.59770234672238
H	0.67163425268703	0.08961468026317	3.60312784060022
H	-0.35060030453724	-0.96651866860264	4.56816887526009
H	0.88111004441166	-1.65737385203050	3.53098045723767
C	-2.56083944685920	0.23260351650773	2.65546808261327
H	-3.27834775575713	0.35179690270267	1.84474249587954
H	-3.09776930813200	-0.17013968334156	3.51732845931715
H	-2.20333830684350	1.22352145406821	2.93573805034716
C	-1.77422122491200	-2.69233946796615	1.99133533721006
C	-0.72198121081844	-3.55367116835601	1.29014758868934
H	-1.06282472830922	-4.59106749078290	1.21587974596439
H	-0.52097605732935	-3.20800323372631	0.27438498834863
H	0.22881186242346	-3.56977312276256	1.83013326469601
C	-3.07519580622392	-2.70649804192485	1.18474305192823
H	-3.41879940399390	-3.73471412898513	1.03299844592710
H	-3.87631018757957	-2.17195302544877	1.69797643797125

H	-2.96833624939397	-2.25020750126311	0.20088338739980
C	-2.05129607730121	-3.28034157752738	3.38058029808872
H	-2.77543014155491	-2.68778556632672	3.94508336085562
H	-2.46899747285255	-4.28776873197667	3.28940816708093
H	-1.14695555373697	-3.36049769396454	3.98661096578688
C	-2.24604363626767	0.75951967132658	-0.78915876163665
C	-3.06828424096568	1.85102062939599	-0.47745988071418
C	-2.75167912671071	-0.23540390103822	-1.63919096671285
C	-4.37850181788837	1.89183543479069	-0.94088083870509
C	-4.06366364824384	-0.16644283474712	-2.09429774400352
C	-4.90046108659738	0.88271788383609	-1.73934859916375
C	-1.86130008629087	-1.34374705517494	-2.13216321498318
H	-1.32793707464736	-1.84286748623993	-1.32354318062840
H	-2.42671162139670	-2.10183602374330	-2.67418696059030
H	-1.09734981914507	-0.95326108914938	-2.80720944836073
H	5.29286134118299	4.70612011183947	1.49382209314978
C	4.58213994396328	3.97200119993856	1.13830243121260
H	3.03959462638413	5.41197557033571	0.75886403212326
H	5.91271348301621	2.31168167668164	1.42580844929690
C	-6.32992936418501	0.91973875094574	-2.19558868004643
H	-6.45872731860430	0.42108107848821	-3.15617790393302
H	-6.98485662344567	0.41713585420500	-1.48052665052036
H	-6.69232568132796	1.94224444421680	-2.30112065878063
H	-4.43970877774940	-0.94672091655538	-2.74819448705216
H	1.42128896178077	3.73046660013023	-0.05890036080643
C	3.31795694097769	4.36674359072614	0.72458847399604
H	4.30322942423530	0.63765317345686	0.62444787195646
C	-2.53875832774390	3.00807436385090	0.32356629560941
H	-1.84307531811426	3.60240028811127	-0.27162897614801
H	-3.34149102805619	3.66857573981769	0.65211345676018

H	-1.98780455193017	2.68446660912082	1.20546083918800
C	2.74652554082391	-1.05510881560949	-1.29138509814333
C	2.62515514440563	-2.44389313984698	-1.22811021656551
C	3.76301808790731	-0.49674166222533	-2.07027536892796
C	3.52294124418657	-3.25813471800882	-1.89687670086436
H	1.81647469585230	-2.88190566567206	-0.65479016708868
C	4.65106682707073	-1.31173897438494	-2.74688588016089
H	3.84807690655553	0.58036735887218	-2.14193445690033
C	4.53778378897682	-2.69421386815846	-2.65692212031514
H	3.42480180999455	-4.33392977756034	-1.83670637358371
H	5.43561620945019	-0.87138646513075	-3.34775938411158
H	5.23481327514523	-3.32988448089829	-3.18677296239254
C	2.75187675744901	2.07364387250971	0.21168355021122
C	4.02867178958166	1.68523244909109	0.64038910922338
C	2.40917426920588	3.42852191029387	0.26648601113849
C	4.93093956275902	2.62579534057086	1.09624921492291
H	-5.00706318569234	2.73660099960548	-0.67804849081737

V3

C	-1.45945925990022	1.04024487375385	0.45945246920768
C	-2.65837493013154	1.38200484985646	1.21707901767843
C	-3.65506832812697	0.42925531580366	1.41090773973065
C	-4.80512390651529	0.74623696889780	2.11743860276368
C	-4.96930841240432	2.01720046146106	2.64677068482966
C	-3.97852080427486	2.97180329350929	2.46018623605673
C	-2.83334991474988	2.65936516600818	1.74753105501543
C	0.04107312234330	1.27789602983633	0.66077540119155
C	-0.69616420486681	1.69128634640222	-0.59299457512006
C	-1.17570928772811	0.28490086147580	-0.78419910857194
N	-0.09658648144288	-0.69843178069053	-0.62190668804381

Si	-0.28829617981409	-2.20949345364027	-1.52308369172974
C	-1.83773105408672	-3.04002508727949	-0.83196258666561
C	-0.61342309414316	-1.72334203324112	-3.31937219446689
C	1.16895335573189	-3.43695246647149	-1.48816765256090
C	1.36889737479568	-4.06828892690005	-0.10938876315013
C	0.80971774552490	-4.55652691034039	-2.47871924482106
C	2.47544762241970	-2.78844393323171	-1.94826450867985
B	0.73443130078021	-0.10656982163600	0.35948060622152
C	2.02218508210902	-0.63542841687645	1.06323376441785
C	3.28917709160750	-0.27029762687247	0.59988420220342
C	3.42402225249278	0.62674167640857	-0.59973654009131
C	4.42363833334076	-0.71056861337373	1.27194519011462
C	4.33188338569648	-1.49126170673505	2.41619509169959
C	5.56350437981718	-1.99163983370946	3.11297850061065
C	3.06579710089550	-1.81182506946483	2.89208888131191
C	1.91609730369756	-1.39176233428593	2.23672555338250
C	0.56001898278632	-1.74351998686884	2.78320880565157
C	-0.81574952647372	2.95482228955503	-1.31362515003526
C	-2.02295569833323	3.33256365633984	-1.90032191002239
C	-2.12630608713614	4.53126708458858	-2.58538089382061
C	-1.02918427950961	5.37608414848839	-2.68620611306033
C	0.17251471736691	5.01188145079780	-2.09849225259378
C	0.27939088744775	3.80742276257646	-1.41993248832696
H	-3.52128068609689	-0.56711589023537	1.00768270893141
H	-5.57226769089564	-0.00373849963366	2.25941782172417
H	-5.86497794646764	2.26451997996744	3.20099795624587
H	-4.10268831830975	3.96739357629963	2.86561049432773
H	-2.07194294666061	3.41365867683891	1.59110112918795
H	1.22070443137457	3.51804581110754	-0.96948123899371
H	-1.97240401699751	0.07816299228793	-1.49056496087207

H	-2.70763702286913	-2.38523981413580	-0.91699399705884
H	-2.08474125150180	-3.96369518837797	-1.35731396139249
H	-1.72292069645963	-3.28259452624591	0.22596781580630
H	0.28156841752033	-1.31393700077248	-3.79075946596665
H	-0.93463325503750	-2.58048829470434	-3.91248632568819
H	-1.39281054541382	-0.96644162607455	-3.41489540995891
H	0.46092543907192	-4.55014767113229	0.25991370905001
H	2.14268296923107	-4.84099541935127	-0.15906567662129
H	1.69532180997518	-3.34217145485207	0.63308474666592
H	0.72867386384585	-4.19777583311228	-3.50593876833311
H	1.59280609093707	-5.32064179306752	-2.46853932514778
H	-0.12617432133818	-5.05972505165628	-2.22600296637423
H	2.83789601435676	-2.05270830920160	-1.23264812988077
H	3.25797086535254	-3.54713172859693	-2.04981603640163
H	2.37735517928430	-2.29889353097368	-2.91981428914993
H	3.16365719023940	1.65747032999041	-0.34595210028874
H	4.44303494651843	0.63655240386950	-0.98571874954108
H	2.76558305361403	0.32556310726620	-1.41612438813884
H	5.40293362574800	-0.43243264044850	0.89645764490432
H	6.42918454451667	-1.36517018466361	2.89966390129674
H	5.43102826634135	-2.01732180042827	4.19466296994186
H	5.81104229178983	-3.00631206251263	2.79415661297184
H	2.97318922255996	-2.40365620150356	3.79688156421799
H	-0.03509901057741	-0.85031302325432	2.98496366769236
H	-0.01258509453478	-2.35188158742492	2.07971676282019
H	0.63406315019507	-2.30436552382714	3.71435312960319
H	-2.88917962764913	2.68886925214489	-1.80718130082690
H	-3.06966588300373	4.81255786498534	-3.03474152464319
H	-1.11283956229765	6.31460338472479	-3.21793293927536
H	1.03207619699862	5.66523047404358	-2.17185742591848

H	0.34769935718860	2.04688357051669	1.36231537196734

TS1

C	-1.70468562949896	0.58473370556101	0.85877134340662
C	-2.97943199470829	1.16390484200308	1.30460575369608
C	-4.17414810141772	0.52111702956855	0.99099023852292
C	-5.38806278972160	1.04315990825927	1.41017921609066
C	-5.42114580722944	2.21192533413329	2.15403672319935
C	-4.23387134072122	2.85322802244298	2.47908028796789
C	-3.02119357099633	2.33496212949281	2.05883676763656
C	-0.40303266317071	0.66150448281759	1.50406793277447
C	-0.52441426462222	1.44215187998313	0.18056778328786
C	-1.49951070472531	-0.12316612299692	-0.36295990612575
N	-0.36643104047807	-0.85457127314250	-0.47611323792435
Si	-0.15135429856279	-2.34373196935875	-1.36621384302025
C	-1.07577417514256	-2.17701157252595	-3.00284981632320
C	1.67936057813693	-2.61260216330546	-1.66110010195284
C	-0.88616771606510	-3.79097089991318	-0.36501776793447
C	-2.37816882381793	-3.55215849131139	-0.12513646558714
C	-0.70222978447446	-5.10495417229351	-1.12882014239035
C	-0.17433827351537	-3.88516898294500	0.98606133036582
B	0.50231211893994	0.13473562502665	0.33795011279539
C	2.06758154300309	0.20202589767709	0.23175359534058
C	2.67782327545813	0.69956108958840	-0.92996483077209
C	1.85792189709125	1.20884579690321	-2.08332032055971
C	4.06420425056772	0.72802749336336	-1.03233777093395
C	4.87996359522056	0.26787088950686	-0.00854848971455
C	6.37529878428452	0.33092053689351	-0.11981474637094
C	4.27139072536126	-0.23621393608599	1.13260963279030
C	2.88791427178780	-0.27364255754015	1.26574122531779

C	2.30000550052779	-0.83774965070477	2.53025828319736
C	-0.56624362093892	2.88250571462539	-0.02730409267310
C	0.51533161192360	3.65753636536787	0.39920409200328
C	0.53116537844197	5.02460124738337	0.17896002690441
C	-0.52971955802674	5.64485856727600	-0.46643318695950
C	-1.60426519518298	4.88121795553046	-0.89686811598565
C	-1.62226694864703	3.51188581111105	-0.68502362527063
H	-4.15812358394771	-0.40762721603343	0.43332224468853
H	-6.30758485646048	0.53000234688174	1.16200371476080
H	-6.36721204278249	2.62014189100022	2.48376573780856
H	-4.25205554095306	3.76641962774739	3.05872738727192
H	-2.10474603578398	2.85404506616223	2.30652820769420
H	-2.46478890068213	2.92718213475250	-1.03227383878184
H	-2.27072614810922	-0.12264621032288	-1.13242893851705
H	-0.66912307681094	-1.35382329400815	-3.59253223976942
H	-0.98524144959821	-3.08200236014915	-3.60582219067161
H	-2.14334602158739	-1.98990549740708	-2.87301281755205
H	2.25330933426375	-2.60900054584907	-0.73399949116174
H	1.85316916663843	-3.56980207550311	-2.15640394791566
H	2.10497030808178	-1.83366893443423	-2.29366403236463
H	-2.94729063710718	-3.51499132245665	-1.05729182122079
H	-2.80665955414205	-4.36052690146418	0.47602918026580
H	-2.56269881592698	-2.62313513063829	0.42119660455211
H	0.35125379772182	-5.33228061236444	-1.30360366289722
H	-1.12122609270870	-5.94372734633447	-0.56389699390164
H	-1.20280920556214	-5.09145508036850	-2.09947610922716
H	-0.28920047935163	-2.96979534669197	1.57092459250489
H	-0.58602877521191	-4.70566909245268	1.58270151090408
H	0.89557480238567	-4.07385614882675	0.87447587933449
H	1.04797483279359	0.52593995576311	-2.34029836244080

H	1.39606060603584	2.16931857007489	-1.85165584643891
H	2.47252883390268	1.34630401249725	-2.97304621083128
H	4.51990139362102	1.11269601717523	-1.93895379018864
H	6.76634481102038	1.23537930033214	0.35103420359109
H	6.85124277517017	-0.51835262128391	0.37066907767658
H	6.70237756683883	0.34054274496959	-1.15936584768207
H	4.89129876067972	-0.61557313403932	1.93857636900413
H	1.86067203015718	-0.05612401691330	3.15228230838008
H	1.50709771218835	-1.55729504396802	2.32803189637689
H	3.05996244290032	-1.33953768344812	3.12902462404748
H	1.35171556744592	3.17668561934831	0.89192650424751
H	1.37940248782079	5.60830394903620	0.51228529990941
H	-0.51674709753360	6.71327492289946	-0.63560115558023
H	-2.43564068868859	5.35299235760208	-1.40431224735201
H	-0.27742545579869	1.19222856632399	2.43828631667733

I

C	-1.84558082628462	0.66537645097327	0.62480378288320
C	-3.11149886613557	1.29583040414804	1.06256453487987
C	-4.32612418327013	0.99494847984008	0.44921723956056
C	-5.50431236173523	1.60225901215258	0.85591528126337
C	-5.49234265539078	2.53474751009723	1.88024527498199
C	-4.28782282426817	2.85429083026795	2.48902614251923
C	-3.11314592069463	2.24288787634195	2.08572546025673
C	-0.58196561622336	0.62656736163688	1.51510503745898
C	-0.57883985577758	1.49871036629882	0.35578692941729
C	-1.79783700634920	-0.50319656916428	-0.23642241176950
N	-0.58910997587140	-0.87848064149568	-0.48699585575030
Si	-0.21091403499757	-2.39954776677899	-1.38346963847706
C	-1.30317953701145	-2.39051072221225	-2.91547360536474

C	1.58929211530256	-2.43123596125818	-1.88095909284033
C	-0.62741710466493	-3.86942338575389	-0.24898843837675
C	-2.12810425610382	-3.94883462074132	0.03909135444983
C	-0.18952017839273	-5.15687985011532	-0.95766264847209
C	0.12787660464527	-3.73445492942416	1.07463330826279
B	0.31500578214319	0.16955358117276	0.29467608884591
C	1.88469139251870	0.17157641562140	0.16401512787930
C	2.47711833758899	0.67407740040101	-1.00906618875350
C	1.63909659490679	1.24170492134488	-2.12229456257334
C	3.85728134976296	0.66616984239495	-1.15761746808344
C	4.69463110836411	0.17817302037487	-0.16198271389906
C	6.18514427074858	0.19158847507468	-0.33816874690026
C	4.11022925258341	-0.29806223483385	1.00071405399692
C	2.72830720028190	-0.30070836747660	1.17859267761823
C	2.18285258957234	-0.79063981687227	2.49180464774458
C	-0.45790655516134	2.94450336183768	0.20665617449671
C	0.63988476820137	3.60547010516396	0.76051716751278
C	0.80611485047700	4.96886489695210	0.58295827717057
C	-0.12772113888027	5.70112512542720	-0.13708017193899
C	-1.22432378664878	5.05356778751913	-0.68632098210184
C	-1.38455048849993	3.68643485454209	-0.52391611637987
H	-4.36896841590952	0.28616198700301	-0.36825925566095
H	-6.43367682057778	1.34746131566803	0.36342369446227
H	-6.40994117318913	3.01227131845480	2.19679129215614
H	-4.25945502478988	3.58879061383553	3.28305469379341
H	-2.18380501228459	2.51921395163487	2.56586115875826
H	-2.24135222782879	3.18871321226078	-0.95967167014390
H	-2.66396737345871	-1.02898145515203	-0.62535848591485
H	-1.08342652167512	-1.52323833255100	-3.54003789930430
H	-1.12391978068744	-3.27878851241801	-3.52364346164642

H	-2.37028107446387	-2.36937810457127	-2.69006185594950
H	2.26859487836240	-2.41035888738446	-1.02952236860834
H	1.78750211066000	-3.34236095568095	-2.44948413649983
H	1.85391707556427	-1.58913718648097	-2.51858889963119
H	-2.72436771655956	-4.04435759757767	-0.87099845689657
H	-2.34826539308633	-4.82416504410308	0.65757377169282
H	-2.49127032471080	-3.08038642213315	0.59267220041635
H	0.88625199184689	-5.18367779228227	-1.13889283851695
H	-0.43153224567494	-6.02835633546842	-0.34205974748352
H	-0.69248573897534	-5.29642515558153	-1.91760165151826
H	-0.19335295878199	-2.85406340673166	1.63399457245821
H	-0.05499353261571	-4.60652697451487	1.70997744960908
H	1.20793709319072	-3.65868253613324	0.93322968149410
H	0.80866398453933	0.59073811968732	-2.39807527696482
H	1.19747803128244	2.19694165358000	-1.83515642562420
H	2.23716317706002	1.41145899322581	-3.01795223278545
H	4.29362381390138	1.05026123152704	-2.07429340434344
H	6.58281460642614	1.20536070356857	-0.25951163497219
H	6.68500781746819	-0.41355789093980	0.41754996124082
H	6.47722762323919	-0.19201034125357	-1.31648538331646
H	4.74586431595065	-0.67806160020739	1.79399263098451
H	1.87523012200735	0.04414073774854	3.12526197816464
H	1.30749540818202	-1.42354614895691	2.36713626270942
H	2.93278046751285	-1.35539282671016	3.04611998991367
H	1.37449332310470	3.03820669695466	1.31913157921192
H	1.66812973504852	5.46299964196323	1.01236269400598
H	-0.00094099364529	6.76757552904495	-0.26899503631910
H	-1.95969282138488	5.61513280297937	-1.24799437782010
H	-0.56986946976763	0.89234178424160	2.56601096932542

TS2

C	-1.89116180751842	0.07393005146784	0.44254648555330
C	-3.19799567538308	0.64267155038020	0.76670962446060
C	-4.17527575681201	0.81719490413098	-0.21712211525146
C	-5.42632320841586	1.31709595220687	0.10037716059678
C	-5.72673227027921	1.67622748351735	1.40692156908692
C	-4.76316047661633	1.52333944998184	2.39223422441534
C	-3.51563243577888	1.01028375298230	2.07627192433255
C	-0.63300045187926	0.47565317120454	1.17928157302618
C	-0.24932349215625	1.34481092650661	0.14973027341812
C	-1.65290125823062	-0.85965788349436	-0.52772904415325
N	-0.34715950310897	-1.13588660123667	-0.74058926584498
Si	0.11180216593803	-2.77028813713315	-1.28548610457092
C	-0.69116794142786	-3.03359033814005	-2.96909965929622
C	1.96912575238806	-2.97889543919394	-1.43289981115258
C	-0.54329333829377	-4.05254207876279	-0.02784018969716
C	-2.07198667469443	-4.13562290611103	-0.03807749432527
C	0.01930893194793	-5.43360138716004	-0.38258057577502
C	-0.07459463790563	-3.66069622680834	1.37582280039060
B	0.43452670191201	-0.09090971915538	-0.02066981733705
C	2.01263888516928	-0.02758300826451	-0.05275738497032
C	2.64711539063258	0.27565609924814	-1.26722190783990
C	1.87647611170483	0.47225050559546	-2.54742764918268
C	4.03474625274494	0.36669324830175	-1.32951174784913
C	4.82858363263658	0.16186358813576	-0.21298465015423
C	6.32347677311714	0.27443922685787	-0.28406625908284
C	4.19592244211211	-0.13522615528685	0.98840589698513
C	2.81372669751482	-0.22393499894495	1.08466945739268
C	2.20312870220432	-0.53222484085444	2.42384778977735
C	-0.06713812775168	2.76916930868594	0.17182502706908

C	-0.78429448750889	3.55027486624286	1.08657127231737
C	-0.64439693675422	4.92599917639228	1.09735597163926
C	0.23935248795756	5.53829553865148	0.21865571669516
C	0.96657526597307	4.77455502260318	-0.68625982865362
C	0.79853847300216	3.40324646128359	-0.72614179415870
H	-3.94053771178949	0.58070090536422	-1.24718558066296
H	-6.16573264216314	1.44383079715135	-0.67963623526099
H	-6.70110379446611	2.07657022371539	1.65320227488838
H	-4.98606009449911	1.79650867795505	3.41549231011821
H	-2.78473924130898	0.87416398900180	2.86347231797125
H	1.35386808216087	2.80477832245541	-1.43321753639284
H	-2.43269228128279	-1.35325886180471	-1.09652045882524
H	-0.27470917024698	-2.34421026002641	-3.70511282077935
H	-0.51631682935247	-4.04659992516682	-3.33559235625803
H	-1.77114727316398	-2.87952971409890	-2.95600025373078
H	2.48865378357443	-2.84876024516460	-0.48396248545825
H	2.17490228103521	-3.99062248992025	-1.78942793124106
H	2.42751539941519	-2.29136264509630	-2.14039575740967
H	-2.46918287890743	-4.37660319146176	-1.02689810049029
H	-2.41285758193523	-4.92153487652454	0.64350550619681
H	-2.54110283901039	-3.20860275442403	0.29464909571157
H	1.10762326411663	-5.47022495442783	-0.31270913200745
H	-0.36993271003365	-6.18916219148104	0.30687196027236
H	-0.25824889441111	-5.75139595864986	-1.39082371505302
H	-0.47268412403417	-2.68972097550331	1.67837399389850
H	-0.40946624424102	-4.39670600747188	2.11367606725950
H	1.01450648976416	-3.60825484478741	1.44514341391988
H	0.83896688248125	0.75393334561046	-2.38000900221105
H	2.34114476313910	1.23746938827007	-3.17176675247157
H	1.85936240760644	-0.44353708238673	-3.14442876704897

H	4.50733378393241	0.60110682597012	-2.27835838500137
H	6.67499416054865	0.32138167838174	-1.31426165376521
H	6.67698819973648	1.17387083656681	0.22361679975827
H	6.81199663650113	-0.57603530280801	0.19297105894832
H	4.79890373701142	-0.30038687338068	1.87562967681190
H	1.68407369023726	0.33556574670185	2.83484677512345
H	1.47262102819472	-1.33826480749758	2.36583689365465
H	2.96609108168872	-0.82296685564769	3.14597468363205
H	-1.47334814526257	3.07050623882005	1.77035383810513
H	-1.21626681402620	5.52312772560354	1.79505160021733
H	0.36004883310551	6.61360704946565	0.23652064526591
H	1.65625828908984	5.25391689759588	-1.36800871145501
H	-0.63307970964464	0.70900160527061	2.24290525590860

B3

C	-2.27249014815023	0.75501046057156	-0.57746432973793
C	-3.69248973623933	1.05960549890593	-0.82986339676782
C	-4.38322867885010	1.95992822174258	-0.01873842291418
C	-5.72058866639355	2.24125021500244	-0.24501771119783
C	-6.39866434034043	1.62404755583483	-1.28587901877212
C	-5.72397025701521	0.72867106469320	-2.10179309242435
C	-4.38361890085558	0.45543383372864	-1.87989322826784
C	-1.34948967479364	1.77892475922670	-0.24147729305122
C	-0.01923889292089	1.53365190690285	-0.04666651065894
C	0.86012406381952	2.65508785177102	0.33958210520766
C	0.47864951562110	3.52226242912924	1.36538019547901
C	1.28820195522444	4.57841035095585	1.75285391058355
C	2.50416291529003	4.79016194813710	1.12120263089826
C	2.89718377489763	3.93769193510388	0.09971920827302
C	2.08704757150726	2.88176301178935	-0.28493492895453

B	0.47640816285922	0.09647273954919	-0.20226051345430
C	2.00827045906150	-0.26042762971002	-0.17890924627117
C	2.66256956315669	-0.51089071104385	-1.39631502776581
C	1.90207957860188	-0.47856412133843	-2.69514594100233
C	4.02350386502583	-0.77646210825548	-1.41306263462196
C	4.77215493149314	-0.80730210741618	-0.24094602281065
C	6.23513111638151	-1.14019809934553	-0.27497600198538
C	4.12475611369762	-0.52760917117520	0.95174501467739
C	2.76349295408600	-0.23644159043276	0.99606387301143
C	2.15408295515890	0.15801878934601	2.31165281774883
N	-0.53170794883406	-0.91004162588783	-0.44020017582342
Si	-0.33384220134793	-2.71259322688755	-0.30063980760684
C	-1.23195400790180	-3.46649216039645	-1.77819089602407
C	1.43767622965930	-3.31779139690015	-0.35809521935071
C	-1.13304460731492	-3.27428499163454	1.34016709213434
C	-2.64050401738701	-3.01151258412130	1.38818578669274
C	-0.90005664756505	-4.78434682116560	1.48219176769196
C	-0.46860689232277	-2.55030508170727	2.51288356530993
C	-1.82780022129829	-0.53105630684528	-0.64195982622376
H	-3.87332206333896	2.43010905109551	0.81305607662374
H	-6.23759727014904	2.93954923540547	0.40012747632015
H	-7.44335167698983	1.84298049690753	-1.46231852229374
H	-6.23881875841239	0.25137960276473	-2.92540638587133
H	-3.85756157506179	-0.21598786732198	-2.54740566811838
H	-1.72588070318775	2.79716442617328	-0.18436702340336
H	-0.45660293916370	3.34859042368937	1.88402945859122
H	0.97181497493899	5.23115409040932	2.55626159306249
H	3.14149772992947	5.61066011159814	1.42374190270998
H	3.84268774049979	4.09408184089261	-0.40303881106535
H	2.41108231394337	2.23059460216956	-1.08478537527415

H	1.29867535163452	0.42643314214243	-2.79139487160249
H	2.57627910300725	-0.51585935786867	-3.55053682792402
H	1.21337691774731	-1.32142195060756	-2.79040003378295
H	4.51619216833013	-0.96435088632385	-2.36178339724167
H	6.73598025344861	-0.84577222182927	0.64665012194439
H	6.39291940352559	-2.21303628598884	-0.40315695674203
H	6.74116701087115	-0.64040585669677	-1.10150488600746
H	4.69587726523378	-0.51887254700341	1.87452618571635
H	2.27811770278525	1.23026626043862	2.47909410757550
H	1.08596163100716	-0.04716315182878	2.35839915411005
H	2.62659246912422	-0.36057615746008	3.14673708655453
H	-0.87235034511323	-3.03093511347304	-2.71229962976151
H	-1.02958181666738	-4.53788218212753	-1.82695366477962
H	-2.31556053823260	-3.34922675525112	-1.76004228186411
H	2.05426346034850	-2.97303306659497	0.46994801491748
H	1.40887189045106	-4.40973101218195	-0.32384252561812
H	1.95055670886131	-3.04168704366644	-1.27690911610407
H	-3.17463023514471	-3.48558554425638	0.56160095246401
H	-3.06154208344694	-3.41907543432921	2.31254109103677
H	-2.88005371492316	-1.94743367507945	1.37563590548048
H	0.15914617656611	-5.03894341739532	1.54181573314489
H	-1.36843334050264	-5.15214894072792	2.40012031888952
H	-1.33365089614274	-5.35207633300193	0.65537497353195
H	-0.66327390117270	-1.47676346109163	2.48579349498975
H	-0.85928419714921	-2.92382887557040	3.46456775971844
H	0.61438807234323	-2.68978318747972	2.53214020315026
H	-2.54563217580884	-1.31451579665717	-0.85369435509860

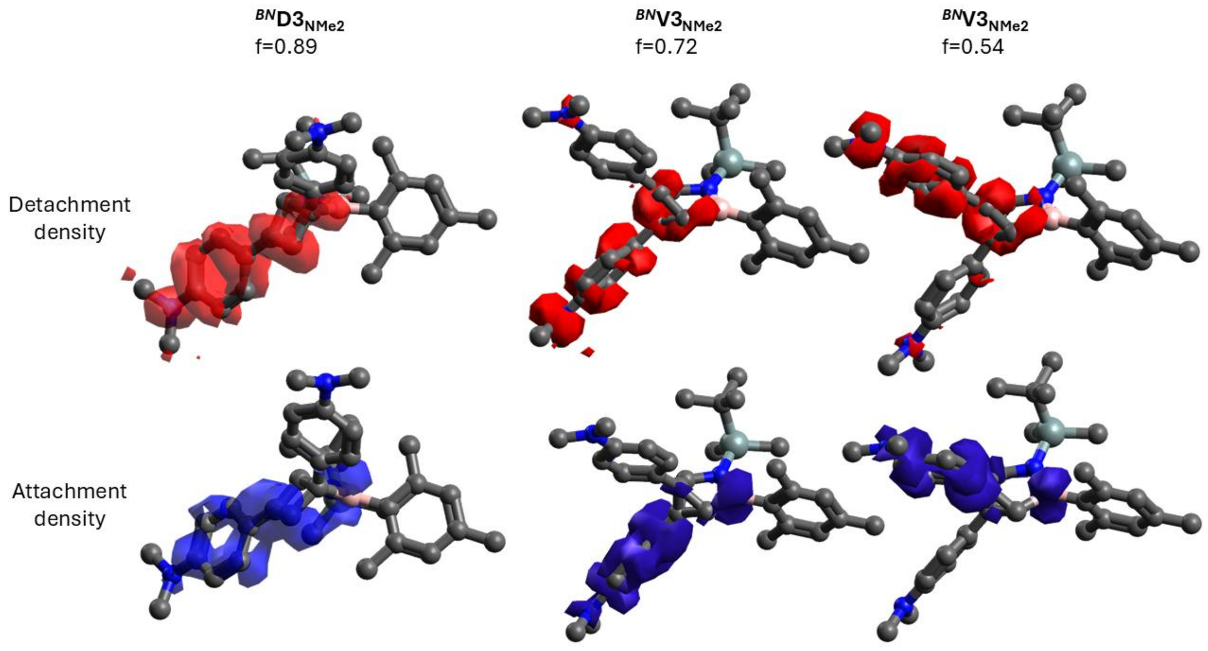

Figure S90: Attachment and detachment densities of the relevant excited states for ^{BN}**D**$_{\mathbf{NMe2}}$ (one excited state, left) and ^{BN}**V**$_{\mathbf{NMe2}}$ (two excited states, on the right) showing CT character from the Ar substituents to the boron atom. Hydrogens omitted for clarity.

9. Ultrafast Spectroscopy

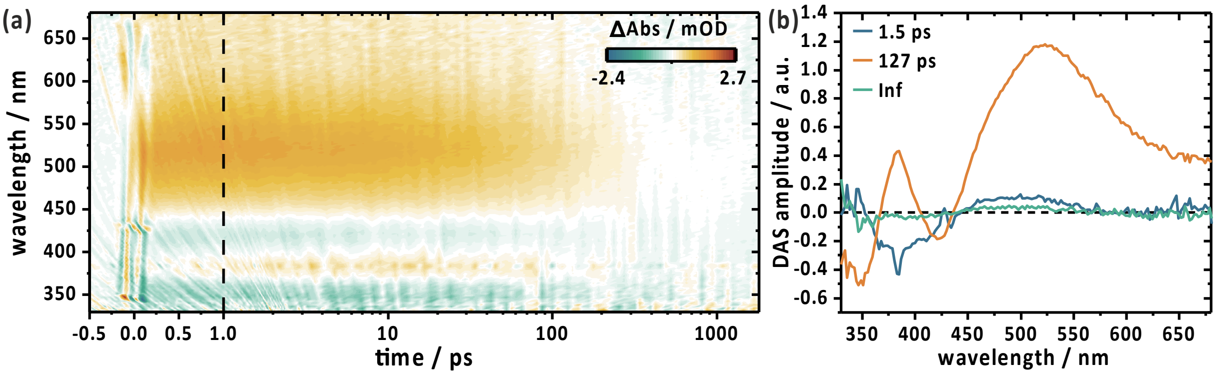

Figure 91: (a) Transient absorption difference spectra of $^{BN}\mathbf{B3}_{\mathbf{NMe2}}$ excited with 386 nm in reference to the ground state absorption. (b) Decay associated spectra (DAS) describing the kinetics of (a). Positive DAS amplitudes account for decay of positive or rise of negative absorption whereas negative DAS amplitudes indicate rise of positive or decay of negative signals.

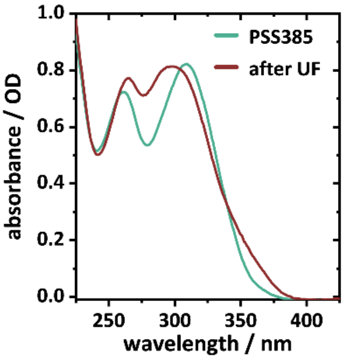

Figure 92: Steady State absorption spectra before and after transient absorption measurements with 332 nm. $^{BN}\mathbf{D3}_{\mathbf{NMe2}}$ is prepared by illuminating $^{BN}\mathbf{B3}_{\mathbf{NMe2}}$ with a 385 nm LED.

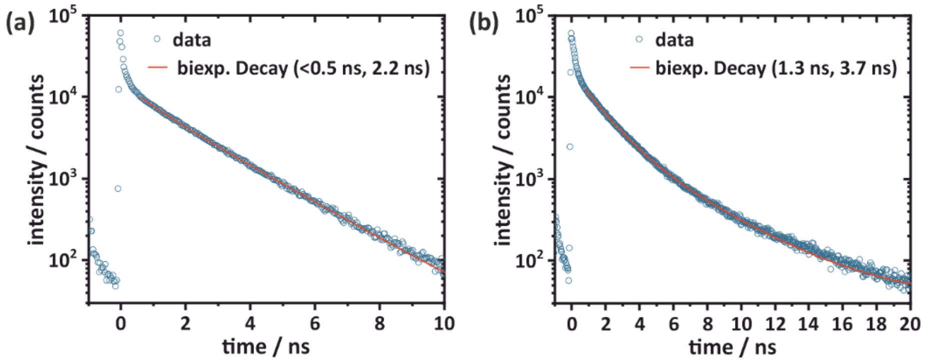

Figure 93: (a) Time-correlated single photon counting (TCSPC) measurements of $^{BN}\mathbf{D3}_{\mathbf{NMe2}}$ (prepared from $^{BN}\mathbf{B3}_{\mathbf{NMe2}}$ with a 385 nm LED), excited with 365 nm. The tail was fitted biexponentially with a lifetime below the time resolution (<0.5 ns) and 2.2 ns. (b) Time-correlated single photon counting (TCSPC) measurements of $^{BN}\mathbf{V3}_{\mathbf{NMe2}}$ (prepared from $^{BN}\mathbf{B3}_{\mathbf{NMe2}}$ with a 365 nm LED), excited with 280 nm. The tail was fitted biexponentially with lifetimes of 1.3 ns and 3.7 ns.

References

(1) Sheldrick, G. Crystal structure refinement with SHELXL. *Acta Crystallographica Section C* **2015**, *71* (1), 3-8. DOI: doi:10.1107/S2053229614024218.
(2) Dolomanov, O.; Bourhis, L.; Gildea, R.; Howard, J.; Puschmann, H. OLEX2: A complete structure solution, refinement and analysis program. *J. Appl. Cryst. J. Appl. Cryst* **2009**, *42*, 339-341. DOI: 10.1107/S0021889808042726.
(3) Bourhis, L. J.; Dolomanov, O. V.; Gildea, R. J.; Howard, J. A.; Puschmann, H. The anatomy of a comprehensive constrained, restrained refinement program for the modern computing environment - Olex2 dissected. *Acta Crystallogr A Found Adv* **2015**, *71* (Pt 1), 59-75. DOI: 10.1107/s2053273314022207 From NLM.
(4) Richter, R. C.; Biebl, S. M.; Einholz, R.; Walz, J.; Maichle-Mössmer, C.; Ströbele, M.; Bettinger, H. F.; Fleischer, I. Inside Cover: Facile Energy Release from Substituted Dewar Isomers of 1,2-Dihydro-1,2-Azaborinines Catalyzed by Coinage Metal Lewis Acids (Angew. Chem. Int. Ed. 30/2024). *Angewandte Chemie International Edition* **2024**, *63* (30), e202411078. DOI: https://doi.org/10.1002/anie.202411078.
(5) Biebl, S. M.; Ziemann, P.; Ströbele, M.; Bettinger, H. F. Mechanistic insights into the thermal ring opening of the Dewar isomer of 1,2-Dihydro-1,2-azaborines. *JACS Au* **2025**, *submitted*.
(6) Kleemiss, F.; Dolomanov, O. V.; Bodensteiner, M.; Peyerimhoff, N.; Midgley, L.; Bourhis, L. J.; Genoni, A.; Malaspina, L. A.; Jayatilaka, D.; Spencer, J. L.; et al. Accurate crystal structures and chemical properties from NoSpherA2. *Chemical Science* **2021**, *12* (5), 1675-1692, 10.1039/D0SC05526C. DOI: 10.1039/D0SC05526C.
(7) Zhao, Y.; Truhlar, D. G. The M06 suite of density functionals for main group thermochemistry, thermochemical kinetics, noncovalent interactions, excited states, and transition elements: two new functionals and systematic testing of four M06-class functionals and 12 other functionals. *Theoretical Chemistry Accounts* **2008**, *120* (1), 215-241. DOI: 10.1007/s00214-007-0310-x.
(8) Ditchfield, R.; Hehre, W. J.; Pople, J. A. Self-Consistent Molecular-Orbital Methods. IX. An Extended Gaussian-Type Basis for Molecular-Orbital Studies of Organic Molecules. *The Journal of Chemical Physics* **1971**, *54* (2), 724-728. DOI: 10.1063/1.1674902 (acccessed 2/18/2025).
(9) Hehre, W. J.; Ditchfield, R.; Pople, J. A. Self—Consistent Molecular Orbital Methods. XII. Further Extensions of Gaussian—Type Basis Sets for Use in Molecular Orbital Studies of Organic Molecules. *The Journal of Chemical Physics* **1972**, *56* (5), 2257-2261. DOI: 10.1063/1.1677527 (acccessed 2/18/2025).
(10) Francl, M. M.; Pietro, W. J.; Hehre, W. J.; Binkley, J. S.; Gordon, M. S.; DeFrees, D. J.; Pople, J. A. Self-consistent molecular orbital methods. XXIII. A polarization-type basis set for second-row elements. *The Journal of Chemical Physics* **1982**, *77* (7), 3654-3665. DOI: 10.1063/1.444267 (acccessed 2/18/2025).
(11) Neese, F. The ORCA program system. *WIREs Computational Molecular Science* **2012**, *2* (1), 73-78. DOI: https://doi.org/10.1002/wcms.81.
(12) McLean, A. D.; Chandler, G. S. Contracted Gaussian basis sets for molecular calculations. I. Second row atoms, Z=11–18. *The Journal of Chemical Physics* **1980**, *72* (10), 5639-5648. DOI: 10.1063/1.438980 (acccessed 2/9/2025).
(13) *Gaussian 16 Rev. C.01*; Wallingford, CT, 2016. (accessed.

(1) Sheldrick, G. M., *Acta Crystallogr., Sect. A* **2008**, *2008*, 112-122,
(2) Dittrich, C. B. H. G. M. S. B., ShelXle: a Qt graphical user interface for SHELXL. *J. Appl. Crystallogr.* **2011**, *44*, 10.1107/S0021889811043202.
(3) Neese, F.; Wennmohs, F.; Becker, U.; Riplinger, C., The ORCA quantum chemistry program package. *The Journal of Chemical Physics* **2020**, *152* (22), 224108, 10.1063/5.0004608.
(4) Neese, F., Software update: The ORCA program system—Version 5.0. *WIREs Computational Molecular Science* **2022**, *12* (5), e1606, https://doi.org/10.1002/wcms.1606.
(5) Grimme, S.; Brandenburg, J. G.; Bannwarth, C.; Hansen, A., Consistent structures and interactions by density functional theory with small atomic orbital basis sets. *The Journal of Chemical Physics* **2015**, *143* (5), 054107, 10.1063/1.4927476.
(6) Helmich-Paris, B.; de Souza, B.; Neese, F.; Izsák, R., An improved chain of spheres for exchange algorithm. *The Journal of Chemical Physics* **2021**, *155* (10), 104109, 10.1063/5.0058766.
(7) Ásgeirsson, V.; Birgisson, B. O.; Bjornsson, R.; Becker, U.; Neese, F.; Riplinger, C.; Jónsson, H., Nudged Elastic Band Method for Molecular Reactions Using Energy-Weighted Springs Combined with Eigenvector Following. *Journal of Chemical Theory and Computation* **2021**, *17* (8), 4929-4945, 10.1021/acs.jctc.1c00462.
(8) Bannwarth, C.; Ehlert, S.; Grimme, S., GFN2-xTB—An Accurate and Broadly Parametrized Self-Consistent Tight-Binding Quantum Chemical Method with Multipole Electrostatics and Density-Dependent Dispersion Contributions. *Journal of Chemical Theory and Computation* **2019**, *15* (3), 1652-1671, 10.1021/acs.jctc.8b01176.
(9) Zhang, Y.; Yang, W., Comment on ``Generalized Gradient Approximation Made Simple''. *Phys. Rev. Lett.* **1998**, *80* (4), 890-890, 10.1103/PhysRevLett.80.890.
(10) Adamo, C.; Barone, V., Toward reliable density functional methods without adjustable parameters: The PBE0 model. *The Journal of Chemical Physics* **1999**, *110* (13), 6158-6170, 10.1063/1.478522.
(11) Grimme, S.; Ehrlich, S.; Goerigk, L., Effect of the damping function in dispersion corrected density functional theory. *J. Comput. Chem.* **2011**, *32* (7), 1456-1465, https://doi.org/10.1002/jcc.21759.
(12) Weigend, F.; Ahlrichs, R., Balanced basis sets of split valence, triple zeta valence and quadruple zeta valence quality for H to Rn: Design and assessment of accuracy. *Physical Chemistry Chemical Physics* **2005**, *7* (18), 3297-3305, 10.1039/B508541A.
